# Phytochemical Characterization and Chemotherapeutic Potential of *Cinnamomum verum* Extracts on the Multiplication of Protozoan Parasites In Vitro and In Vivo

**DOI:** 10.3390/molecules25040996

**Published:** 2020-02-24

**Authors:** Gaber El-Saber Batiha, Amany Magdy Beshbishy, Azirwan Guswanto, Arifin Nugraha, Tserendorj Munkhjargal, Mohamed M. Abdel-Daim, Juan Mosqueda, Ikuo Igarashi

**Affiliations:** 1 National Research Center for Protozoan Diseases, Obihiro University of Agriculture and Veterinary Medicine, Nishi 2-13, Inada-cho, Obihiro, Hokkaido 080-8555, Japan; amanimagdi2008@gmail.com (A.M.B.); guswanto7@gmail.com (A.G.); arifin.budiman.fkh@gmail.com (A.N.); joel.mosqueda@uaq.mx (J.M.); igarcpmi@obihiro.ac.jp (I.I.); 2 Department of Pharmacology and Therapeutics, Faculty of Veterinary Medicine, Damanhour University, Damanhour, Al Beheira 22511, Egypt; 3 Laboratory of Helminthology, Institute of Veterinary Medicine, Zaisan−17042, Ulaanbaatar, Mongolia; tserenmunh@gmail.com; 4 Department of Zoology, College of Science, King Saud University, P.O. Box 2455, Riyadh 11451, Saudi Arabia; abdeldaim.m@vet.suez.edu.eg; 5 Pharmacology Department, Faculty of Veterinary Medicine, Suez Canal University, Ismailia 41522, Egypt; 6 Facultad de Ciencias Naturales, Universidad Autónoma de Querétaro, Avenida de las Ciencias s/n, Juriquilla 76230, Querétaro, Mexico

**Keywords:** *Cinnamomum verum*, antipiroplasm drugs, phytochemical estimation, bioactive constituents

## Abstract

*Cinnamomum verum* is a commonly used herbal plant that has several documented properties against various diseases. The existing study evaluated the inhibitory effect of acetonic extract of *C. verum* (AECV) and ethyl acetate extract of *C. verum* (EAECV) against piroplasm parasites in vitro and in vivo. The drug-exposure viability assay was tested on Madin-Darby bovine kidney (MDBK), mouse embryonic fibroblast (NIH/3T3) and human foreskin fibroblast (HFF) cells. Qualitative phytochemical estimation revealed that AECV and EAECV containing multiple bioactive constituents namely alkaloids, tannins, saponins, terpenoids and remarkable amounts of polyphenols and flavonoids. AECV and EAECV inhibited *B. bovis*, *B. bigemina*, *B. divergens*, *B. caballi*, and *T. equi* multiplication at half-maximal inhibitory concentrations (IC_50_) of 23.1 ± 1.4, 56.6 ± 9.1, 33.4 ± 2.1, 40.3 ± 7.5, 18.8 ± 1.6 µg/mL, and 40.1 ± 8.5, 55.6 ± 1.1, 45.7 ± 1.9, 50.2 ± 6.2, and 61.5 ± 5.2 µg/mL, respectively. In the cytotoxicity assay, AECV and EAECV affected the viability of MDBK, NIH/3T3 and HFF cells with half-maximum effective concentrations (EC_50_) of 440 ± 10.6, 816 ± 12.7 and 914 ± 12.2 µg/mL and 376 ± 11.2, 610 ± 7.7 and 790 ± 12.4 µg/mL, respectively. The in vivo experiment showed that AECV and EAECV were effective against *B. microti* in mice at 150 mg/kg. These results showed that *C. verum* extracts are potential antipiroplasm drugs after further studies in some clinical cases.

## 1. Introduction

Theileriosis and babesiosis are tick-transmitted diseases with significant economic impacts in the bovine and equine industries all over the world [1]. *Babesia divergens* and *B. microti* mainly affect cattle and rodents, respectively, and reveal zoonotic significance [2]. *Babesia divergens* is the main reason for babesiosis in Europe, mainly in immunocompromised humans, while *B. microti* is the main known etiologic agent responsible for human babesiosis in the USA. *Babesia bovis*, as well as *B. bigemina*, are the most pathogenic agents in cattle [1], while *Theileria equi* and *Babesia caballi* are considered the most devastating infections in horses. Horses and cattle that survive acute infections may become permanent sources for continuous transmission [3].

The prevention and control of babesiosis and theileriosis rely on vector control, the use of vaccines, and drugs available on the market [3,4]. Unfortunately, only a few drugs are available, such as a combination of clindamycin and quinine, atovaquone-azithromycin, diminazene aceturate (DA), and imidocarb dipropionate [5]. However, some human cases showed resistance to atovaquone and azithromycin combination, moreover, imidocarb dipropionate has shown strong toxicity to the host, and DA–resistant parasites have emerged [2,6]. Although several studies have documented the effectiveness of antipiroplasmic herbal extracts such as *Syzygium aromaticum* methanolic extract [7], methanolic *Cuminum cyminum* seeds extract and acetonic *Myrtus communis* roots [8], and methanolic *Olea europaea* and acetonic *Acacia laeta* [9] against the growth of piroplasm parasites, none of these extracts are used by veterinarians for the treatment of clinical cases [1]. Notably, there is an urgent and continuing need to identify alternative drugs to treat piroplasmosis.

Over the past few decades, research on herbal plants has provided modern medicine with several useful chemical ingredients that have been used to manage various ailments. However, many people in developing countries, especially in Africa and Asia, still rely on crude herbal extracts to treat several human and animal ailments [10,11]. This is partly because these extracts are inexpensive and easily accessible [12]. *Cinnamomum* genus consists of thousands of species that are distributed all over the world and is considered as one of the most important and popular spices used in cooking as well as traditional and modern medicines [13]. *Cinnamomum verum*, *C*. *osmophloeum*, *C. cassia*, *C. camphora*, *C. burmannii*, *C. loureiroi* and *C. zeylanicum* are the main economically important *Cinnamomum* species. Interestingly, they are commercially used for many medicinal purposes and in the perfume industry and can be included in various types of foodstuffs. Several reports have documented antiseptic, antiviral, antibacterial, antifungal, antidiabetic, hypocholesteremic, carminative, astringent, and blood purifier activities [13,14]. Recently, Zhang et al. [15] reported that *C. cassia* possesses many pharmacological activities, including antibacterial, anti-inflammatory, antitumor, anti-obesity, analgesic, antidiabetic, antiviral, cardiovascular protective, neuroprotective, cytoprotective and immunoregulatory properties. The effective role of cinnamon in the inhibition of different diseases is attributed to the presence of various chemical constituents in high concentrations. For instance, camphor is one of the significant bioactive molecules extracted from *C. camphora* that has been used in pharmaceuticals, mainly liniments and insecticides and shows antidiabetic affect [16]. Barros et al. [17] documented that essential oils extracted from *C. zeylanicum* bark, significantly enhanced the osmotic fragility curve of red blood cells (RBCs), and promoting toxic effects on RBCs membranes. Another research on *C. osmophloeum* (*C*. *osmophloeum*) indicated that the essential oil from cinnamon leaves contains a high concentration of cinnamaldehyde. (E)-cinnamaldehyde is considered one of the main bioactive molecules of essential oil extracted from *C. zeylanicum* that is responsible for its activity [14]. Cinnamaldehyde is the major constituent of *Cinnamomum* spices that possesses anaesthetic, antipyretic, antiallergic, metal ion chelating, lipo-protective, antioxidant, antibacterial and antiproliferative activity against several human cancer cell lines including leukemia, ovarian, breast (T47D) and lung (NCI-H322) cancer cell lines by inhibiting cancer progression [18].

*Cinnamomum verum* is a plant commonly used in traditional medicine. *C. verum* belongs to the genus *Cinnamomum*, which includes about 250 species of plants distributed worldwide, especially in parts of Africa and Asia [13]. *C. verum* has several medicinal properties, such as anti-inflammatory, hypoglycemic, antibacterial, antioxidant, spasmolytic, antidiarrheal, antifungal, antitumor, analgesic, gastroprotective, anticancer, and anthelmintic ones [14,19,20]. Interestingly, recent studies have documented the antimalarial efficacy of *C. verum* extract [21,22]. However, to our knowledge, no data has been reported to show the efficacy of *C. verum* herbal extracts against the growth of piroplasm parasites. *C. verum* has shown several medicinal properties because it contains many phytochemical ingredients, such as cinnamic acid, cinnamaldehyde, cinnamate, and numerous polyphenols [23]. More recently, Kwan et al. [24] identified new compounds in ethanolic *C. verum* extract, such as chlorogenic acid, catechin, protocatechuic acid, icariin, aesculetin, and quercetin. Therefore, it is important to use an extraction method that can harvest the effective phytochemical molecules from the *C. verum* plant. Unfortunately, extraction solvents that include methanol, ethanol, acetone, and ethyl acetate have shown variation in the amount and types of bioactive molecules harvested [25]. This study aimed to assess the effectiveness of acetonic extract of *C. verum* (AECV) and ethyl acetate extract of *C. verum* (EAECV) against the growth of *B. bovis*, *B. bigemina*, *B. divergens*, *B*. *caballi* and *T. equi* using the in vitro fluorescence assay. Furthermore, the chemotherapeutic potential of AECV and EAECV on *B. microti* in mice was investigated.

## 2. Results

### 2.1. Plant Extraction, Phytochemical and Total Phenolic and Flavonoid Contents Evaluation of C. verum Extracts

The yield percentage of the *C. verum* extracts was 8.34% *w*/*w* dry matter and dark in color. Preliminary examination of AECV and EAECV pointed to the existence of different phytoconstituents such as tannins, saponins, alkaloids and terpenoids that may be responsible for their pharmacological activities. Considerable amounts of phenolic and flavonoid contents were observed in AECV and EAECV. Notably, AECV (76.5 ± 0.94 mg of GAE/g DW) showed higher total phenolic content than EAECV (64.4 ± 1.57 mg of GAE/g DW). Moreover, AECV (43.7 ± 1.8 mg of CAE/g DW) had the higher total flavonoid content than EAECV (40.3 ± 0.9 mg of CAE/g DW).

### 2.2. Gas Chromatography-Mass Spectrometry (GC-MS) Analysis

The GC-MS analysis of AECV and EAECV revealed the existence of 17 and 26 phytochemical compounds, respectively. The identified chemical composition of AECV is shown in Table 1 and Figure S1 and represented 17 compounds, while the identified chemical composition of EAECV is shown in Table 2 and Figure S2 and represented 26 compounds.

**Figure 1:**
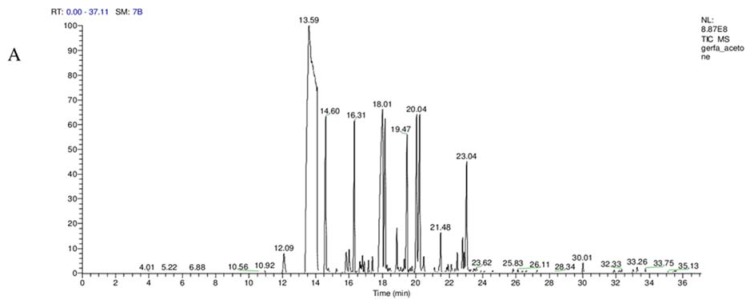

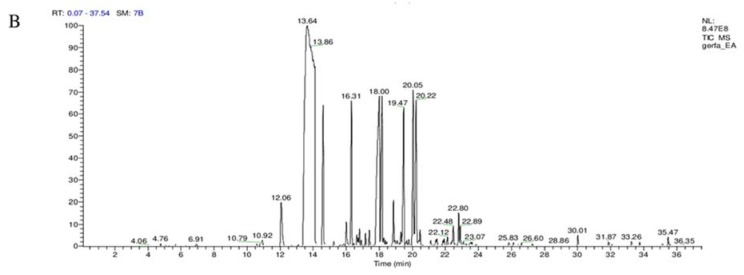
Gas chromatography-mass spectrometry analysis in the AECV (**A**) and EAECV (**B**).

The phytochemical compounds’ identification was established on the basis of the peak area, and retention time. The active principles with their retention time (RT) and percentage of peak area (%) are expressed in Figure 1A,B.

### 2.3. The Growth Inhibitory Effect of AECV and EAECV In Vitro

**Figure 2:**
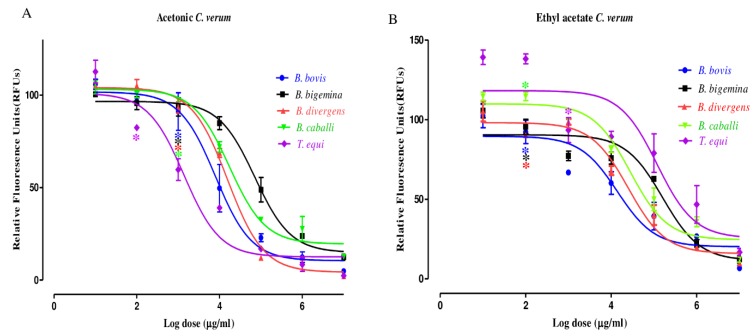
The dose-response curves acetonic extract of *C. verum* (AECV) (**A**) and ethyl acetate extract of *C. verum* (EAECV) (**B**) against *Babesia* and *Theileria* parasites in vitro. The curves showing the growth inhibition of *B. bovis*, *B. bigemina*, *B. divergens, B. caballi* and *T. equi* treated with various concentrations of AECV and EAECV. The result was determined by the fluorescence assay after 96 h of incubation. The values obtained from three separate trials were used to determine the IC_50_’s using the non-linear regression (curve fit analysis) in GraphPad Prism software (GraphPad Software Inc. San Diego, CA, USA).

The preliminary evaluation of *C. verum* extracts was performed to detect their efficacy on bovine and equine RBCs. The proliferation of *B. bigemina* and *B. caballi* did not significantly differ between AECV- and EAECV-treated RBCs and untreated RBCs for the two tested species (data not shown). For the growth-inhibitory effect, AECV (Figure 2A) and EAECV (Figure 2B) affected the multiplication of *B. bovis*, *B. bigemina*, *B. divergens*, *B. caballi*, and *T. equi* in a dose-dependent manner. *B. bovis* multiplication was suppressed significantly (*t*-test: *t*_(6)_ = 2.412, *p* < 0.0001) at 6.25 µM AECV, whereas *B. divergens*, *B. bigemina* and *B. caballi* multiplication was suppressed significantly (t-test: *t*_(6)_ = 1.765, *p* < 0.0001) at 6.25 µM AECV and *T. equi* multiplication was suppressed significantly (t-test: *t*_(6)_ = 3.583, *p* < 0.0001) at 3.125 µM AECV (Figure 2A). EAECV inhibited the in vitro multiplication of *B. bovis* (*t*-test: *t*_(6)_ = 3. 589, *p* = 0.01), *B. bigemina* and *B. divergens* significantly (*t*-test: *t*_(6)_ = 1.960, *p* = 0.001) at 3.125 µM. EAECV inhibited the multiplication of *B. caballi* and *T. equi* significantly (*t*-test: *t*_(6)_ = 0.919, *p* < 0.0001) at 3.125, 6.25 µM, respectively (Figure 2B).

AECV and EAECV exhibited IC_50_ values of 23.1 ± 1.4, 56.6 ± 9.1, 33.4 ± 2.1, 40.3 ± 7.5, and 18.8 ± 1.6 µg/mL and 40.1 ± 8.5, 55.6 ± 1.1, 45.7 ± 1.9, 50.2 ± 6.2, and 61.5 ± 5.2 µg/mL against *B. bovis*, *B. bigemina*, *B. divergens*, *B. caballi*, and *T. equi*, respectively (Table 3). The IC_50_ values of DA, methanolic *Syzygium aromaticum* (MESA) and methanolic *Olea europaea* extracts (MEOE) that inhibited the multiplication of *B. bovis*, *B. bigemina*, *B. divergens*, *B. caballi* and *T. equi* were 0.25, 0.11, 0.35, 0.003, and 0.37 µg/mL and 109.8 ± 3.8, 8.7 ± 0.09, 76.4 ± 4.5, 19.6 ± 2.2, and 60 ± 7.3μg/ mL and 107.1 ± 9.2, 47.7 ± 2.3, 101.1 ± 8.9, 105.5 ± 11.1, 19.3 ± 2.1 µg/mL, respectively (Table S1).

### 2.4. The Viability of Parasites Treated with AECV and EAECV and Morphological Changes of Treated Parasites

In the presence of 2 × IC_50_ EAECV, the regrowth of *B. bovis*, *B. bigemina*, *B. divergens*, *B. caballi*, and *T. equi* was completely suppressed. AECV at 2 × IC_50_ completely suppressed the regrowth of *B. bovis* and *B. divergens*, while *B. caballi*, *B. bigemina*, and *T. equi* could not regrow at 4 × IC_50_ (Table 4).

**Figure 3:**
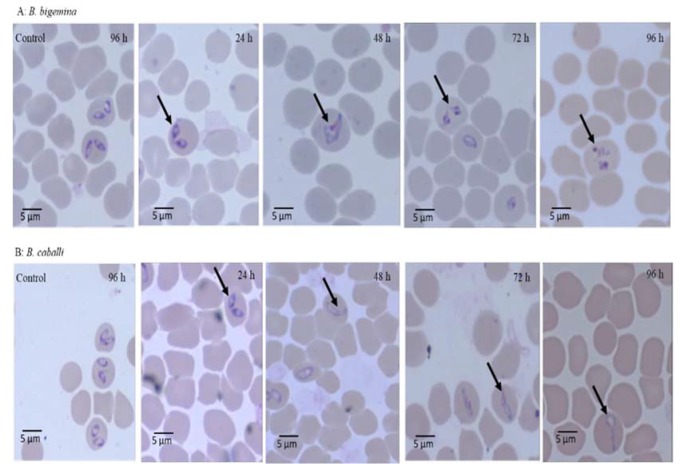
Morphological changes observed in ethyl acetate *C. verum* extract-treated *B. bigemina* and *B. caballi*. Light micrographs of ethyl acetate *C. verum* extract-treated *B. bigemina* and *B. caballi* in an in vitro culture taken after 24, 48, 72, and 96 h. The arrows show the spindle shapes of dividing parasites at 24 and 48 h, while at 72 and 96 h, dot-shaped remnants of dying parasites were observed as compared to the piriform shape of normal *B. bigemina* and *B. caballi* (control).

**Figure 4:**
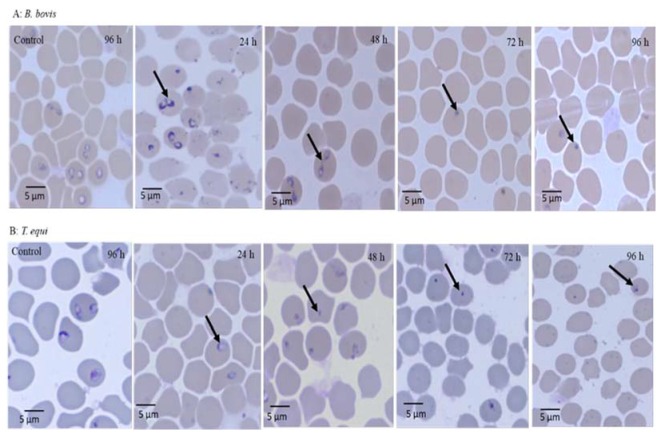
Morphological changes observed in acetonic *C. verum* extract-treated *B. bovis* and *T. equi*. Light micrographs of acetonic *C. verum* extract-treated *B. bovis* and *T. equi* in an in vitro culture taken after 24, 48, 72, and 96 h. *B. bovis*–treated parasites at 24 and 48 h appear spindle-shaped, while at 72 and 96 h, they appear dot-shaped as compared to the normal piriform shape of *B. bovis* (control). *T. equi*–treated parasites at 24 and 48 h appear smaller and pyknotic, while at 72 and 96 h, the dying parasites appeared dot-shaped as compared to the oval shape of *T. equi* (control).

The micrographs of EAECV-treated *B. bigemina* and *B. caballi* and of AECV-treated *B. bovis* and *T. equi* are shown in Figure 3 and Figure 4, respectively. At 24 and 48 h, all captured micrographs revealed spindle shapes dividing parasites compared to the piriform shape of untreated *B. bovis*, *B. bigemina*, and *B. caballi*, while dot-shaped residues of deadly parasites were observed within the erythrocytes at 72 and 96 h.

At 24 and 48 h, *T. equi* parasites treated with AECV were smaller and pyknotic compared to the oval form of the untreated parasites. In subsequent micrographs taken at 72 and 96 h, the dying parasites appeared dot-shaped.

### 2.5. Toxicity of AECV and EAECV on Normal Cells

The cytotoxicity of *C. verum* extracts on host cells were estimated using MDBK, NIH/3T3, and HFF cell lines. AECV and EAECV showed EC_50_ values of 440 ± 10.6, 816 ± 12.7 and 914 ± 12.2 µg/L and 376 ± 11.2, 610 ± 7.7 and 790 ± 12.4 µg/mL on MDBK, NIH/3T3, and HFF cell lines, respectively. DA did not affect the viability of MDBK, NIH/3T3, and HFF cell viability even at 100 µg/mL, while MESA and MEOE affected the viability of MDBK, NIH/3T3, and HFF cell lines at EC_50_ 894.7 ± 4.9, >1000 and >1000 µg/mL and 794.7 ± 41.9, ˃1500 and ˃1500 µg/mL, respectively (Additional file 1: Table S1). The highest selectivity indexes (the ratio of cell line EC_50_ to the parasite IC_50_) of the AECV and EAECV were found to be 23.4 times higher than the IC_50_ on *T. equi*, and 9.4 times higher than the IC_50_ on *B. bovis*, respectively (Table 3).

### 2.6. Combination Effect of Extracts In Vitro

Combinations of AECV and EAECV with DA and *S. aromaticum* and *O. europaeae* methanolic extracts were assessed in the in vitro cultures of *B. bovis*, *B. bigemina*, *B. divergens*, *B. caballi*, and *T. equi*. The combined treatment of AECV and EAECV with DA (Table 5) and MESA (Table 6) and MEOE (Table 7) was synergistic and additive against *B.* *bovis*, *B. bigemina*, *B. divergens*, *B. caballi*, and *T. equi*.

### 2.7. The In Vivo Chemotherapeutic Effect of AECV and EAECV

**Figure 5:**
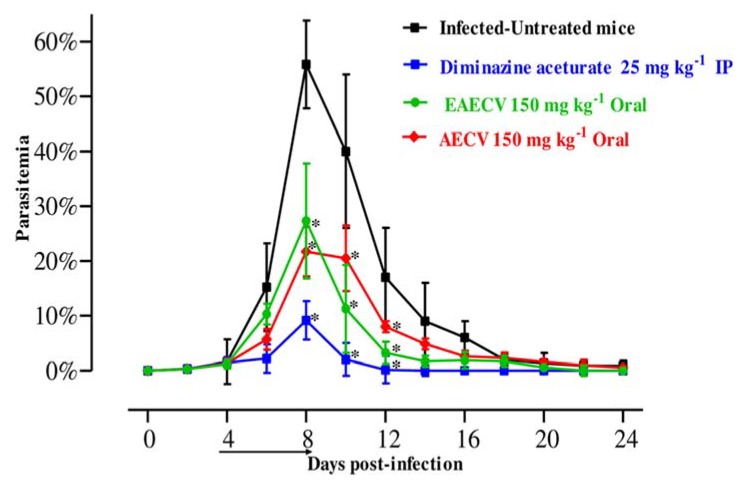
Growth inhibition of *C. verum* extracts on *B. microti* in mice. Inhibitory effect of *C. verum* extracts on the growth of *B. microti* in mice, based on observations taken from five mice per experimental group. EAECV, ethyl acetate *C. verum* extract; AECV, acetonic *C. verum* extract. The arrow indicates 5 consecutive days of treatment. Asterisks indicate statistically significant (*p* < 0.05) differences of parasitemia between treated groups and the untreated control group based on one-way ANOVA Tukey’s test, available in the GraphPad Prism software. Parasitemia was calculated by counting infected RBCs among 2000 RBCs using Giemsa-stained thin blood smears. The data were the mean and standard deviation from two separate experiments.

To examine the chemotherapeutic effects of EAECV and AECV in vivo, *B. microti*-infected BALB/c mice were treated with EAECV and AECV for five days after infection reach 1% parasitemia. Control group treated with double-distilled water (DDW) exhibited rapid growth of parasitemia reached 55.8% on day 8 post-infection (p.i.) and the parasitemia decreased gradually on the following days. In groups treated with EAECV or AECV, the level of parasitemia was cleared at a statistically significant lower percentage of parasitemia than that of the control group (ANOVA: *F*_(1.988, 32.74)_ = 2.917, *p* < 0.0001 for 150 mg/kg AECV; ANOVA: *F*_(2, 32.02)_ = 2.945, *p* < 0.0001 for 150 mg/kg EAECV) from days 6 to 12 p.i. Among all treated mice, the peak parasitemia level was 21.7% and 27.3% in 150 mg/kg AECV and EAECV, respectively on day 8 p.i. The peak parasitemia level was 9.1% in 25 mg/kg DA (Figure 5).

**Figure 6:**
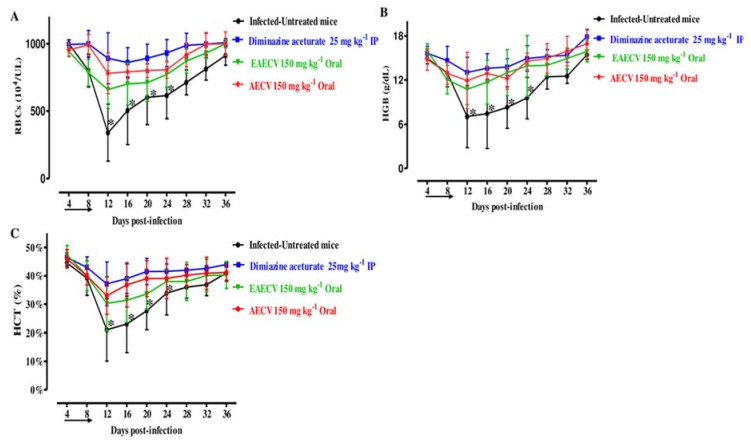
RBCs, hemoglobin, and hematocrit changes in *C. verum* extract-treated mice. Graphs showing changes in the number of red blood cells (RBCs) (**A**), hemoglobin concentration (HGB) (**B**), and hematocrit percentage (HCT) (**C**) in mice treated with diminazene aceturate and two *C. verum* extracts. EAECA, ethyl acetate *C. verum* extract; AECA, acetonic *C. verum* extract. The arrow indicates 5 consecutive days of treatment. Asterisks indicate statistical significance (*p* < 0.05) based on one-way ANOVA Tukey’s test, available in the GraphPad Prism software. The data were the mean and standard deviation from two separate experiments (five mice per group).

The comparison of the hematology parameters during in vivo studies showed no significant difference in RBCs (Figure 6A), hemoglobin (Figure 6B), or hematocrit percentage (Figure 6C) for the groups treated with AECV or EAECV as compared to the DA-treated group on days 8 and 12. While statistically significant differences in the RBCs (ANOVA: *F*_(3, 12.02)_ = 6.015, *p* < 0.002), HGB concentration (ANOVA: *F*_(2.984, 11.61)_ = 4.978, *p* < 0.006), and HCT (ANOVA: *F*_(3.103, 10.47)_ = 3.843, *p* < 0.01) was detected between the drug-treated groups and DDW group on days 8 and 12.

## 3. Discussion

The current study documents the efficacy of various *C. verum* extracts, namely, acetonic and ethyl acetate against the in vitro growth of *B. bovis*, *B. bigemina*, *B. divergens*, *B. caballi*, and *T. equi* in vitro and on *B. microti* in vivo. Initially, AECV and EAECV were examined for the existence of various biologically active compounds and the preliminary and qualitative screening emphasized the presence of significant amounts of polyphenols, terpenoids, alkaloid, flavonoids, and tannin. It has been shown that all these secondary metabolites have many therapeutic and antiprotozoal properties against several parasites namely *Plasmodium*, *Leishmania*, *Trypanosoma*, *Schistosoma* and *Trichomonas Vaginalis* and they are known to be pharmacologically active components [26].

The GC-MS analysis was used to identify and detect phytochemical constituents present in AECV and EAECV. The analysis revealed that AECV consisted of 17 compounds and the main chemical components detected were (*E*)-cinnamaldehyde (52.87%), chromen-2-one (10.63%), *o*-methoxycinnamaldehyde, (5.04%), γ-muurolene (4.92%), cadina-1(10),4-diene (4.64%) and acetic acid cinnamyl ester (4.35%), while EAECV was found to possess 26 compounds and the main chemical components identified were (*E*)-cinnamaldehyde (53.81%), coumarin (9.92%), γ-muurolene (5.37%), *p*-methoxycinnamaldehyde, (4.91%), acetic acid cinnamyl ester (4.83%), cadina-1(10),4-diene (4.78%) and cinnamyl alcohol (4.27%). Our results indicating that EAECV consist of slightly higher percent of (*E*)-cinnamaldehyde, γ-muurolene, and acetic acid cinnamyl ester, suggesting that ethyl acetate is the better solvent of extraction. This finding is consistent with Dvorackova et al. [25] who revealed that different extraction solvents have shown variation in the amount and types of bioactive molecules harvested.

AECV and EAECV showed varying levels of effectiveness against all tested species. For instance, AECV was most effective against *B. bovis*, *B. divergens*, *B. caballi* and *T. equi*, while EAECV was most effective against *B. bigemina.* Different *C. verum* extracts exhibited varying IC_50_ values against *Babesia* and *Theileria* species, which implies that the mode of extraction is a key determinant of the effectiveness or concentration of active molecules in the final extract. Moreover, the preliminary and qualitative screening results showed that AECV contains higher total phenolic and flavonoid content than EAECV and these results explaining the highest antipiroplasmic and inhibitory effect of AECV. This finding was consistent with a previous study performed by Nkanwen et al. [21] who tested ethyl acetate/*n-*hexane *C. verum* extract for anti-plasmodial efficacy and showed an inhibitory effect on the *Plasmodium falciparum* enoyl-ACP reductase enzyme. More recently, Parvazi et al. [22] showed that aqueous *C. verum* extract possesses inhibitory activity against *P. falciparum* in vitro, by alteration of the following metabolites: l-aspartic acid, succinic acid, β-alanine, glutathione, and 2-methylbutyryl glycine.

Recently, several studies documented the antiprotozoal activities of different phytochemical molecules detected by our GC-MS analysis. For example, von Son-de Fernex et al. [27] and Williams et al. [28] documented the in vitro anthelmintic activity of 2*H*-chromen-2-one and cinnamaldehyde, the main chemical component in AECV and EAECV against *Cooperia Punctata* and the swine nematode *Ascaris suum*, whereas Kpadonou et al. [29] disclosed the antitrypanosomal efficacy of caryophyllene oxide against *Trypanosoma brucei brucei* with IC_50_ value of 17.67 µg/mL. Moreover, Bosquiroli et al. [30] proved antileishmanial activity of caryophyllene and caryophyllene oxide against promastigotes of *L. infantum*. In addition to that, Le et al. [31] reported the anti-protozoal activity of *C. verum* essential oil and its constituents (cinnamaldehyde and caryophyllene) against *Leishmania*, *Plasmodium* and *Trypanosoma*. Therefore, we hypothesized that cinnamaldehyde, caryophyllene, 2*H*-chromen-2-one, and caryophyllene oxide are the main active compounds responsible for the antipiroplasmic activity of *C. verum* extracts.

The viability assay showed that AECV and EAECV were effective against *Babesia* and *Theileria* parasites. This result was comparable with that of Parvazi et al. [22] who showed that aqueous *C. verum* extract inhibited the growth of *P. falciparum* by preventing amino acid biosynthesis through inhibiting carbon dioxide fixation and disabling the synthesis of alanine, aspartame, and glutamate. Based on this finding, we suggest that *C. verum* extracts have multiple mechanisms against piroplasm parasites. Compared with previous studies on the viability of treated piroplasm parasites, EAECV suppressed the regrowth of *Babesia* and *Theileria* parasites at lower concentrations than the methanolic extract of *O. europaea* and chalcone hydrate [6,9]. Furthermore, AECV suppressed the regrowth of *B. bovis* and *B. divergens* at lower concentrations than the methanolic extract of *S. aromaticum* [7]. Nevertheless, not all *C. verum* extracts completely inhibited the regrowth of piroplasm parasites at low concentrations. For instance, EAECV is able to clear all the parasites which indicate that the ethyl acetate extraction method harvested more bioactive molecules that can eliminate *Babesia* and *Theileria* parasites.

Morphological observations in micrographs of *C. verum*-treated parasites showed that the *C. verum* extract-treated *Babesia* and *Theileria* parasites were not able to eject and died inside the RBCs. Additionally, the malformations observed in the piroplasm parasites might be due to the ability of *C. verum* extracts to damage the parasite metabolome. The suggested mode of action for *P. falciparum* was reported previously by Parvazi et al. [22] who revealed that aqueous *C. verum* extract disrupts the thioredoxin and glutathione system, resulting in a change in the parasite metabolome, inhibiting its growth.

The experiments to determine cytotoxicity showed that *C. verum* extracts affected the viability of the MDBK, NIH/3T3, and HFF cell lines at a slightly high selectivity index. This means that the bioactive compounds that exist in *C. verum* extracts were more likely to affect piroplasm parasites than the host cells. *C. verum* extracts contain a mixture of polyphenols with cinnamaldehyde that plays a significant therapeutic role in cancer cell lines by depolarization of the mitochondrial membrane potential, leading to cellular apoptosis without cytotoxic activities on host cells [32]. One possible explanation of the modest selectivity index of AECV and EAECV is due to the presence of alkaloids and terpenoids [30,33]. The aforementioned results indicating the safety of *C. verum* extracts for the treatment of animals and humans following further clinical studies.

Drugs with a combination of *C. verum* extracts with DA, MESA, and MEOE were assessed in vitro against *B. bovis*, *B. bigemina*, *B. divergens*, *B. caballi*, and *T. equi*. The combined application of *C. verum* extracts with DA, MESA, and MEOE showed synergistic and additive effects. One possible explanation is that *C. verum* extracts contain many bioactive ingredients that may interact differently in combination treatments [23].

Acetonic and ethyl acetate *C. verum* extracts produced promising in vivo antibabesial activity. Oral administration of 150 mg/kg of AECV and EAECV resulted in 61.1% and 51.7% inhibition, respectively compared with 83.7% inhibition showed by 25 mg/kg DA on day 8 p.i. The growth inhibitory effects of AECV and EAECV on *B. microti* were lower than the 80 % inhibition revealed by 150 mg/kg MEOE, 64% inhibition shown by acetonic *Acacia laeta* extracts [9] and 69.2% shown by MESA [7]. However, the inhibitory efficacy of AECV on the multiplication of *B. microti* was higher than the 60% revealed by 150 mg/kg methanolic *Peganum harmala* seed extract, 55.1% inhibition noticed by 150 mg/kg ethanolic *Artemisia absinthium* leaf extract [34] and 42.4% inhibition shown by methanolic *Camellia sinensis* extract [7]. Furthermore, the inhibitory efficacy of EAECV on the multiplication of *B. microti* was higher than 42.4% shown by methanolic *Camellia sinensis* extract [7]. The chemosuppression effect produced by AECV and EAECV on *B. microti* may indicate the presence of potential compounds with higher antibabesial activity.

*C. verum* extracts have strong anti-inflammatory [15] and antioxidant effects and improve reduced glutathione synthesis in the liver [35,36]. Such medicinal effects are useful because piroplasmosis infection is related to reactive oxygen and nitrogen species overproduction, which leads to oxidative stress [37]. Thus, identifying the active compound is necessary for contriving a higher chemosuppression effect from these extracts for the future discovery of a novel potential drug against piroplasmosis. The limitation of this study is the use of the whole extract to confirm its antipiroplasmic efficacy, and it is recommended to assess the antipiroplasmic efficacy of the GC-MS identified compounds for the future discovery of a novel lead potential drug against piroplasmosis. As well, evaluate the actual mode of action employed against the recovery of piroplasm parasites.

## 4. Materials and Methods

### 4.1. Preparation of Extracts

#### 4.1.1. Chemicals and Solvents

The extraction solvents, namely, 99.5% acetone (Nacalai Tesque, Kyoto, Japan) and 99.5% ethyl acetate (Chameleon Reagent, Osaka, Japan), were used to obtain *C. verum* extracts. Stock solutions (10 mM) in dimethyl sulfoxide (DMSO) of DA (Ciba-Geigy Japan Limited, Tokyo, Japan) and *C. verum* extracts were stored at –30 °C and used for antibabesiosis evaluation. Aluminum chloride (AlCl_3_; Sigma-Aldrich, Tokyo, Japan) was dissolved in distillate water and used to detect the total flavonoid in the *C. verum* extracts. The SYBR Green I (SGI) nucleic acid stain (10,000×, Lonza, Alpharetta, GA, USA) was used for fluorescence assay after mixing with the lysis buffer consisting of Tris (130 mM at pH 7.5), EDTA (10 mM), saponin (0.016% *w*/*v*), and Triton X-100 (1.6% *v*/*v*) which was filtered using a 0.22 µm polyethersulfone and kept at 4 °C.

#### 4.1.2. Plant Material

Locally produced cinnamon bark *C. verum* (*Ceylon cinnamon*) was purchased from a spice store in Egypt, identified and voucher specimen number was placed by the members of the Department of Pharmacology and Chemotherapeutics, Faculty of Veterinary Medicine, Damanhour University, Egypt. The voucher specimen number of *C. verum* is A 0177107 (DPV). The bark was dried in an electric drying oven at 30 °C (Sanyo, Osaka, Japan) and pounded using a 60–80 mm mesh. The finely ground *C. verum* powder (100 g) was dissolved in 250 mL of each solvent (acetone and ethyl acetate) and then stirred at 25 °C for 72 h as described elsewhere [9,35]. The obtained solutions were filtered using Whatmann filter paper no. 1., concentrated using a rotary evaporator (RotavaporR-200/205, BUCHI^®^, Flawil, Switzerland) and lyophilized using a Freeze dry vacuum system (Labconco, Kansas City, MO, USA) [7,8]. Crude extracts were weighed followed by the addition of 1 mL of DMSO to 100 mg of the extract to be stored at −30 °C. The resulting extract weight was 8.34 g and the yield percentage was calculated using the following equation [38]:

(1) $$\mathrm{Percentage}\;\mathrm{yield}\;\mathrm{of}\;\mathrm{extracts}\;=\;\frac{\mathrm{Weight}\;\mathrm{of}\;\mathrm{extracted}\;\mathrm{material}}{\mathrm{Weight}\;\mathrm{of}\;\mathrm{original}\;\mathrm{plant}\;\mathrm{material}\;\mathrm{used}}\;\times \;100$$

#### 4.1.3. Phytochemical Examination of Plant Extracts

AECV and EAECV were examined for the existence of terpenoids, saponins, tannins, and alkaloids using several qualitative tests described previously [39].

#### 4.1.4. Determination of Total Phenolic Content

The concentration of total phenol present in AECV and EAECV was detected using the Folin-Ciocalteu (FC) reagent method as described elsewhere [40]. A volume of 0.5 mL of AECV and EAECV (1 mg/mL) was added to 1.5 mL of 10% FC reagent (diluted 1:10 with deionized water) and mixed for 5 min. After that, an aliquot (3 mL) of 7.5% Na_2_CO_3_ solution was added and further incubated at 30 °C for 2 h. Finally, the absorbance was calculated at 760 nm and the content of total phenolic compounds was detected from a gallic acid standard curve and expressed as mg/g gallic acid equivalent (GAE) of the dry weight of the extract (mg GAE/g DW).

#### 4.1.5. Determination of Total Flavonoid Content

Aluminum chloride (AlCl_3_) colorimetric method was used for the examination of total flavonoid content in AECV and EAECV as previously determined [40]. Briefly, an aliquot (1 mL) of AECV and EAECV was added to 3 mL of solvent extracts, 3.8 mL of distilled water, 200 µL of 1 M potassium acetate and 200 µL of 10% AlCl_3_ and incubated for 30 min. The absorbance was measured at 420 nm and the flavonoid content was detected from a catechin standard curve and expressed as mg/g catechin equivalents of the dry weight of individual extract (mg CAE/g DW).

#### 4.1.6. GC-MS Analysis

The chemical composition of AECV and EAECV was performed using a Trace GC-ISQ mass spectrometer (Thermo Scientific, Austin, TX, USA) with a direct capillary column TG–5MS (30 m × 0.25 mm × 0.25 µm film thickness) as previously described [1,41]. The column oven temperature was initially held at 50 °C and then increased by 5 °C/min to 250 °C withhold 1 min then increased to 300 at the rate of 30 °C /min. The injector temperatures were kept at 260 °C. Helium was used as a carrier gas at a constant flow rate of 1 mL/min. The solvent delay was 4 min and diluted samples of 1 µL were injected automatically using an AS3000 Autosampler coupled with GC in the split mode. EI mass spectra were collected at 70 eV ionization voltages over the range of *m*/*z* 50–650 in full scan mode. The ion source and transfer line were set at 250 °C and 270 °C, respectively. The components were identified by comparison of their retention times and mass spectra with those of WILEY 09 and NIST 11 mass spectral databases.

### 4.2. Parasites and Culture Conditions

*Babesia bigemina* Argentine strain, *B. bovis* Texas strain, *B. divergens* Germany strain, USDA strains of *B. caballi* and *T. equi*, and Munich strain of *B. microti* [41,42] were used in this study. The bovine and equine parasites used in this study were maintained in purified bovine or equine RBCs (collected from the animal farm of Obihiro University of Agriculture and Veterinary Medicine and stored at 4 °C) and incubated in a humidified chamber at 37 °C under an atmosphere of 5% CO_2_, 5% O_2_, and 90% N_2_ using a microaerophilic stationary-phase culture [6,7]. *Babesia bovis* and *B. bigemina* were grown in medium 199 (M199) supplemented with 40% bovine serum, *B. divergens* was grown in RPMI 1640 medium (Sigma-Aldrich) supplemented with 40% bovine serum, while *T. equi* was grown in M199 supplemented with 40% equine serum and 13.6 µg of hypoxanthine (ICN Biomedical Inc., Santa Ana, CA, USA) per mL. GIT medium supplemented with 40% equine serum was used for *B. caballi* cultivation. Amphotericin B (0.15 μg/mL), penicillin G (60 U/mL) and streptomycin (60 U/mL) (all from Sigma-Aldrich) were then prepared and added to all medium to prevent bacterial contamination.

### 4.3. Evaluation of the Impacts of AECV and EAECV on RBCs of Cattle and Horse

The hemolytic efficacy of AECV and EAECV on cattle and horse RBCs was evaluated in vitro as previously reported elsewhere [6,42]. Initially, AECV and EAECV extracts at 600 µg/mL were cultivated at 37 °C with purified bovine and equine RBCs for 3 h. Afterward, the pretreated-RBCs were washed three times with PBS and mixed with bovine and equine parasites. To monitor the parasitemia and any side effects as a result of the pretreatment, Giemsa-stained blood smears were prepared daily.

### 4.4. The In Vitro Growth Inhibitory Effects of AECV and EAECV

The in vitro *Babesia* fluorescent assay was carried out as previously reported elsewhere [42] to evaluate the inhibitory effects of AECV and EAECV. The stock supply of RBCs with 1% parasitemia was prepared by diluting parasite-infected RBCs (iRBCs) with uninfected RBCs. Briefly, in three separate trials, different concentrations of AECV and EAECV were prepared in the culture medium using two-fold dilution and added in 96-well plates in triplicate with 1% parasitemia and 5% HCT for *B. divergens*, *B. caballi* and *T. equi* and 2.5% HCT for *B. bovis* and *B. bigemina*. The positive control had DMSO at a final concentration of 0.3% and iRBCs, whereas uninfected RBCs and the culture medium served as the negative control. Subsequently, all parasites were incubated for 4 successive days without changing medium at 37 °C humidified multi-gas water-jacketed incubator in an atmosphere of 5% CO_2_, 5% O_2_, and 90% N_2_. On the fourth day of culture, an aliquot (100 µL) of lysis buffer containing 2 × SG1 was added to each well; thereafter, it was wrapped with aluminum foil to prevent direct exposure to light. After a 6-h incubation at room temperature, fluorescence readings were acquired on a spectrofluorimeter (Fluoroskan Ascent, Thermo Fisher Scientific, Oceanside, CA, USA) with excitation and emission wavelengths of 485 and 518 nm, respectively.

### 4.5. Viability Test and Morphological Changes

The viability studies were monitored via microscopy as described previously [6]. Briefly, an aliquot (20 µL) of iRBCs (1% parasitemia) was cultivated in 200 µL of media containing various concentrations of AECV and EAECV for 4 successive days, changing media daily. The concentrations used in this experiment were 0.25 ×, 0.5 ×, 1 ×, 2 ×, and 4 × the IC_50_. On the fifth day, a mixture of iRBCs (6 µL) from each well and fresh equine or bovine RBCs (14 µL) was transferred to a new 96-well plate, cultured in drug-free media and then left for an additional 8 days. The parasite prevalence was determined via light microscopy in order to evaluate parasite growth.

### 4.6. Cell Cultures

Cultures of Madin–Darby bovine kidney (MDBK) (ECACC), mouse embryonic fibroblast (NIH/3T3 ATCC^®^ CRL-1658™) and human foreskin fibroblast (HFF HFF-1 ATCC^®^ SCRC-1041™) cell lines were retrieved from −80 °C stock and cultured continuously in our laboratory under atmosphere 5% CO_2_ at 37 °C. The MDBK cell line was cultured in Minimum Essential Medium Eagle (MEM; Gibco, Grand Island, NY, USA), while NIH/3T3 and HFF cell lines were in Dulbecco Modified Eagle’s Medium (DMEM, Gibco). Each medium was treated with 1% glutamine, 10% fetal bovine serum, and 0.5% penicillin/streptomycin (Gibco). The medium was changed every 3 to 4 days, and once 80% confluence was reached, cells were harvested as per sub-culture protocol. To confirm the absence of mycoplasma contamination, 4,6-diamidino-2-phenylindole dihydrochloride (DAPI; Sigma-Aldrich, St. Louis, MO, USA) staining was used.

### 4.7. Cytotoxic Action of AECV and EAECV on Normal Cells

The cell viability assay was performed as described elsewhere [42,43]. Briefly, 100 µL of cells were seeded in a 96-well plate at a density of 5 × 10^4^ cells/mL in DMEM or MEM with fetal bovine serum and incubated overnight under atmosphere 5% CO_2_ at 37 °C for attachment. Aliquots (10 µL) of two-fold AECV and EAECV dilutions were added to each well to attain final concentrations of 15.8 to 1000 µM in triplicate and incubated for an additional 24 h. The wells with only a culture medium were used as a negative control, while the wells containing cells and the medium with 0.4% DMSO were used as a positive control. After 24 h, 10 µL of Cell Counting Kits-8 (CCK-8) was added, and the plate was incubated for another 3 h. The absorbance was measured using a microplate reader at 450 nm.

### 4.8. In Vitro Drug Combination Test

The combination therapies of AECV and EAECV with DA were evaluated using the fluorescence assay in the in vitro culture of *B. bovis*, *B. bigemina*, *B. divergens, B. caballi*, and *T. equi* as reported previously elsewhere [30]. Briefly, two-drug combinations of AECV and EAECV with DA, MESA and MEOE at five selected concentrations 0.25 ×, 0.5 ×, 1 ×, 2 ×, and 4 × the IC_50_ were added in 96 well-plates in duplicate. The drug cultivation and the fluorescence values were calculated after adding a lysis buffer containing 2 × SGI to each well of the 96-well plate as described above. Combination index (CI) values were calculated using CompuSyn software and the degree of synergism was determined as the weighted average of CI values using the formulae, ((1 × IC_50_) + (2 × IC_75_) + (3 × IC_90_) + (4 × IC_95_))/10 and results were explained using the reference CI scale; lower than 0.90 was considered synergism, between 0.90–1.10 was considered additive, while higher than 1.10 was considered antagonism developed previously [44,45].

### 4.9. In Vivo Chemotherapeutic Effects of AECV and EAECV

AECV and EAECV were evaluated for in vivo growth-inhibition assay using *B. microti*–infected BALB/c mice according to a procedure described elsewhere [6,7]. Twenty-five 8-week-old female BALB/c mice were housed under a pathogen-free environment with controlled temperature (22 °C) and humidity and a 12 h light/dark cycle and randomly distributed into five groups. The mice in groups 2 through 5 received 0.5 mL of inoculum (1 × 10^7^ *B. microti* iRBC) by intraperitoneal (i.p.) injection. Group 1 served as a negative control and was neither infected nor treated. At 1% parasitemia, drug treatment of the mice by i.p. started, continuing for five days. Group 2 served as a positive control and received 5% DMSO in DDW. Group 3 served as a reference drug control and received 25 mg/kg body weight (BW) of DA. Groups 4 and 5 received an oral administration of AECV and EAECV at 150 mg/kg, respectively. The levels of parasitemia were detected in all infected groups by microscopy using Giemsa-stained thin blood smears prepared every two days from venous tail blood until day 32 post-infection (p.i.).

The hematology profiles, including HCT percentage, HGB concentration, and number of RBCs were examined to detect the effect of *C. verum* extracts on the retrogression of anemia related to *Babesia* infection. The blood parameters were monitored by collecting 10 μL of mouse blood every 4 days using the Celltac α MEK-6450 automatic hematology analyzer (Celltac α MEK-6450, Nihon Kohden Corporation, Tokyo, Japan). After the in vivo inhibition assay, all mice were euthanized using an inhalation anesthesia system containing isoflurane by placing mice in the induction chamber, adjusting the oxygen flowmeter to 0.8 to 1.5 L/min and vaporizer to 3% to 5%. When mice were completely anesthetized, all of them were killed by cervical dislocation according to the ethical approval established by Fundamental Guidelines for Proper Conduct of Animal Experiment and Related Activities in Academic Research Institutions, the Ministry of Education, Culture, Sports and Technology (MEXT), Tokyo, Japan.

### 4.10. Ethical Statement

The in vivo studies were conducted based on the local guidelines for animal experimentation, as approved by the Obihiro University of Agriculture and Veterinary Medicine, Japan (accession numbers 28-111-2, 28-110, and 1417-2). This ethical approval was developed through the basic guidelines for the proper conduct of animal experimentation and related activities in Academic Research Institutions, Ministry of Education, Culture, Sports and Technology (MEXT), Japan.

### 4.11. Statistical Analysis

IC_50_ values of AECV, EAECV, DA, MESA, and MEOE was determined from the in vitro growth inhibition by nonlinear regression (curve fit) on a GraphPad Prism (GraphPad Software Inc., San Diego, CA, USA). The differences among group mean values on parasitemia and hematology profiles in the *B. microti*–infected mouse model were analyzed using a one-way ANOVA Tukey’s test in GraphPad Prism version 5.0. The difference was considered significant if *p* < 0.05 [46].

## 5. Conclusions

The current study showed the effectiveness of AECV and EAECV against the multiplication of several piroplasm parasites in vitro. *C. verum* extracts showed no apparent adverse effects in mice and showed efficacy in vitro, therefore, they could have potential value in treating clinical diseases caused by *Babesia* and *Theileria* in animals and humans. Taken together, these findings support that AECV and EAECV could be a potential source of alternate chemotherapy and discovering lead compounds that can be used for treatment of equine and bovine piroplasmosis as well as human babesiosis. However, further experiments are needed to evaluate the side effects of *C. verum* extracts on the histopathological and biochemical changes in different tissues and different times of treated mice.

## Figures and Tables

**Table 1:** The chemical composition of AECV by GC-MS.

Peak	R.t *	Name	Area %	Molecular Weight	Molecular Formula
1	12.09	(*Z*)-3-Phenylacrylaldehyde	0.82	132	C_9_H_8_O
2	13.59	(*E*)-Cinnamaldehyde	52.87	132	C_9_H_8_O
3	14.59	(*E*)-3-Phenyl-2-propen-1-ol	4.70	134	C_9_H_10_O
4	15.82	Cinnamic acid	0.69	148	C_9_H_8_O_2_
5	16.00	(+)-Cyclosativene	0.62	204	C_15_H_24_
6	16.31	α-Copaene	3.72	204	C_15_H_24_
7	16.80	(+)-Sativene	0.34	204	C_15_H_24_
8	18.01	Chromen-2-one	10.63	146	C_9_H_6_O_2_
9	18.15	Acetic acid cinnamyl ester	4.35	176	C_11_H_12_O_2_
10	19.47	γ-Muurolene	4.92	204	C_15_H_24_
11	20.04	Cadina-1(10),4-diene	4.64	204	C_15_H_24_
12	20.23	Cinnamaldehyde, *o*-methoxy-	5.04	162	C_10_H^10^O^2^
13	20.47	Calacorene	0.44	172	C_13_H_16_
14	21.48	1-Phenylbicyclo(4.1.0)heptane	1.05	172	C_13_H_16_
15	22.48	Epicubenol	0.41	222	C_15_H_26_O
16	22.80	T-Muurolol	0.67	222	C_15_H_26_O
17	23.04	6-Phenyl-3,5-hexadien-2-one	3.88	172	C_12_H_12_O

* R.t, retention time (min).

**Table 2:** The chemical composition of EAECV by GC-MS.

Peak	R.t *	Name	Area %	Molecular Weight	Molecular Formula
1	10.92	2-Methylbenzofuran	0.24	132	C_9_H_8_O
2	12.06	(*Z*)-3-Phenylacrylaldehyde	1.94	132	C_9_H_8_O
3	13.64	(*E*)-Cinnamaldehyde	53.81	132	C_9_H_8_O
4	14.60	Cinnamyl alcohol	4.27	134	C_9_H_10_O
5	16.00	(+)-Cyclosativene	0.62	204	C_15_H_24_
6	16.31	α-Copaene	3.68	204	C_15_H_24_
7	16.64	(+)-Sativene	0.70	204	C_15_H_24_
8	17.16	Isosativene	0.28	204	C_15_H_24_
9	17.39	Caryophyllene	0.38	204	C_15_H_24_
10	18.00	Coumarin	9.92	146	C_9_H_6_O_2_
11	18.16	Acetic acid, cinnamyl ester	4.83	176	C_11_H_12_O_2_
12	18.26	α-Caryophyllene	0.11	204	C_15_H_24_
13	19.30	Eremophila-1(10),11-diene	0.26	204	C_15_H_24_
14	19.47	γ-Muurolene	5.37	204	C_15_H_24_
15	19.66	α -Bisabolene	0.12	204	C_15_H_24_
16	20.04	Cadina-1(10),4-diene	4.78	204	C_15_H_24_
17	20.22	*p*-Methoxy-cinnamaldehyde	4.91	162	C_10_H_10_O_2_
18	20.47	α-Calacorene	0.43	200	C_15_H_20_
19	21.11	Caryophyllenyl alcohol	0.14	222	C_15_H_26_O
20	21.41	Caryophyllene oxide	0.14	220	C_15_H_24_O
21	21.48	γ-Elemene	0.16	204	C_15_H_24_
22	21.92	Globulol	0.14	222	C_15_H_26_O
23	22.12	Carotol	0.22	222	C_15_H_26_O
24	22.48	Cubenol	0.53	222	C_15_H_26_O
25	22.80	α-Cadinol	0.77	222	C_15_H_26_O
26	30.01	Palmitic acid vinyl ester	0.60	282	C_18_H_34_O_2_

* R.t, retention time (min).

**Table 3:** IC_50_ and selectivity index of *C. verum* extracts.

*C. verum* Extracts	Parasites	IC_50_ (µg/mL) ^a^		EC_50_ (µg/mL) ^b^			Selective Indices ^c^	
			MDBK	NIH/3T3	HFF	MDBK	NIH/3T3	HFF
AECV	*B. bovis*	23.1 ± 1.4	440 ± 10.6	816 ± 12.7	914 ± 12.2	19	35.3	39.6
	*B. bigemina*	56.6 ± 9.1	440 ± 10.6	816 ± 12.7	914 ± 12.2	7.8	14.4	16.1
	*B. divergens*	33.4 ± 2.1	440 ± 10.6	816 ± 12.7	914 ± 12.2	13.2	24.4	27.2
	*B. caballi*	40.3 ± 7.5	440 ± 10.6	816 ± 12.7	914 ± 12.2	10.9	20.2	22.7
	*T. equi*	18.8 ± 1.6	440 ± 10.6	816 ± 12.7	914 ± 12.2	23.4	43.4	48.6
EAECV	*B. bovis*	40.1 ± 8.5	376 ± 11.2	610 ± 7.7	790 ± 12.4	9.4	15.2	19.7
	*B. bigemina*	55.6 ± 1.1	376 ± 11.2	610 ± 7.7	790 ± 12.4	6.8	11	14.2
	*B. divergens*	45.7 ± 1.9	376 ± 11.2	610 ± 7.7	790 ± 12.4	8.2	13.3	17.3
	*B. caballi*	50.2 ± 6.2	376 ± 11.2	610 ± 7.7	790 ± 12.4	7.5	12.2	15.7
	*T. equi*	61.5 ± 5.2	376 ± 11.2	610 ± 7.7	790 ± 12.4	6.1	9.9	12.8

^a^ Half-maximal inhibitory concentration of extracts on piroplasm parasites in vitro. ^b^ Half-maximal effective concentration of extracts on cell lines. The values were determined from the dose-response curve using non-linear regression (curve fit analysis). The values are the means of triplicate experiments. ^c^ Ratio of the cell lines EC_50_ to the parasite IC_50_. High numbers are favorable.

**Table 4:** The viability of parasites treated with *Cinnamomum verum* extracts.

*C. verum* Extracts	Concentrations	Parasites				
		*B. bovis*	*B. bigemina*	*B. divergens*	*B. caballi*	*T. equi*
AECV	0.25 × IC_50_	+	+	+	+	+
	0.5 × IC_50_	+	+	+	+	+
	1 × IC_50_	+	+	+	+	+
	2 × IC_50_	−	+	−	+	+
	4 × IC_50_	−	−	−	−	−
EAECV	0.25 × IC_50_	+	+	+	+	+
	0.5 × IC_50_	+	+	+	+	+
	1 × IC_50_	+	+	+	+	+
	2 × IC_50_	−	−	−	−	−
	4 × IC_50_	−	−	−	−	−

Results are expressed as the mean values from three separate trials ± SD, a positive (+) indicates parasites regrowth, and a negative (−) indicates total parasites clearance after withdrawing the drug pressure using microscopy assay.

**Table 5:** Combination effect of DA and *C. verum* extracts.

Drug	*C. verum* Extracts ^a^	Parasites	CI Values (µg/ mL)				Weighted Average CI Values ^b^	Degree of Association ^c^
			IC_50_	IC_75_	IC_90_	IC_95_		
DA	AECV	*B. bovis*	0.8390	0.9030	1.110	0.9778	0.9890	Additive
	AECV	*B. bigemina*	1.3120	0.6710	0.9180	0.8080	0.8640	Synergism
	AECV	*B. divergens*	1.8250	0.9289	0.8772	0.8941	0.9891	Additive
	AECV	*B. caballi*	0.9950	0.9941	0.7791	0.8942	0.8898	Synergism
	AECV	*T. equi*	1.4860	1.0032	0.5638	0.6765	0.7890	Synergism
DA	EAECV	*B. bovis*	2.4480	0.6991	0.8968	0.8008	0.9740	Additive
	EAECV	*B. bigemina*	1.8330	0.6234	0.6771	0.6946	0.7890	Synergism
	EAECV	*B. divergens*	1.9150	0.7678	0.7834	0.7723	0.8890	Synergism
	EAECV	*B. caballi*	1.3350	0.7968	0.4687	0.5763	0.6640	Synergism
	EAECV	*T. equi*	3.1812	0.4276	0.7876	0.8977	0.9990	Additive

^a^*Cinnamomum verum* extracts —DA combination at a five selected concentrations of 0.25 × IC_50_, 0.5 × IC_50_, IC_50_, 2 × IC_50_, and 4 × IC_50_. ^b^ The average weighed CI value was calculated with the formula [(1 × IC_50_) + (2 × IC_75_) + (3 × IC_90_) + (4 × IC_95_)]/10. ^c^ The degree of synergism was determined based on the following CI value: <0.90 (synergism), 0.90–1.10 (additive), and >1.10 (antagonism). CI value, combination index value.

**Table 6:** Combination effect of methanolic *S*. *aromaticum* and *C. verum* extracts.

Herbal Extract	*C. verum* Extracts ^a^	Parasites	CI Values (µg/ mL)				Weighted Average CI Values ^b^	Degree of Association ^c^
			IC_50_	IC_75_	IC_90_	IC_95_		
MESA	AECV	*B. bovis*	0.3934	1.6034	0.8870	0.9072	0.9890	Additive
	AECV	*B. bigemina*	1.1050	1.6691	0.9668	0.9908	1.0340	Additive
	AECV	*B. divergens*	0.0027	1.1924	0.9787	0.8947	0.8897	Synergism
	AECV	*B. caballi*	0.9003	0.3189	0.2451	0.1279	0.2785	Synergism
	AECV	*T. equi*	3.1770	1.1043	0.8792	0.9667	1.1890	Additive
MESA	EAECV	*B. bovis*	1.9959	1.2378	0.9931	0.9398	1.1210	Additive
	EAECV	*B. bigemina*	0.4911	1.3267	0.7699	0.7912	0.8619	Synergism
	EAECV	*B. divergens*	1.7883	0.9831	0.8819	0.8917	0.9967	Additive
	EAECV	*B. caballi*	0.8594	1.4123	0.8192	0.9936	1.0121	Additive
	EAECV	*T. equi*	1.6923	1.3279	0.9739	0.8453	1.0651	Additive

^a^*Cinnamomum verum* extracts— methanolic *S. aromaticum* extract combination at a five selected concentrations of 0.25 × IC_50_, 0.5 × IC_50_, IC_50_, 2 × IC_50_, and 4 × IC_50_. ^b^ The average weighed CI value was calculated with the formula [(1 × IC_50_) + (2 × IC_75_) + (3 × IC_90_) + (4 × IC_95_)]/10. ^c^ The degree of synergism was determined based on the following CI value: <0.90 (synergism), 0.90–1.10 (additive), and >1.10 (antagonism). CI value, combination index value.

**Table 7:** Combination effect of methanolic *O. europaea* and *C. verum* extracts.

Drug	*C. verum* Extracts ^a^	Parasites	CI Values (µg/ mL)				Weighted Average CI Values ^b^	Degree of Association ^c^
			IC_50_	IC_75_	IC_90_	IC_95_		
MEOE	AECV	*B. bovis*	2.019	0.9993	0.9956	0.9713	1.0890	Additive
	AECV	*B. bigemina*	1.970	0.6910	0.7688	0.7708	0.8742	Synergism
	AECV	*B. divergens*	2.4530	0.6124	0.6776	0.7948	0.8890	Synergism
	AECV	*B. caballi*	2.6570	0.4539	0.3421	0.3245	0.5890	Synergism
	AECV	*T. equi*	2.7060	0.5423	0.3649	0.4391	0.6642	Synergism
MEOE	EAECV	*B. bovis*	1.8820	1.1932	0.8793	0.9935	1.0880	Additive
	EAECV	*B. bigemina*	1.3950	0.9939	0.7928	0.8823	0.9290	Synergism
	EAECV	*B. divergens*	1.3960	0.6728	0.5692	0.5381	0.6602	Synergism
	EAECV	*B. caballi*	1.2170	0.9817	0.8923	0.7582	0.8890	Synergism
	EAECV	*T. equi*	1.6742	0.8924	0.7718	0.7529	0.8786	Synergism

^a^*Cinnamomum verum* extracts—methanolic *O. europaea* extract combination at a five selected concentrations of 0.25 × IC_50_, 0.5 × IC_50_, IC_50_, 2 × IC_50_, and 4 × IC_50_. ^b^ The average weighed CI value was calculated with the formula [(1 × IC_50_) + (2 × IC_75_) + (3 × IC_90_) + (4 × IC_95_)]/10. ^c^ The degree of synergism was determined based on the following CI value: <0.90 (synergism), 0.90–1.10 (additive), and >1.10 (antagonism). CI value, combination index value.

## Supplementary Materials

The following are available online. Table S1. IC_50_ and selectivity index of diminazene aceturate (DA), methanolic *S. aromaticum* (MESA) and methanolic *O. europaea* (MEOE). Figure S1. GC-MS report for compounds identified from AECV. Figure S2. GC-MS report for compounds identified from EAECV.

## Abbreviations

AECV: Acetonic extract of *C. verum*; EAECV: Ethyl acetate extract of *C. verum*; MDBK: Madin-Darby bovine kidney; NIH/3T3: Mouse embryonic fibroblast; HFF: Human foreskin fibroblast; IC_50_: Half maximal inhibitory concentration; EC_50_: Half maximum effective concentrations; DMSO: Dimethyl sulfoxide; DA: Diminazene aceturate; MESA: Methanolic extract of *S. aromaticum*; MEOE: Methanolic extract of *O. europaea*; EDTA: Ethylenediaminetetraacetic acid; RBCs: Red blood cells; M199: Medium 199; iRBCs: infected red blood cells; MEM: Minimum Essential Medium Eagle; DMEM: Dulbecco’s Modified Eagle’s Medium; CCK-8: Cell Counting Kit-8; CI: Combination index; DDW: Double distilled water; HGB: Hemoglobin; HCT: Hematocrit.
