# Supplementary material for: Phytochemical Characterization and Chemotherapeutic Potential of Cinnamomum verum Extracts on the Multiplication of Protozoan Parasites In Vitro and In Vivo
Source: Molecules. 2020 Feb 24;25(4):996. doi: 10.3390/molecules25040996 (PMC7070835; doi:10.3390/molecules25040996)

My GC-MS Report

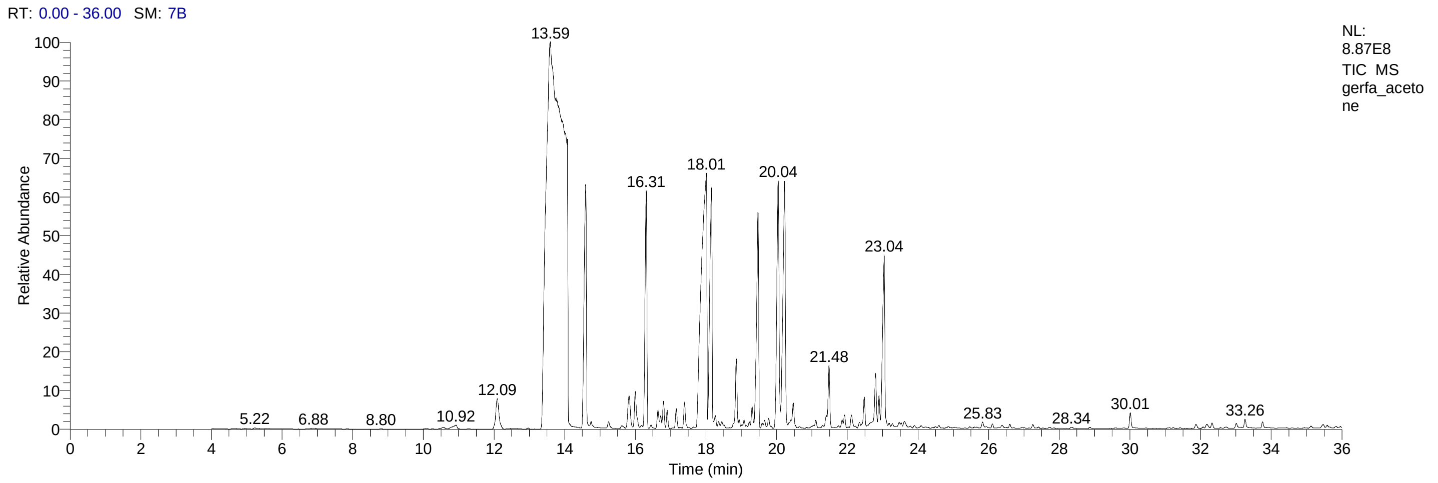

RT	Area %	Peak Area
12.09	0.82	426551844.35
13.59	52.87	27490372653.35
14.59	4.70	2444275758.69
15.82	0.69	358181589.01
16.00	0.62	323448015.47
16.31	3.72	1934080489.00
16.80	0.34	175677656.25
17.39	0.37	193108979.13
18.01	10.63	5524798093.56
18.15	4.35	2259611528.08
18.86	1.00	519143271.26
19.31	0.28	146352862.98
19.47	3.92	2037223466.72
20.04	4.64	2412482316.33
20.23	5.04	2621519142.89
20.47	0.44	227988039.01
21.48	1.05	546572802.75
22.48	0.41	215654397.46
22.80	0.67	347516631.91
22.90	0.28	147839625.80
23.04	3.15	1639644594.88

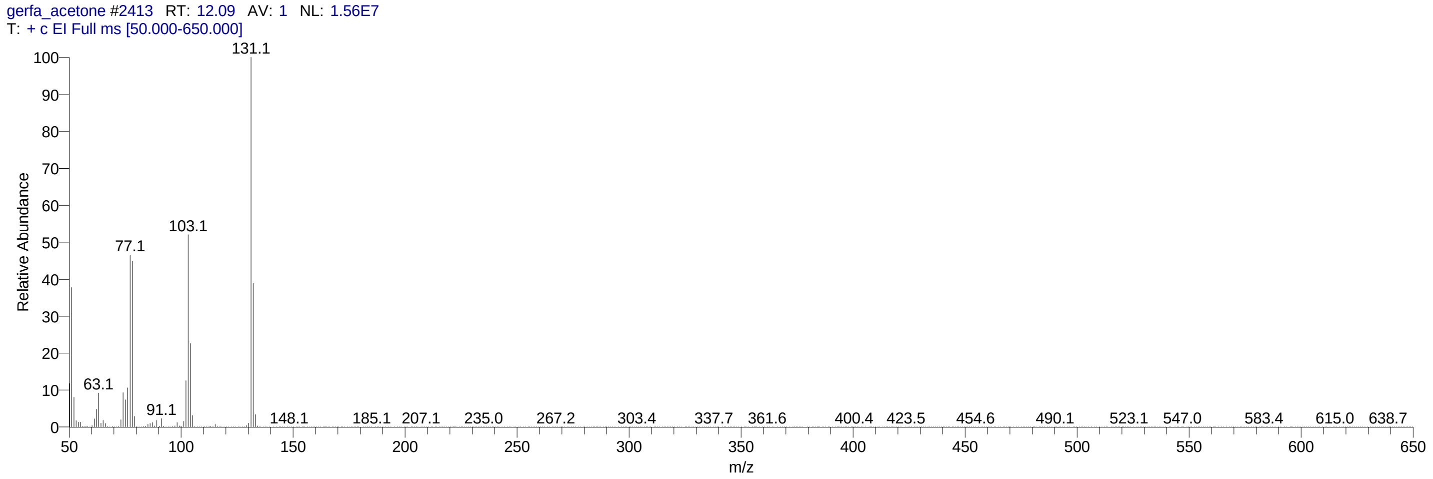

My GC-MS Report

RT	Compound Name	Area %	Molecular Formula	Molecular Weight	Cas #	MF	Library
12.09	(Z)-3-Phenylacrylaldehyde	0.82	C9H8O	132	57194-69-1	961	mainlib
12.09	Cinnamaldehyde, (E)-	0.82	C9H8O	132	14371-10-9	946	mainlib
12.09	2-Propenal, 3-phenyl-	0.82	C9H8O	132	104-55-2	945	replib

Compound Structure | Hit Spectrum

(Z)-3-Phenylacrylaldehyde
Formula C9H8O, MW 132, CAS# 57194-69-1, Entry# 115751
2-Propenal, 3-phenyl-, (2Z)-

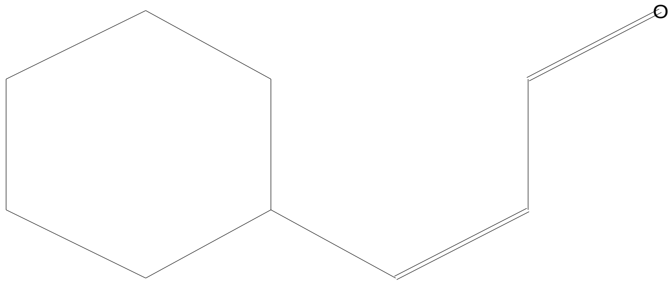

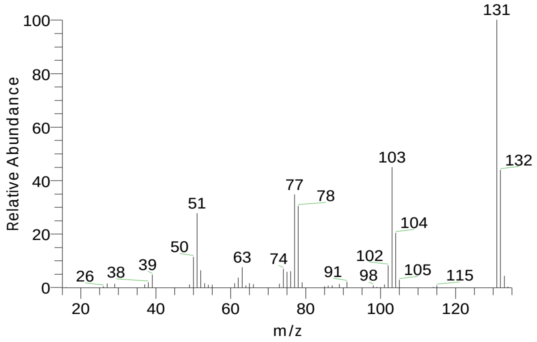

Cinnamaldehyde, (E)-
Formula C9H8O, MW 132, CAS# 14371-10-9, Entry# 115750
(E)-Cinnamaldehyde

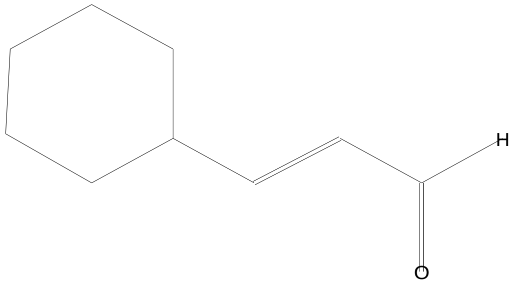

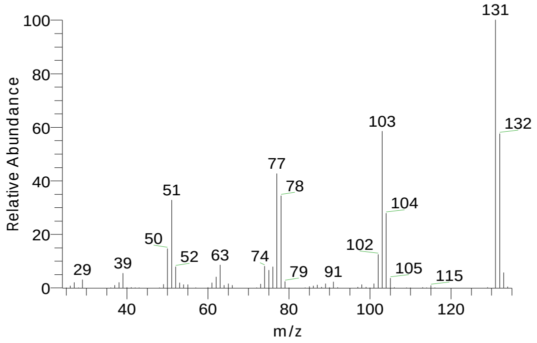

2-Propenal, 3-phenyl-
Formula C9H8O, MW 132, CAS# 104-55-2, Entry# 20950
Cinnamaldehyde

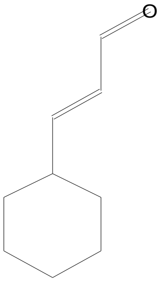

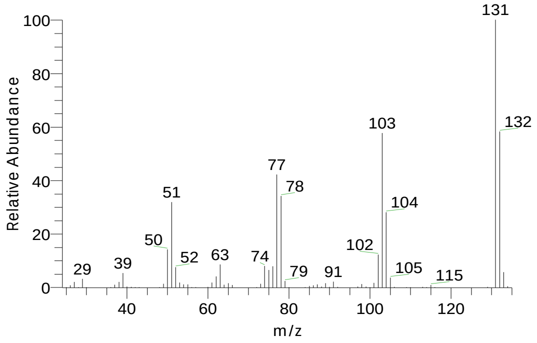

My GC-MS Report

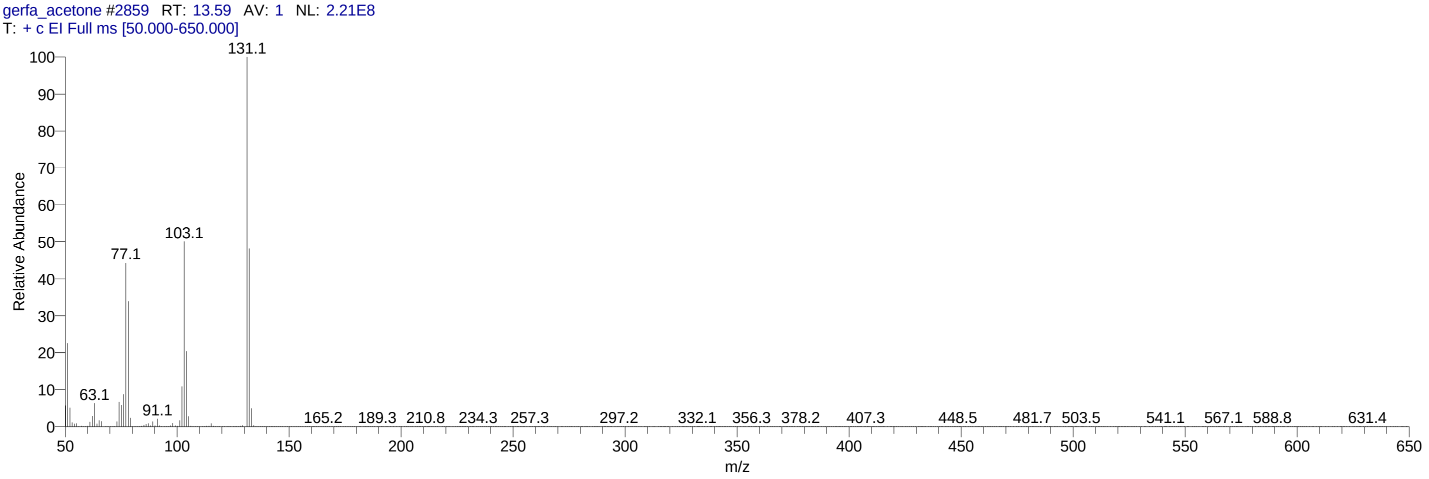

RT	Compound Name	Area %	Molecular Formula	Molecular Weight	Cas #	MF	Library
13.59	(Z)-3-Phenylacrylaldehyde	52.87	C9H8O	132	57194-69-1	960	mainlib
13.59	Cinnamaldehyde, (E)-	52.87	C9H8O	132	14371-10-9	947	mainlib
13.59	2-Propenal, 3-phenyl-	52.87	C9H8O	132	104-55-2	946	replib

Compound Structure	Hit Spectrum

(Z)-3-Phenylacrylaldehyde
Formula C9H8O, MW 132, CAS# 57194-69-1, Entry# 115751
2-Propenal, 3-phenyl-, (2Z)-

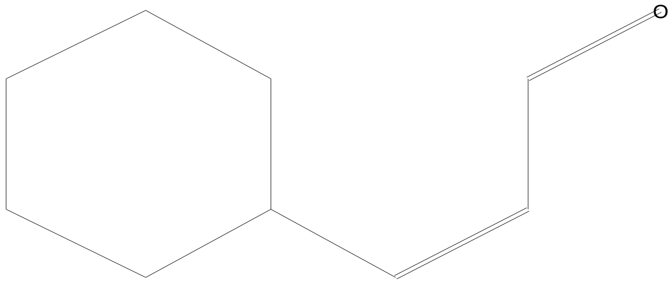

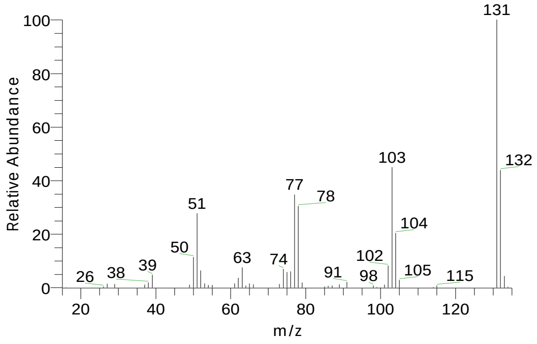

Cinnamaldehyde, (E)-
Formula C9H8O, MW 132, CAS# 14371-10-9, Entry# 115750
(E)-Cinnamaldehyde

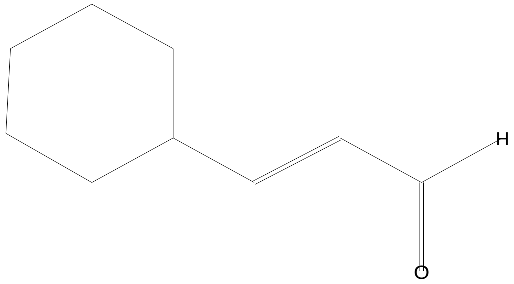

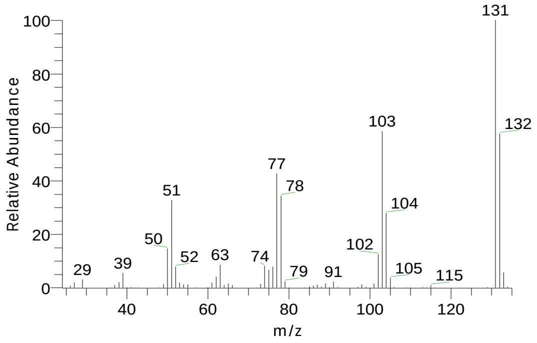

2-Propenal, 3-phenyl-
Formula C9H8O, MW 132, CAS# 104-55-2, Entry# 20950
Cinnamaldehyde

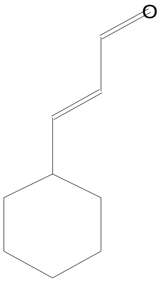

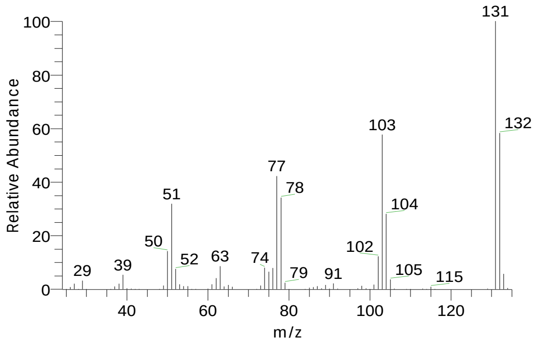

My GC-MS Report

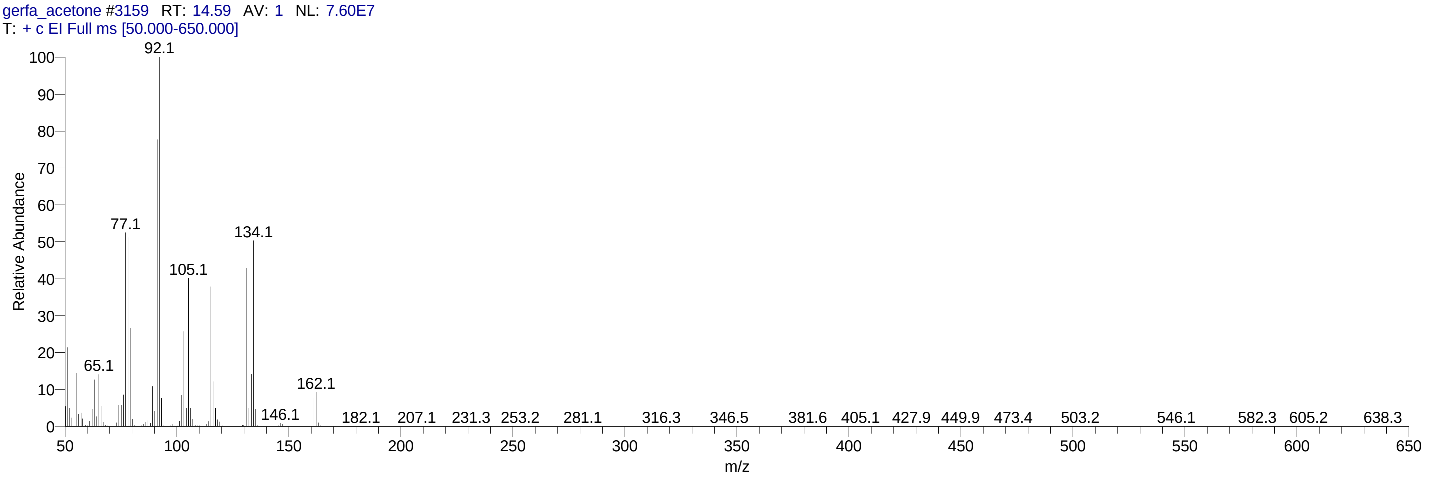

RT	Compound Name	Area %	Molecular Formula	Molecular Weight	Cas #	MF	Library
14.59	2-Propen-1-ol, 3-phenyl-, (E)-	4.70	C9H10O	134	4407-36-7	914	mainlib
14.59	2-Propen-1-ol, 3-phenyl-	4.70	C9H10O	134	104-54-1	903	replib
14.59	3-PHENYL-2-PROPEN-1-OL	4.70	C9H10O	134	NA	903	WileyRegistry8e

Compound Structure	Hit Spectrum

2-Propen-1-ol, 3-phenyl-, (E)-
Formula C9H10O, MW 134, CAS# 4407-36-7, Entry# 65164
$:28OOCCDEMITAIZTP-QPJJXVBHSA-N

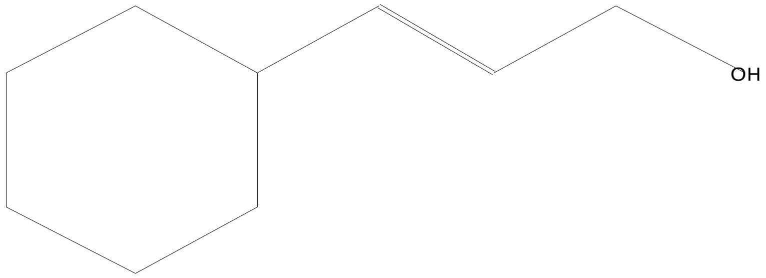

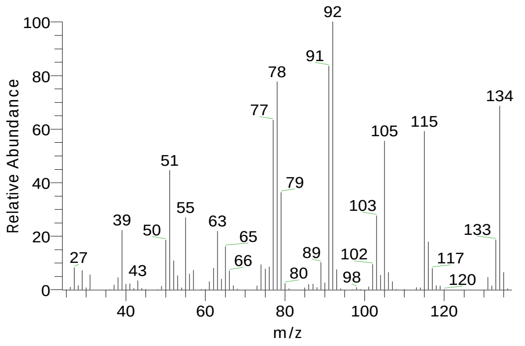

2-Propen-1-ol, 3-phenyl-
Formula C9H10O, MW 134, CAS# 104-54-1, Entry# 13932
Cinnamyl alcohol

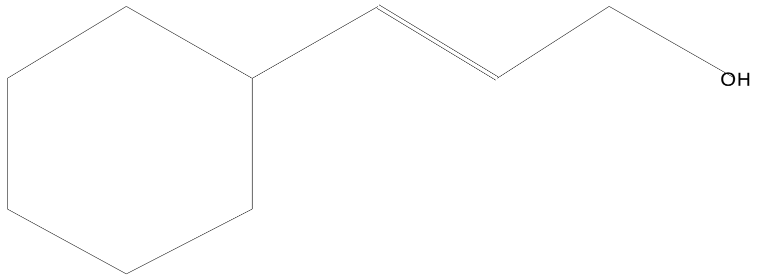

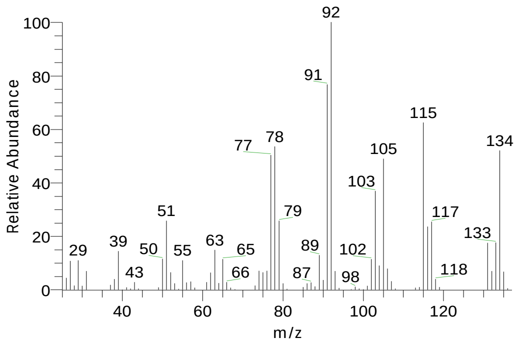

3-PHENYL-2-PROPEN-1-OL
Formula C9H10O, MW 134, CAS# NA, Entry# 384433
ZIMTALKOHOL

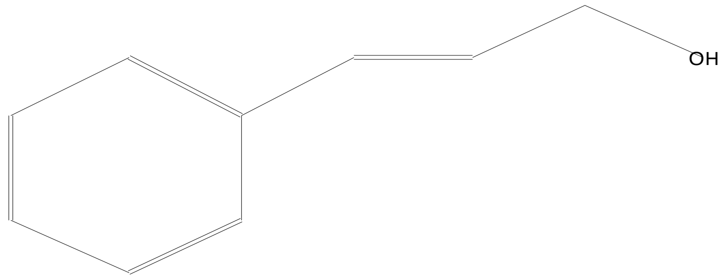

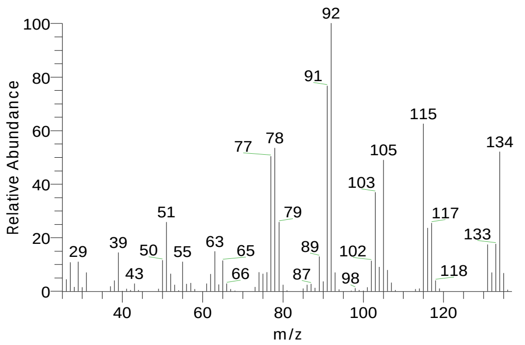

My GC-MS Report

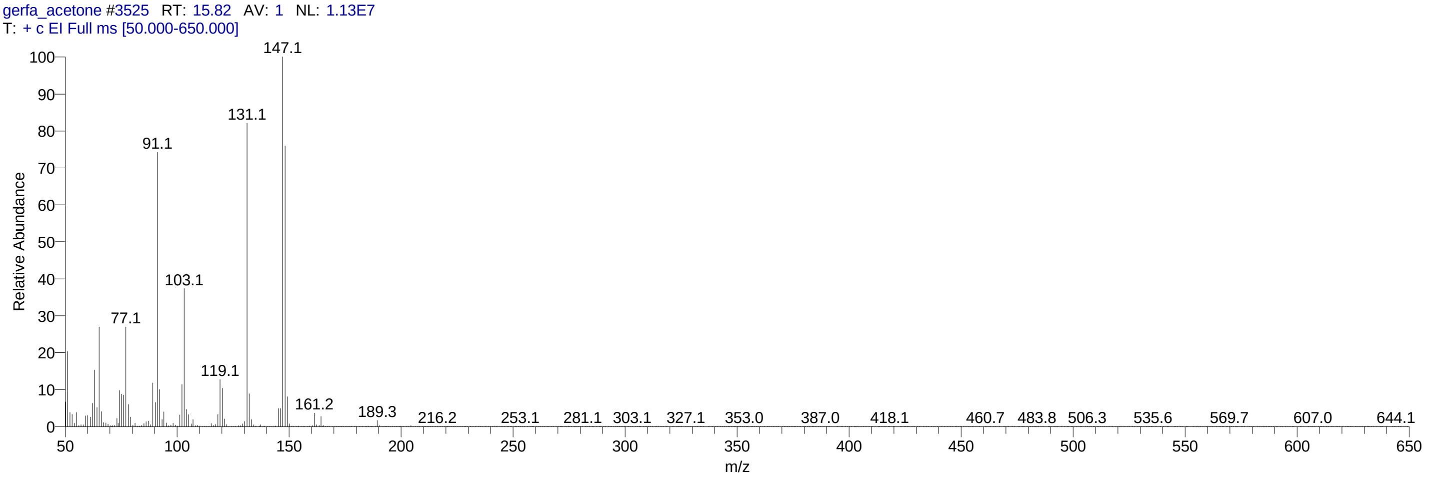

RT	Compound Name	Area %	Molecular Formula	Molecular Weight	Cas #	MF	Library
15.82	2-PROPENOIC ACID, 3-PHENYL-, (E)-	0.69	C9H8O2	148	140-10-3	850	WileyRegistry8e
15.82	2-PROPENOIC ACID, 3-PHENYL-	0.69	C9H8O2	148	621-82-9	850	WileyRegistry8e
15.82	2-Propenoic acid, 3-phenyl-	0.69	C9H8O2	148	621-82-9	855	replib

Compound Structure | Hit Spectrum

2-PROPENOIC ACID, 3-PHENYL-, (E)-
Formula C9H8O2, MW 148, CAS# 140-10-3, Entry# 32043
CINNAMIC ACID

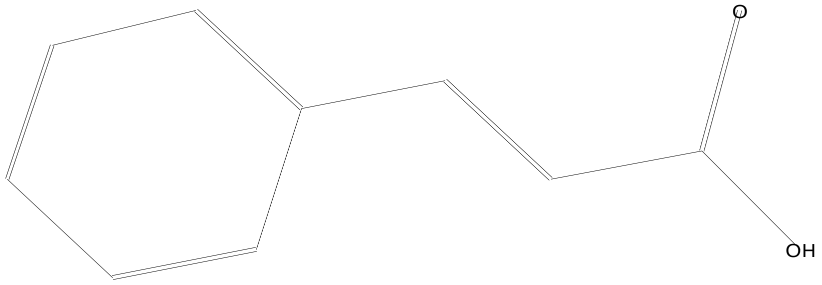

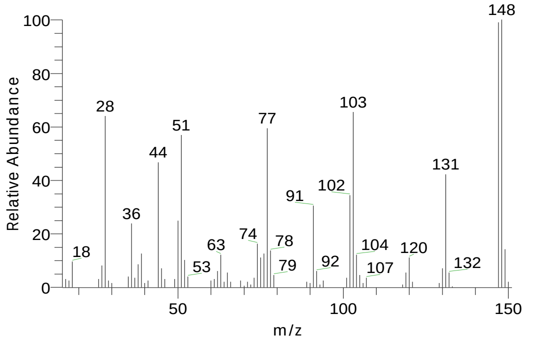

2-PROPENOIC ACID, 3-PHENYL-
Formula C9H8O2, MW 148, CAS# 621-82-9, Entry# 32046
CINNAMIC ACID

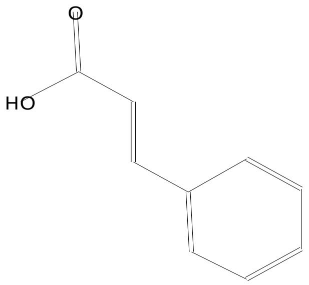

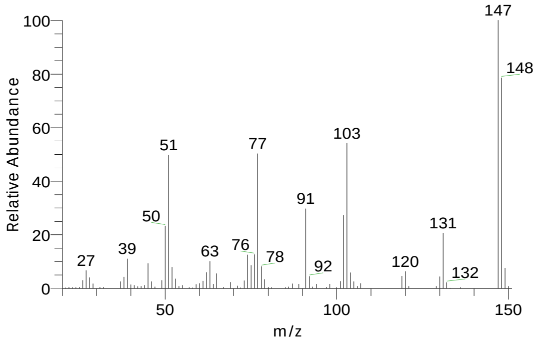

2-Propenoic acid, 3-phenyl-
Formula C9H8O2, MW 148, CAS# 621-82-9, Entry# 23248
Cinnamic acid

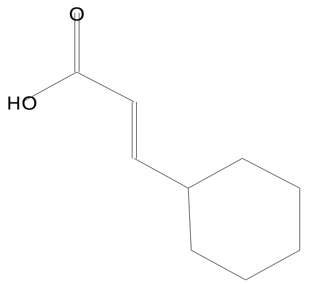

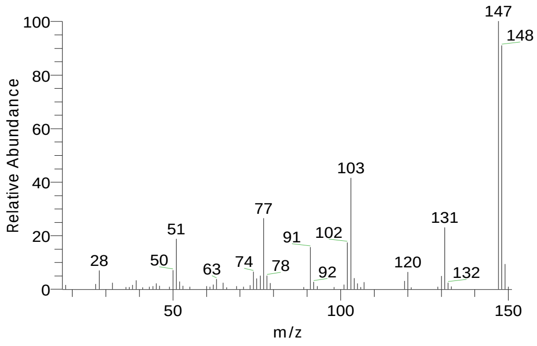

My GC-MS Report

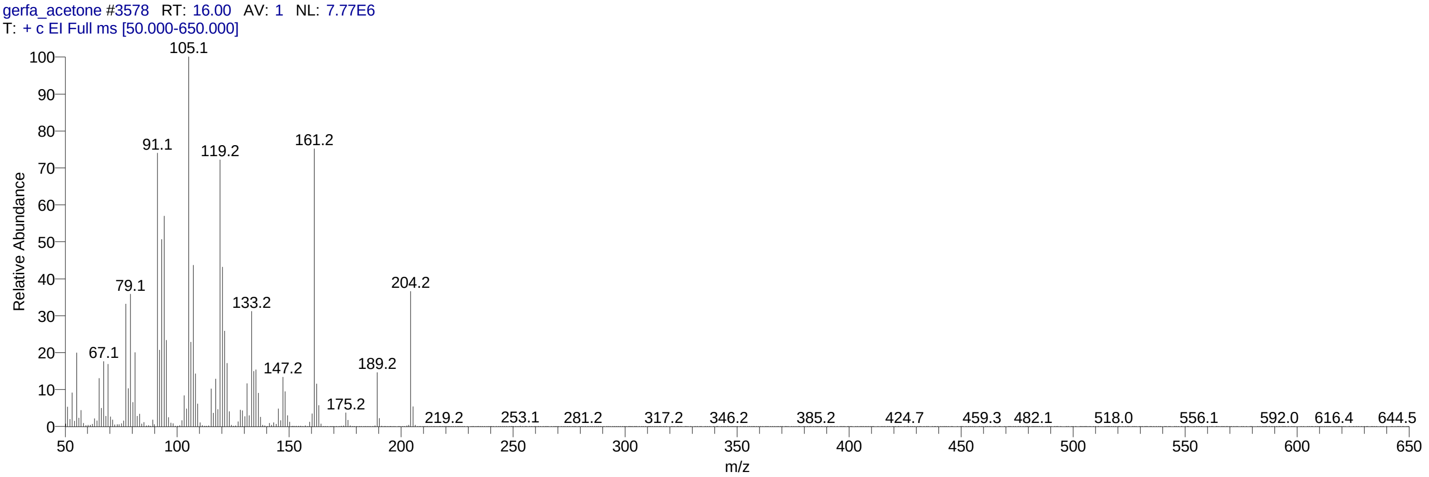

RT	Compound Name	Area %	Molecular Formula	Molecular Weight	Cas #	MF	Library
16.00	1,2,4-Metheno-1H-indene, octahydro-1,7a-dimethyl-5-(1-methylethyl)-, [1S-(1à,2à,3aá,4à,5à,7aá,8S*)]-	0.62	C15H24	204	22469-52-9	951	replib
16.00	(+)-CYCLOSATIVEN	0.62	C15H24	204	NA	928	WileyRegistry8e
16.00	1,2,4-Metheno-1H-indene, octahydro-1,7a-dimethyl-5-(1-methylethyl)-, [1S-(1à,2à,3aá,4à,5à,7aá,8S*)]-	0.62	C15H24	204	22469-52-9	928	mainlib

Compound Structure

Hit Spectrum

Formula C15H24, MW 204, CAS# 22469-52-9, Entry# 16426
(+)-Cyclosativene

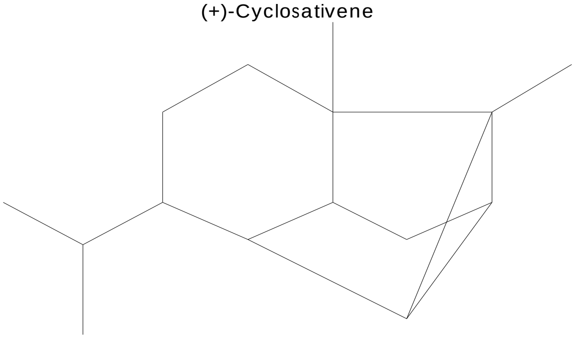

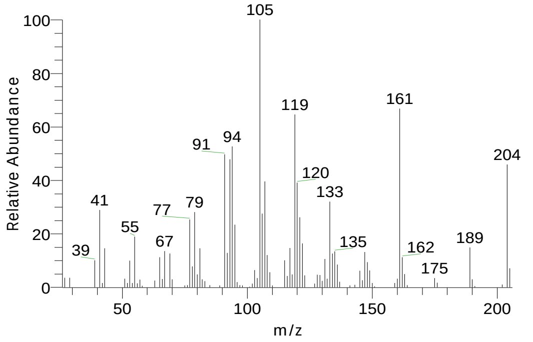

(+)-CYCLOSATIVEN
Formula C15H24, MW 204, CAS# NA, Entry# 385560

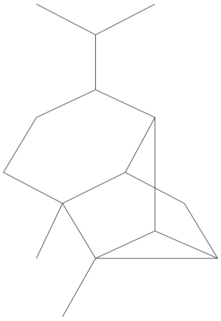

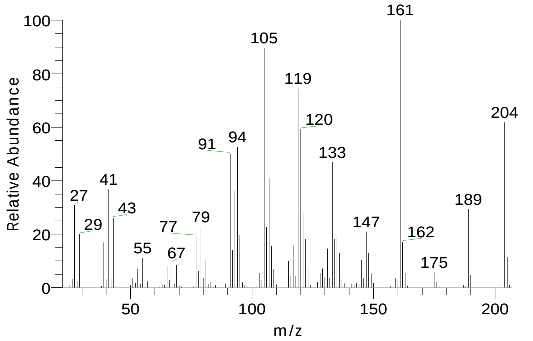

My GC-MS Report

Compound Structure | Hit Spectrum

Formula C15H24, MW 204, CAS# 22469-52-9, Entry# 150503
(+)-Cyclosativene

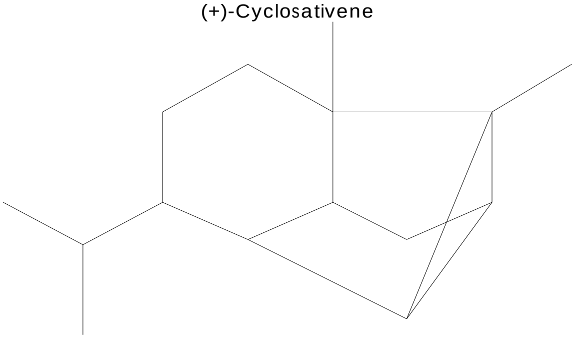

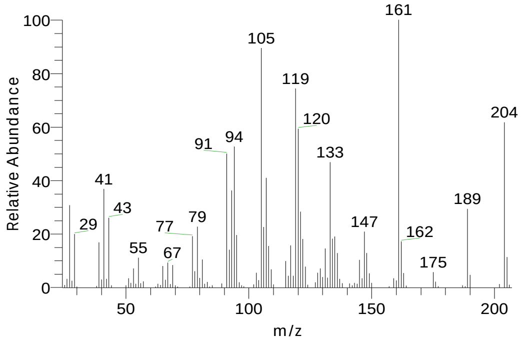

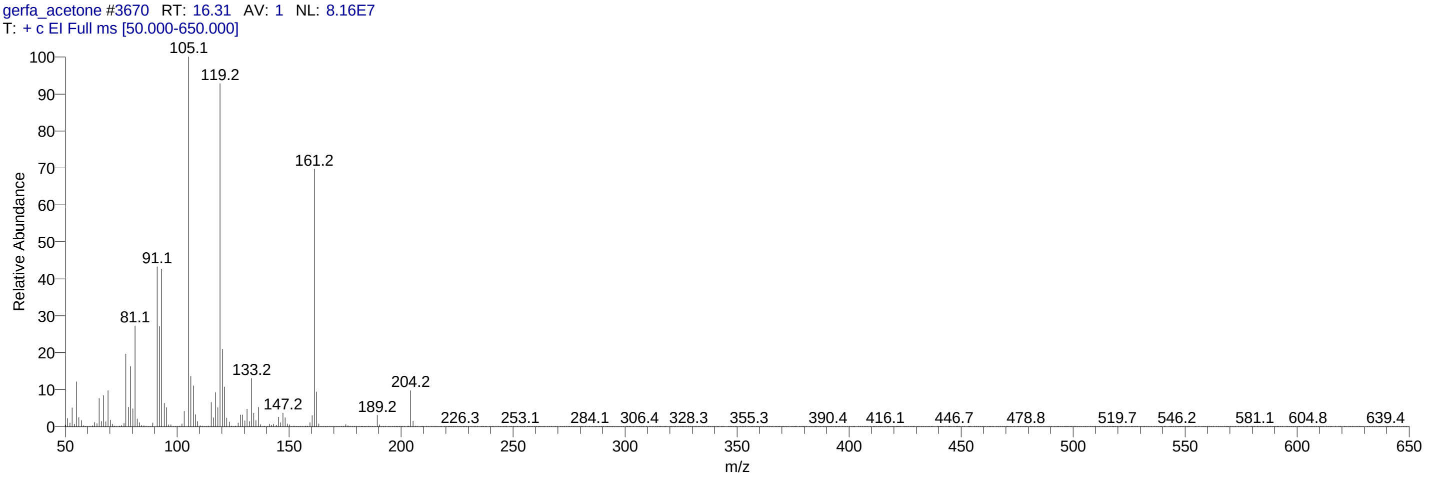

RT	Compound Name	Area %	Molecular Formula	Molecular Weight	Cas #	MF	Library
16.31	TRICYCLO[4.4.0.0(2,7)]DEC-3-ENE, 1,3-DIMETHYL-8-(1-METHYLETHYL)-, ST	3.72	C15H24	204	3856-25-5	951	WileyRegistry8e
16.31	.alfa.-Copaene	3.72	C15H24	204	NA	934	mainlib
16.31	TRICYCLO[4.4.0.0(2,7)]DEC-3-ENE, 1,3-DIMETHYL-8-(1-METHYLETHYL)-, ST	3.72	C15H24	204	3856-25-5	956	WileyRegistry8e

Compound Structure | Hit Spectrum

TRICYCLO[4.4.0.0(2,7)]DEC-3-ENE, 1,3-DIMETHYL-8-(1-METHYLETHYL)-, ST
Formula C15H24, MW 204, CAS# 3856-25-5, Entry# 89462
TRICYCLO[4.4.0.0(2,7)]DEC-3-ENE, 1,3-DIMETHYL-8-(1-METHYLETHYL)-, STEREOISOMER

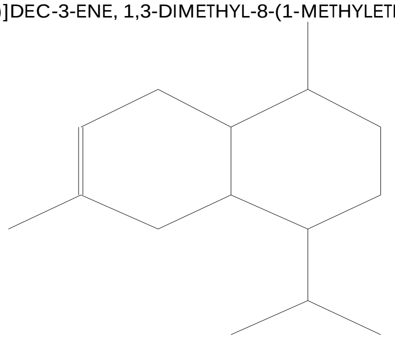

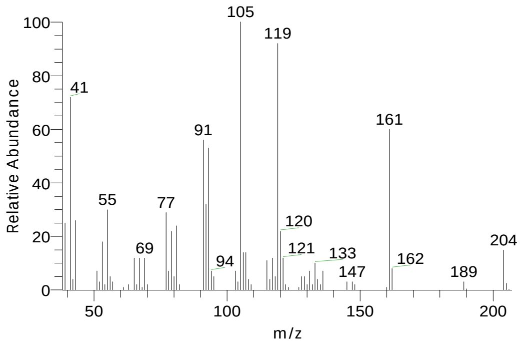

My GC-MS Report

Compound Structure | Hit Spectrum

.alfa.-Copaene
Formula C15H24, MW 204, CAS# NA, Entry# 150578
$:28VLXDPFLIRFYIME-UHFFFAOYSA-N

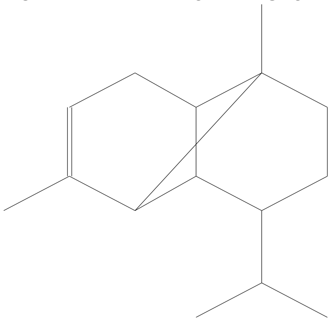

SI 933, RSI 934, mainlib, Entry# 150578, CAS# NA, .alfa.-Copaene

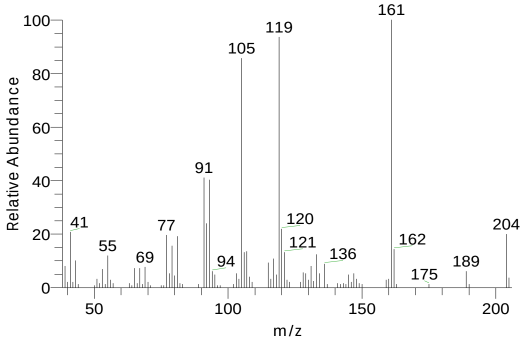

TRICYCLO[4.4.0.0(2,7)]DEC-3-ENE, 1,3-DIMETHYL-8-(1-METHYLETHYL)-, ST
Formula C15H24, MW 204, CAS# 3856-25-5, Entry# 89463
TRICYCLO[4.4.0.0(2,7)]DEC-3-ENE, 1,3-DIMETHYL-8-(1-METHYLETHYL)-, STEREOISOMER

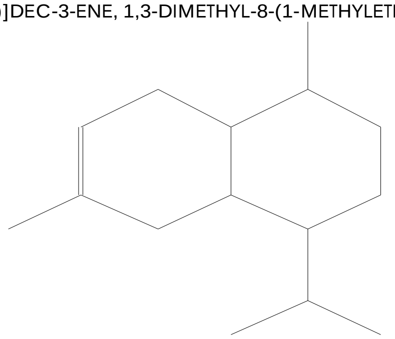

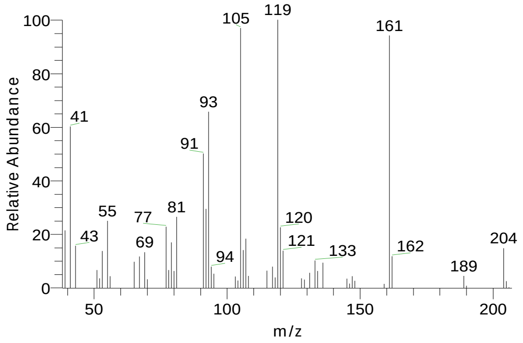

gerfa_acetone #3816 RT: 16.80 AV: 1 NL: 4.55E6
T: + c EI Full ms [50.000-650.000]

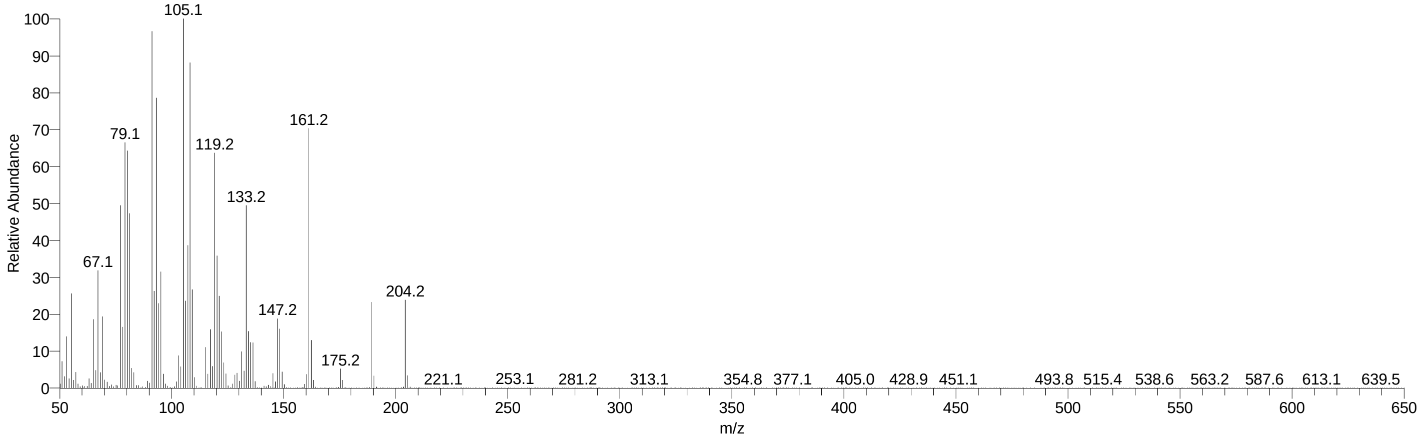

RT	Compound Name	Area %	Molecular Formula	Molecular Weight	Cas #	MF	Library
16.80	1,4-METHANO-1H-INDENE, OCTAHYDRO-4-METHYL-8-METHYLENE-7 -(1-METHYLETHYL)-, [1S-(1à,3Aá,4à,7à,7Aá)]-	0.34	C15H24	204	3650-28-0	894	WileyRegistry8e
16.80	1,4-Methano-1H-indene, octahydro-4-methyl-8-methylene-7-(1-methylethyl)-, [1S-(1à,3aá,4à,7à,7aá)]-	0.34	C15H24	204	3650-28-0	892	mainlib
16.80	1,4-METHANO-1H-INDENE, OCTAHYDRO-4-METHYL-8-METHYLENE-7 -(1-METHYLETHYL)-, [1S-(1à,3Aá,4à,7à,7Aá)]-	0.34	C15H24	204	3650-28-0	892	WileyRegistry8e

My GC-MS Report

Compound Structure | Hit Spectrum

Formula C15H24, MW 204, CAS# 3650-28-0, Entry# 89186
(+)-SATIVEN

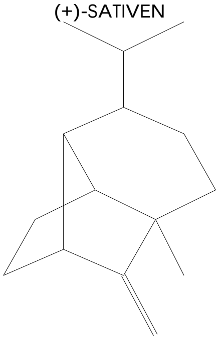

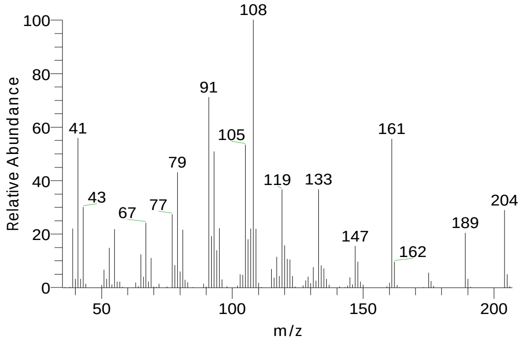

Formula C15H24, MW 204, CAS# 3650-28-0, Entry# 84625
(+)-Sativen

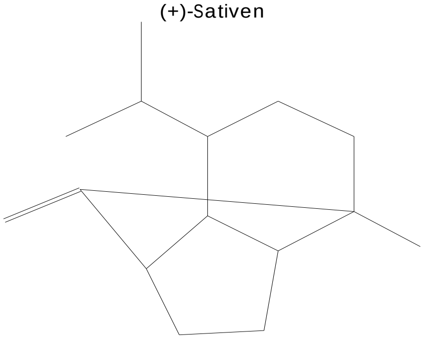

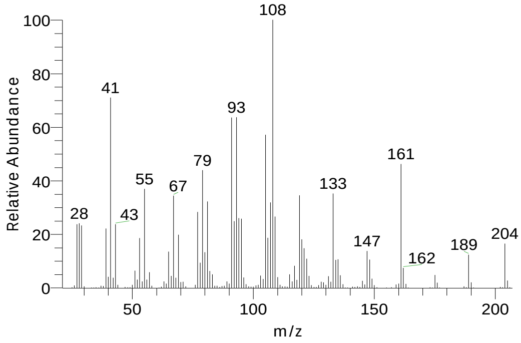

Formula C15H24, MW 204, CAS# 3650-28-0, Entry# 89185
(+)-SATIVEN

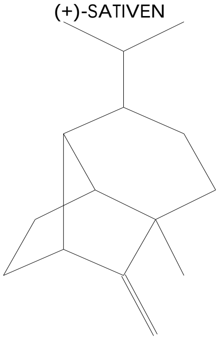

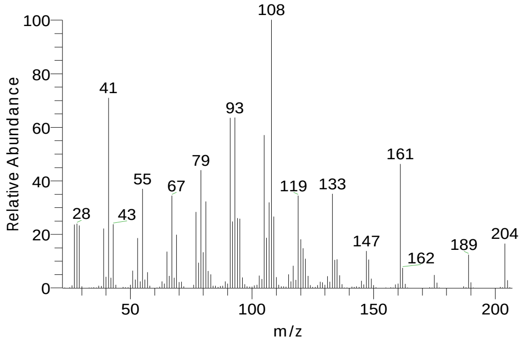

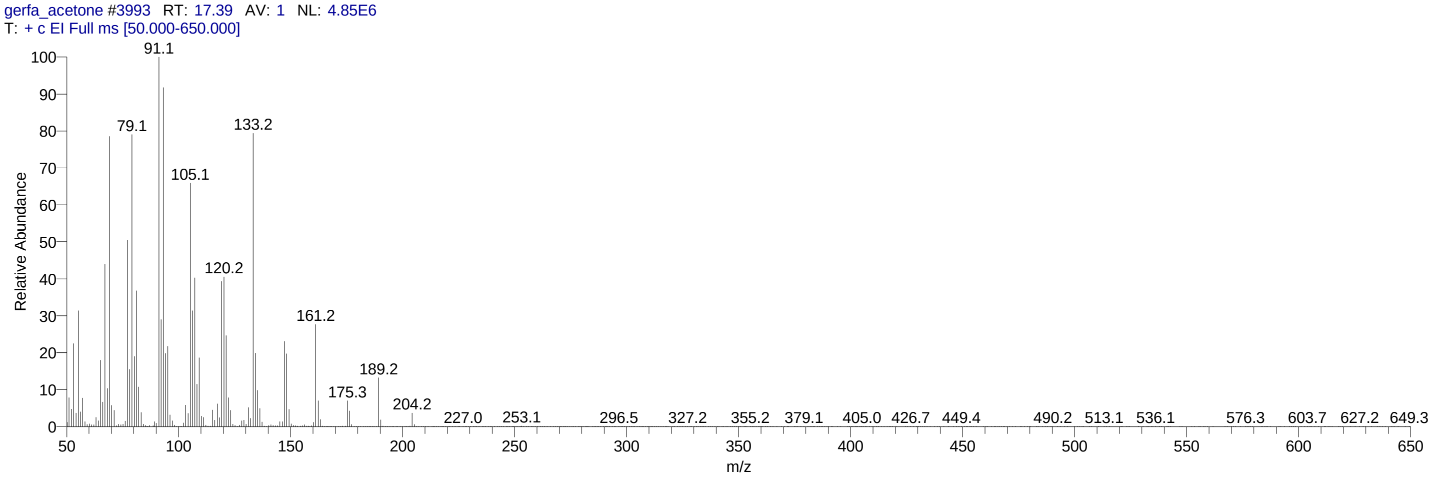

RT	Compound Name	Area %	Molecular Formula	Molecular Weight	Cas #	MF	Library
17.39	BICYCLO[7.2.0]UNDEC-4-ENE, 4,11,11-TRIMETHYL-8-METHYLENE-, [1R-(1R*,4E,9S*)]-	0.37	C15H24	204	87-44-5	950	WileyRegistry8e

My GC-MS Report

RT	Compound Name	Area %	Molecular Formula	Molecular Weight	Cas #	MF	Library
17.39	BICYCLO[7.2.0]UNDEC-4-ENE, 4,11,11-TRIMETHYL-8-METHYLENE-, [1R-(1R*,4E,9S*)]-	0.37	C15H24	204	87-44-5	951	WileyRegistry8e
17.39	BICYCLO[7.2.0]UNDEC-4-ENE, 4,11,11-TRIMETHYL-8-METHYLENE-, [1R-(1R*,4E,9S*)]-	0.37	C15H24	204	87-44-5	953	WileyRegistry8e

Compound Structure	Hit Spectrum

BICYCLO[7.2.0]UNDEC-4-ENE, 4,11,11-TRIMETHYL-8-METHYLENE-, [1R-(1R*,4E,9S*)]-
Formula C15H24, MW 204, CAS# 87-44-5, Entry# 89162
2,6,10,10-TETRAMETHYLBICYCLO[7.2.0]UNDECA-1,6-DIENE #

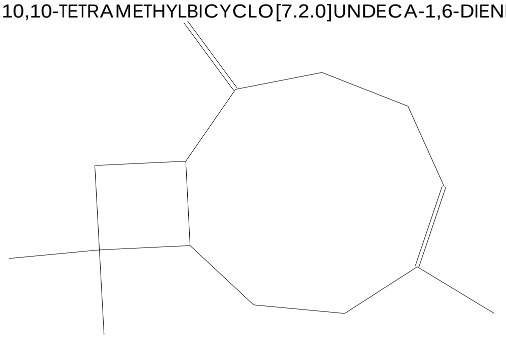

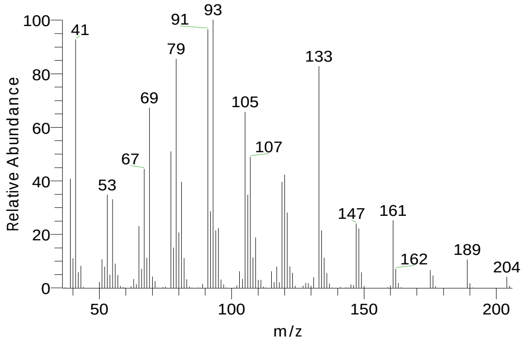

BICYCLO[7.2.0]UNDEC-4-ENE, 4,11,11-TRIMETHYL-8-METHYLENE-, [1R-(1R*,4E,9S*)]-
Formula C15H24, MW 204, CAS# 87-44-5, Entry# 89170
2,6,10,10-TETRAMETHYLBICYCLO[7.2.0]UNDECA-1,6-DIENE #

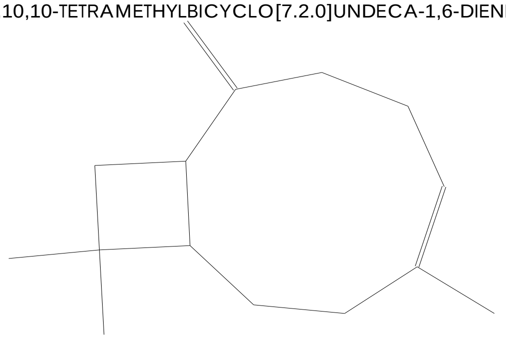

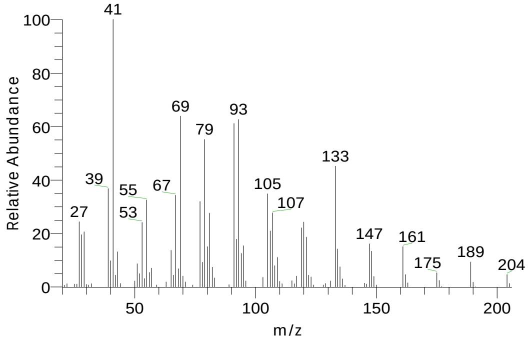

BICYCLO[7.2.0]UNDEC-4-ENE, 4,11,11-TRIMETHYL-8-METHYLENE-, [1R-(1R*,4E,9S*)]-
Formula C15H24, MW 204, CAS# 87-44-5, Entry# 89167
2,6,10,10-TETRAMETHYLBICYCLO[7.2.0]UNDECA-1,6-DIENE #

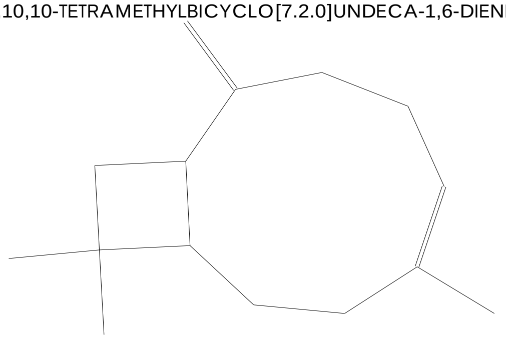

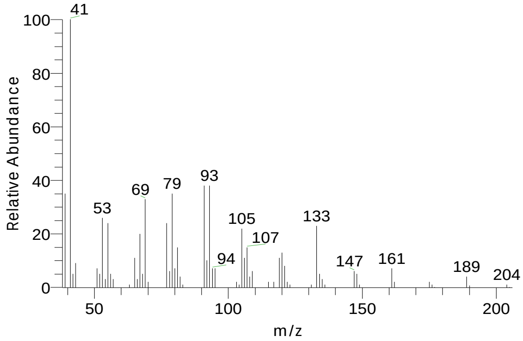

My GC-MS Report

gerfa_acetone #4176 RT: 18.01 AV: 1 NL: 1.48E8
T: + c EI Full ms [50.000-650.000]

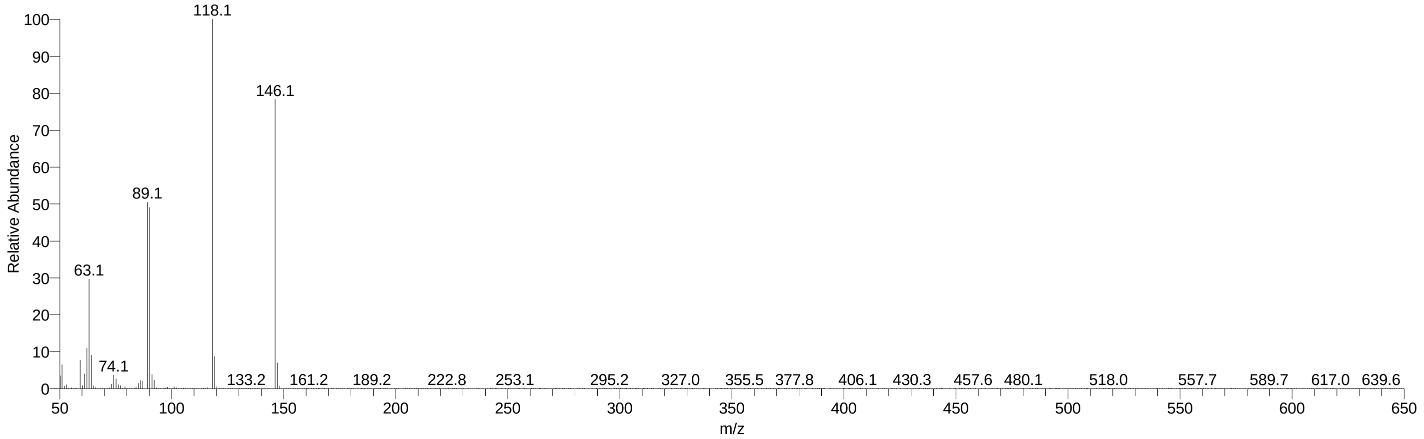

RT	Compound Name	Area %	Molecular Formula	Molecular Weight	Cas #	MF	Library
18.01	2H-1-BENZOPYRAN-2-ONE	10.63	C9H6O2	146	91-64-5	973	WileyRegistry8e
18.01	Coumarin	10.63	C9H6O2	146	91-64-5	960	replib
18.01	Coumarin	10.63	C9H6O2	146	91-64-5	943	replib

Compound Structure	Hit Spectrum

2H-1-BENZOPYRAN-2-ONE
Formula C9H6O2, MW 146, CAS# 91-64-5, Entry# 30896
CHROMEN-2-ONE

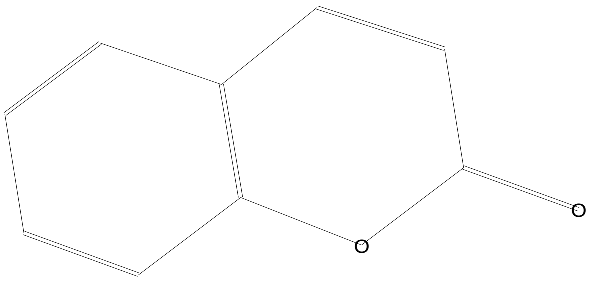

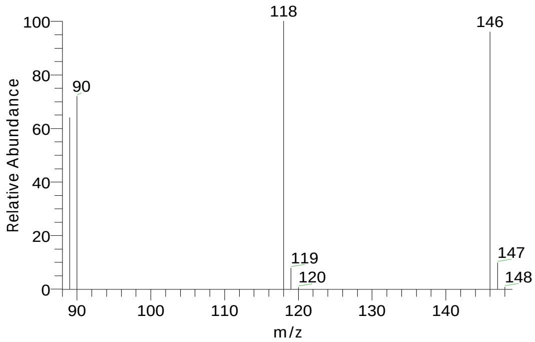

Coumarin
Formula C9H6O2, MW 146, CAS# 91-64-5, Entry# 18483
2H-1-Benzopyran-2-one

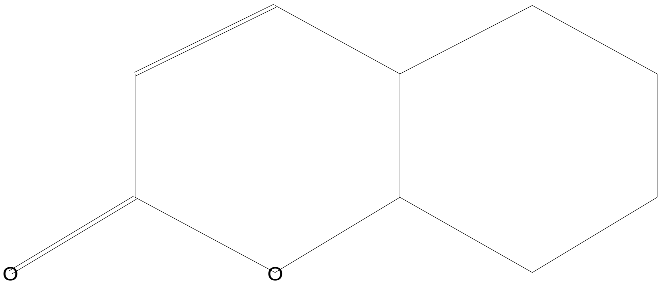

SI 948, RSI 960, replib, Entry# 18483, CAS# 91-64-5, Coumarin

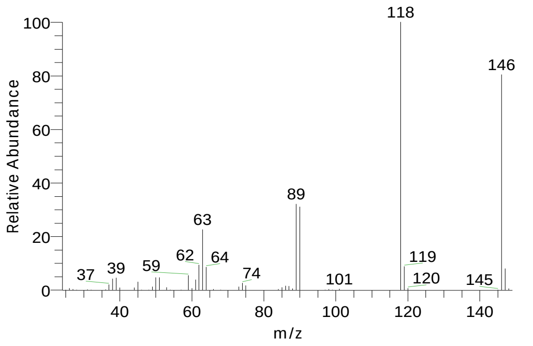

Coumarin
Formula C9H6O2, MW 146, CAS# 91-64-5, Entry# 18488
2H-1-Benzopyran-2-one

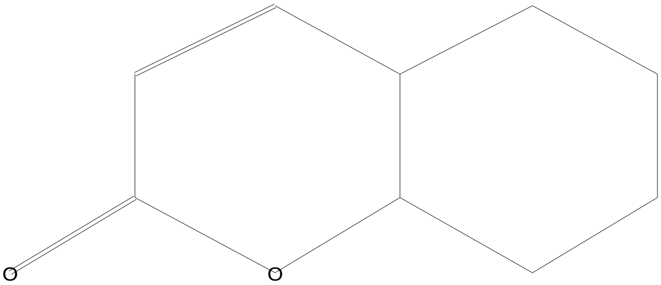

SI 943, RSI 943, replib, Entry# 18488, CAS# 91-64-5, Coumarin

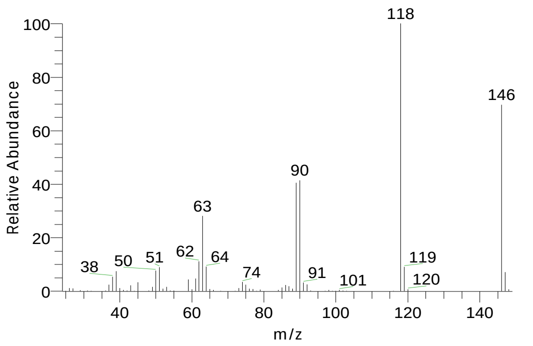

My GC-MS Report

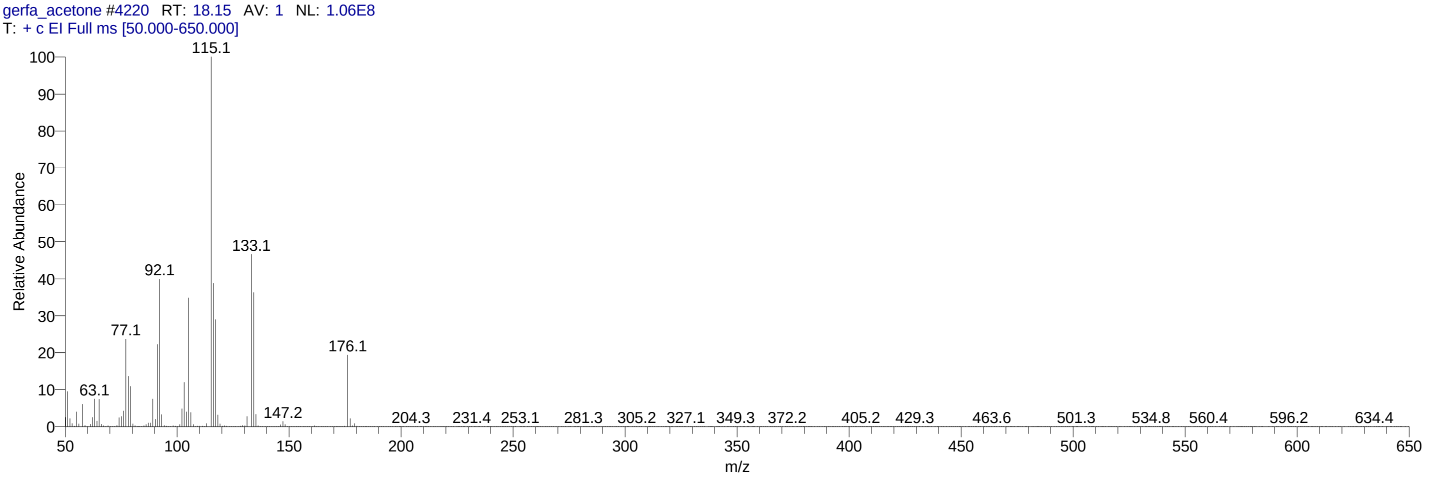

RT	Compound Name	Area %	Molecular Formula	Molecular Weight	Cas #	MF	Library
18.15	Acetic acid, cinnamyl ester	4.35	C11H12O2	176	103-54-8	946	mainlib
18.15	CINNAMYL ALCOHOL, ACETATE, (E)-	4.35	C11H12O2	176	21040-45-9	965	WileyRegistry8e
18.15	2-PROPEN-1-OL, 3-PHENYL-, ACETATE	4.35	C11H12O2	176	103-54-8	932	WileyRegistry8e

Compound Structure	Hit Spectrum

Acetic acid, cinnamyl ester
Formula C11H12O2, MW 176, CAS# 103-54-8, Entry# 11373
2-Propen-1-ol, 3-phenyl-, acetate

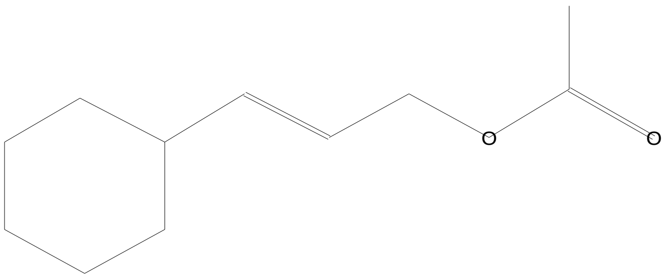

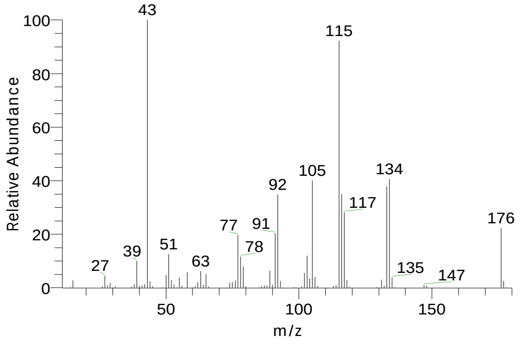

CINNAMYL ALCOHOL, ACETATE, (E)-
Formula C11H12O2, MW 176, CAS# 21040-45-9, Entry# 59025
(2E)-3-PHENYL-2-PROPENYL ACETATE #

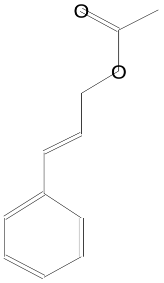

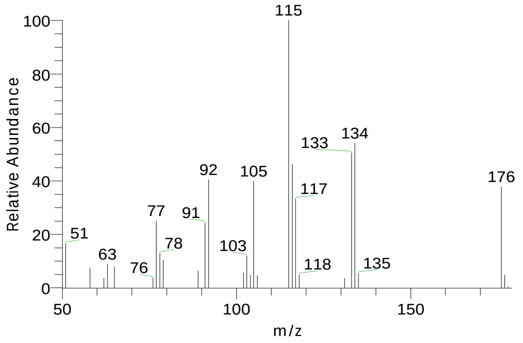

2-PROPEN-1-OL, 3-PHENYL-, ACETATE
Formula C11H12O2, MW 176, CAS# 103-54-8, Entry# 58949
ACETIC ACID CINNAMYL ESTER

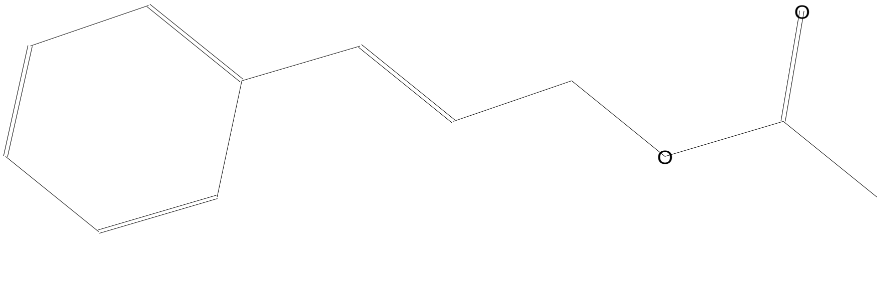

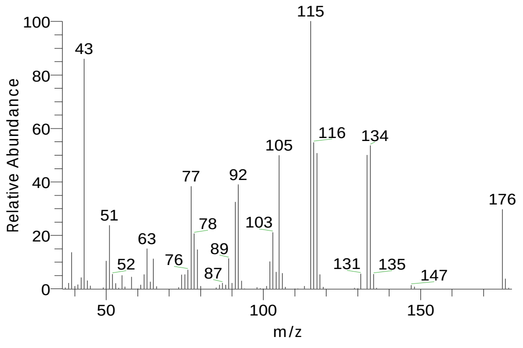

My GC-MS Report

gerfa_acetone #4430 RT: 18.86 AV: 1 NL: 1.74E7
T: + c EI Full ms [50.000-650.000]

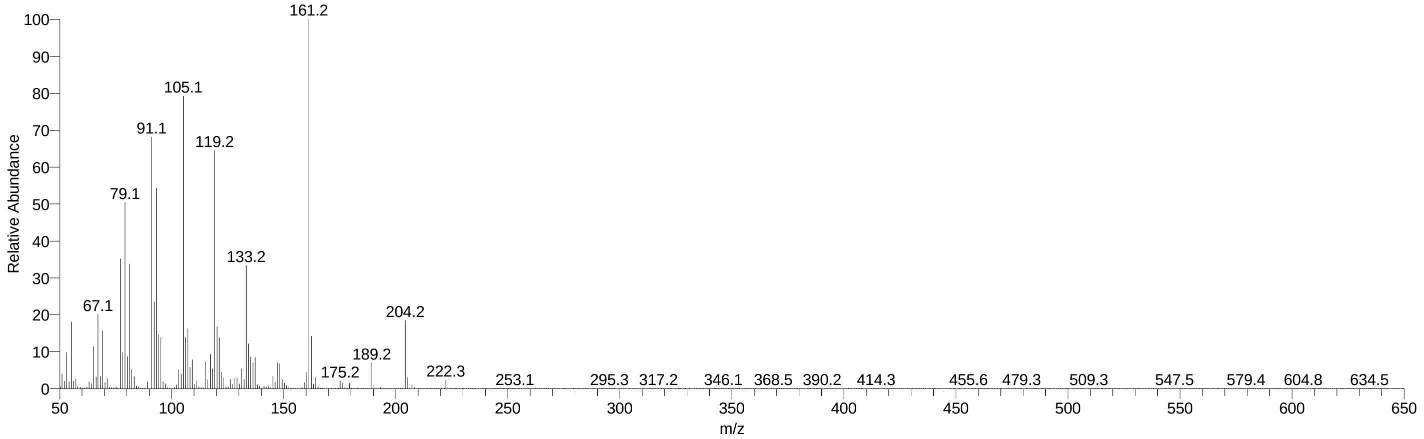

RT	Compound Name	Area %	Molecular Formula	Molecular Weight	Cas #	MF	Library
18.86	ç-Muurolene	1.00	C15H24	204	30021-74-0	933	replib
18.86	ç-Muurolene	1.00	C15H24	204	30021-74-0	933	replib
18.86	Naphthalene, 1,2,4a,5,6,8a-hexahydro-4,7-dimethyl-1-(1-methylethyl)-	1.00	C15H24	204	483-75-0	954	replib

Compound Structure | Hit Spectrum

ç-Muurolene
Formula C15H24, MW 204, CAS# 30021-74-0, Entry# 24906

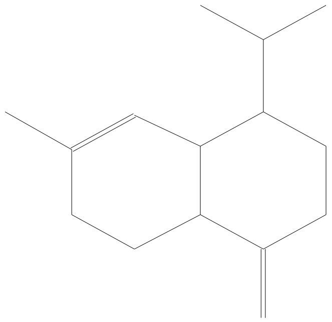

SI 916, RSI 933, replib, Entry# 24906, CAS# 30021-74-0, ç-Muurolene

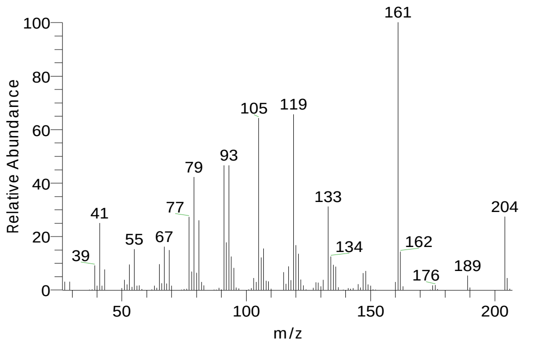

ç-Muurolene
Formula C15H24, MW 204, CAS# 30021-74-0, Entry# 24843

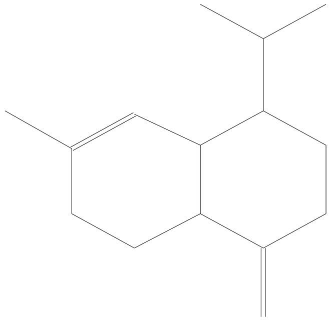

SI 915, RSI 933, replib, Entry# 24843, CAS# 30021-74-0, ç-Muurolene

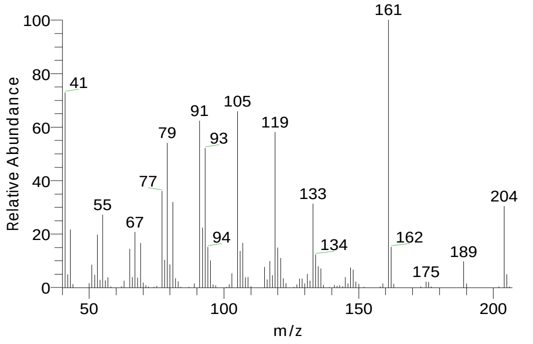

My GC-MS Report

Compound Structure | Hit Spectrum

Naphthalene, 1,2,4a,5,6,8a-hexahydro-4,7-dimethyl-1-(1-methylethyl)-
Formula C15H24, MW 204, CAS# 483-75-0, Entry# 24889
1-Isopropyl-4,7-dimethyl-1,2,4a,5,6,8a-hexahydronaphthalene #

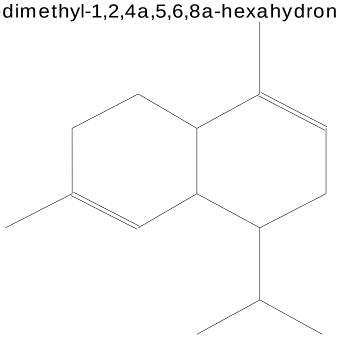

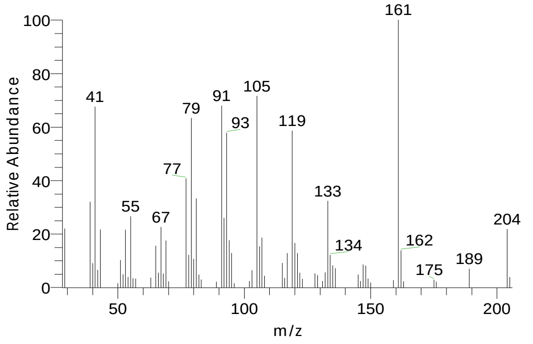

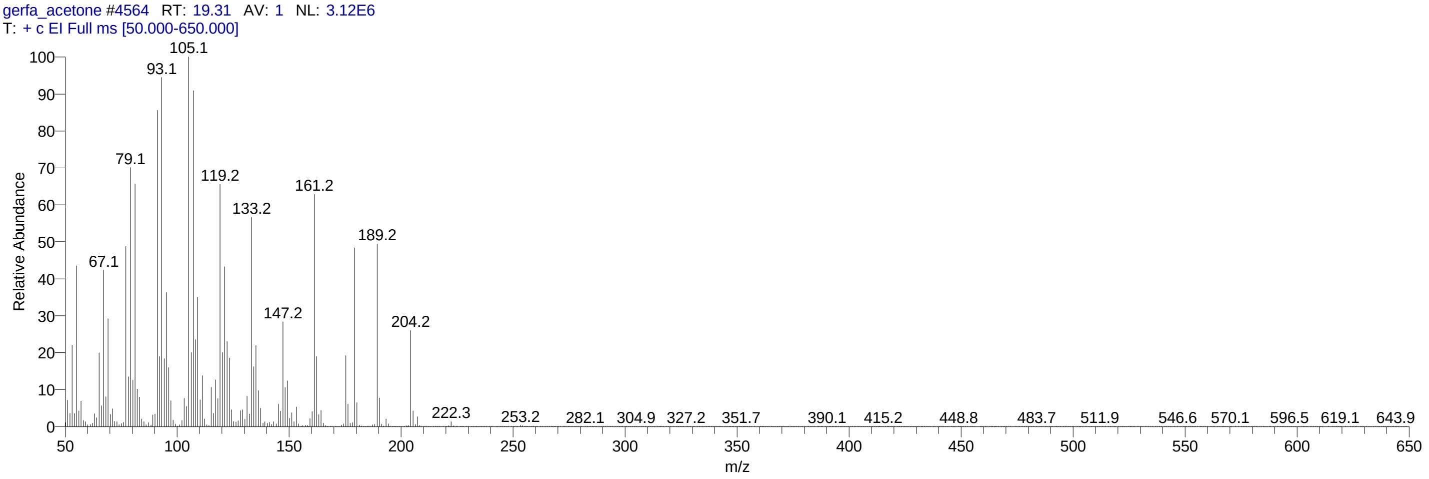

RT	Compound Name	Area %	Molecular Formula	Molecular Weight	Cas #	MF	Library
19.31	Azulene, 1,2,3,3a,4,5,6,7-octahydro-1,4-dimethyl-7-(1-methylethenyl)-, [1R-(1à,3aá,4à,7á)]-	0.28	C15H24	204	22567-17-5	918	replib
19.31	Aromandendrene	0.28	C15H24	204	489-39-4	905	replib
19.31	NAPHTHALENE, 1,2,3,5,6,7,8,8A-OCTAHYDRO-1,8A-DIMETHYL-7-(1-METHYLETHENYL)-, [1R-(1à,7á,8Aà)]-	0.28	C15H24	204	4630-07-3	891	WileyRegistry8e

Compound Structure | Hit Spectrum

Azulene, 1,2,3,3a,4,5,6,7-octahydro-1,4-dimethyl-7-(1-methylethenyl)-, [1R-(1à,3aá,4à,7á)]-
Formula C15H24, MW 204, CAS# 22567-17-5, Entry# 1321
1á,4áH,10áH-Guaia-5,11-diene

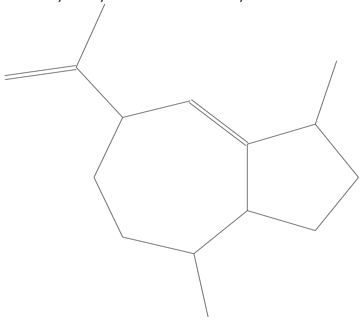

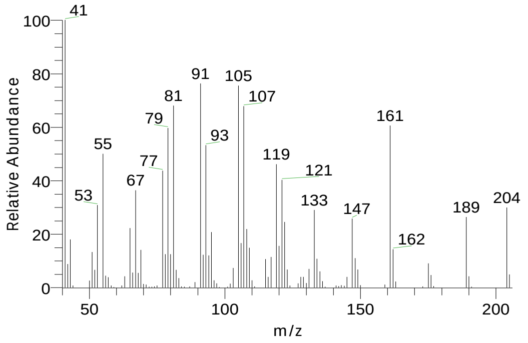

My GC-MS Report

Compound Structure | Hit Spectrum

Aromandendrene
Formula C15H24, MW 204, CAS# 489-39-4, Entry# 13310

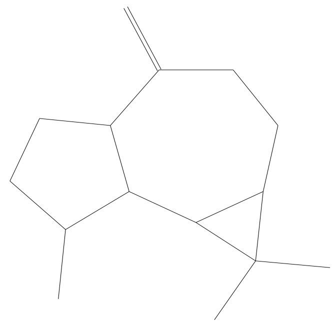

SI 857, RSI 905, replib, Entry# 13310, CAS# 489-39-4, Aromandendrene

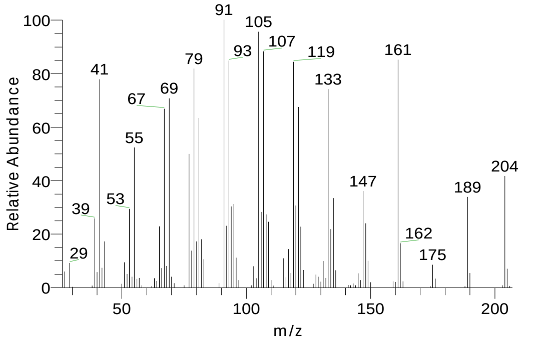

Formula C15H24, MW 204, CAS# 4630-07-3, Entry# 89318
3-ISOPROPENYL-4A,5-DIMETHYL-1,2,3,4,4A,5,6,7-OCTAHYDRONAPHTHALENE #

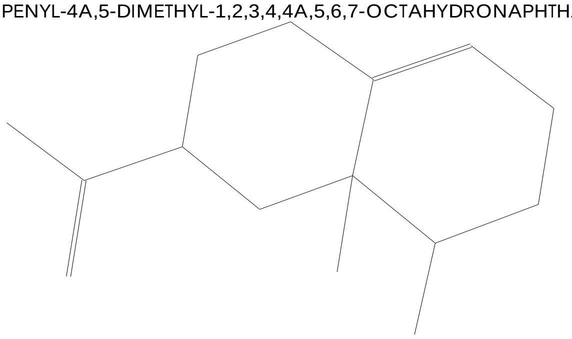

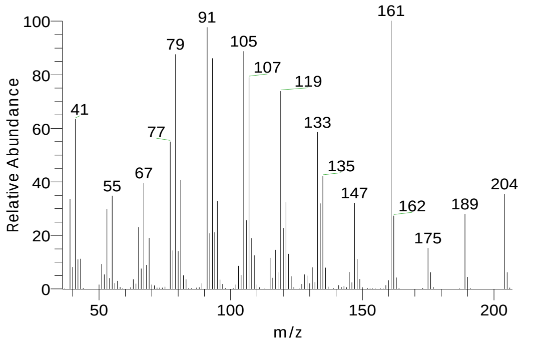

gerfa_acetone #4613 RT: 19.47 AV: 1 NL: 9.29E7
T: + c EI Full ms [50.000-650.000]

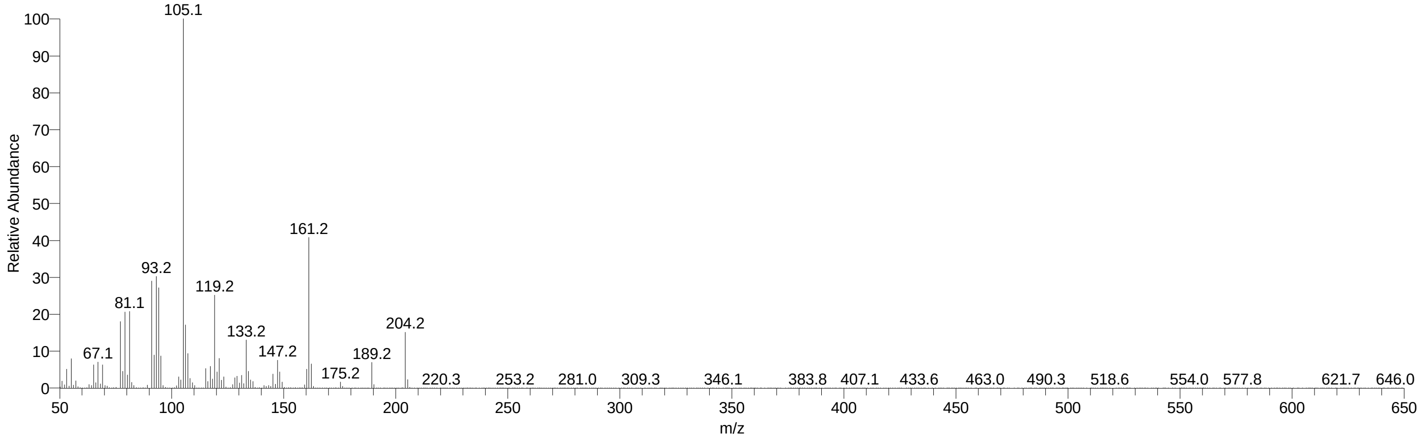

RT	Compound Name	Area %	Molecular Formula	Molecular Weight	Cas #	MF	Library
19.47	à-Muurolene	3.92	C15H24	204	10208-80-7	954	replib
19.47	à-Muurolene	3.92	C15H24	204	31983-22-9	959	replib
19.47	NAPHTHALENE, 1,2,4A,5,6,8A-HEXAHYDRO-4,7-DIMETHYL-1-(1-METHYLETHYL)-, (1à,4Aà,8Aà)-	3.92	C15H24	204	31983-22-9	930	WileyRegistry8e

My GC-MS Report

Compound Structure	Hit Spectrum

à-Muurolene
Formula C15H24, MW 204, CAS# 10208-80-7, Entry# 16423
Naphthalene, 1,2,4a,5,6,8a-hexahydro-4,7-dimethyl-1-(1-methylethyl)-, (1S,4aS,8aR)-

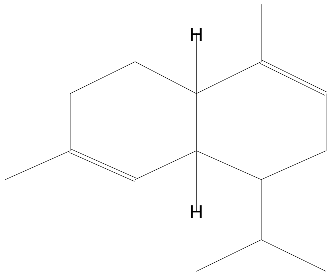

SI 954, RSI 954, replib, Entry# 16423, CAS# 10208-80-7, à-Muurolene

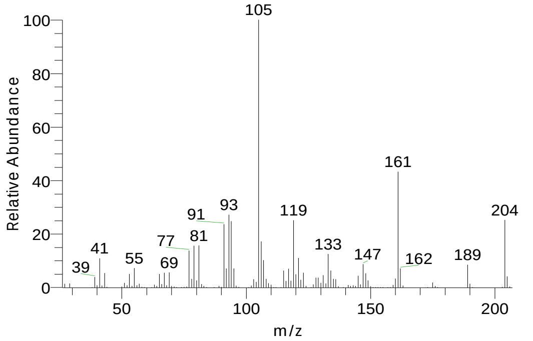

à-Muurolene
Formula C15H24, MW 204, CAS# 31983-22-9, Entry# 16421
[1à,4aà,8aà]-1,2,4a,5,6,8a-hexahydro-4-7-dimethyl-1-[1-methylethyl]naphthalene

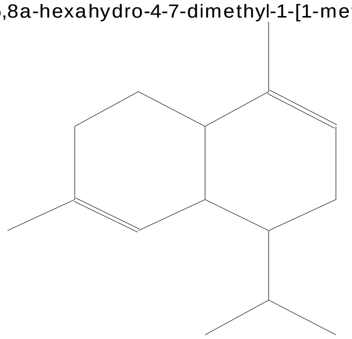

SI 953, RSI 959, replib, Entry# 16421, CAS# 31983-22-9, à-Muurolene

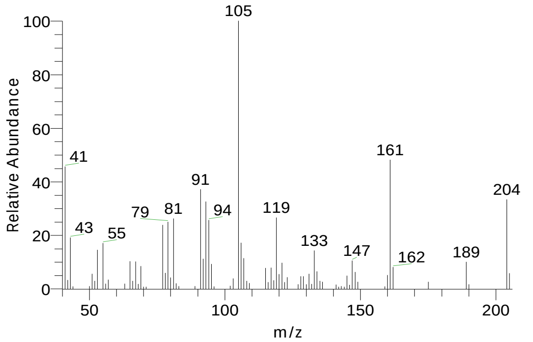

NAPHTHALENE, 1,2,4A,5,6,8A-HEXAHYDRO-4,7-DIMETHYL-1-(1-METHYLETHYL)-, (1à,4Aà,8Aà)-
Formula C15H24, MW 204, CAS# 31983-22-9, Entry# 89350
NAPHTHALENE, 1,2,4Aá,5,6,8Aá-HEXAHYDRO-1á-ISOPROPYL-4,7-DIMETHYL-

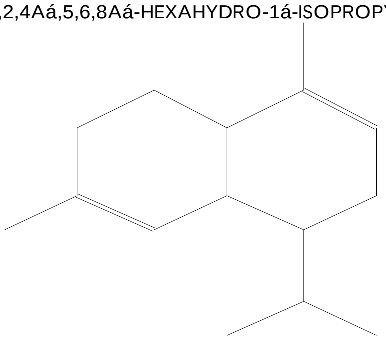

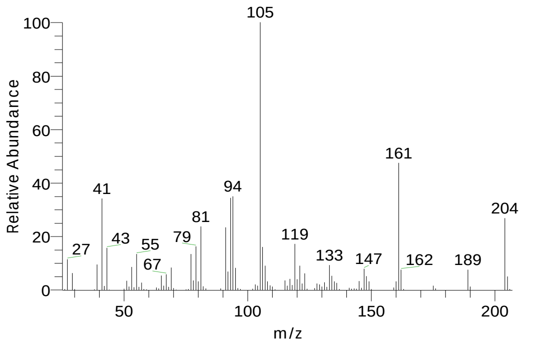

gerfa_acetone #4783 RT: 20.04 AV: 1 NL: 5.97E7
T: + c EI Full ms [50.000-650.000]

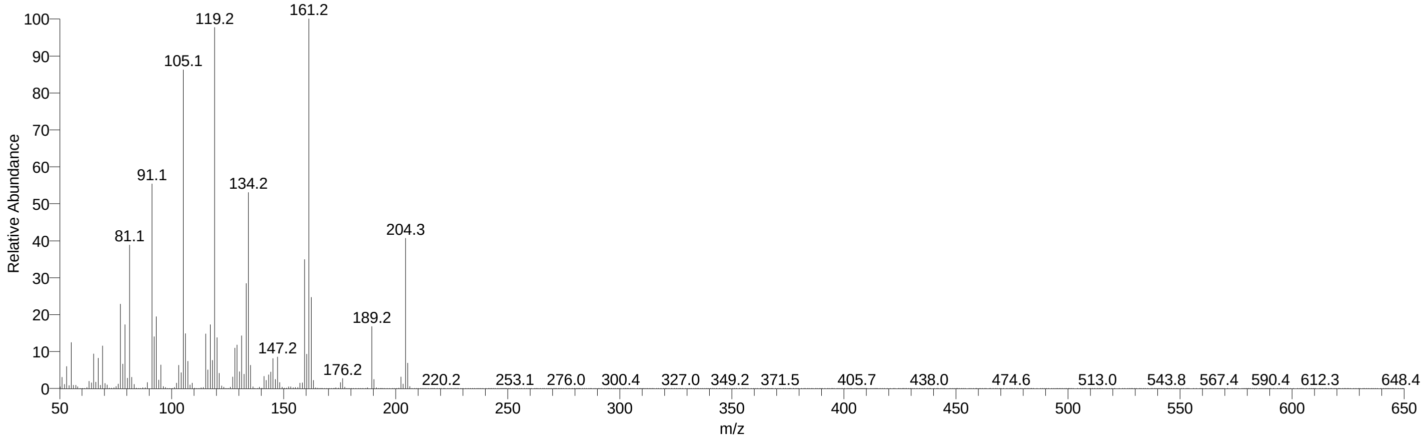

RT	Compound Name	Area %	Molecular Formula	Molecular Weight	Cas #	MF	Library
20.04	NAPHTHALENE, 1,2,3,5,6,8A-HEXAHYDRO-4,7-DIMETHYL-1-(1-METHYLETHYL)-, (1S-CIS)-	4.64	C15H24	204	483-76-1	937	WileyRegistry8e
20.04	1-Isopropyl-4,7-dimethyl-1,2,3,5,6,8a-hexahydronaphthalene	4.64	C15H24	204	16729-01-4	920	mainlib

My GC-MS Report

RT	Compound Name	Area %	Molecular Formula	Molecular Weight	Cas #	MF	Library
20.04	NAPHTHALENE, 1,2,3,5,6,8A-HEXAHYDRO-4,7-DIMETHYL-1-(1-METHYLETHYL)-, (1S-CIS)-	4.64	C15H24	204	483-76-1	895	WileyRegistry8e

Compound Structure | Hit Spectrum

NAPHTHALENE, 1,2,3,5,6,8A-HEXAHYDRO-4,7-DIMETHYL-1-(1-METHYLETHYL)-, (1S-CIS)-
Formula C15H24, MW 204, CAS# 483-76-1, Entry# 89277
1-ISOPROPYL-4,7-DIMETHYL-1,2,3,5,6,8A-HEXAHYDRONAPHTHALENE #

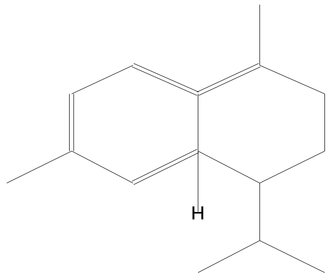

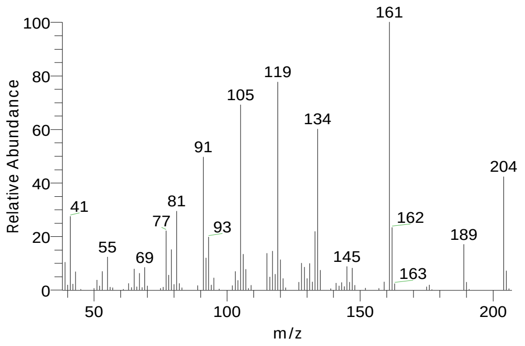

1-Isopropyl-4,7-dimethyl-1,2,3,5,6,8a-hexahydronaphthalene
Formula C15H24, MW 204, CAS# 16729-01-4, Entry# 150583
Cadina-1(10),4-diene

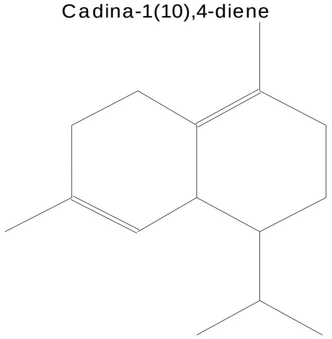

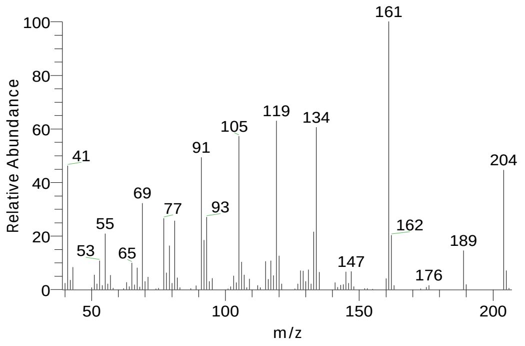

NAPHTHALENE, 1,2,3,5,6,8A-HEXAHYDRO-4,7-DIMETHYL-1-(1-METHYLETHYL)-, (1S-CIS)-
Formula C15H24, MW 204, CAS# 483-76-1, Entry# 89273
1-ISOPROPYL-4,7-DIMETHYL-1,2,3,5,6,8A-HEXAHYDRONAPHTHALENE #

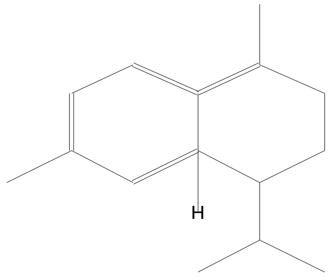

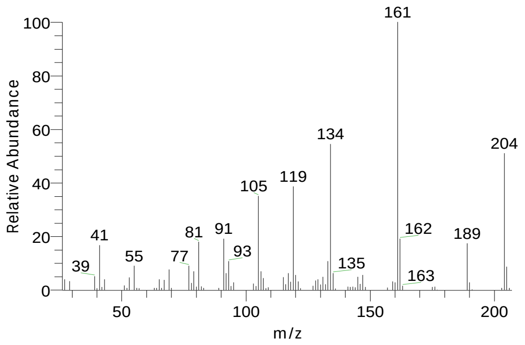

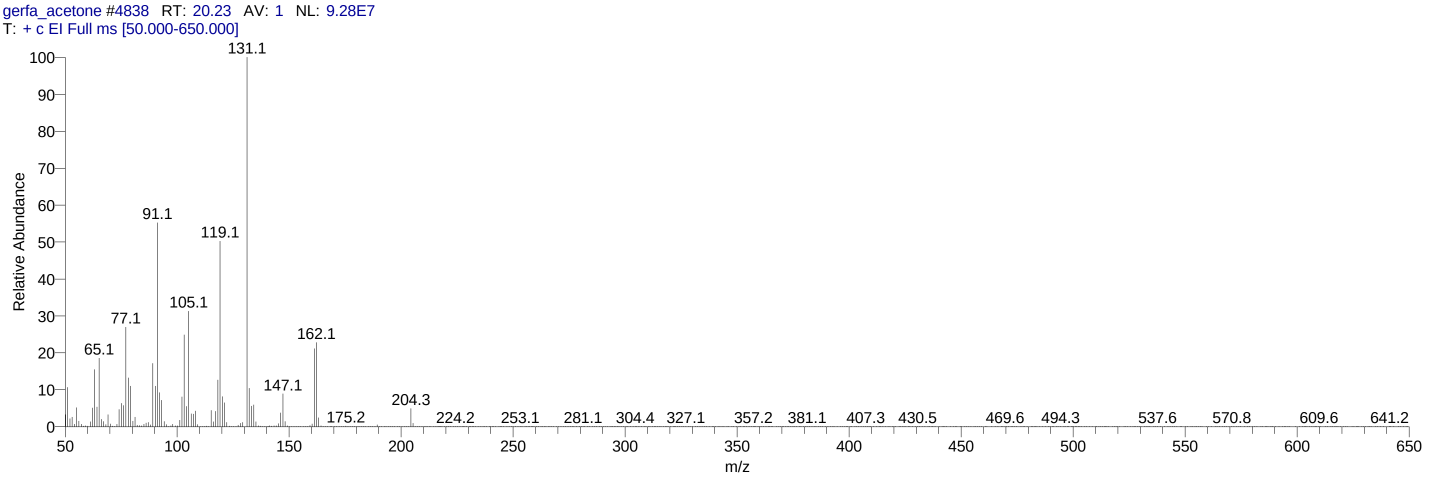

My GC-MS Report

RT	Compound Name	Area %	Molecular Formula	Molecular Weight	Cas #	MF	Library
20.23	2-Propenal, 3-(2-methoxyphenyl)-	5.04	C10H10O2	162	1504-74-1	881	mainlib
20.23	(2E)-3-(2-METHOXYPHENYL)-2-PROPENAL #	5.04	C10H10O2	162	1504-74-1	891	WileyRegistry8e
20.23	2-PROPENAL, 3-(4-METHOXYPHENYL)-	5.04	C10H10O2	162	1963-36-6	880	WileyRegistry8e

Compound Structure | Hit Spectrum

2-Propenal, 3-(2-methoxyphenyl)-
Formula C10H10O2, MW 162, CAS# 1504-74-1, Entry# 115637
Cinnamaldehyde, o-methoxy-

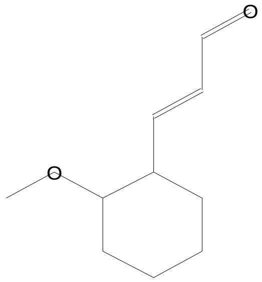

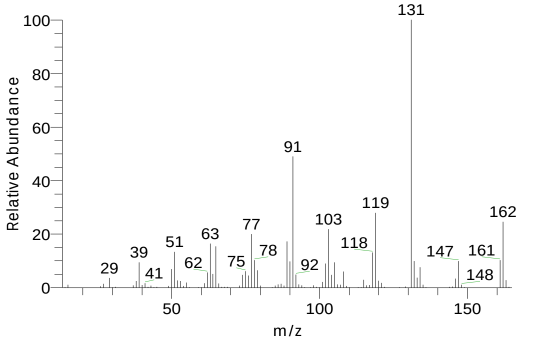

(2E)-3-(2-METHOXYPHENYL)-2-PROPENAL #
Formula C10H10O2, MW 162, CAS# 1504-74-1, Entry# 45156
(2E)-3-(2-METHOXYPHENYL)-2-PROPENAL

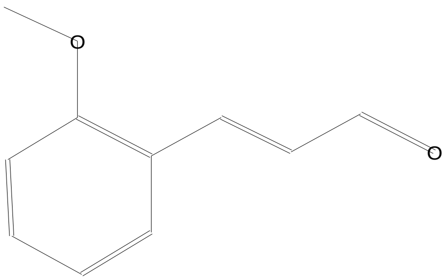

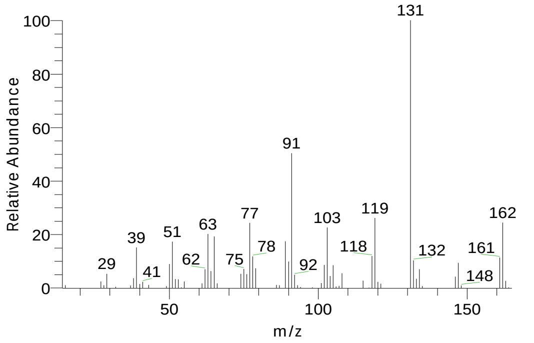

2-PROPENAL, 3-(4-METHOXYPHENYL)-
Formula C10H10O2, MW 162, CAS# 1963-36-6, Entry# 45236
CINNAMALDEHYDE, P-METHOXY-

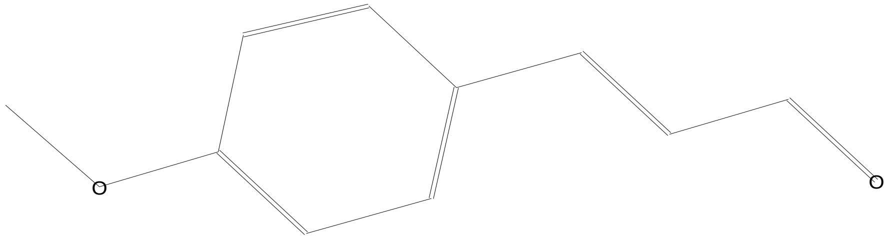

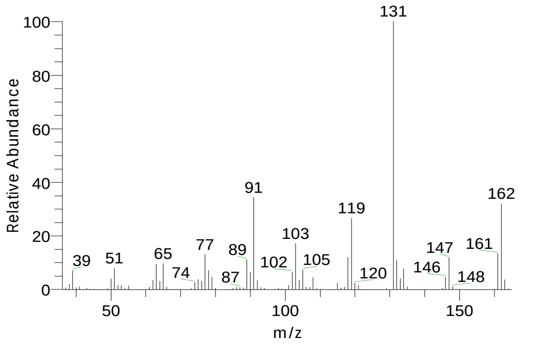

My GC-MS Report

gerfa_acetone #4911 RT: 20.47 AV: 1 NL: 1.43E7
T: + c EI Full ms [50.000-650.000]

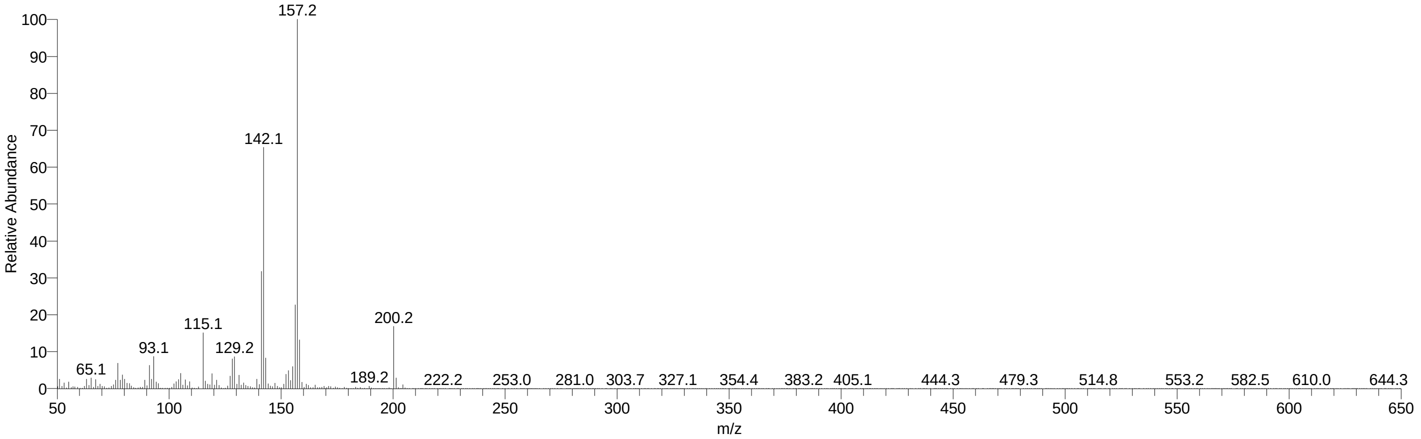

RT	Compound Name	Area %	Molecular Formula	Molecular Weight	Cas #	MF	Library
20.47	4-Isopropyl-6-methyl-1-methylene-1,2,3,4-tetrahydronaphthalene	0.44	C15H20	200	50277-34-4	926	mainlib
20.47	1-ISOPROPYL-4,7-DIMETHYL-1,2-DIHYDRONAPHTHALENE #	0.44	C15H20	200	21391-99-1	950	WileyRegistry8e
20.47	1,1,6-TRIMETHYL-1,2-DIHYDRO NAPHTHALENE	0.44	C13H16	172	NA	946	WileyRegistry8e

Compound Structure

Hit Spectrum

4-Isopropyl-6-methyl-1-methylene-1,2,3,4-tetrahydronaphthalene
Formula C15H20, MW 200, CAS# 50277-34-4, Entry# 146807
Naphthalene, 1,2,3,4-tetrahydro-6-methyl-1-methylene-4-(1-methylethyl)-

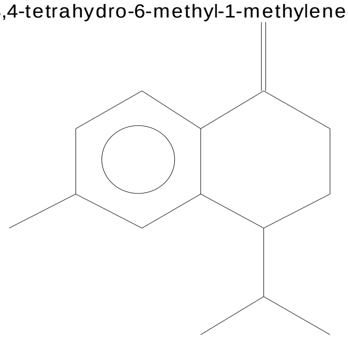

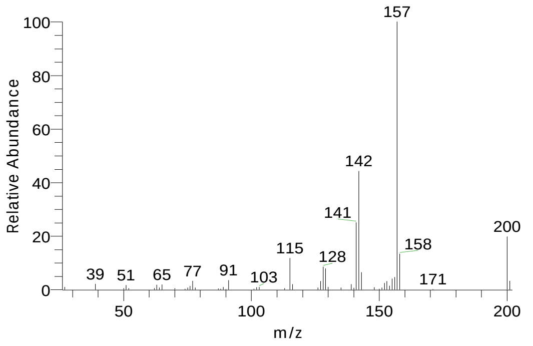

1-ISOPROPYL-4,7-DIMETHYL-1,2-DIHYDRONAPHTHALENE #
Formula C15H20, MW 200, CAS# 21391-99-1, Entry# 85030
à-CALACORENE

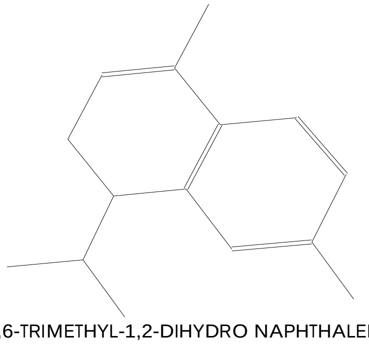

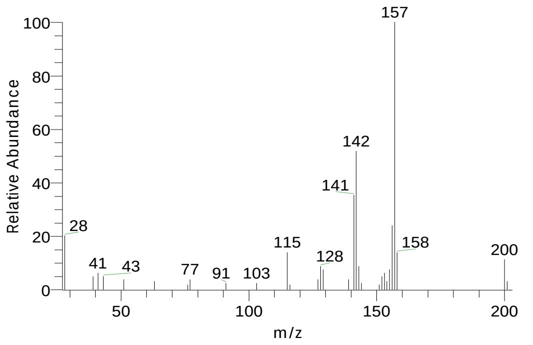

1,1,6-TRIMETHYL-1,2-DIHYDRO NAPHTHALENE
Formula C13H16, MW 172, CAS# NA, Entry# 56092
CALACORENE

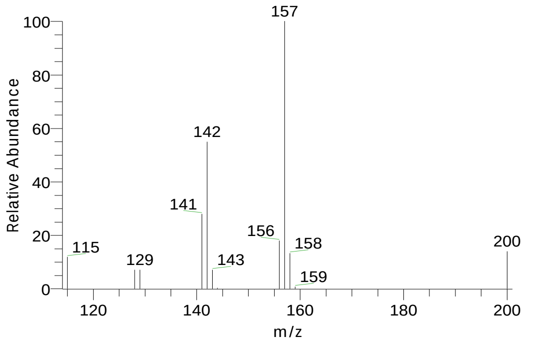

My GC-MS Report

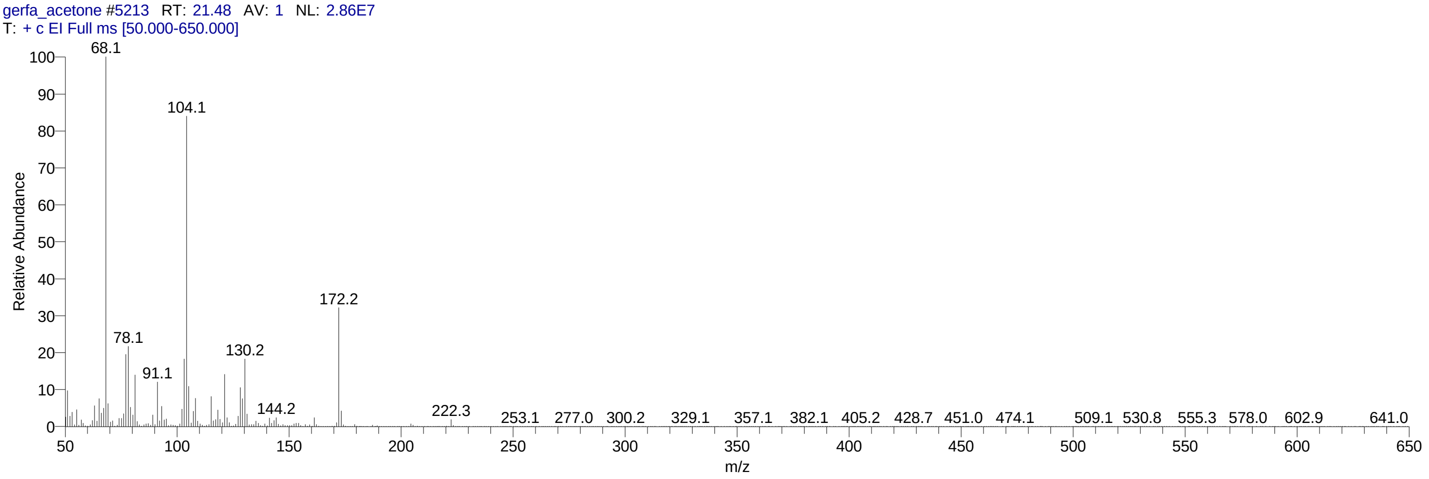

RT	Compound Name	Area %	Molecular Formula	Molecular Weight	Cas #	MF	Library
21.48	2-PROPANONE, 1-[2-(1-PROPYNYL)PHENYL]-	1.05	C12H12O	172	93769-24-5	901	WileyRegistry8e
21.48	1-Phenylbicyclo(4.1.0)heptane	1.05	C13H16	172	2415-82-9	676	mainlib
21.48	Benzene, [(cyclohex-1-en-1-yl)methyl]-	1.05	C13H16	172	4714-09-4	693	mainlib

Compound Structure	Hit Spectrum

2-PROPANONE, 1-[2-(1-PROPYNYL)PHENYL]-
Formula C12H12O, MW 172, CAS# 93769-24-5, Entry# 55965
1-[2-(1-PROPYN-1-YL)PHENYL]-2-PROPANOL

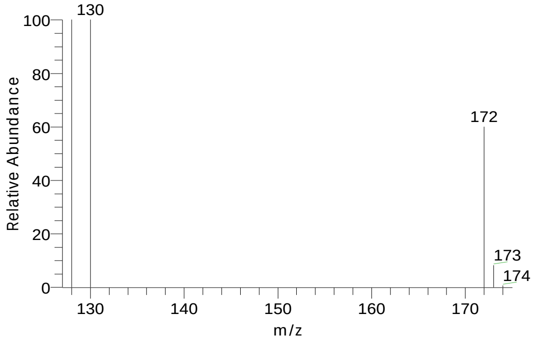

1-Phenylbicyclo(4.1.0)heptane
Formula C13H16, MW 172, CAS# 2415-82-9, Entry# 113978
$:28GJUWIDAFIWWNSH-UHFFFAOYSA-N

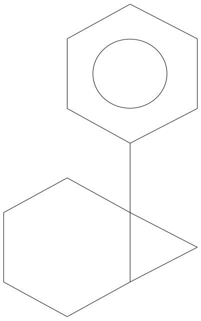

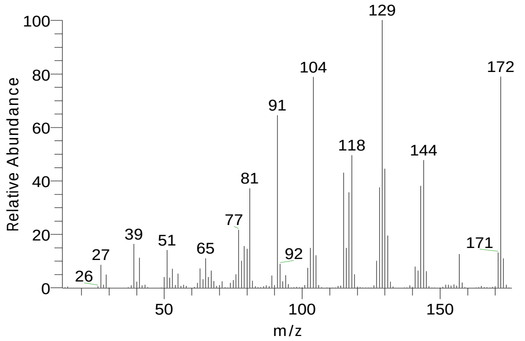

Benzene, [(cyclohex-1-en-1-yl)methyl]-
Formula C13H16, MW 172, CAS# 4714-09-4, Entry# 50602
(1-Cyclohexen-1-ylmethyl)benzene #

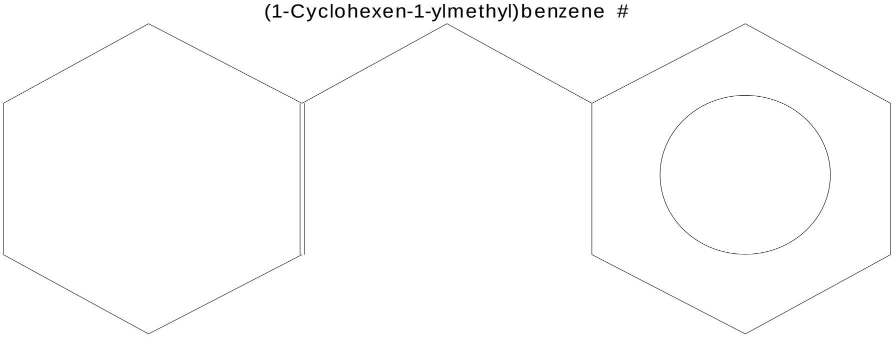

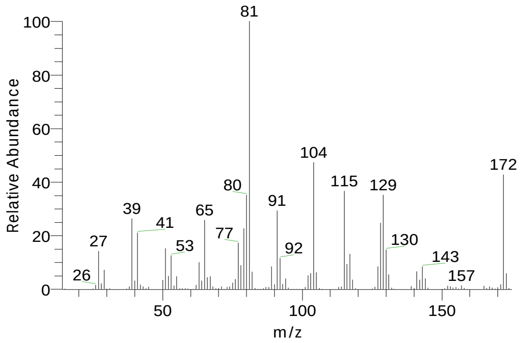

My GC-MS Report

gerfa_acetone #5510 RT: 22.48 AV: 1 NL: 9.29E6
T: + c EI Full ms [50.000-650.000]

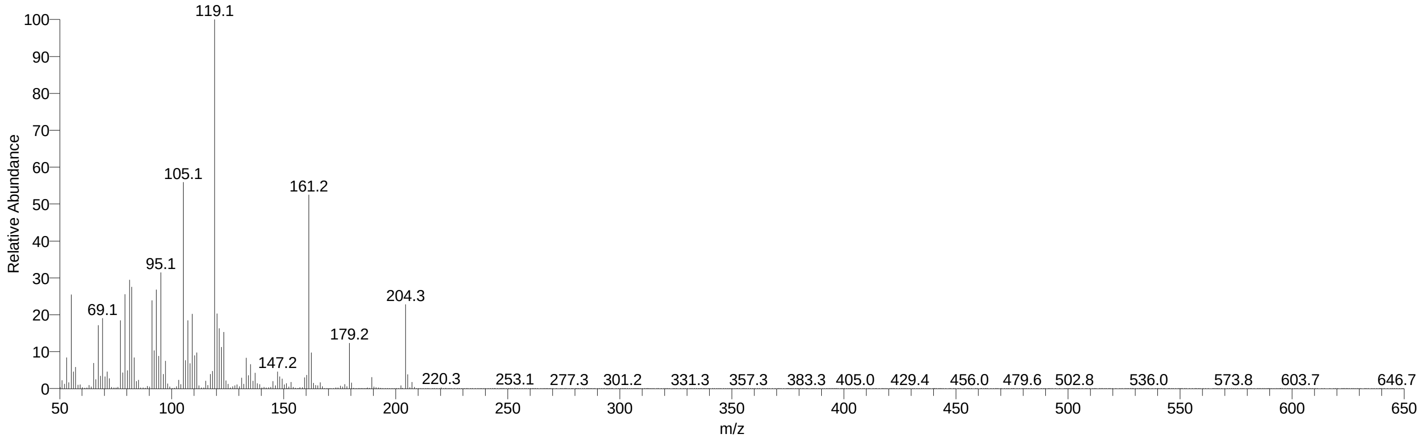

RT	Compound Name	Area %	Molecular Formula	Molecular Weight	Cas #	MF	Library
22.48	Di-epi-1,10-cubenol	0.41	C15H26O	222	73365-77-2	938	mainlib
22.48	Epicubenol	0.41	C15H26O	222	19912-67-5	931	mainlib
22.48	Cubenol	0.41	C15H26O	222	21284-22-0	912	mainlib

Compound Structure	Hit Spectrum

Di-epi-1,10-cubenol
Formula C15H26O, MW 222, CAS# 73365-77-2, Entry# 100531
(1S,4S,4aS,8aR)-1-Isopropyl-4,7-dimethyl-1,3,4,5,6,8a-hexahydronaphthalen-4a(2H)-ol

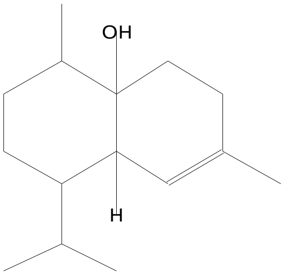

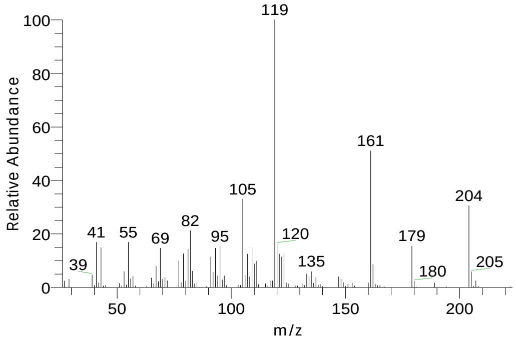

SI 915, RSI 931, mainlib, Entry# 100535, CAS# 19912-67-5, Epicubenol

Epicubenol
Formula C15H26O, MW 222, CAS# 19912-67-5, Entry# 100535
(1S,4R,4aS,8aR)-1-Isopropyl-4,7-dimethyl-1,2,3,4,4a,5,6,8a-octahydronaphthalen-4a-ol

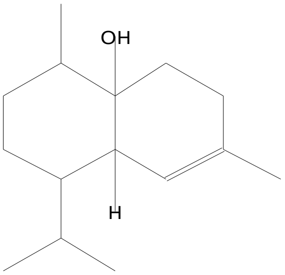

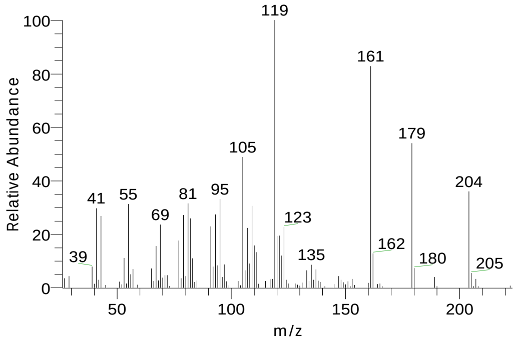

SI 889, RSI 912, mainlib, Entry# 98767, CAS# 21284-22-0, Cubenol

Cubenol
Formula C15H26O, MW 222, CAS# 21284-22-0, Entry# 98767
1-Isopropyl-4,7-dimethyl-1,3,4,5,6,8a-hexahydro-4a(2H)-naphthalenol-, [1S-(1à,4á,4aá,8aà)]-

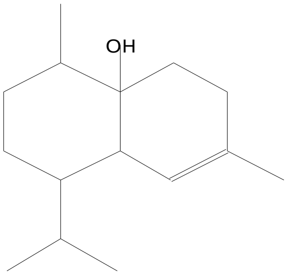

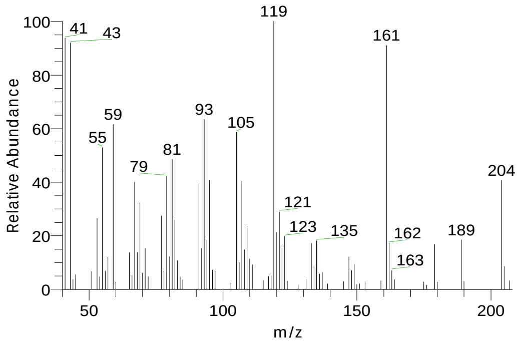

My GC-MS Report

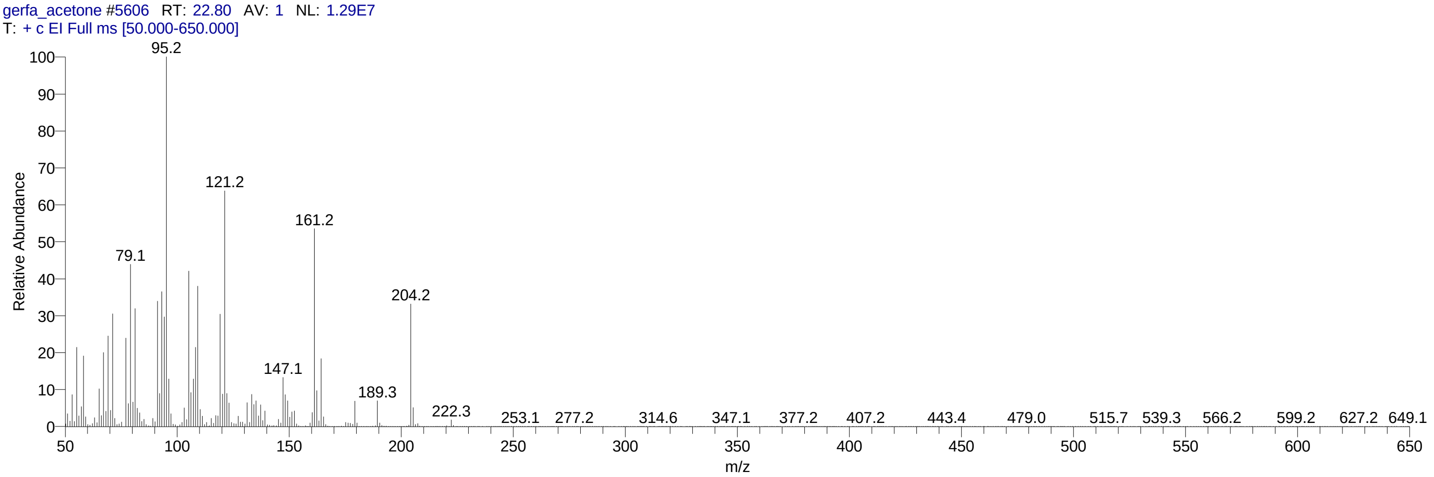

RT	Compound Name	Area %	Molecular Formula	Molecular Weight	Cas #	MF	Library
22.80	.tau.-Muurolol	0.67	C15H26O	222	19912-62-0	944	replib
22.80	à-Cadinol	0.67	C15H26O	222	481-34-5	927	replib
22.80	.tau.-Muurolol	0.67	C15H26O	222	19912-62-0	911	mainlib

Compound Structure | Hit Spectrum

.tau.-Muurolol
Formula C15H26O, MW 222, CAS# 19912-62-0, Entry# 3342
4-Isopropyl-1,6-dimethyl-1,2,3,4,4a,7,8,8a-octahydro-1-naphthalenol-, [1S-(1à,4à,4aà,8aà)]-

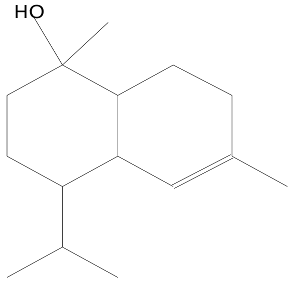

SI 926, RSI 944, replib, Entry# 3342, CAS# 19912-62-0, .tau.-Muurolol

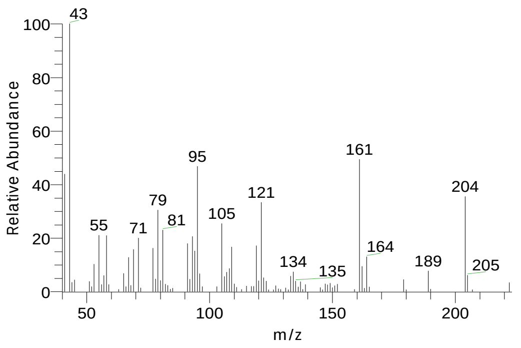

à-Cadinol
Formula C15H26O, MW 222, CAS# 481-34-5, Entry# 3029
4-Isopropyl-1,6-dimethyl-1,2,3,4,4a,7,8,8a-octahydro-1-naphthalenol #

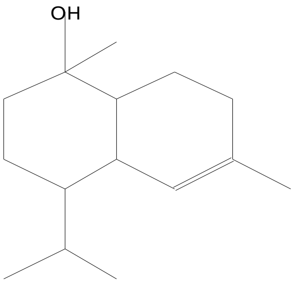

SI 916, RSI 927, replib, Entry# 3029, CAS# 481-34-5, à-Cadinol

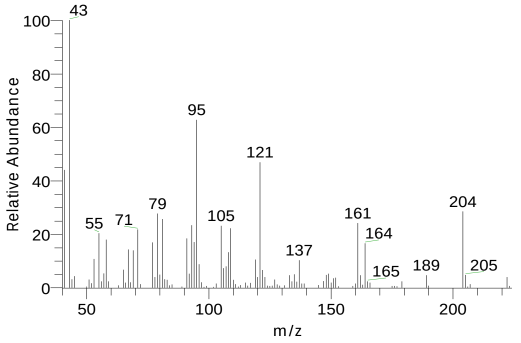

.tau.-Muurolol
Formula C15H26O, MW 222, CAS# 19912-62-0, Entry# 68534
4-Isopropyl-1,6-dimethyl-1,2,3,4,4a,7,8,8a-octahydro-1-naphthalenol-, [1S-(1à,4à,4aà,8aà)]-

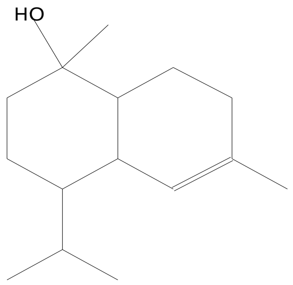

SI 910, RSI 911, mainlib, Entry# 68534, CAS# 19912-62-0, .tau.-Muurolol

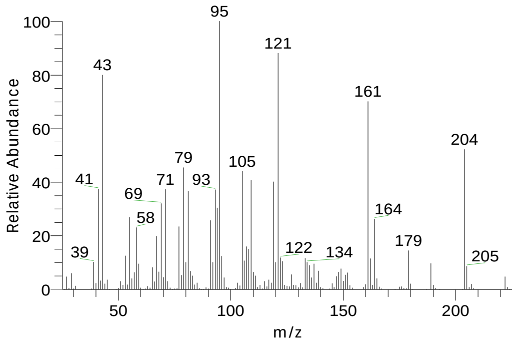

My GC-MS Report

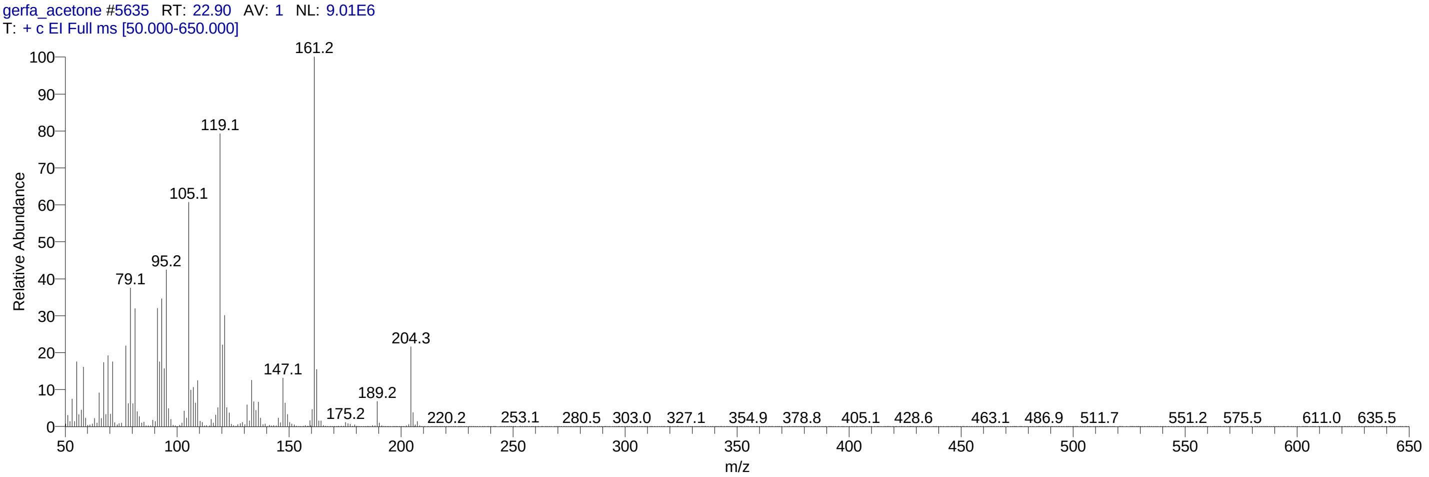

RT	Compound Name	Area %	Molecular Formula	Molecular Weight	Cas #	MF	Library
22.90	1-Naphthalenol, 1,2,3,4,4a,7,8,8a-octahydro-1,6-dimethyl-4-(1-methylethyl)-, [1R-(1à,4á,4aá,8aá)]-	0.28	C15H26O	222	19435-97-3	926	mainlib
22.90	1-NAPHTHALENOL, 1,2,3,4,4A,7,8,8A-OCTAHYDRO-1,6-DIMETHYL-4-(1-METHYLETHYL)-, [1R-(1à,4á,4Aá,8Aá)]-	0.28	C15H26O	222	19435-97-3	926	WileyRegistry8e
22.90	1-Naphthalenol, 1,2,3,4,4a,7,8,8a-octahydro-1,6-dimethyl-4-(1-methylethyl)-, [1R-(1à,4á,4aá,8aá)]-	0.28	C15H26O	222	19435-97-3	941	replib

Compound Structure	Hit Spectrum

Formula C15H26O, MW 222, CAS# 19435-97-3, Entry# 150252
(1R,4S,4aR,8aS)-4-Isopropyl-1,6-dimethyl-1,2,3,4,4a,7,8,8a-octahydronaphthalen-1-ol

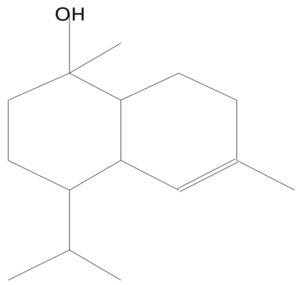

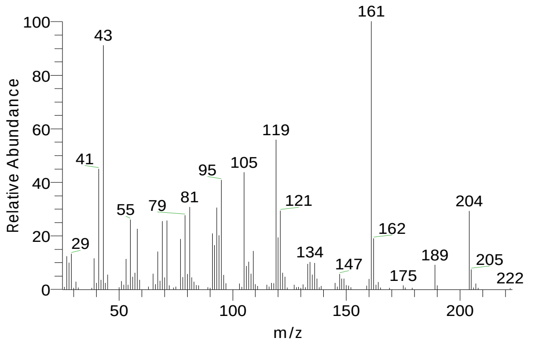

Formula C15H26O, MW 222, CAS# 19435-97-3, Entry# 109844
4-ISOPROPYL-1,6-DIMETHYL-1,2,3,4,4A,7,8,8A-OCTAHYDRO-1-NAPHTHALENOL #

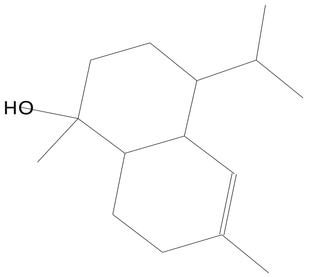

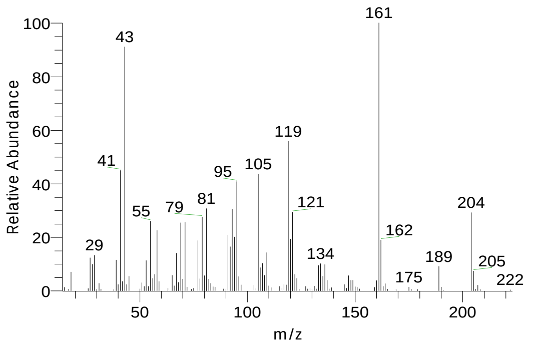

My GC-MS Report

Compound Structure | Hit Spectrum

Formula C15H26O, MW 222, CAS# 19435-97-3, Entry# 24848
(1R,4S,4aR,8aS)-4-Isopropyl-1,6-dimethyl-1,2,3,4,4a,7,8,8a-octahydronaphthalen-1-ol

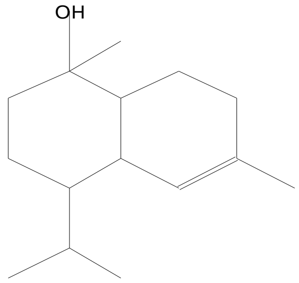

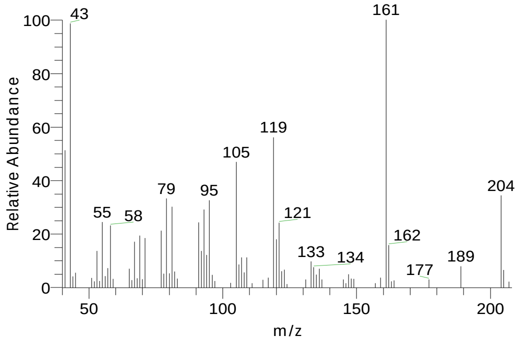

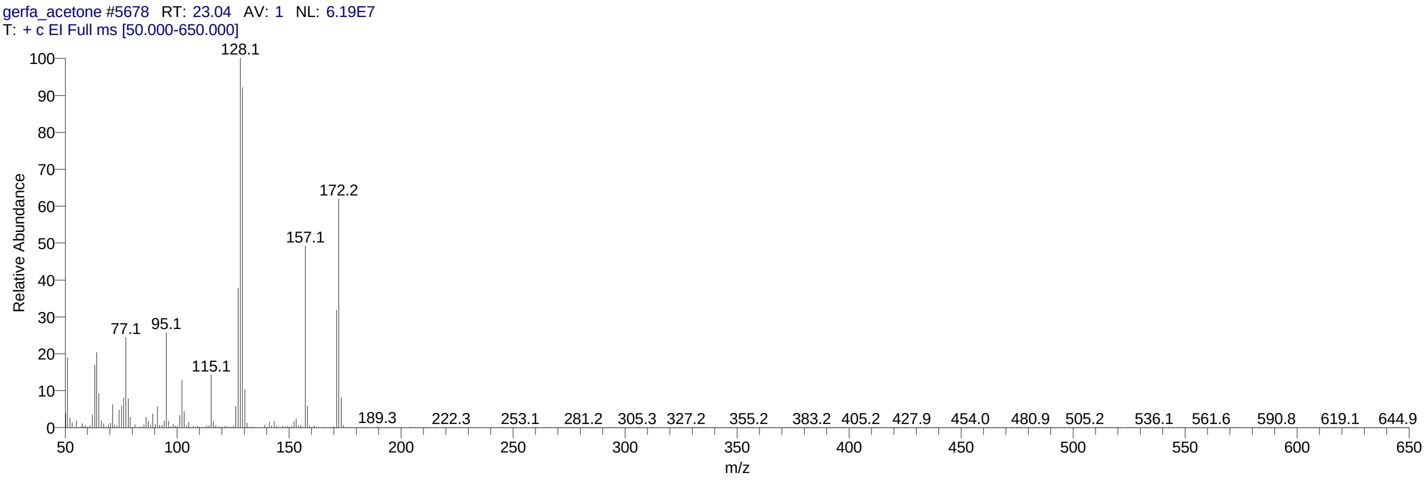

RT	Compound Name	Area %	Molecular Formula	Molecular Weight	Cas #	MF	Library
23.04	6-Phenyl-3,5-hexadien-2-one	3.15	C12H12O	172	4173-44-8	927	mainlib
23.04	1(4H)-NAPHTHALENONE, 4,4-DIMETHYL-	3.15	C12H12O	172	16020-16-9	958	WileyRegistry8e
23.04	(3'E,2R,2'RS)-2-T-BUTYL-6-[2'-HYDROXY-4'-PHENYL-3'-BUTENYL]-4H-1,3-DIOXIN-4-ONE	3.15	C18H22O4	302	NA	897	WileyRegistry8e

Compound Structure | Hit Spectrum

6-Phenyl-3,5-hexadien-2-one
Formula C12H12O, MW 172, CAS# 4173-44-8, Entry# 112566
6-Phenylhexa-3,5-dien-2-one

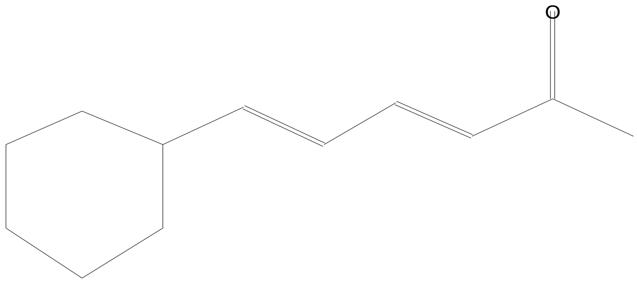

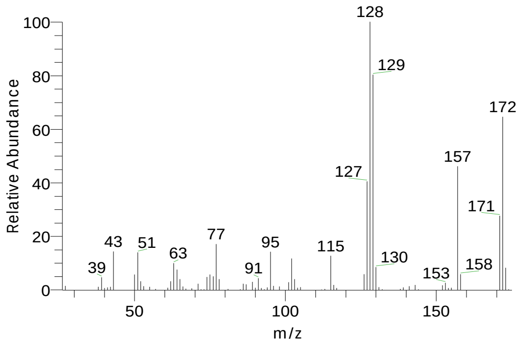

My GC-MS Report

Compound Structure | Hit Spectrum

1(4H)-NAPHTHALENONE, 4,4-DIMETHYL-
Formula C12H12O, MW 172, CAS# 16020-16-9, Entry# 55977
4,4-DIMETHYLNAPHTHALEN-1(4H)-ONE

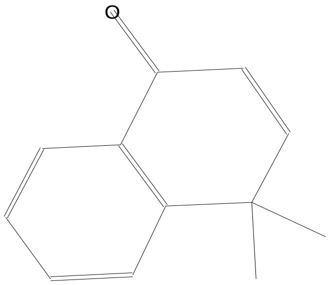

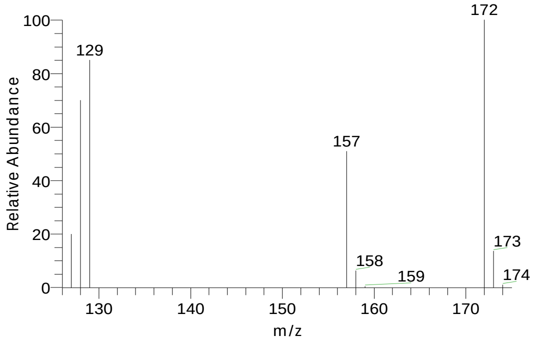

(3'E,2R,2'RS)-2-T-BUTYL-6-[2'-HYDROXY-4'-PHENYL-3'-BUTENYL]-4H-1,3-DIOXIN-4-ONE
Formula C18H22O4, MW 302, CAS# NA, Entry# 191419

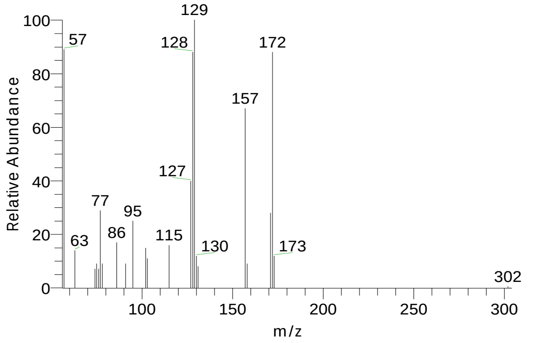

Supplement: Supplementary file 1 [file molecules-25-00996-s001.zip › molecules-727712-SM-final/Figure S1.pdf]
