# Supplementary material for: Phytochemical Characterization and Chemotherapeutic Potential of Cinnamomum verum Extracts on the Multiplication of Protozoan Parasites In Vitro and In Vivo
Source: Molecules. 2020 Feb 24;25(4):996. doi: 10.3390/molecules25040996 (PMC7070835; doi:10.3390/molecules25040996)

My GC-MS Report

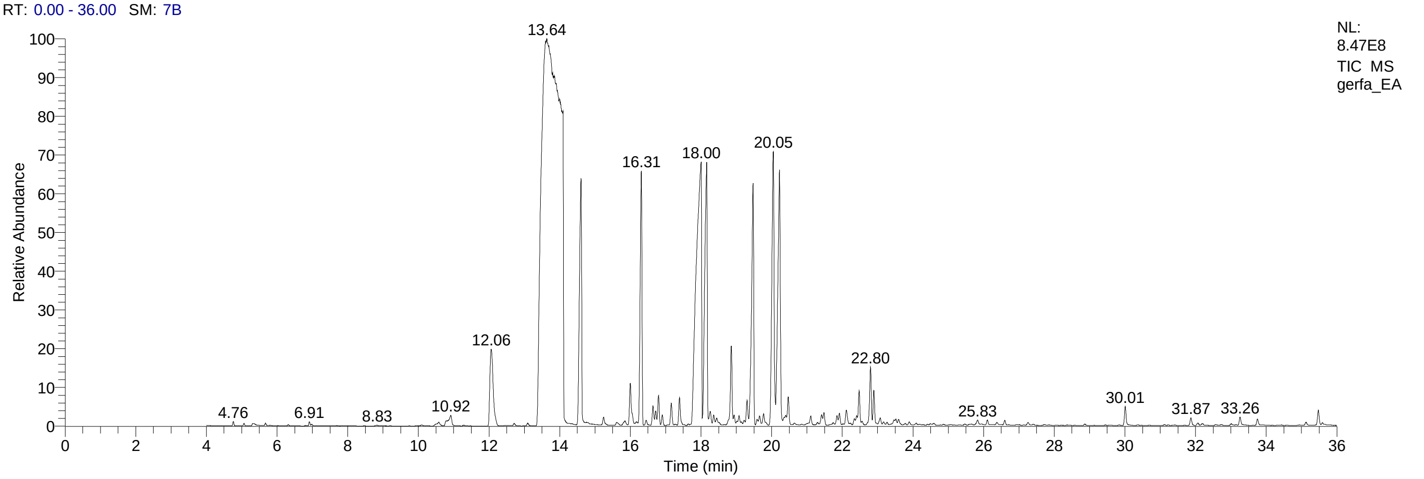

RT	Area %	Peak Area
10.92	0.24	128173820.51
12.06	1.94	1035114737.02
13.64	53.81	28642212603.14
14.60	4.27	2271905049.14
16.00	0.62	331863513.20
16.31	3.68	1956479496.62
16.64	0.24	127515039.35
16.72	0.16	84334567.09
16.80	0.35	188369875.93
16.90	0.11	57520312.66
17.16	0.28	146669995.78
17.39	0.38	200364399.84
18.00	9.92	5280783018.22
18.16	4.83	2570696982.40
18.26	0.11	59233142.90
18.86	1.05	557328644.63
19.30	0.26	137888084.26
19.47	4.14	2201097941.66
19.66	0.12	63533698.33
19.77	0.18	96526750.45
20.04	4.78	2545719837.35
20.22	4.91	2615571936.08
20.47	0.43	228816648.36
21.11	0.14	75018362.93
21.41	0.14	76347365.03
21.48	0.16	86057337.89
21.85	0.11	60872365.51
21.92	0.14	75870020.41
22.12	0.22	118894434.31
22.48	0.53	283703031.75
22.80	0.77	411968559.79
22.89	0.37	199251685.20
30.01	0.25	135159887.79
33.26	0.12	64939646.82
35.47	0.21	109604833.07

My GC-MS Report

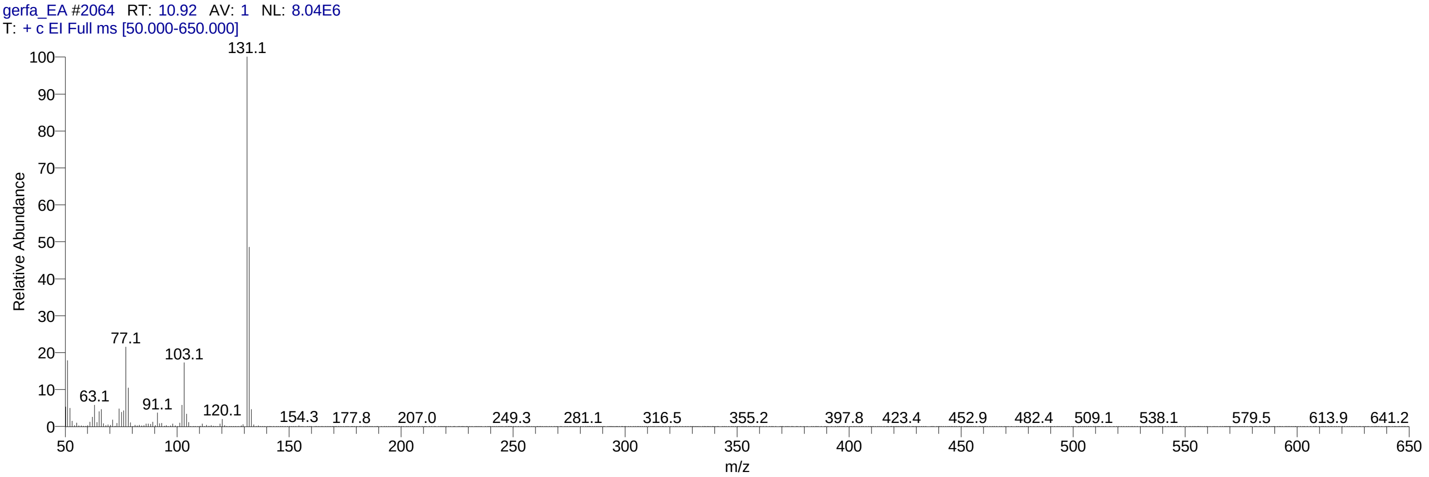

RT	Compound Name	Area %	Molecular Formula	Molecular Weight	Cas #	MF	Library
10.92	Benzofuran, 2-methyl-	0.24	C9H8O	132	4265-25-2	901	mainlib
10.92	Benzofuran, 2-methyl-	0.24	C9H8O	132	4265-25-2	905	replib
10.92	BENZOFURAN, 2-METHYL-	0.24	C9H8O	132	4265-25-2	905	WileyRegistry8e

Compound Structure	Hit Spectrum

Benzofuran, 2-methyl-
Formula C9H8O, MW 132, CAS# 4265-25-2, Entry# 115872
2-Methylbenzofuran

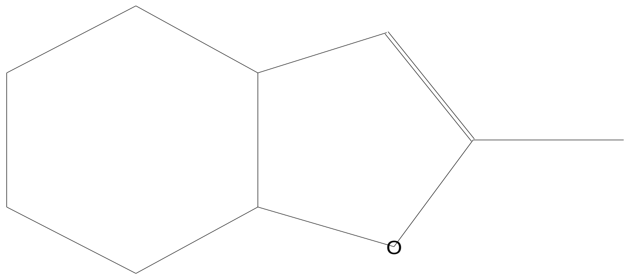

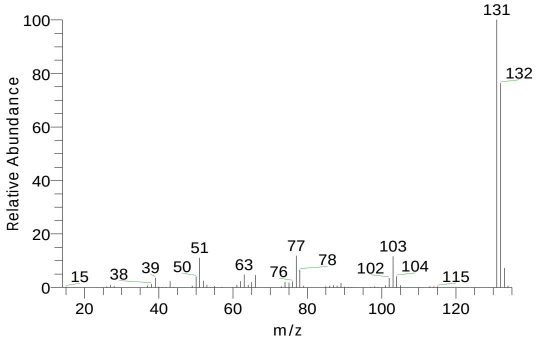

Benzofuran, 2-methyl-
Formula C9H8O, MW 132, CAS# 4265-25-2, Entry# 20943
2-Methylbenzofuran

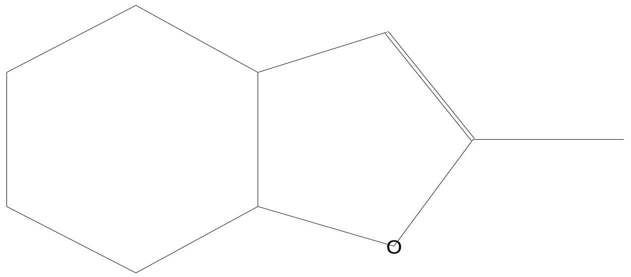

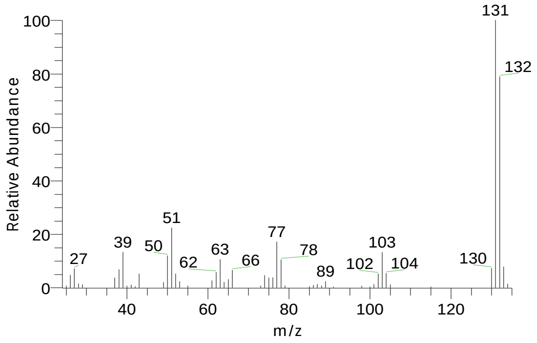

BENZOFURAN, 2-METHYL-
Formula C9H8O, MW 132, CAS# 4265-25-2, Entry# 20576
2-METHYLBENZOFURAN

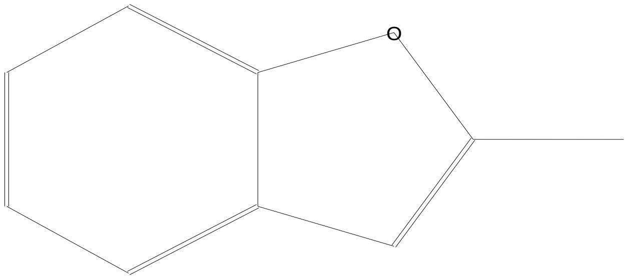

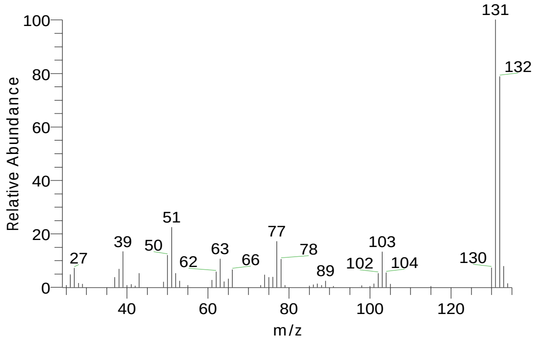

My GC-MS Report

gerfa_EA #2405 RT: 12.06 AV: 1 NL: 4.21E7
T: + c EI Full ms [50.000-650.000]

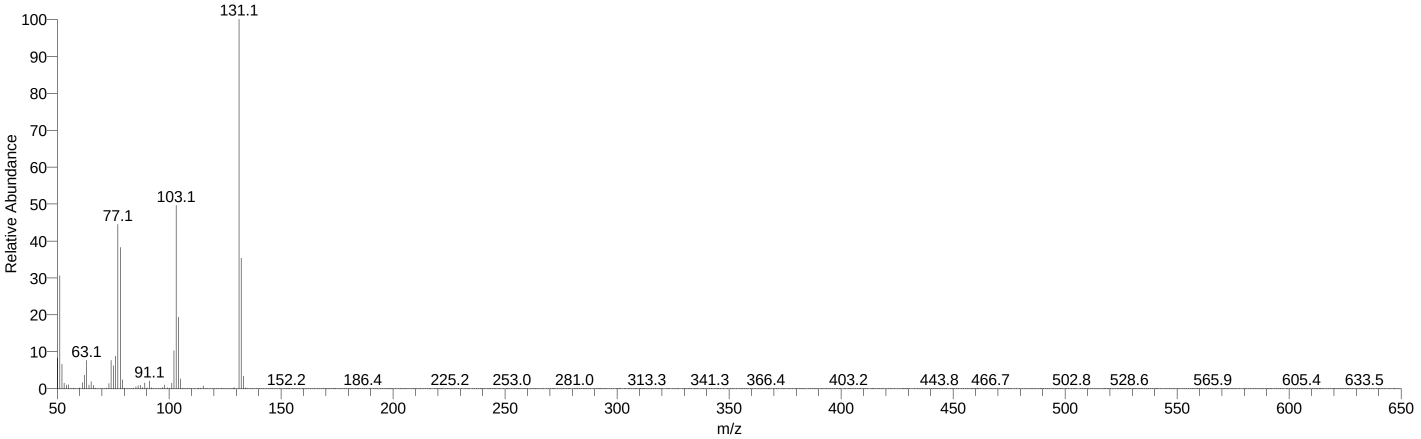

RT	Compound Name	Area %	Molecular Formula	Molecular Weight	Cas #	MF	Library
12.06	(Z)-3-Phenylacrylaldehyde	1.94	C9H8O	132	57194-69-1	960	mainlib
12.06	2-Propenal, 3-phenyl-	1.94	C9H8O	132	104-55-2	949	replib
12.06	Cinnamaldehyde, (E)-	1.94	C9H8O	132	14371-10-9	949	mainlib

Compound Structure	Hit Spectrum

(Z)-3-Phenylacrylaldehyde
Formula C9H8O, MW 132, CAS# 57194-69-1, Entry# 115751
2-Propenal, 3-phenyl-, (2Z)-

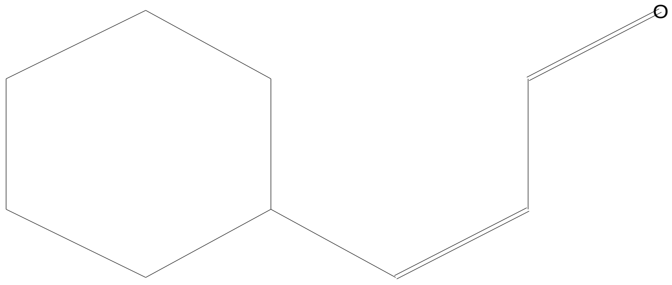

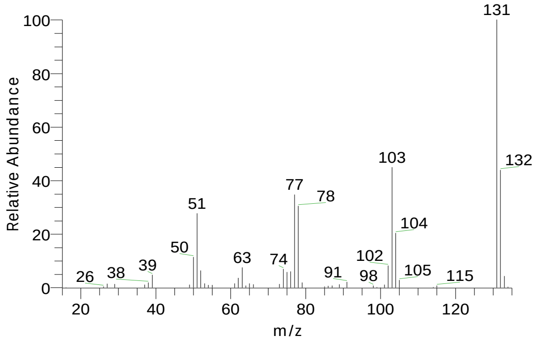

2-Propenal, 3-phenyl-
Formula C9H8O, MW 132, CAS# 104-55-2, Entry# 20950
Cinnamaldehyde

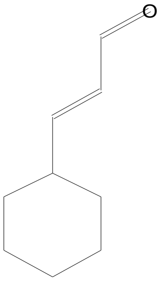

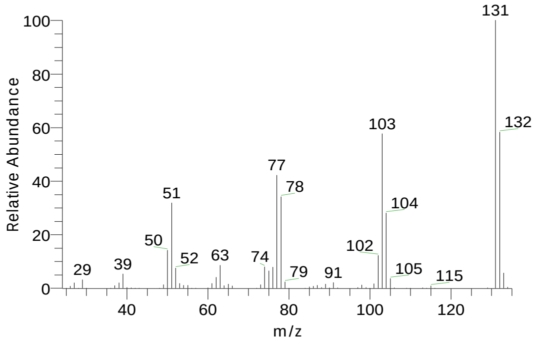

Cinnamaldehyde, (E)-
Formula C9H8O, MW 132, CAS# 14371-10-9, Entry# 115750
(E)-Cinnamaldehyde

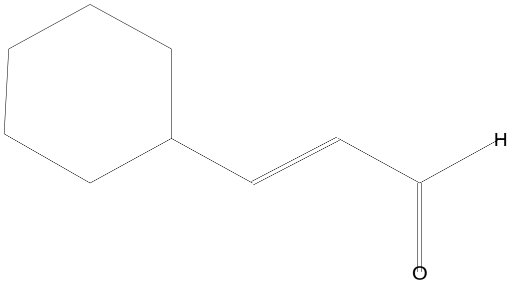

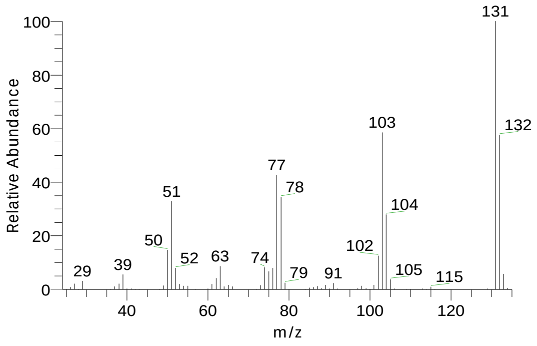

My GC-MS Report

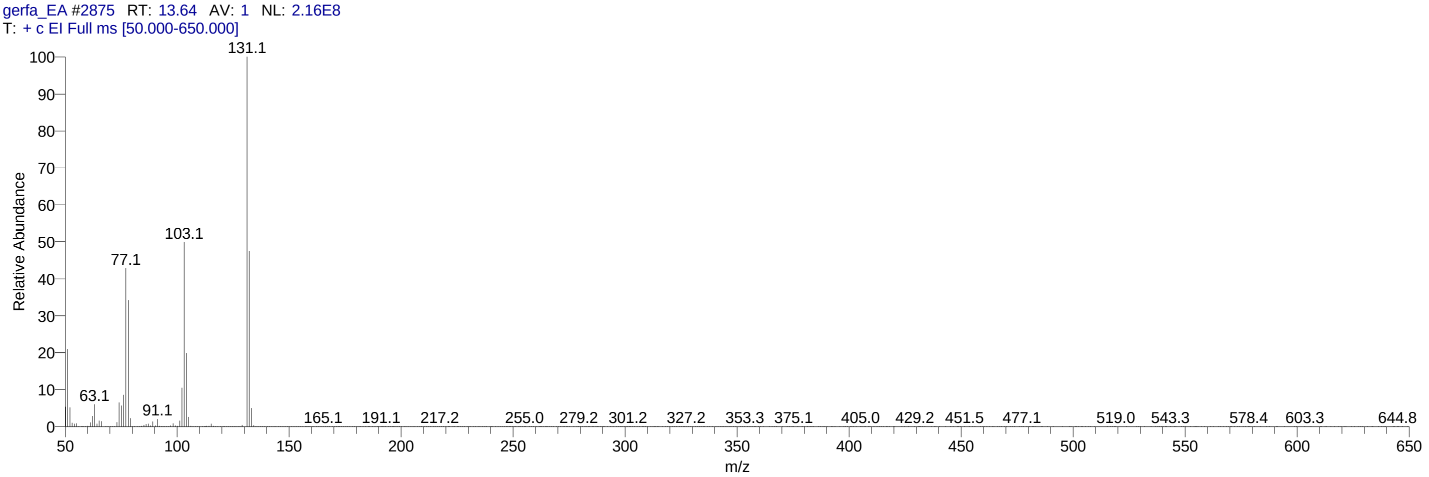

RT	Compound Name	Area %	Molecular Formula	Molecular Weight	Cas #	MF	Library
13.64	(Z)-3-Phenylacrylaldehyde	53.81	C9H8O	132	57194-69-1	959	mainlib
13.64	2-Propenal, 3-phenyl-	53.81	C9H8O	132	104-55-2	943	replib
13.64	Cinnamaldehyde, (E)-	53.81	C9H8O	132	14371-10-9	943	mainlib

Compound Structure	Hit Spectrum

(Z)-3-Phenylacrylaldehyde
Formula C9H8O, MW 132, CAS# 57194-69-1, Entry# 115751
2-Propenal, 3-phenyl-, (2Z)-

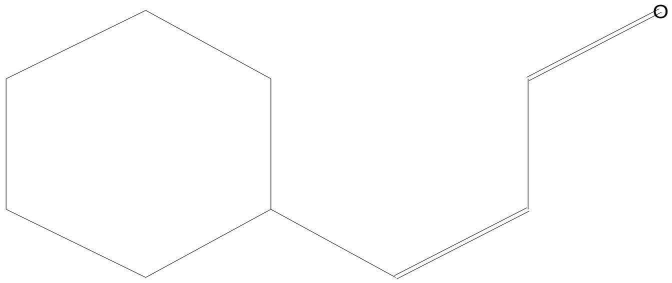

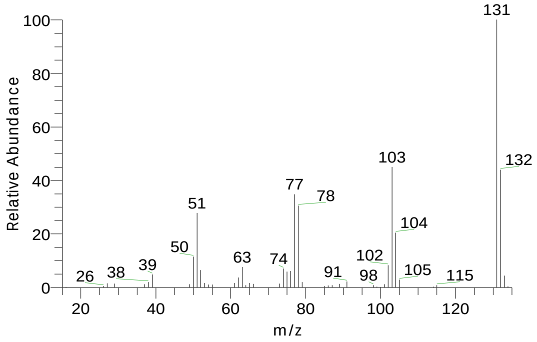

2-Propenal, 3-phenyl-
Formula C9H8O, MW 132, CAS# 104-55-2, Entry# 20950
Cinnamaldehyde

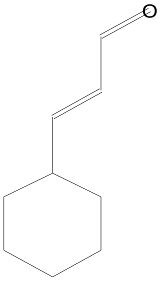

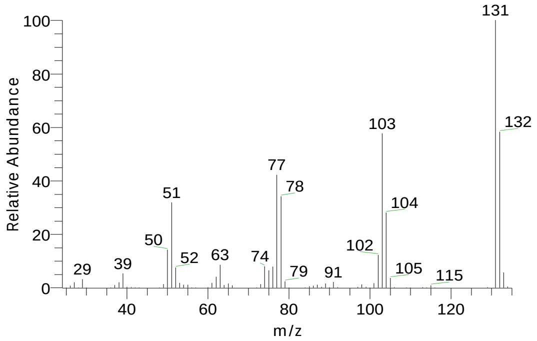

Cinnamaldehyde, (E)-
Formula C9H8O, MW 132, CAS# 14371-10-9, Entry# 115750
(E)-Cinnamaldehyde

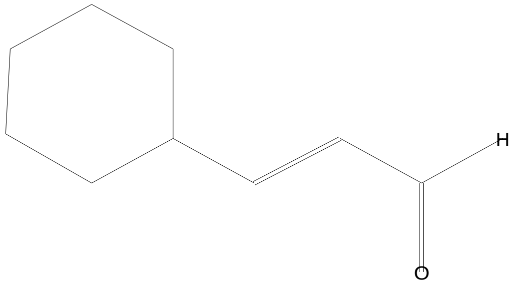

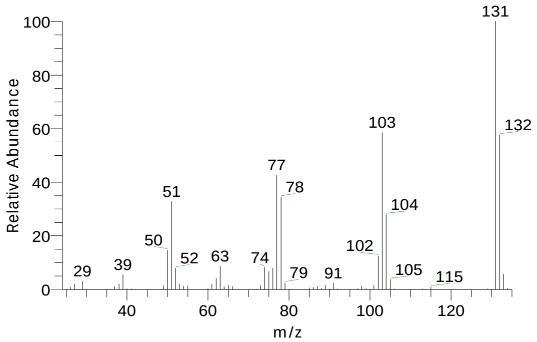

My GC-MS Report

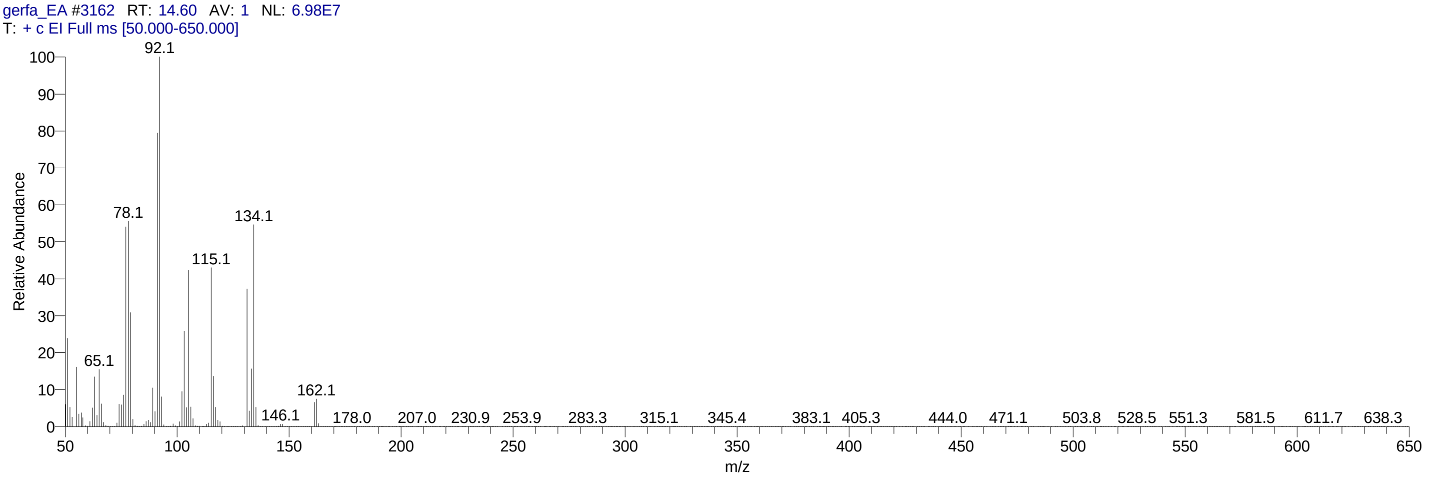

RT	Compound Name	Area %	Molecular Formula	Molecular Weight	Cas #	MF	Library
14.60	2-Propen-1-ol, 3-phenyl-, (E)-	4.27	C9H10O	134	4407-36-7	926	mainlib
14.60	2-Propen-1-ol, 3-phenyl-	4.27	C9H10O	134	104-54-1	914	mainlib
14.60	2-Propen-1-ol, 3-phenyl-	4.27	C9H10O	134	104-54-1	908	replib

Compound Structure | Hit Spectrum

2-Propen-1-ol, 3-phenyl-, (E)-
Formula C9H10O, MW 134, CAS# 4407-36-7, Entry# 65164
$:28OOCCDEMITAIZTP-QPJJXVBHSA-N

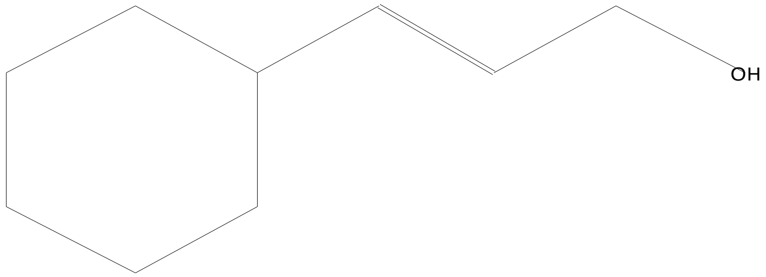

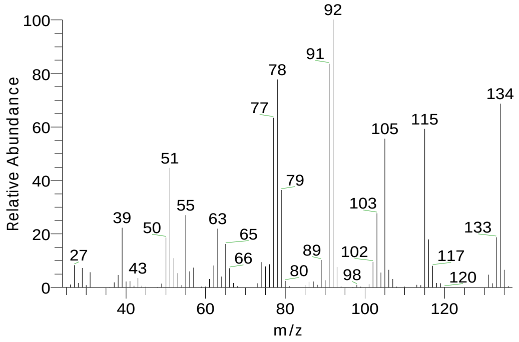

2-Propen-1-ol, 3-phenyl-
Formula C9H10O, MW 134, CAS# 104-54-1, Entry# 65165
Cinnamyl alcohol

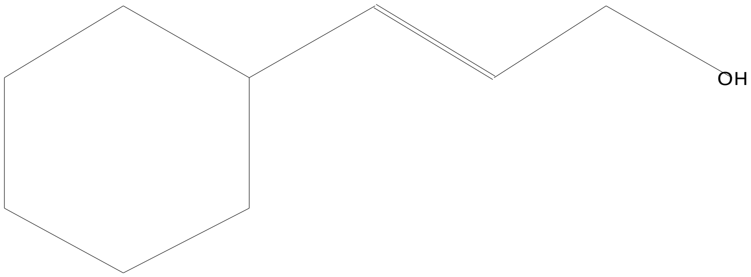

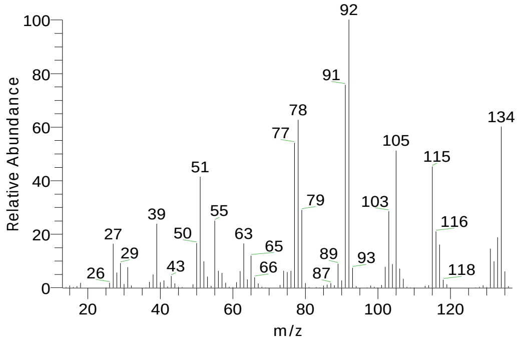

2-Propen-1-ol, 3-phenyl-
Formula C9H10O, MW 134, CAS# 104-54-1, Entry# 13932
Cinnamyl alcohol

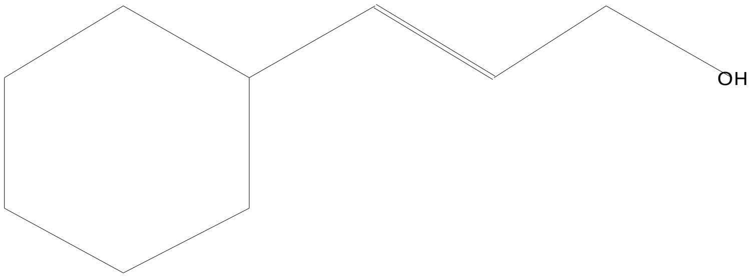

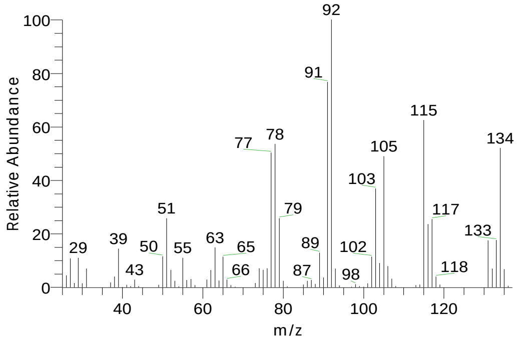

My GC-MS Report

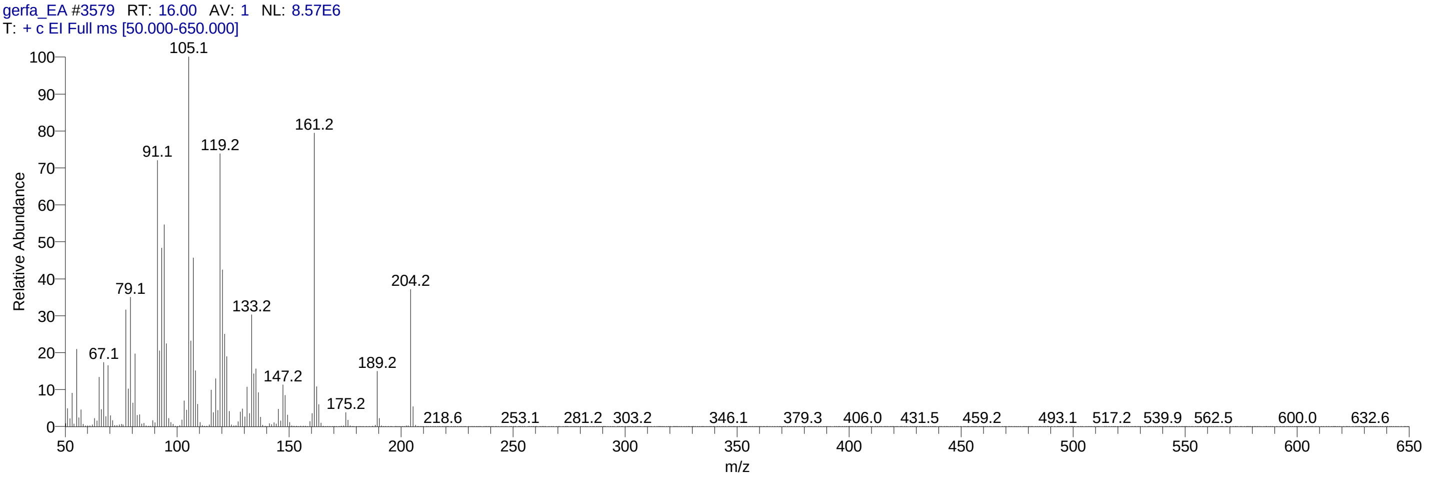

RT	Compound Name	Area %	Molecular Formula	Molecular Weight	Cas #	MF	Library
16.00	1,2,4-Metheno-1H-indene, octahydro-1,7a-dimethyl-5-(1-methylethyl)-, [1S-(1à,2à,3aá,4à,5à,7aá,8S*)]-	0.62	C15H24	204	22469-52-9	935	replib
16.00	(+)-CYCLOISOSATIVENE	0.62	C15H24	204	NA	915	WileyRegistry8e
16.00	1,2,4-Metheno-1H-indene, octahydro-1,7a-dimethyl-5-(1-methylethyl)-, [1S-(1à,2à,3aá,4à,5à,7aá,8S*)]-	0.62	C15H24	204	22469-52-9	911	replib

Compound Structure	Hit Spectrum

Formula C15H24, MW 204, CAS# 22469-52-9, Entry# 16426
(+)-Cyclosativene

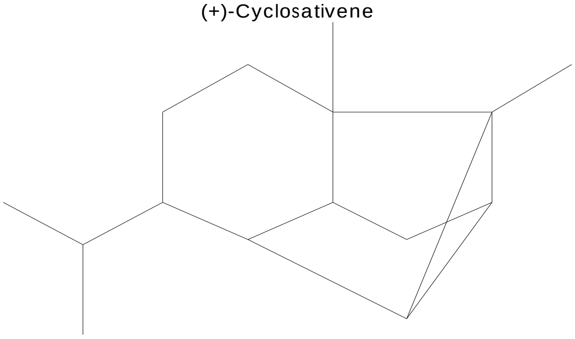

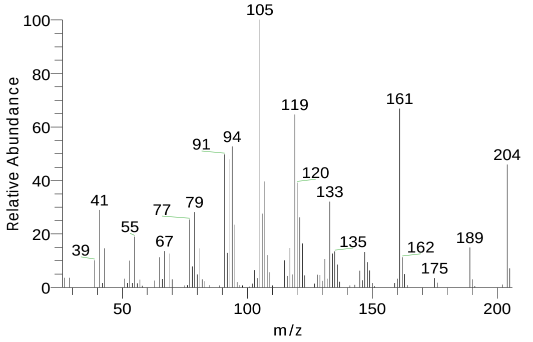

(+)-CYCLOISOSATIVENE
Formula C15H24, MW 204, CAS# NA, Entry# 89651

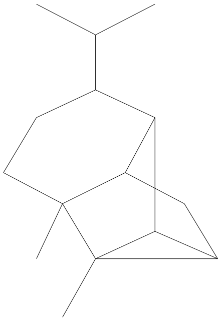

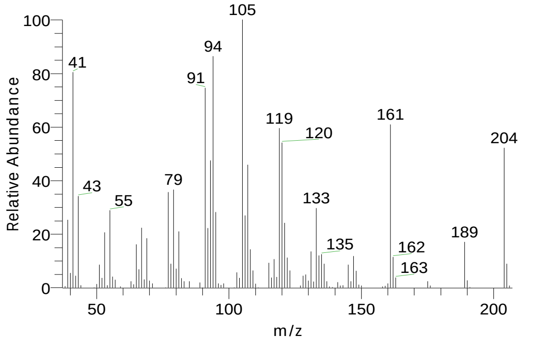

My GC-MS Report

Compound Structure | Hit Spectrum

Formula C15H24, MW 204, CAS# 22469-52-9, Entry# 16246
(+)-Cyclosativene

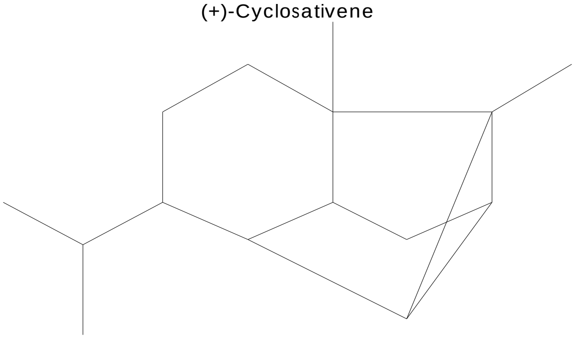

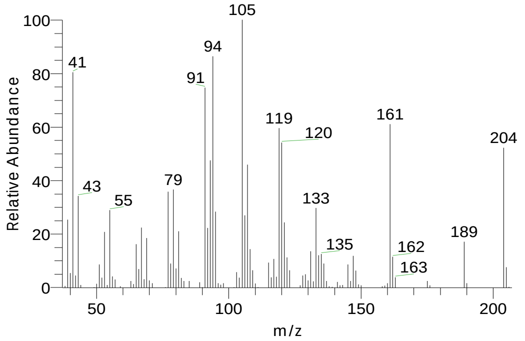

gerfa_EA #3671 RT: 16.31 AV: 1 NL: 7.59E7
T: + c EI Full ms [50.000-650.000]

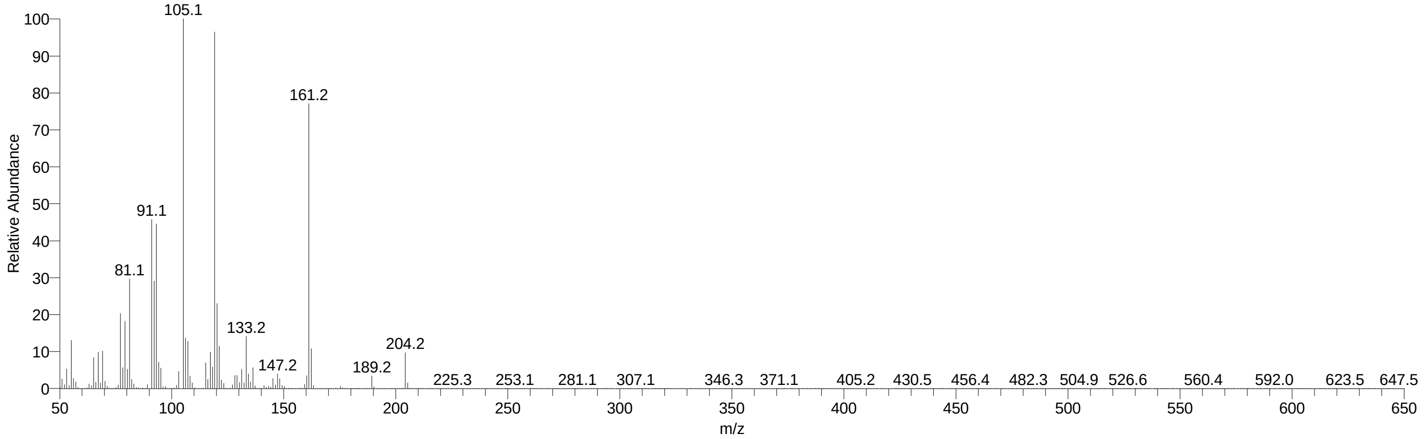

RT	Compound Name	Area %	Molecular Formula	Molecular Weight	Cas #	MF	Library
16.31	TRICYCLO[4.4.0.0(2,7)]DEC-3-ENE, 1,3-DIMETHYL-8-(1-METHYLETHYL)-, ST	3.68	C15H24	204	3856-25-5	951	WileyRegistry8e
16.31	.alfa.-Copaene	3.68	C15H24	204	NA	930	mainlib
16.31	TRICYCLO[4.4.0.0(2,7)]DEC-3-ENE, 1,3-DIMETHYL-8-(1-METHYLETHYL)-, ST	3.68	C15H24	204	3856-25-5	952	WileyRegistry8e

Compound Structure | Hit Spectrum

TRICYCLO[4.4.0.0(2,7)]DEC-3-ENE, 1,3-DIMETHYL-8-(1-METHYLETHYL)-, ST
Formula C15H24, MW 204, CAS# 3856-25-5, Entry# 89462
TRICYCLO[4.4.0.0(2,7)]DEC-3-ENE, 1,3-DIMETHYL-8-(1-METHYLETHYL)-, STEREOISOMER

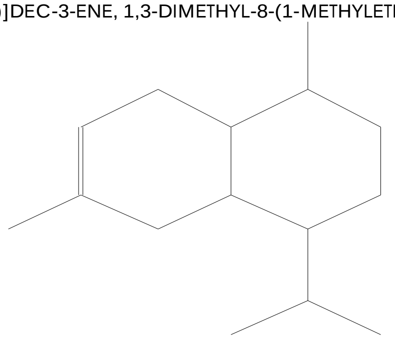

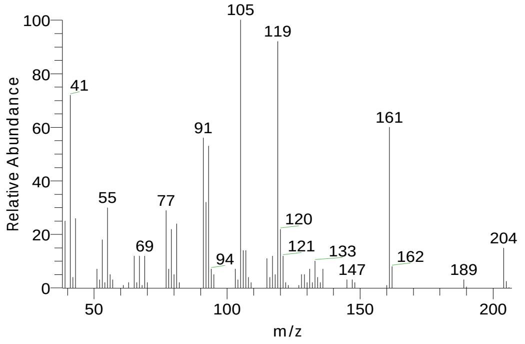

My GC-MS Report

Compound Structure | Hit Spectrum

.alfa.-Copaene
Formula C15H24, MW 204, CAS# NA, Entry# 150578
$:28VLXDPFLIRFYIME-UHFFFAOYSA-N

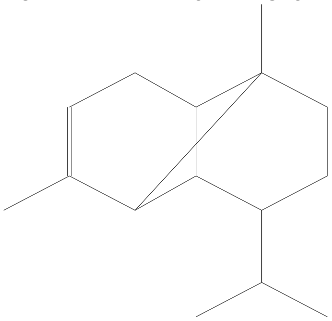

SI 928, RSI 930, mainlib, Entry# 150578, CAS# NA, .alfa.-Copaene

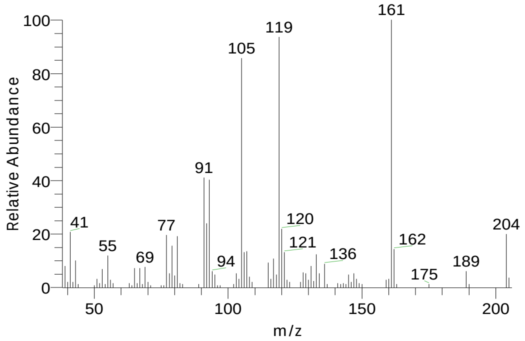

TRICYCLO[4.4.0.0(2,7)]DEC-3-ENE, 1,3-DIMETHYL-8-(1-METHYLETHYL)-, ST
Formula C15H24, MW 204, CAS# 3856-25-5, Entry# 89463
TRICYCLO[4.4.0.0(2,7)]DEC-3-ENE, 1,3-DIMETHYL-8-(1-METHYLETHYL)-, STEREOISOMER

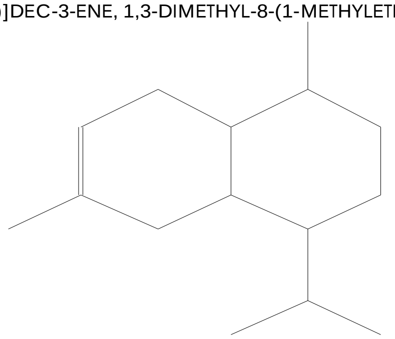

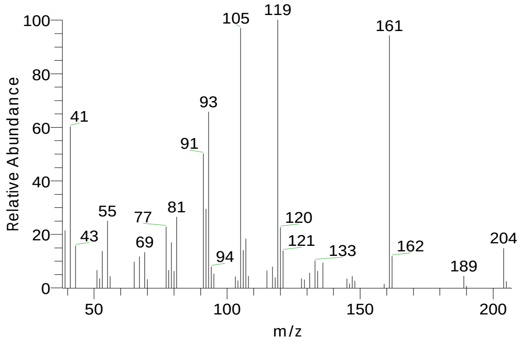

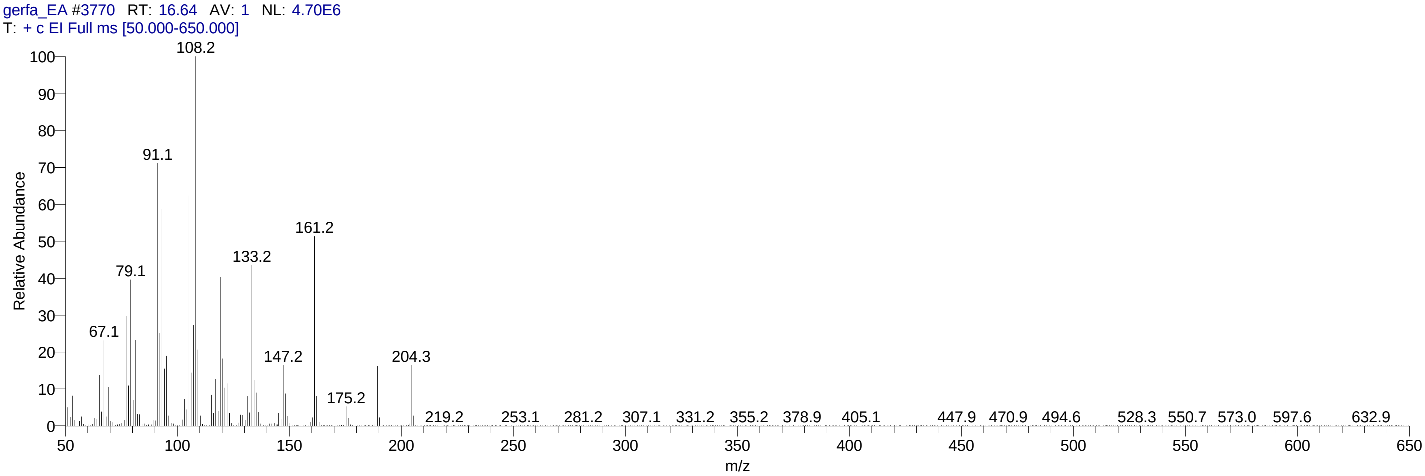

RT	Compound Name	Area %	Molecular Formula	Molecular Weight	Cas #	MF	Library
16.64	1,4-Methano-1H-indene, octahydro-4-methyl-8-methylene-7-(1-methylethyl)-, [1S-(1à,3aá,4à,7à,7aá)]-	0.24	C15H24	204	3650-28-0	915	mainlib
16.64	(+)-SATIVEN	0.24	C15H24	204	3650-28-0	915	WileyRegistry8e
16.64	1,4-METHANO-1H-INDENE, OCTAHYDRO-4-METHYL-8-METHYLENE-7-(1-METHYLETHYL)-, [1S-(1à,3Aá,4à,7à,7Aá)]-	0.24	C15H24	204	3650-28-0	915	WileyRegistry8e

My GC-MS Report

Compound Structure | Hit Spectrum

Formula C15H24, MW 204, CAS# 3650-28-0, Entry# 84625
(+)-Sativen

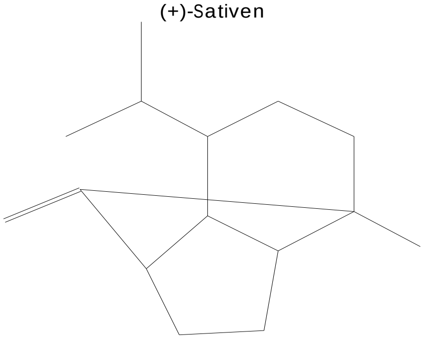

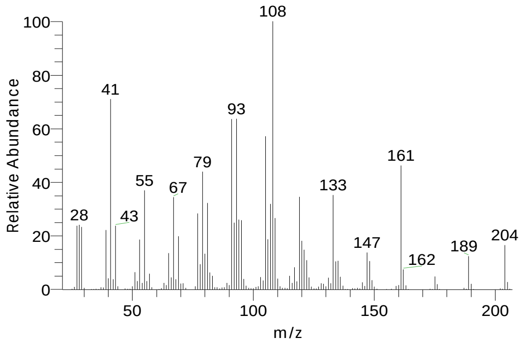

(+)-SATIVEN
Formula C15H24, MW 204, CAS# 3650-28-0, Entry# 392536

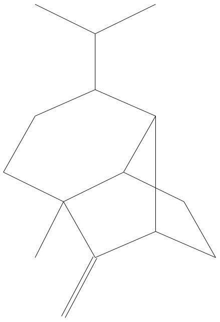

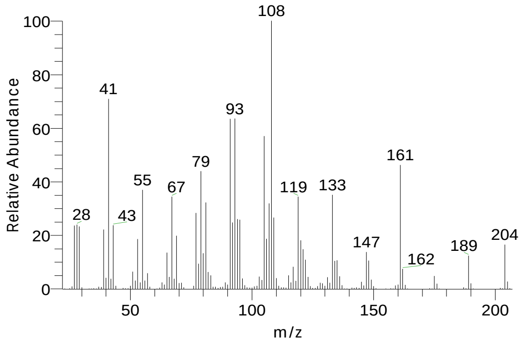

Formula C15H24, MW 204, CAS# 3650-28-0, Entry# 89185
(+)-SATIVEN

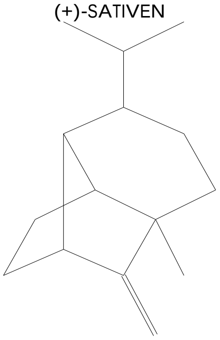

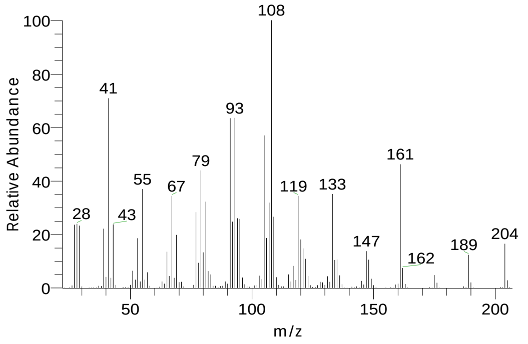

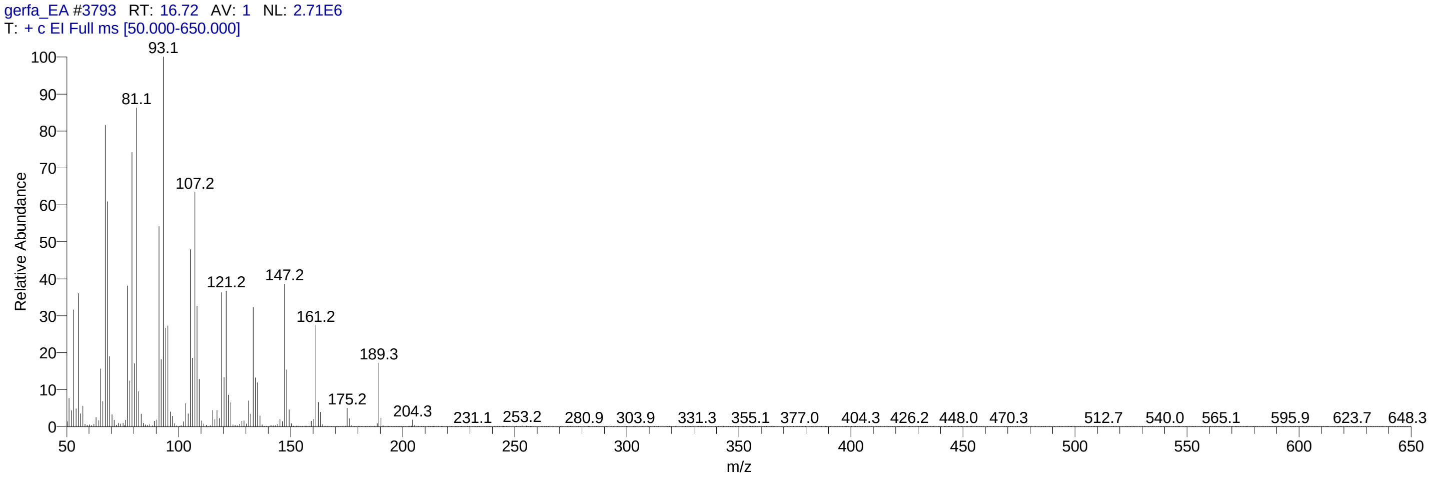

RT	Compound Name	Area %	Molecular Formula	Molecular Weight	Cas #	MF	Library
16.72	CYCLOHEXANE, 1-ETHENYL-1-METHYL-2,4-BIS(1-METHYLETHENYL)-, [1S-(1à,2á,4á)]-	0.16	C15H24	204	515-13-9	949	WileyRegistry8e

My GC-MS Report

RT	Compound Name	Area %	Molecular Formula	Molecular Weight	Cas #	MF	Library
16.72	CYCLOHEXANE, 1-ETHENYL-1-METHYL-2,4-BIS(1-METHYLETHENYL)-, [1S-(1à,2á,4á)]-	0.16	C15H24	204	515-13-9	936	WileyRegistry8e
16.72	Cyclohexane, 1-ethenyl-1-methyl-2,4-bis(1-methylethenyl)-, [1S-(1à,2á,4á)]-	0.16	C15H24	204	515-13-9	914	replib

Compound Structure	Hit Spectrum

CYCLOHEXANE, 1-ETHENYL-1-METHYL-2,4-BIS(1-METHYLETHENYL)-, [1S-(1à,2á,4á)]-
Formula C15H24, MW 204, CAS# 515-13-9, Entry# 89511
2,4-DIISOPROPENYL-1-METHYL-1-VINYLCYCLOHEXANE #

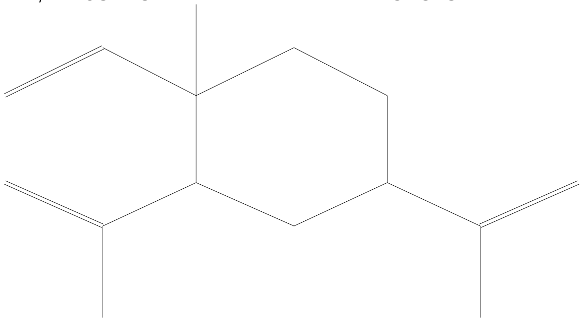

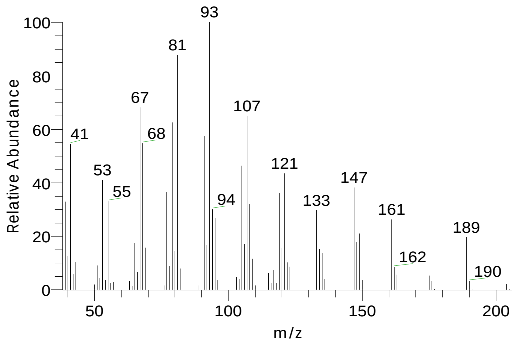

CYCLOHEXANE, 1-ETHENYL-1-METHYL-2,4-BIS(1-METHYLETHENYL)-, [1S-(1à,2á,4á)]-
Formula C15H24, MW 204, CAS# 515-13-9, Entry# 89566
2,4-DIISOPROPENYL-1-METHYL-1-VINYLCYCLOHEXANE #

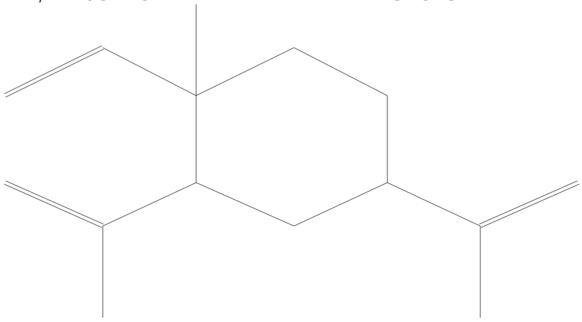

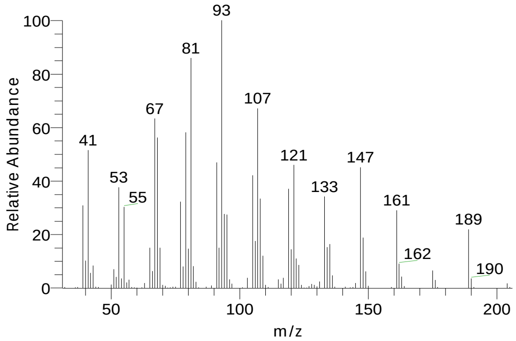

Cyclohexane, 1-ethenyl-1-methyl-2,4-bis(1-methylethenyl)-, [1S-(1à,2á,4á)]-
Formula C15H24, MW 204, CAS# 515-13-9, Entry# 11582
Cyclohexane, 2,4-diisopropenyl-1-methyl-1-vinyl-, (1S,2R,4R)- (-)-

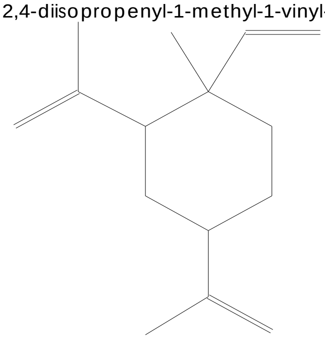

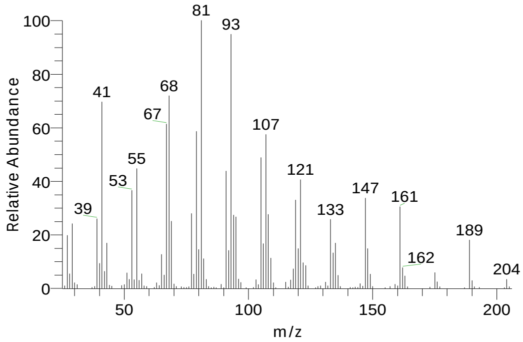

My GC-MS Report

gerfa_EA #3817 RT: 16.80 AV: 1 NL: 4.87E6
T: + c EI Full ms [50.000-650.000]

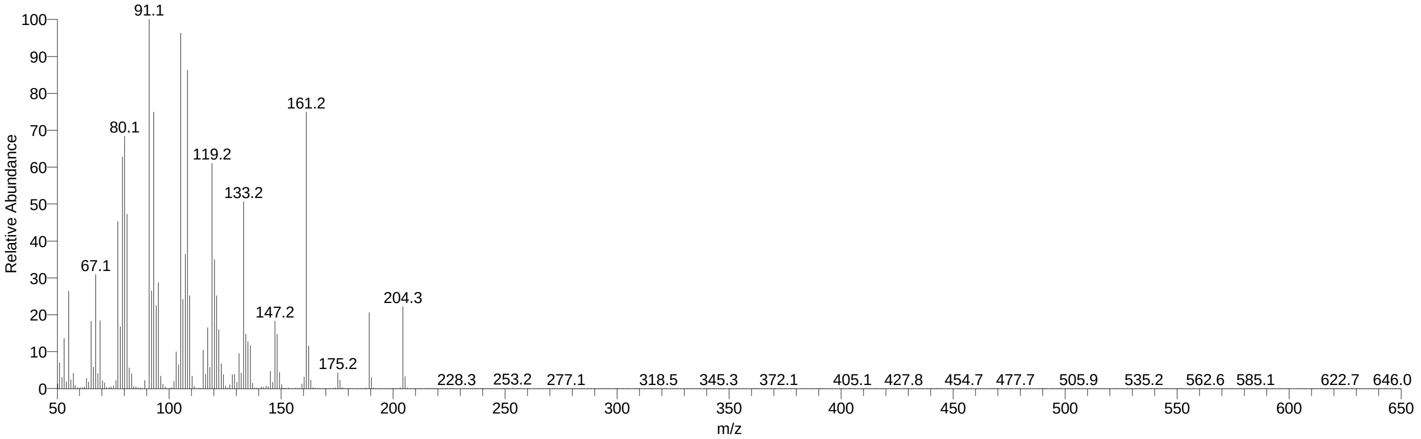

RT	Compound Name	Area %	Molecular Formula	Molecular Weight	Cas #	MF	Library
16.80	1,4-Methano-1H-indene, octahydro-4-methyl-8-methylene-7-(1-methylethyl)-, [1S-(1à,3aá,4à,7à,7aá)]-	0.35	C15H24	204	3650-28-0	908	mainlib
16.80	1,4-METHANO-1H-INDENE, OCTAHYDRO-4-METHYL-8-METHYLENE-7-(1-METHYLETHYL)-, [1S-(1à,3Aá,4à,7à,7Aá)]-	0.35	C15H24	204	3650-28-0	908	WileyRegistry8e
16.80	(+)-SATIVEN	0.35	C15H24	204	3650-28-0	908	WileyRegistry8e

Compound Structure | Hit Spectrum

Formula C15H24, MW 204, CAS# 3650-28-0, Entry# 84625
(+)-Sativen

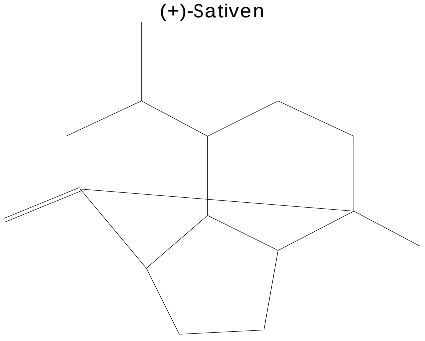

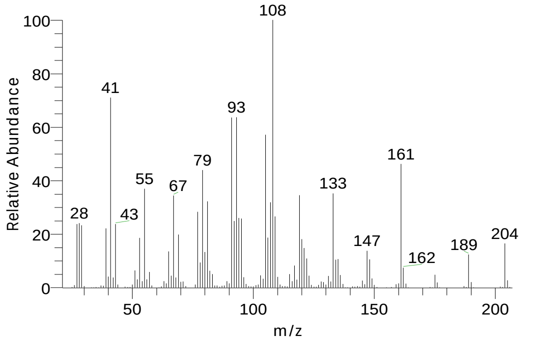

Formula C15H24, MW 204, CAS# 3650-28-0, Entry# 89185
(+)-SATIVEN

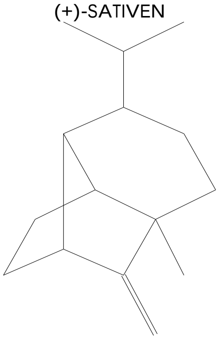

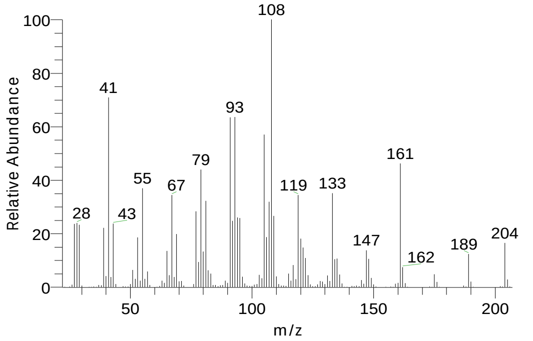

My GC-MS Report

Compound Structure | Hit Spectrum

(+)-SATIVEN
Formula C15H24, MW 204, CAS# 3650-28-0, Entry# 392536

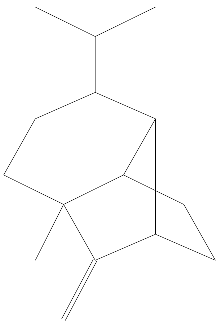

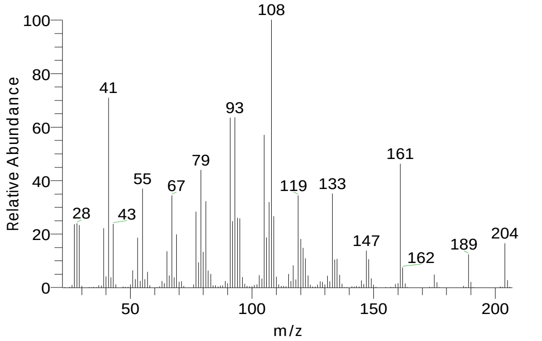

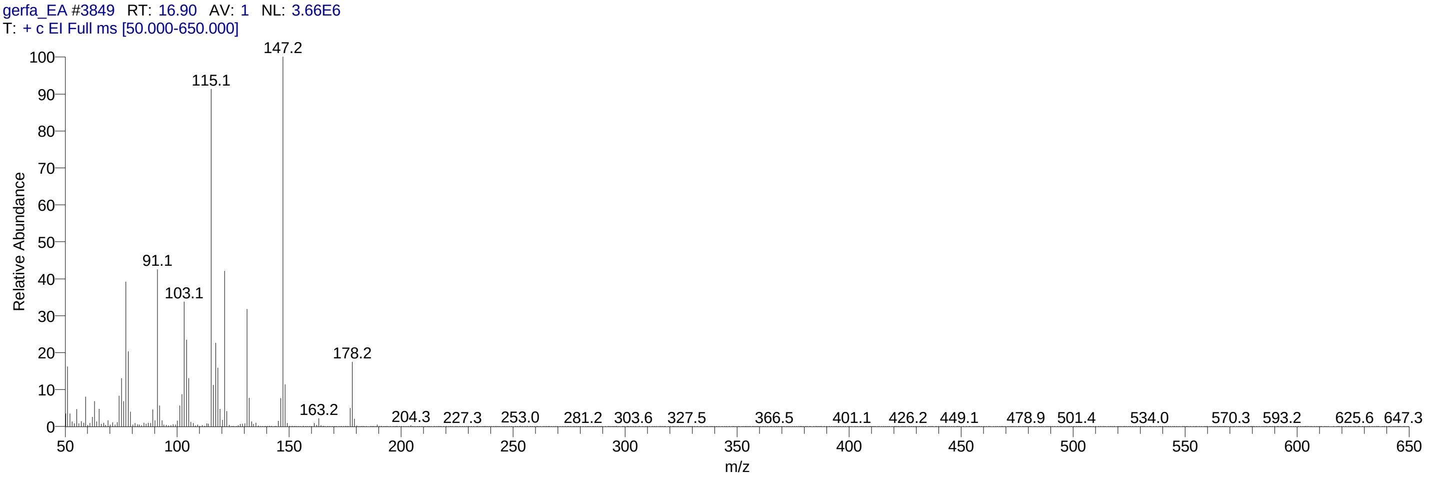

RT	Compound Name	Area %	Molecular Formula	Molecular Weight	Cas #	MF	Library
16.90	CINNAMALDEHYDE DIMETHYL ACETAL	0.11	C11H14O2	178	NA	921	WileyRegistry8e
16.90	9-Methoxybicyclo[6.1.0]nona-2,4,6-triene	0.11	C10H12O	148	826-14-2	774	mainlib
16.90	9-METHOXYBICYCLO[6.1.0]NONA-2,4,6-TRIENE	0.11	C10H12O	148	NA	774	WileyRegistry8e

Compound Structure | Hit Spectrum

CINNAMALDEHYDE DIMETHYL ACETAL
Formula C11H14O2, MW 178, CAS# NA, Entry# 60971

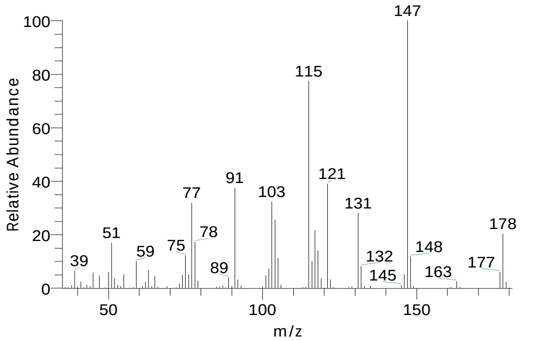

My GC-MS Report

Compound Structure | Hit Spectrum

9-Methoxybicyclo[6.1.0]nona-2,4,6-triene
Formula C10H12O, MW 148, CAS# 826-14-2, Entry# 94587
Bicyclo[6.1.0]nona-2,4,6-trien-9-yl methyl ether #

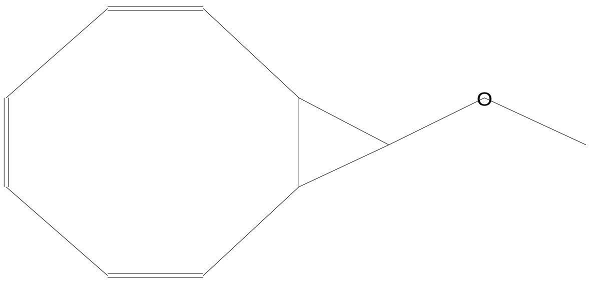

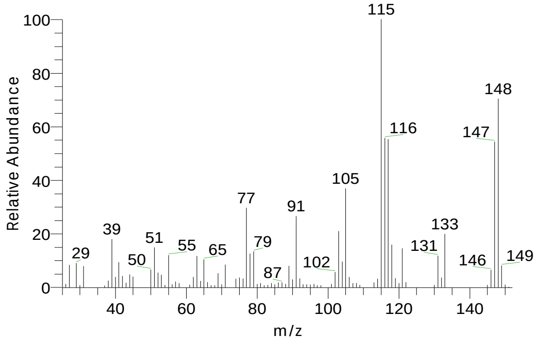

9-METHOXYBICYCLO[6.1.0]NONA-2,4,6-TRIENE
Formula C10H12O, MW 148, CAS# NA, Entry# 370644
9-METHOXY-BICYCLO[6.1.0]NONA-2,4,6-TRIENE

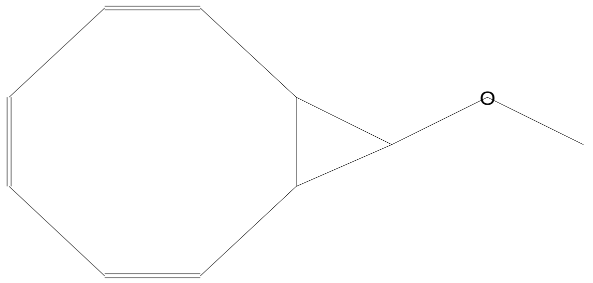

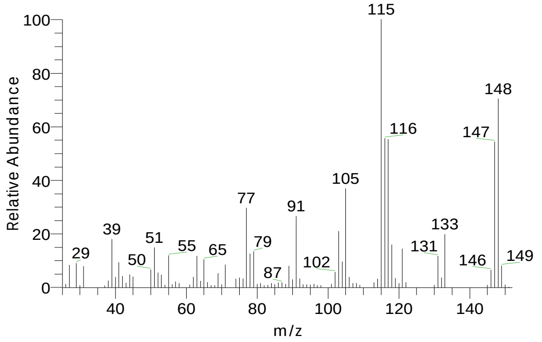

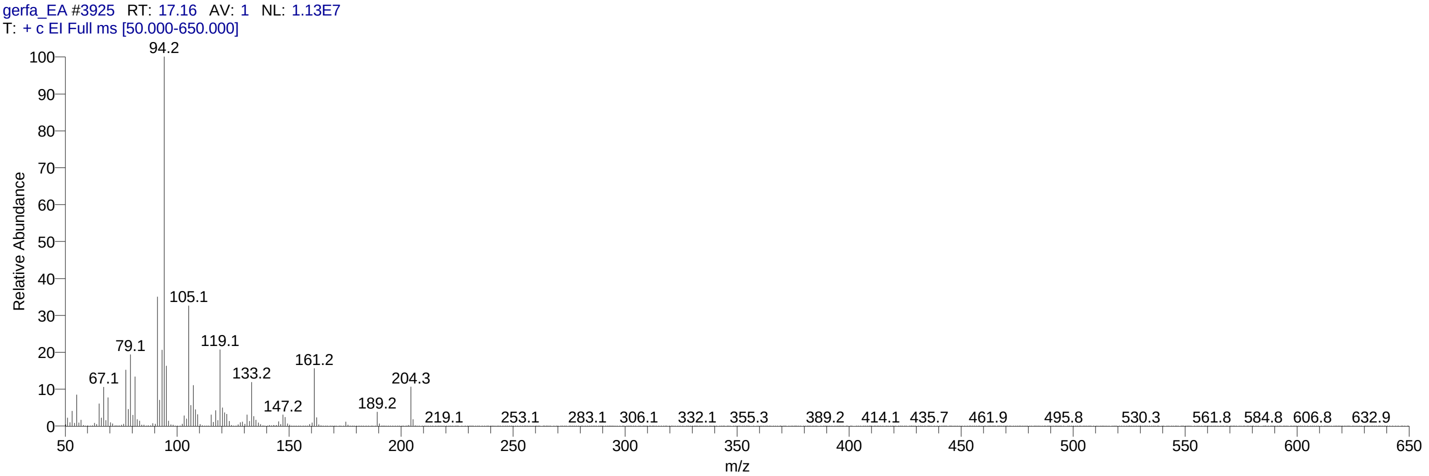

RT	Compound Name	Area %	Molecular Formula	Molecular Weight	Cas #	MF	Library
17.16	Isosativene	0.28	C15H24	204	24959-83-9	958	replib
17.16	(-)-ISOSATIVENE	0.28	C15H24	204	24959-83-9	929	WileyRegistry8e
17.16	Isosativene	0.28	C15H24	204	24959-83-9	923	replib

My GC-MS Report

Compound Structure | Hit Spectrum

Isosativene
Formula C15H24, MW 204, CAS# 24959-83-9, Entry# 14356

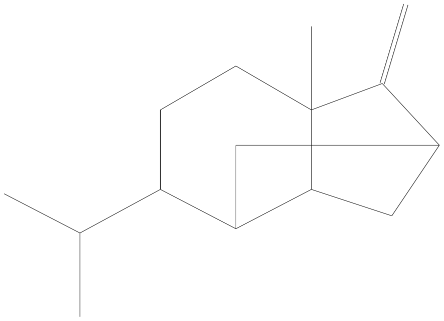

SI 938, RSI 958, replib, Entry# 14356, CAS# 24959-83-9, Isosativene

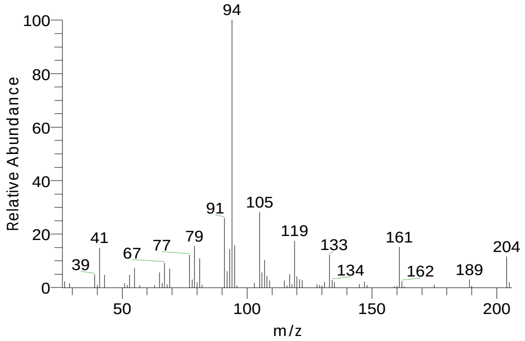

(-)-ISOSATIVENE
Formula C15H24, MW 204, CAS# 24959-83-9, Entry# 89650

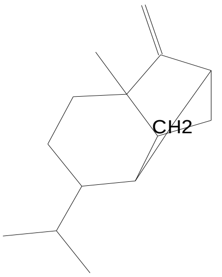

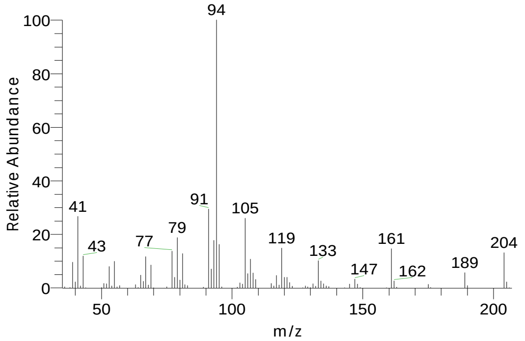

Isosativene
Formula C15H24, MW 204, CAS# 24959-83-9, Entry# 14330

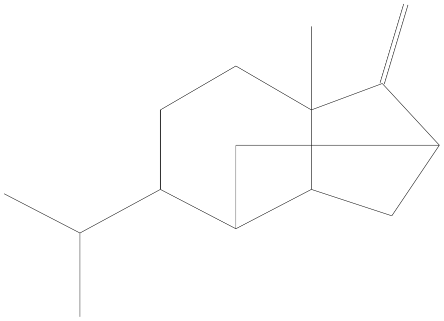

SI 917, RSI 923, replib, Entry# 14330, CAS# 24959-83-9, Isosativene

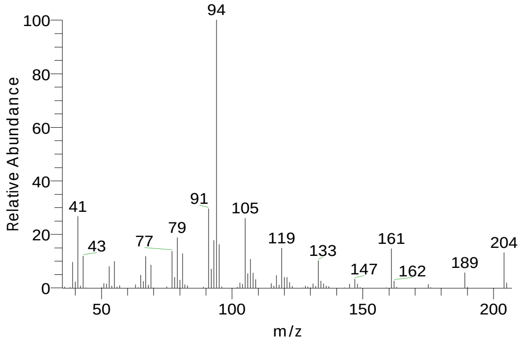

gerfa_EA #3994 RT: 17.39 AV: 1 NL: 5.00E6
T: + c EI Full ms [50.000-650.000]

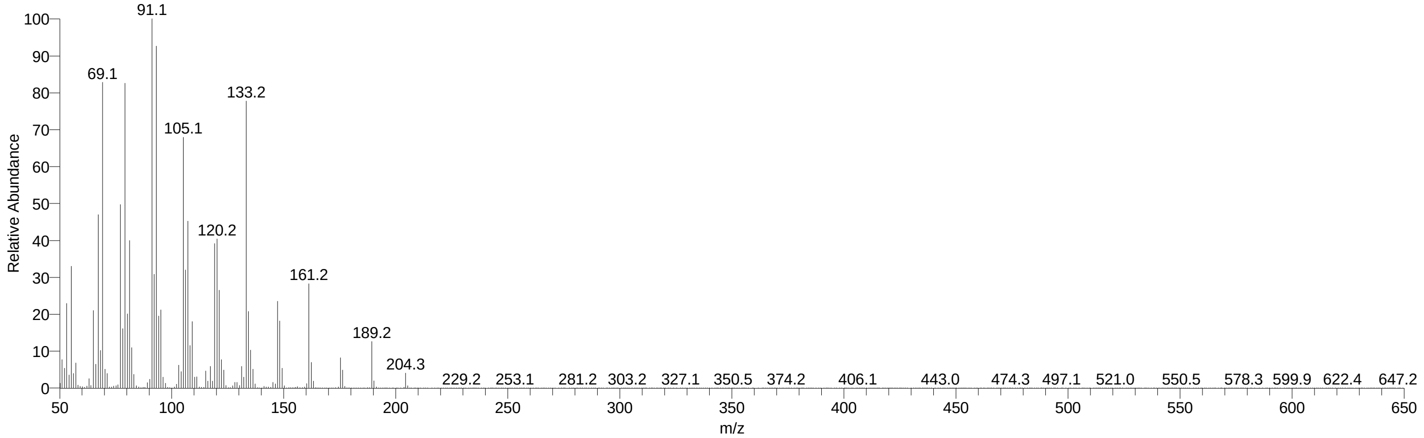

RT	Compound Name	Area %	Molecular Formula	Molecular Weight	Cas #	MF	Library
17.39	BICYCLO[7.2.0]UNDEC-4-ENE, 4,11,11-TRIMETHYL-8-METHYLENE-, [1R-(1R*,4E,9S*)]-	0.38	C15H24	204	87-44-5	951	WileyRegistry8e

My GC-MS Report

RT	Compound Name	Area %	Molecular Formula	Molecular Weight	Cas #	MF	Library
17.39	BICYCLO[7.2.0]UNDEC-4-ENE, 4,11,11-TRIMETHYL-8-METHYLENE-, [1R-(1R*,4E,9S*)]-	0.38	C15H24	204	87-44-5	948	WileyRegistry8e
17.39	Caryophyllene	0.38	C15H24	204	87-44-5	934	mainlib

Compound Structure | Hit Spectrum

BICYCLO[7.2.0]UNDEC-4-ENE, 4,11,11-TRIMETHYL-8-METHYLENE-, [1R-(1R*,4E,9S*)]-
Formula C15H24, MW 204, CAS# 87-44-5, Entry# 89162
2,6,10,10-TETRAMETHYLBICYCLO[7.2.0]UNDECA-1,6-DIENE #

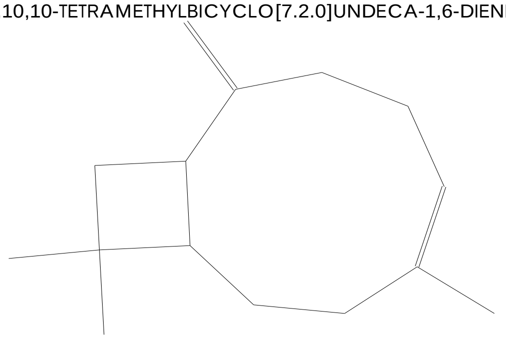

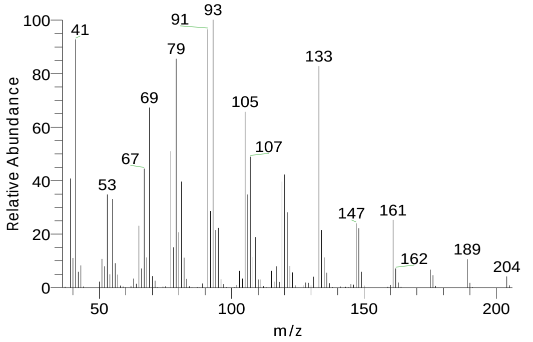

BICYCLO[7.2.0]UNDEC-4-ENE, 4,11,11-TRIMETHYL-8-METHYLENE-, [1R-(1R*,4E,9S*)]-
Formula C15H24, MW 204, CAS# 87-44-5, Entry# 89170
2,6,10,10-TETRAMETHYLBICYCLO[7.2.0]UNDECA-1,6-DIENE #

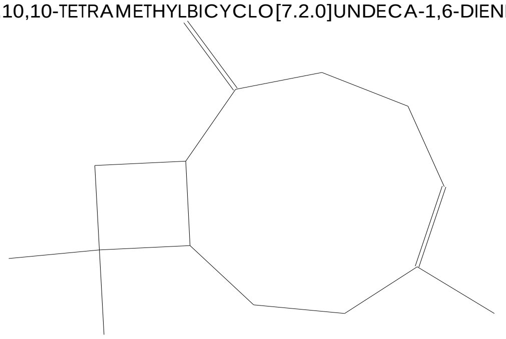

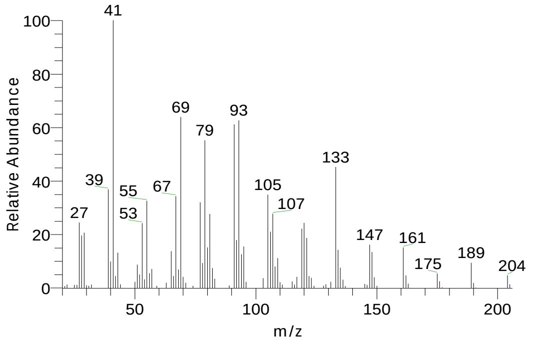

SI 932, RSI 934, mainlib, Entry# 66572, CAS# 87-44-5, Caryophyllene

Caryophyllene
Formula C15H24, MW 204, CAS# 87-44-5, Entry# 66572
Bicyclo[7.2.0]undec-4-ene, 4,11,11-trimethyl-8-methylene-, [1R-(1R*,4E,9S*)]-

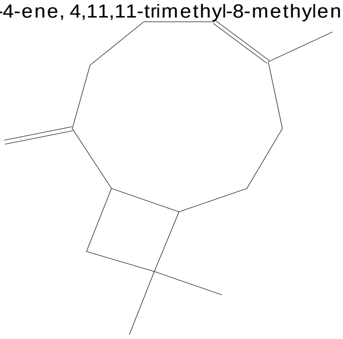

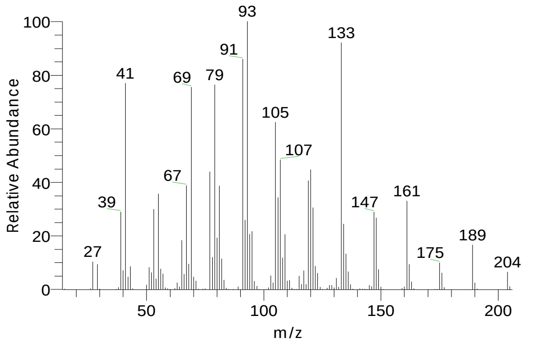

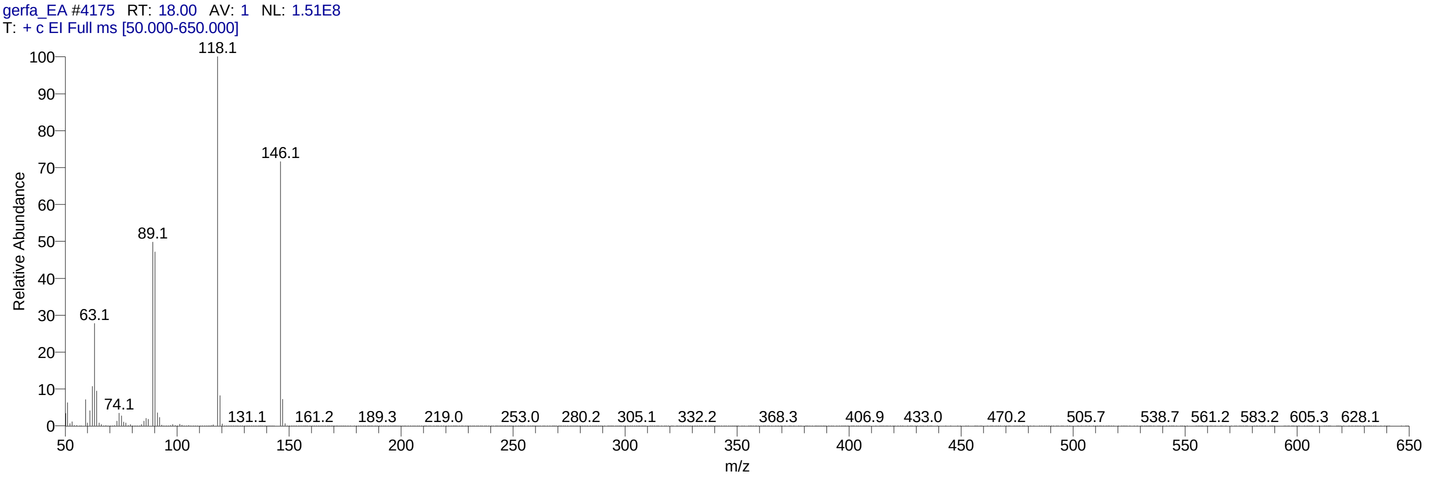

My GC-MS Report

RT	Compound Name	Area %	Molecular Formula	Molecular Weight	Cas #	MF	Library
18.00	2H-1-BENZOPYRAN-2-ONE	9.92	C9H6O2	146	91-64-5	972	WileyRegistry8e
18.00	Coumarin	9.92	C9H6O2	146	91-64-5	972	replib
18.00	Coumarin	9.92	C9H6O2	146	91-64-5	946	replib

Compound Structure | Hit Spectrum

2H-1-BENZOPYRAN-2-ONE
Formula C9H6O2, MW 146, CAS# 91-64-5, Entry# 30896
CHROMEN-2-ONE

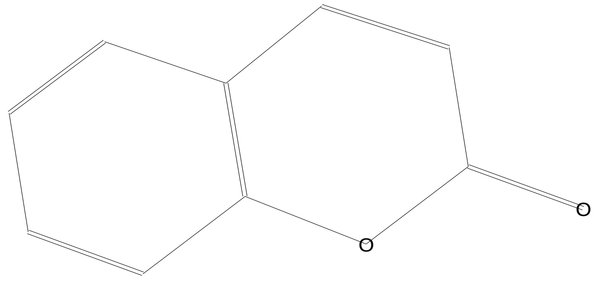

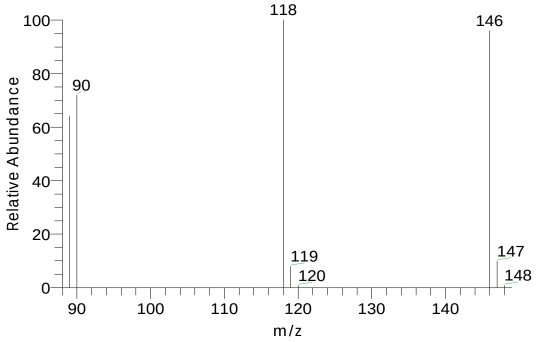

SI 960, RSI 972, replib, Entry# 18483, CAS# 91-64-5, Coumarin

Coumarin
Formula C9H6O2, MW 146, CAS# 91-64-5, Entry# 18483
2H-1-Benzopyran-2-one

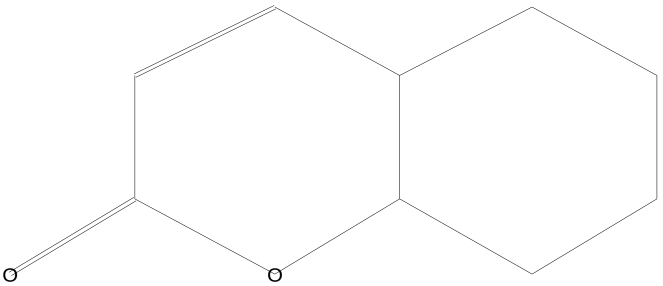

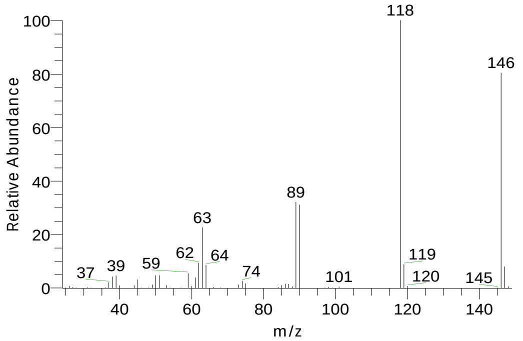

SI 946, RSI 946, replib, Entry# 18488, CAS# 91-64-5, Coumarin

Coumarin
Formula C9H6O2, MW 146, CAS# 91-64-5, Entry# 18488
2H-1-Benzopyran-2-one

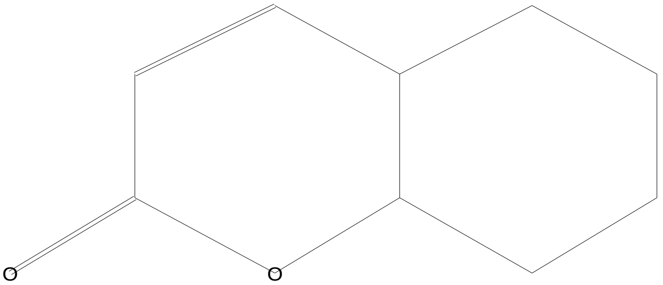

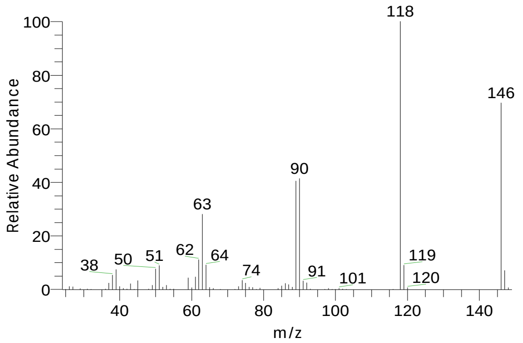

gerfa_EA #4223 RT: 18.16 AV: 1 NL: 1.10E8
T: + c EI Full ms [50.000-650.000]

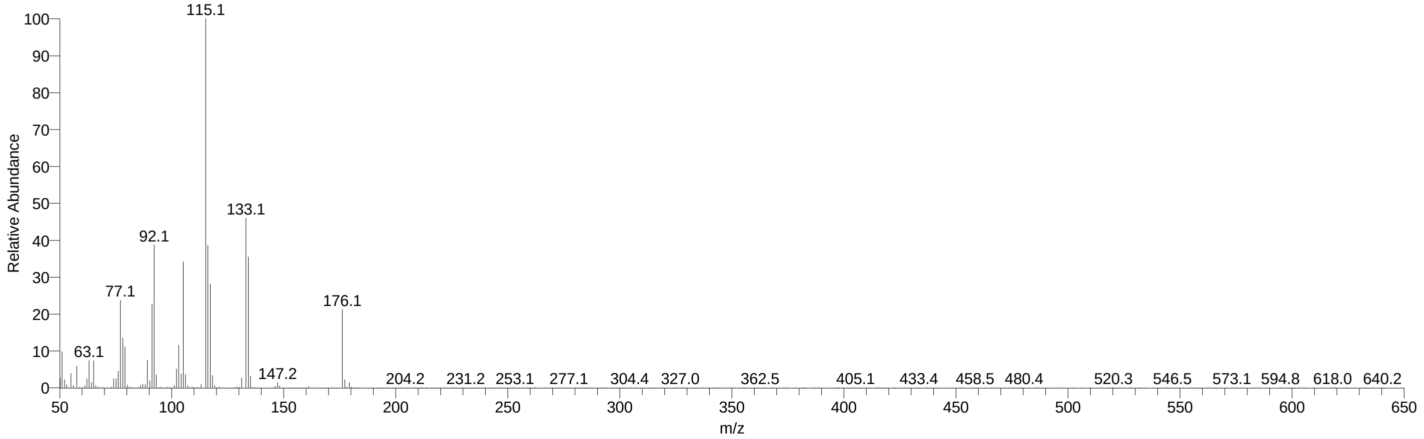

My GC-MS Report

RT	Compound Name	Area %	Molecular Formula	Molecular Weight	Cas #	MF	Library
18.16	Acetic acid, cinnamyl ester	4.83	C11H12O2	176	103-54-8	955	mainlib
18.16	2-PROPEN-1-OL, 3-PHENYL-, ACETATE	4.83	C11H12O2	176	103-54-8	943	WileyRegistry8e
18.16	CINNAMYL ALCOHOL, ACETATE, (E)-	4.83	C11H12O2	176	21040-45-9	968	WileyRegistry8e

Compound Structure | Hit Spectrum

Acetic acid, cinnamyl ester
Formula C11H12O2, MW 176, CAS# 103-54-8, Entry# 11373
2-Propen-1-ol, 3-phenyl-, acetate

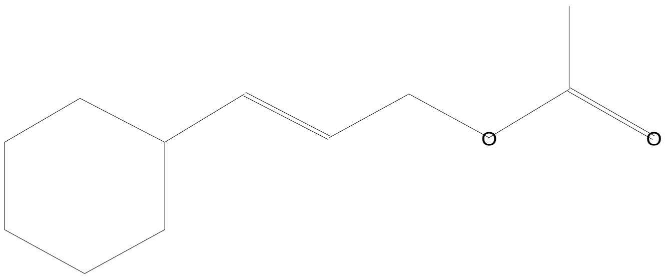

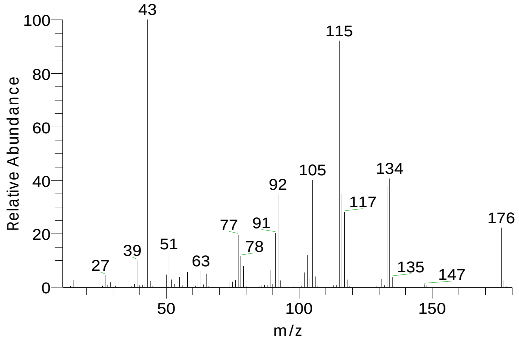

2-PROPEN-1-OL, 3-PHENYL-, ACETATE
Formula C11H12O2, MW 176, CAS# 103-54-8, Entry# 58949
ACETIC ACID CINNAMYL ESTER

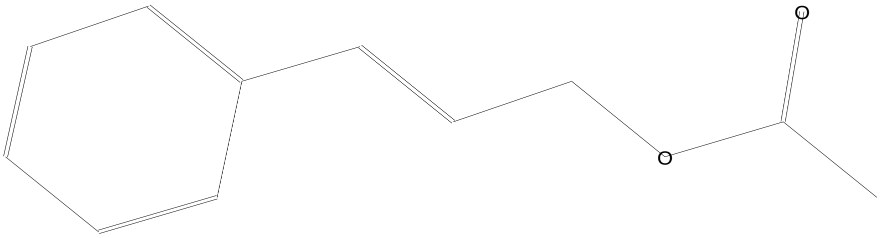

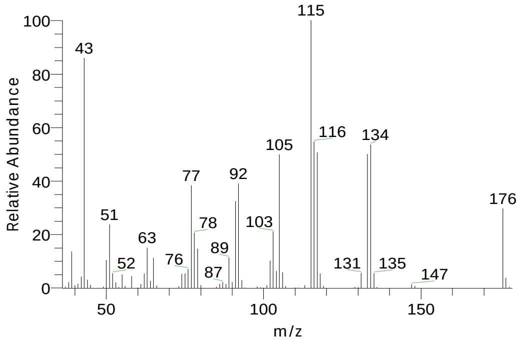

CINNAMYL ALCOHOL, ACETATE, (E)-
Formula C11H12O2, MW 176, CAS# 21040-45-9, Entry# 59025
(2E)-3-PHENYL-2-PROPENYL ACETATE #

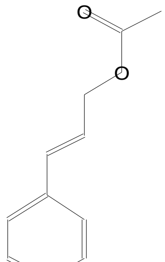

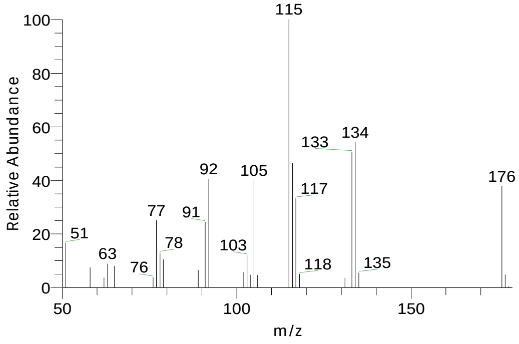

My GC-MS Report

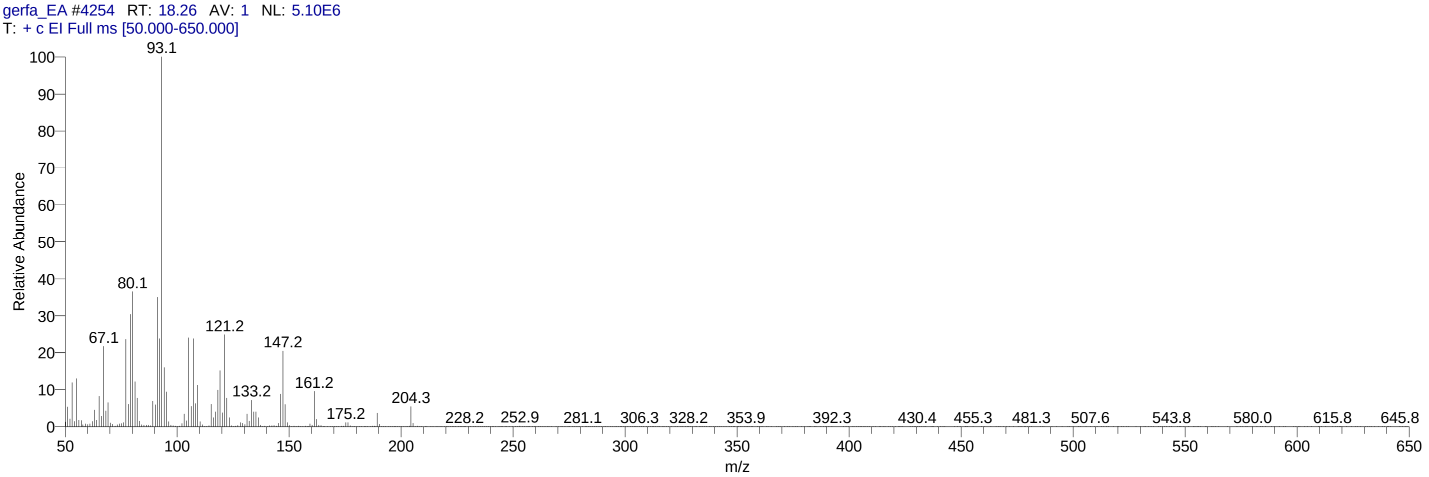

RT	Compound Name	Area %	Molecular Formula	Molecular Weight	Cas #	MF	Library
18.26	Humulene	0.11	C15H24	204	6753-98-6	907	replib
18.26	Humulene	0.11	C15H24	204	6753-98-6	895	replib
18.26	1,4,8-CYCLOUNDECATRIENE, 2,6,6,9-TETRAMETHYL-, (E,E,E)-	0.11	C15H24	204	6753-98-6	871	WileyRegistry8e

Compound Structure	Hit Spectrum

Humulene
Formula C15H24, MW 204, CAS# 6753-98-6, Entry# 14003
à-Caryophyllene

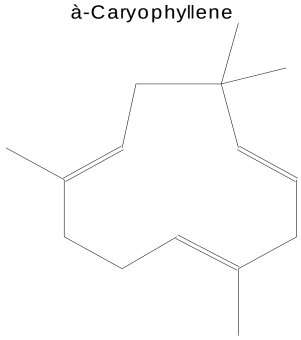

SI 887, RSI 907, replib, Entry# 14003, CAS# 6753-98-6, Humulene

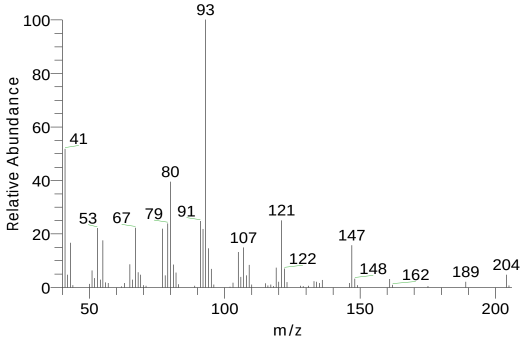

Humulene
Formula C15H24, MW 204, CAS# 6753-98-6, Entry# 14104
à-Caryophyllene

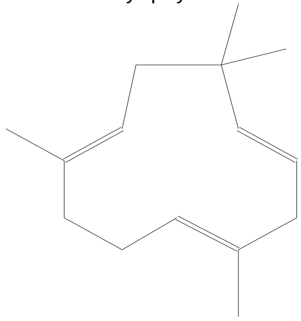

SI 861, RSI 895, replib, Entry# 14104, CAS# 6753-98-6, Humulene

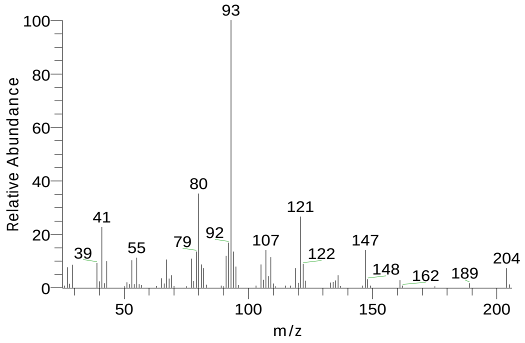

1,4,8-CYCLOUNDECATRIENE, 2,6,6,9-TETRAMETHYL-, (E,E,E)-
Formula C15H24, MW 204, CAS# 6753-98-6, Entry# 89108
2,6,6,9-TETRAMETHYL-1,4,8-CYCLOUNDECATRIENE #

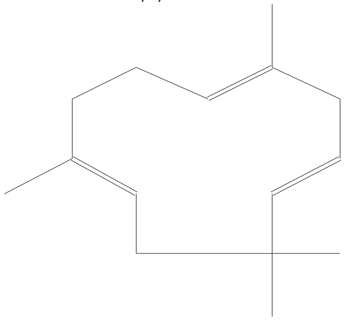

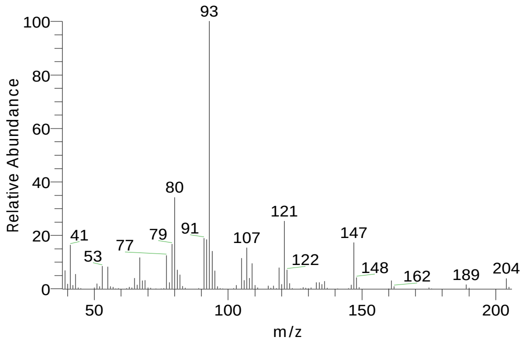

My GC-MS Report

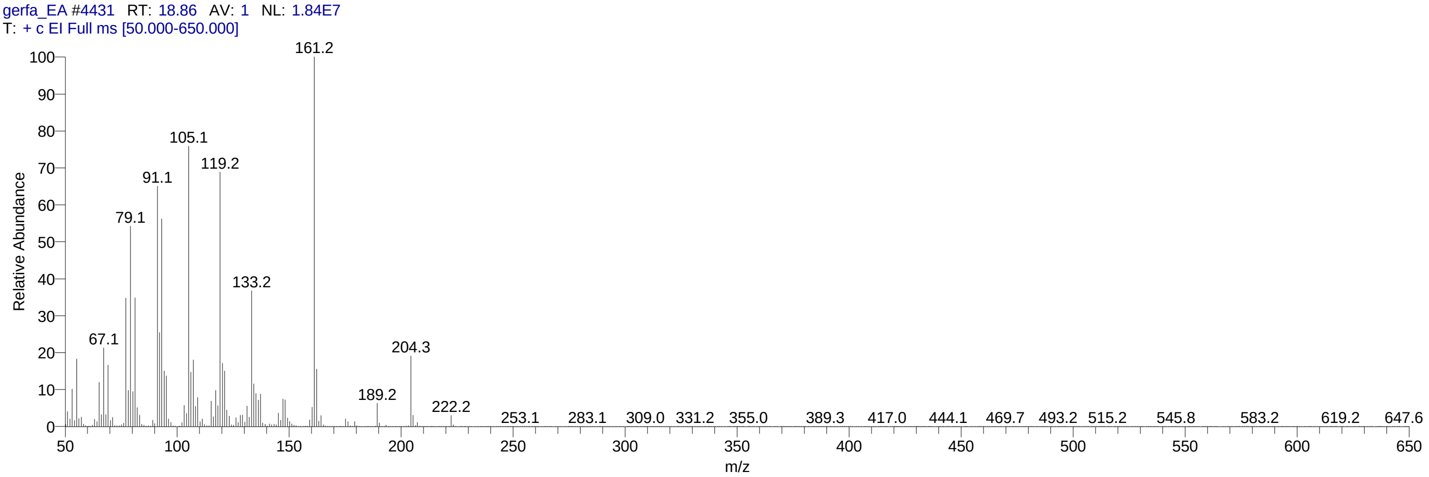

RT	Compound Name	Area %	Molecular Formula	Molecular Weight	Cas #	MF	Library
18.86	ç-Muurolene	1.05	C15H24	204	30021-74-0	938	replib
18.86	Naphthalene, 1,2,4a,5,6,8a-hexahydro-4,7-dimethyl-1-(1-methylethyl)-	1.05	C15H24	204	483-75-0	954	replib
18.86	ç-Muurolene	1.05	C15H24	204	30021-74-0	929	replib

Compound Structure

ç-Muurolene
Formula C15H24, MW 204, CAS# 30021-74-0, Entry# 24906

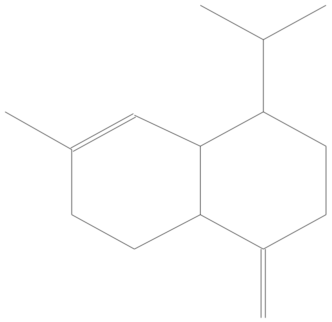

Naphthalene, 1,2,4a,5,6,8a-hexahydro-4,7-dimethyl-1-(1-methylethyl)-
Formula C15H24, MW 204, CAS# 483-75-0, Entry# 24889
1-Isopropyl-4,7-dimethyl-1,2,4a,5,6,8a-hexahydronaphthalene #

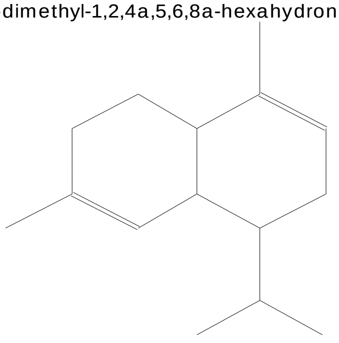

Hit Spectrum

SI 921, RSI 938, replib, Entry# 24906, CAS# 30021-74-0, ç-Muurolene

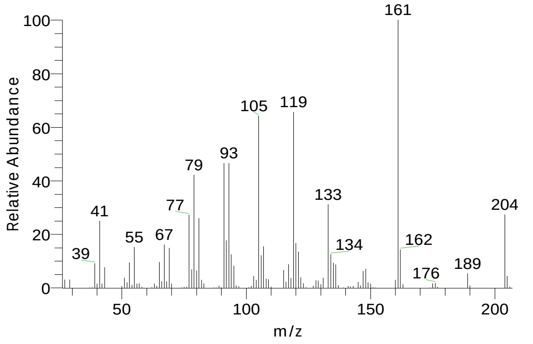

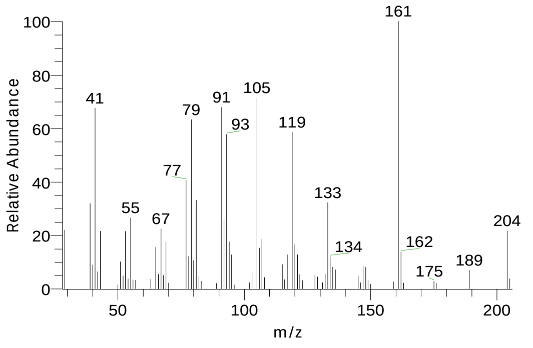

My GC-MS Report

Compound Structure	Hit Spectrum

ç-Muurolene
Formula C15H24, MW 204, CAS# 30021-74-0, Entry# 24843

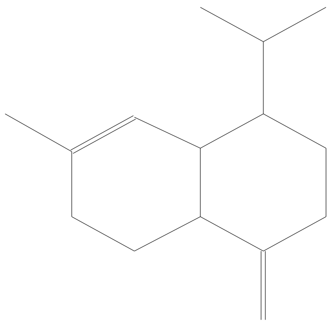

SI 909, RSI 929, replib, Entry# 24843, CAS# 30021-74-0, ç-Muurolene

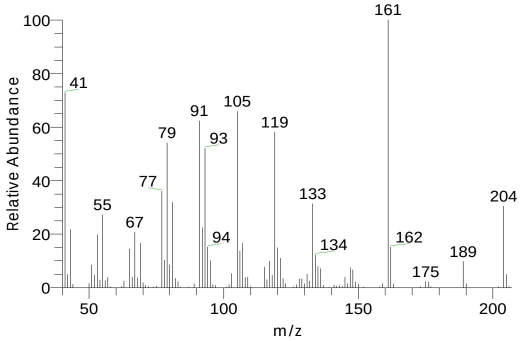

gerfa_EA #4564 RT: 19.30 AV: 1 NL: 3.51E6
T: + c EI Full ms [50.000-650.000]

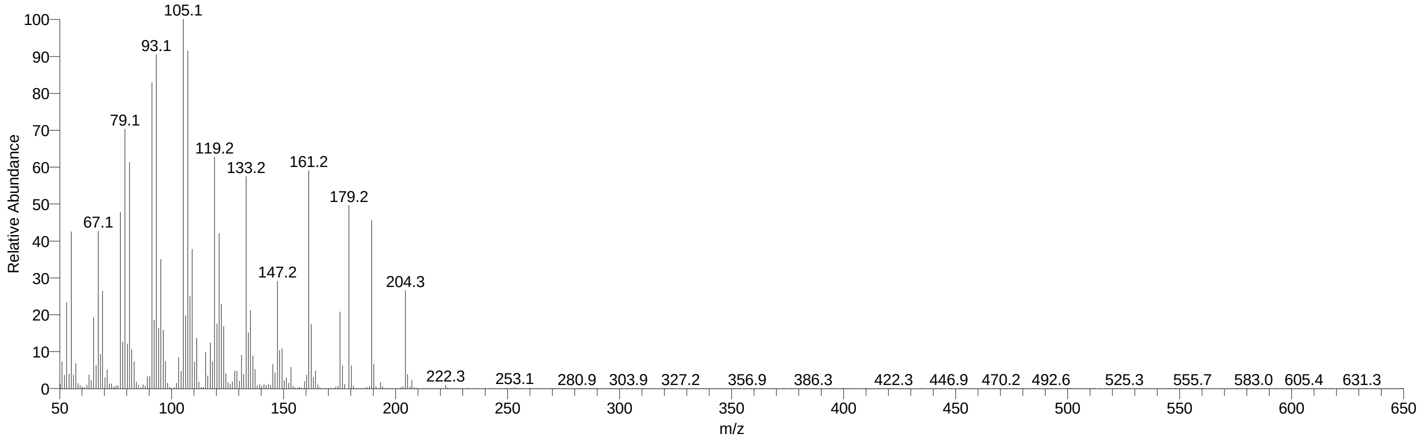

RT	Compound Name	Area %	Molecular Formula	Molecular Weight	Cas #	MF	Library
19.30	Azulene, 1,2,3,3a,4,5,6,7-octahydro-1,4-dimethyl-7-(1-methylethenyl)-, [1R-(1à,3aá,4à,7á)]-	0.26	C15H24	204	22567-17-5	918	replib
19.30	Aromandendrene	0.26	C15H24	204	489-39-4	909	replib
19.30	Naphthalene, 1,2,3,5,6,7,8,8a-octahydro-1,8a-dimethyl-7-(1-methylethenyl)-, [1S-(1à,7à,8aà)]-	0.26	C15H24	204	10219-75-7	898	mainlib

Compound Structure	Hit Spectrum

Azulene, 1,2,3,3a,4,5,6,7-octahydro-1,4-dimethyl-7-(1-methylethenyl)-, [1R-(1à,3aá,4à,7á)]-
Formula C15H24, MW 204, CAS# 22567-17-5, Entry# 1321
1á,4áH,10áH-Guaia-5,11-diene

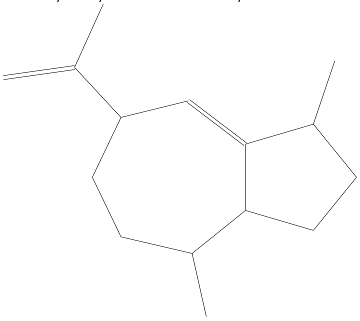

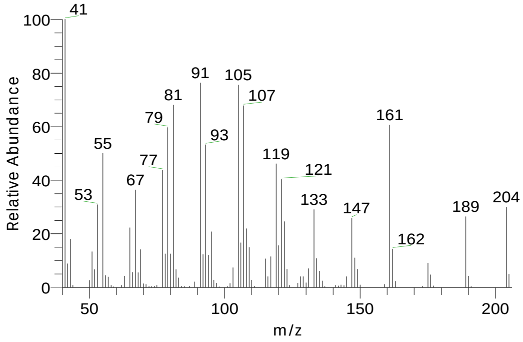

My GC-MS Report

Compound Structure | Hit Spectrum

Aromandendrene
Formula C15H24, MW 204, CAS# 489-39-4, Entry# 13310

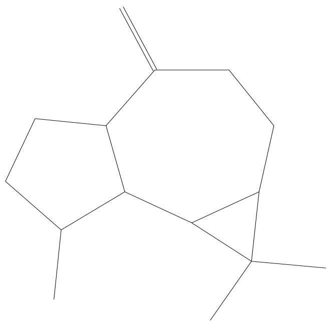

SI 860, RSI 909, replib, Entry# 13310, CAS# 489-39-4, Aromandendrene

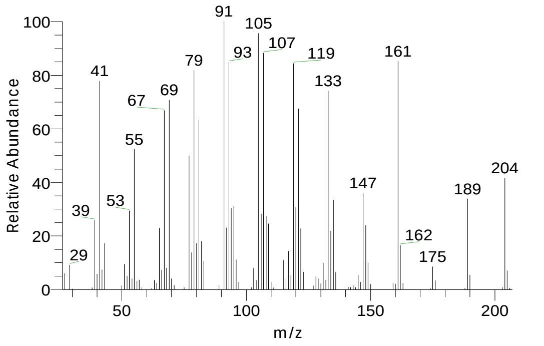

Formula C15H24, MW 204, CAS# 10219-75-7, Entry# 83645
Eremophila-1(10),11-diene

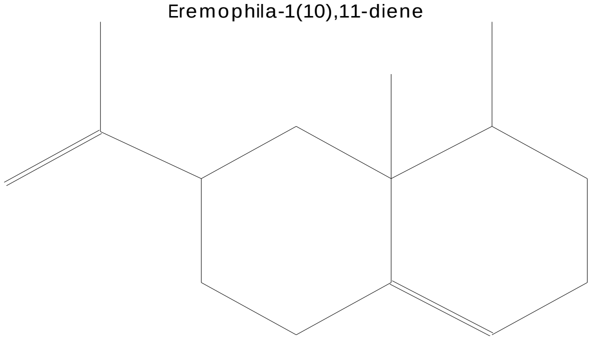

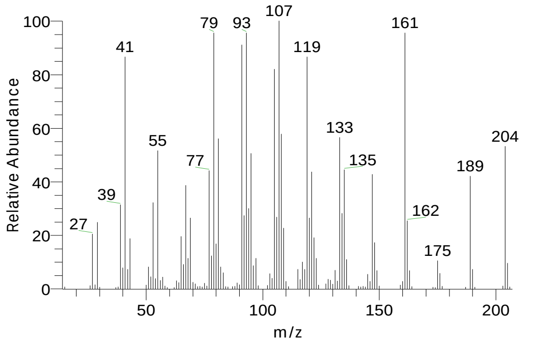

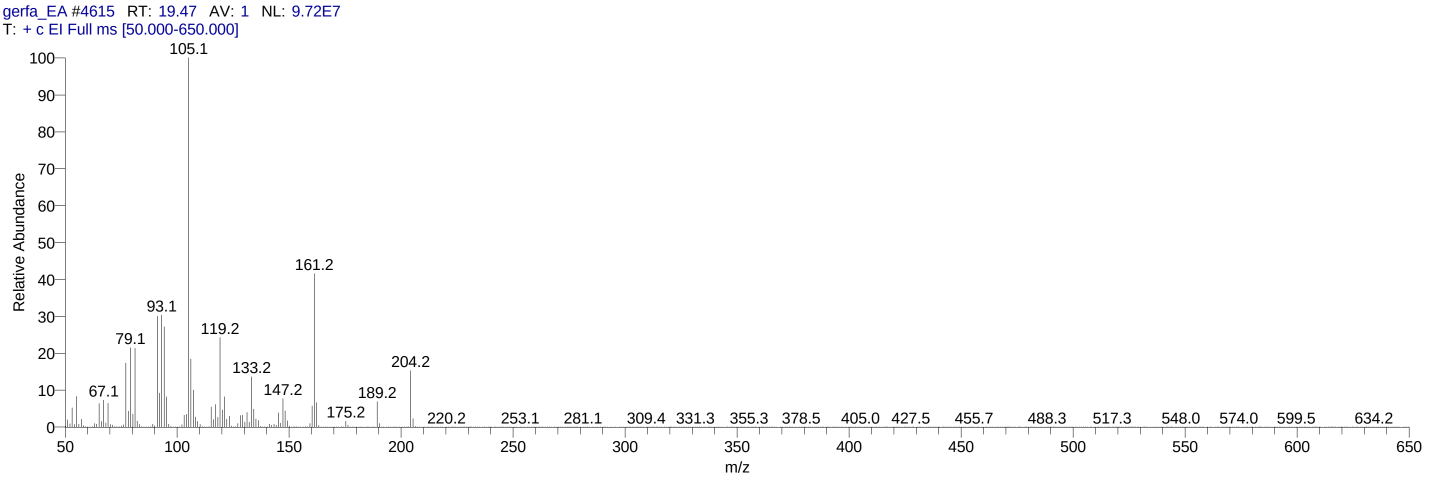

RT	Compound Name	Area %	Molecular Formula	Molecular Weight	Cas #	MF	Library
19.47	à-Muurolene	4.14	C15H24	204	31983-22-9	960	replib
19.47	à-Muurolene	4.14	C15H24	204	10208-80-7	945	replib
19.47	NAPHTHALENE, 1,2,4A,5,6,8A-HEXAHYDRO-4,7-DIMETHYL-1-(1-METHYLETHYL)-, (1à,4Aà,8Aà)-	4.14	C15H24	204	31983-22-9	926	WileyRegistry8e

My GC-MS Report

Compound Structure	Hit Spectrum

à-Muurolene
Formula C15H24, MW 204, CAS# 31983-22-9, Entry# 16421
[1à,4aà,8aà]-1,2,4a,5,6,8a-hexahydro-4-7-dimethyl-1-[1-methylethyl]naphthalene

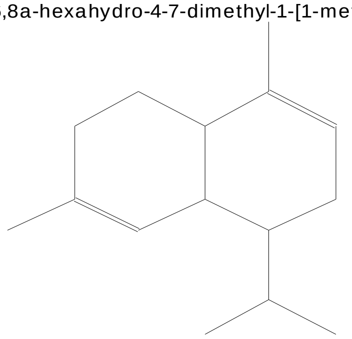

SI 952, RSI 960, replib, Entry# 16421, CAS# 31983-22-9, à-Muurolene

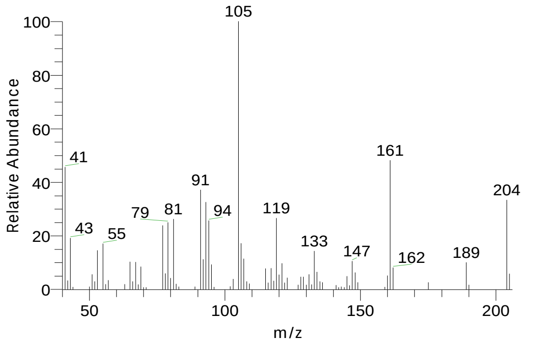

à-Muurolene
Formula C15H24, MW 204, CAS# 10208-80-7, Entry# 16423
Naphthalene, 1,2,4a,5,6,8a-hexahydro-4,7-dimethyl-1-(1-methylethyl)-, (1S,4aS,8aR)-

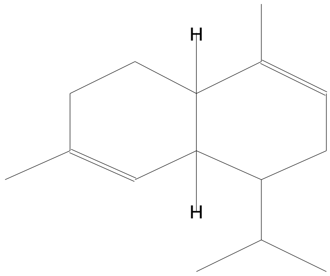

SI 945, RSI 945, replib, Entry# 16423, CAS# 10208-80-7, à-Muurolene

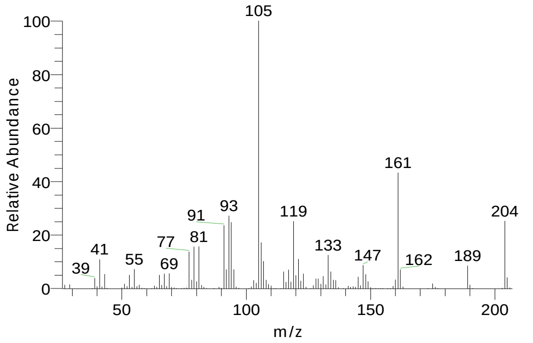

NAPHTHALENE, 1,2,4A,5,6,8A-HEXAHYDRO-4,7-DIMETHYL-1-(1-METHYLETHYL)-, (1à,4Aà,8Aà)-
Formula C15H24, MW 204, CAS# 31983-22-9, Entry# 89350
NAPHTHALENE, 1,2,4Aá,5,6,8Aá-HEXAHYDRO-1á-ISOPROPYL-4,7-DIMETHYL-

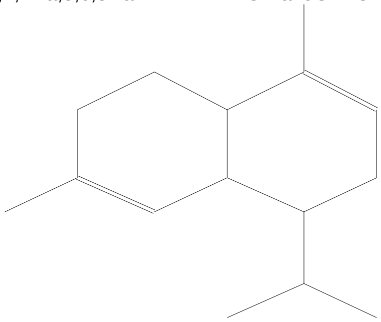

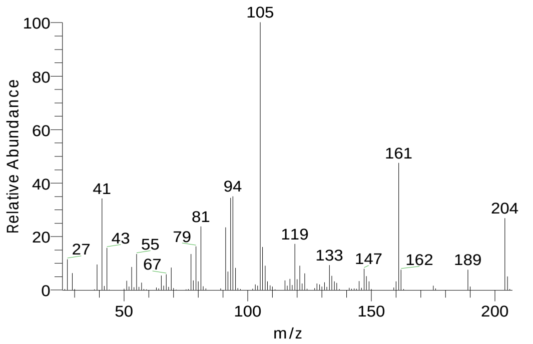

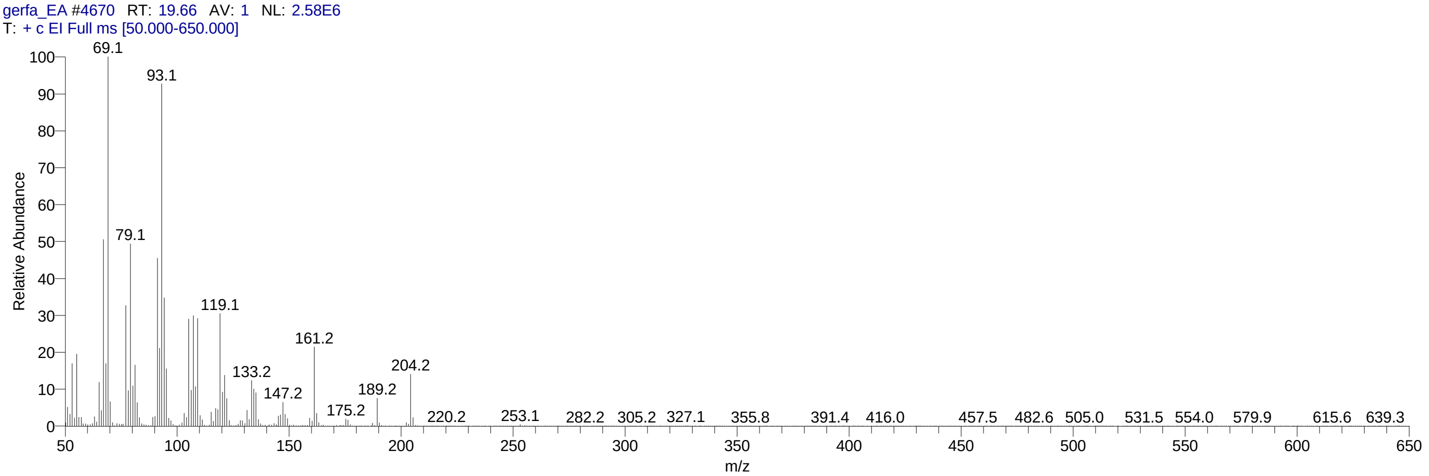

RT	Compound Name	Area %	Molecular Formula	Molecular Weight	Cas #	MF	Library
19.66	CYCLOHEXENE, 1-METHYL-4-(5-METHYL-1-METHYLENE-4-HEXENYL)-, (S)-	0.12	C15H24	204	495-61-4	933	WileyRegistry8e
19.66	á-Bisabolene	0.12	C15H24	204	495-61-4	896	replib

My GC-MS Report

RT	Compound Name	Area %	Molecular Formula	Molecular Weight	Cas #	MF	Library
19.66	1-METHYL-4-(5-METHYL-1-METHYLENE-4-HEXENYL)-1-CYCLOHEXENE	0.12	C15H24	204	NA	896	WileyRegistry8e

Compound Structure

Hit Spectrum

CYCLOHEXENE, 1-METHYL-4-(5-METHYL-1-METHYLENE-4-HEXENYL)-, (S)-
Formula C15H24, MW 204, CAS# 495-61-4, Entry# 89082
1-METHYL-4-(5-METHYL-1-METHYLENE-4-HEXENYL)-1-CYCLOHEXENE #

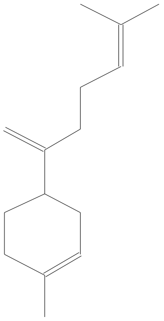

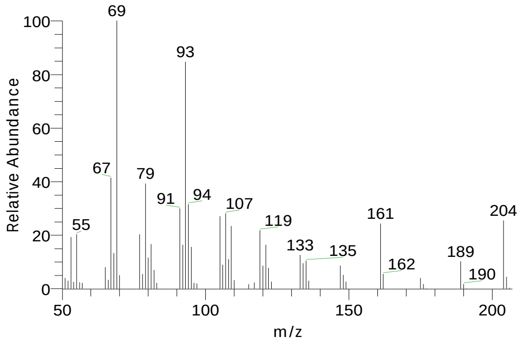

SI 889, RSI 896, replib, Entry# 8536, CAS# 495-61-4, á-Bisabolene

á-Bisabolene
Formula C15H24, MW 204, CAS# 495-61-4, Entry# 8536
Cyclohexene, 1-methyl-4-(5-methyl-1-methylene-4-hexenyl)-, (S)-

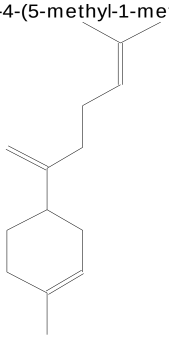

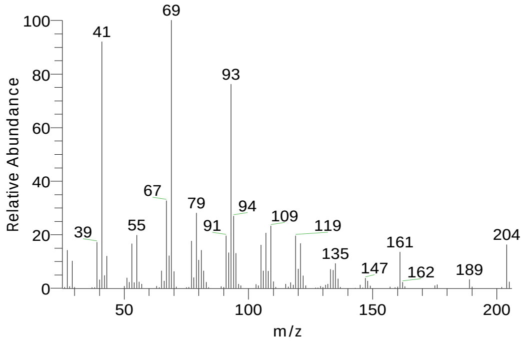

1-METHYL-4-(5-METHYL-1-METHYLENE-4-HEXENYL)-1-CYCLOHEXENE
Formula C15H24, MW 204, CAS# NA, Entry# 388678
BETA-BISABOLEN

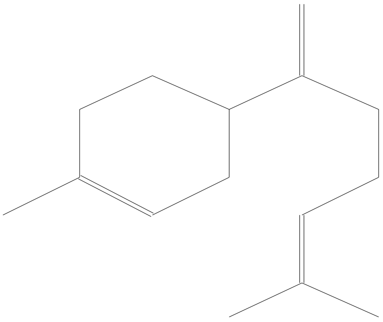

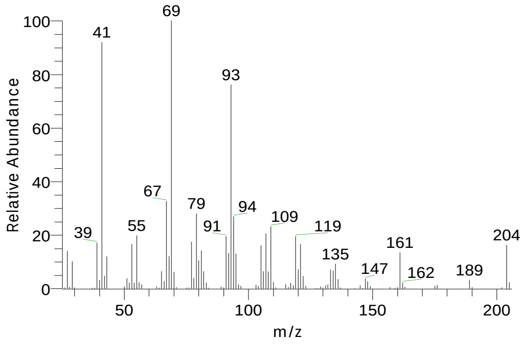

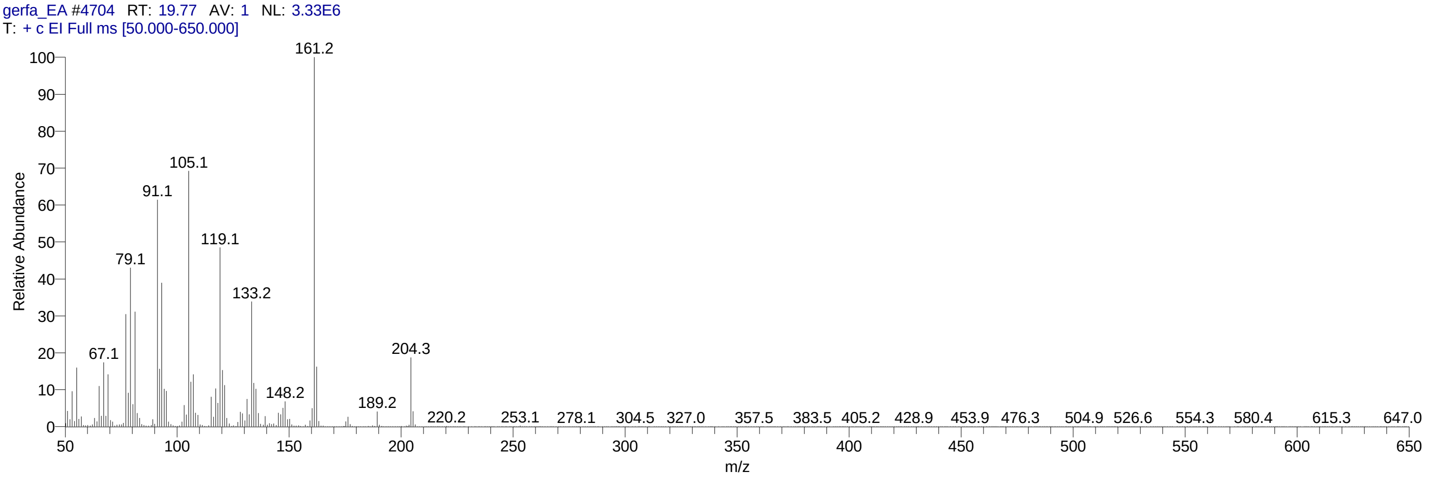

My GC-MS Report

RT	Compound Name	Area %	Molecular Formula	Molecular Weight	Cas #	MF	Library
19.77	Naphthalene, 1,2,3,4,4a,5,6,8a-octahydro-7-methyl-4-methylene-1-(1-methylethyl)-, (1à,4aá,8aà)-	0.18	C15H24	204	39029-41-9	932	replib
19.77	Naphthalene, 1,2,3,4,4a,5,6,8a-octahydro-7-methyl-4-methylene-1-(1-methylethyl)-, (1à,4aá,8aà)-	0.18	C15H24	204	39029-41-9	932	replib
19.77	ç-Muurolene	0.18	C15H24	204	30021-74-0	929	replib

Compound Structure | Hit Spectrum

Formula C15H24, MW 204, CAS# 39029-41-9, Entry# 24895
ç-Cadinene

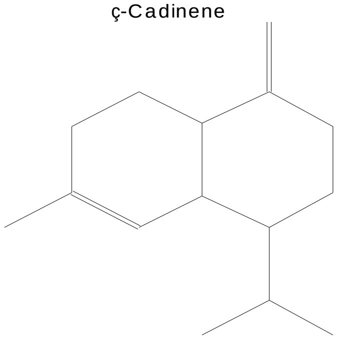

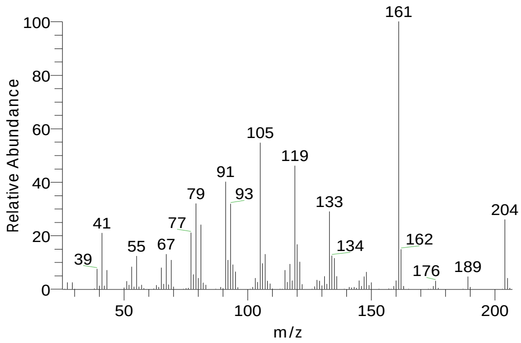

Formula C15H24, MW 204, CAS# 39029-41-9, Entry# 24890
ç-Cadinene

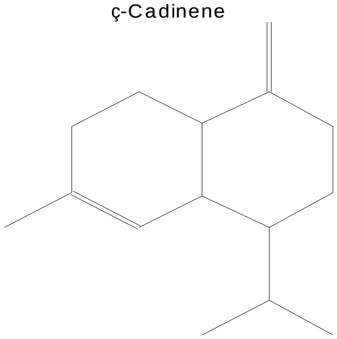

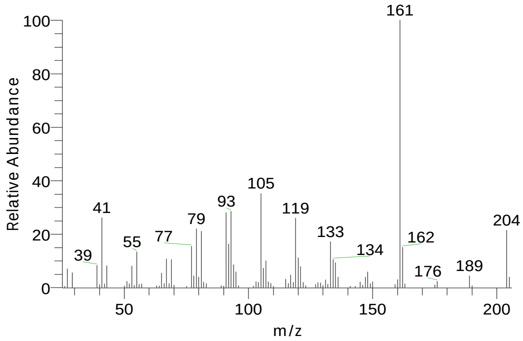

ç-Muurolene
Formula C15H24, MW 204, CAS# 30021-74-0, Entry# 24906

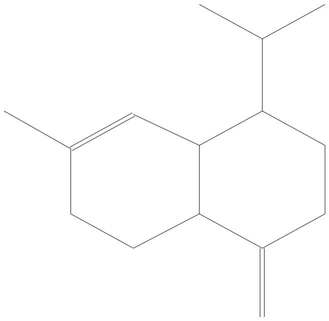

SI 921, RSI 929, replib, Entry# 24906, CAS# 30021-74-0, ç-Muurolene

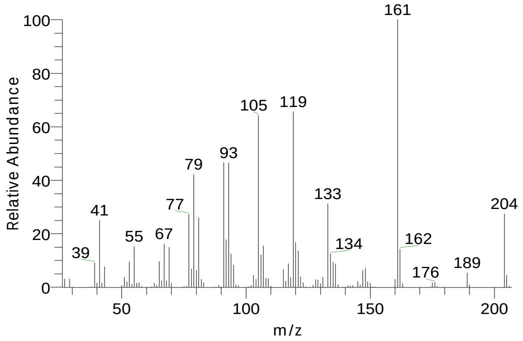

My GC-MS Report

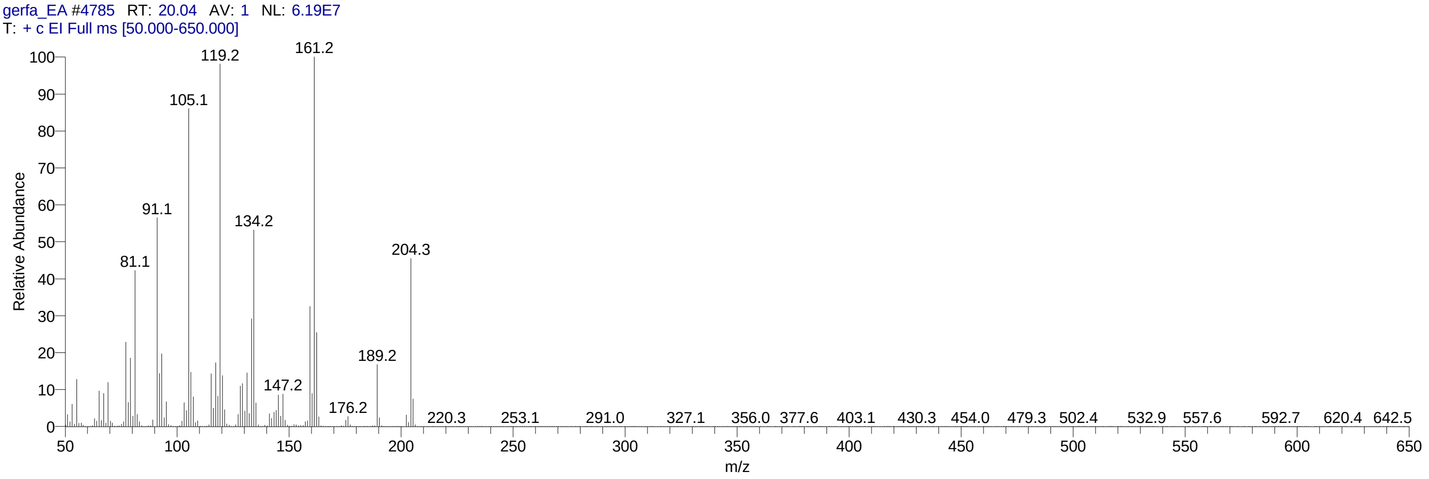

RT	Compound Name	Area %	Molecular Formula	Molecular Weight	Cas #	MF	Library
20.04	NAPHTHALENE, 1,2,3,5,6,8A-HEXAHYDRO-4,7-DIMETHYL-1-(1-METHYLETHYL)-, (1S-CIS)-	4.78	C15H24	204	483-76-1	942	WileyRegistry8e
20.04	NAPHTHALENE, 1,2,3,5,6,8A-HEXAHYDRO-4,7-DIMETHYL-1-(1-METHYLETHYL)-, (1S-CIS)-	4.78	C15H24	204	483-76-1	900	WileyRegistry8e
20.04	1-Isopropyl-4,7-dimethyl-1,2,3,5,6,8a-hexahydronaphthalene	4.78	C15H24	204	16729-01-4	921	mainlib

Compound Structure | Hit Spectrum

NAPHTHALENE, 1,2,3,5,6,8A-HEXAHYDRO-4,7-DIMETHYL-1-(1-METHYLETHYL)-, (1S-CIS)-
Formula C15H24, MW 204, CAS# 483-76-1, Entry# 89277
1-ISOPROPYL-4,7-DIMETHYL-1,2,3,5,6,8A-HEXAHYDRONAPHTHALENE #

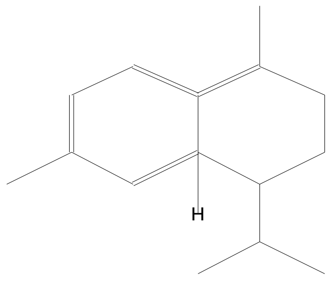

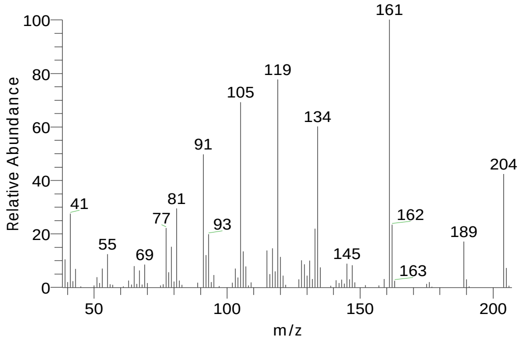

NAPHTHALENE, 1,2,3,5,6,8A-HEXAHYDRO-4,7-DIMETHYL-1-(1-METHYLETHYL)-, (1S-CIS)-
Formula C15H24, MW 204, CAS# 483-76-1, Entry# 89273
1-ISOPROPYL-4,7-DIMETHYL-1,2,3,5,6,8A-HEXAHYDRONAPHTHALENE #

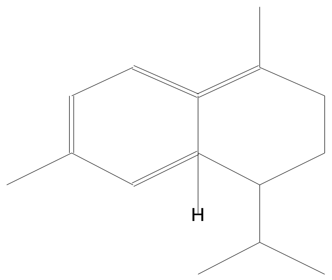

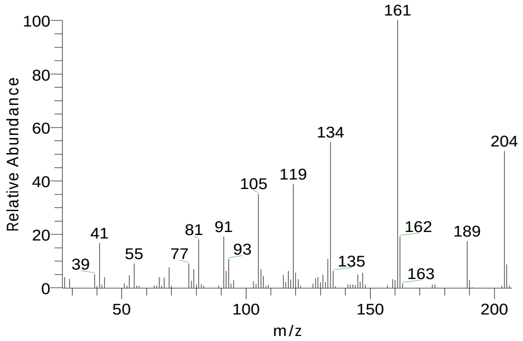

My GC-MS Report

Compound Structure | Hit Spectrum

1-Isopropyl-4,7-dimethyl-1,2,3,5,6,8a-hexahydronaphthalene
Formula C15H24, MW 204, CAS# 16729-01-4, Entry# 150583
Cadina-1(10),4-diene

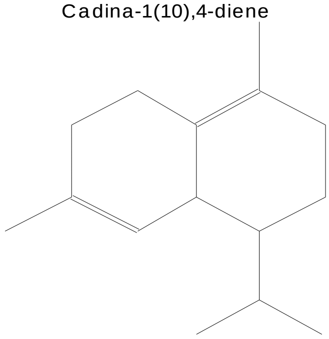

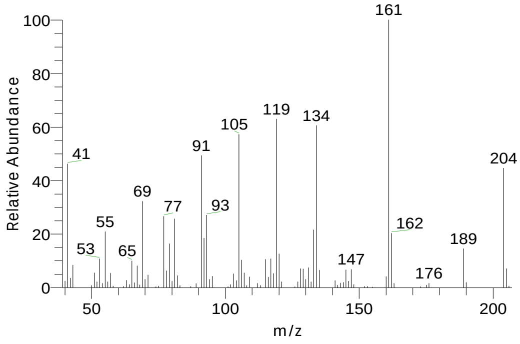

gerfa_EA #4838 RT: 20.22 AV: 1 NL: 9.18E7
T: + c EI Full ms [50.000-650.000]

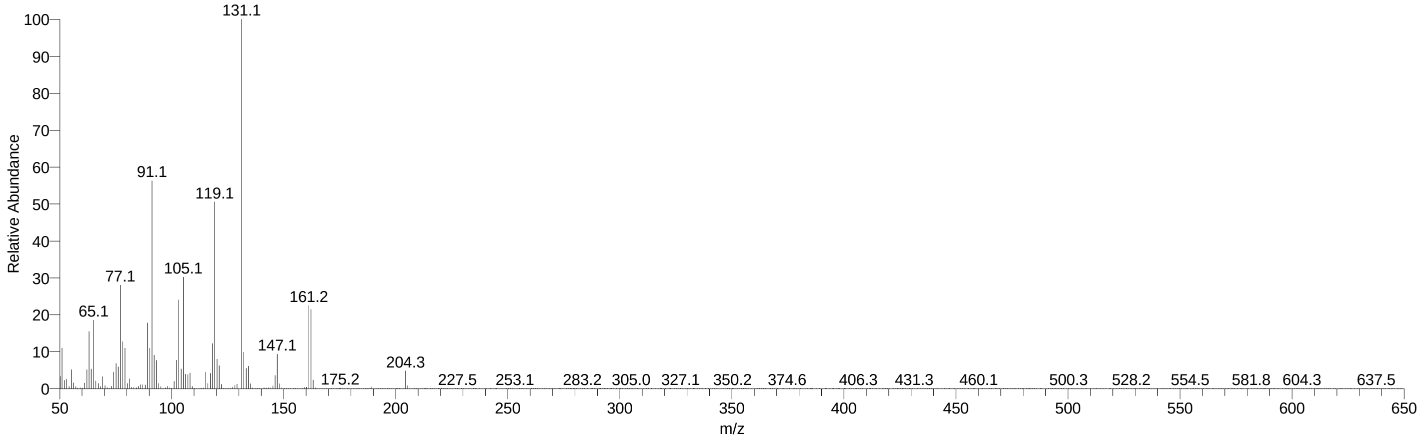

RT	Compound Name	Area %	Molecular Formula	Molecular Weight	Cas #	MF	Library
20.22	2-PROPENAL, 3-(4-METHOXYPHENYL)-	4.91	C10H10O2	162	1963-36-6	883	WileyRegistry8e
20.22	(2E)-3-(2-METHOXYPHENYL)-2-PROPENAL #	4.91	C10H10O2	162	1504-74-1	891	WileyRegistry8e
20.22	2-Propenal, 3-(2-methoxyphenyl)-	4.91	C10H10O2	162	1504-74-1	876	mainlib

Compound Structure | Hit Spectrum

2-PROPENAL, 3-(4-METHOXYPHENYL)-
Formula C10H10O2, MW 162, CAS# 1963-36-6, Entry# 45236
CINNAMALDEHYDE, P-METHOXY-

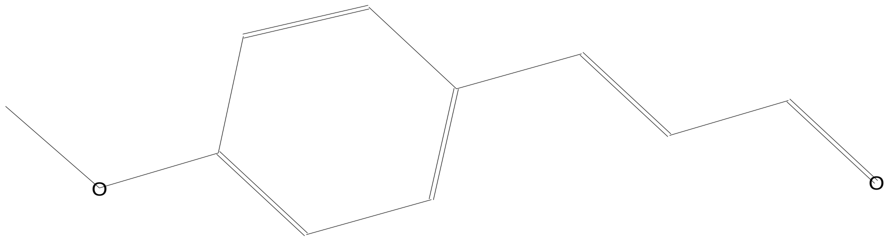

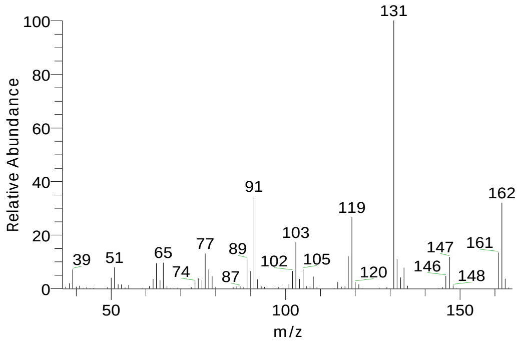

My GC-MS Report

Compound Structure | Hit Spectrum

(2E)-3-(2-METHOXYPHENYL)-2-PROPENAL #
Formula C10H10O2, MW 162, CAS# 1504-74-1, Entry# 45156
(2E)-3-(2-METHOXYPHENYL)-2-PROPENAL

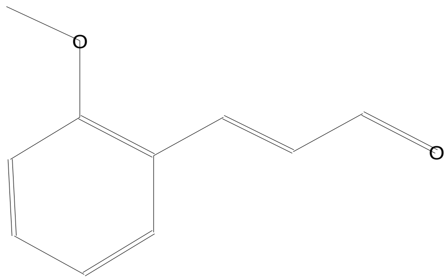

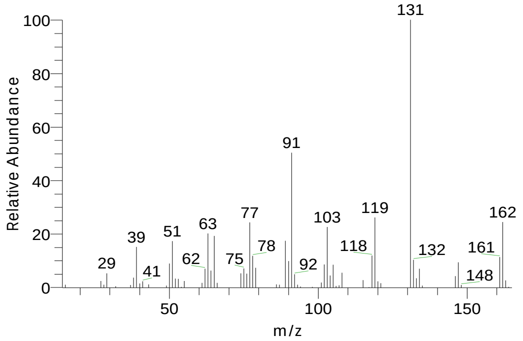

2-Propenal, 3-(2-methoxyphenyl)-
Formula C10H10O2, MW 162, CAS# 1504-74-1, Entry# 115637
Cinnamaldehyde, o-methoxy-

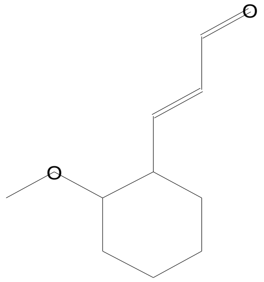

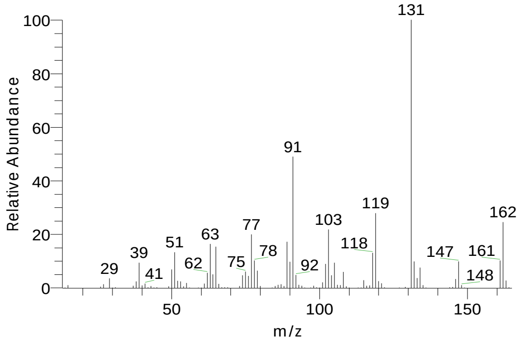

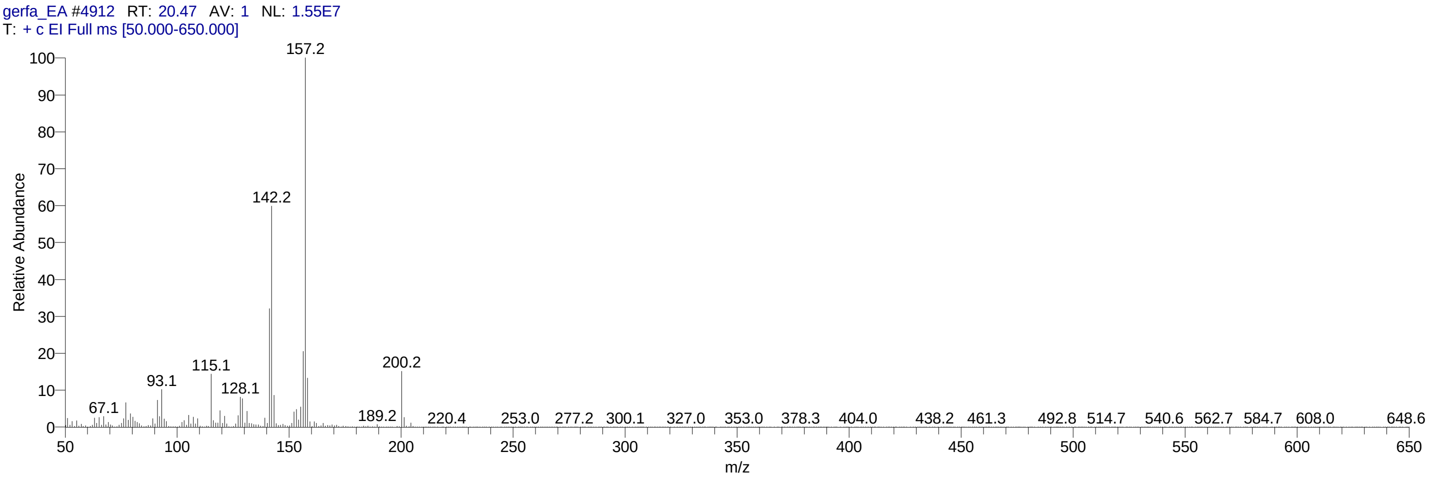

RT	Compound Name	Area %	Molecular Formula	Molecular Weight	Cas #	MF	Library
20.47	4-Isopropyl-6-methyl-1-methylene-1,2,3,4-tetrahydronaphthalene	0.43	C15H20	200	50277-34-4	934	mainlib
20.47	1,1,6-TRIMETHYL-1,2-DIHYDRONAPHTHALENE	0.43	C13H16	172	NA	956	WileyRegistry8e
20.47	à-Calacorene	0.43	C15H20	200	21391-99-1	940	mainlib

My GC-MS Report

Compound Structure	Hit Spectrum

4-Isopropyl-6-methyl-1-methylene-1,2,3,4-tetrahydronaphthalene
Formula C15H20, MW 200, CAS# 50277-34-4, Entry# 146807
Naphthalene, 1,2,3,4-tetrahydro-6-methyl-1-methylene-4-(1-methylethyl)-

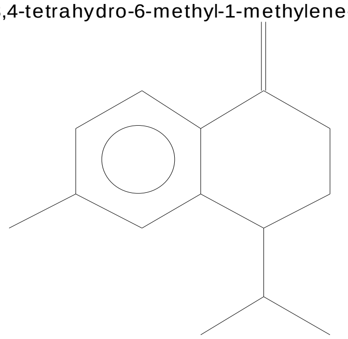

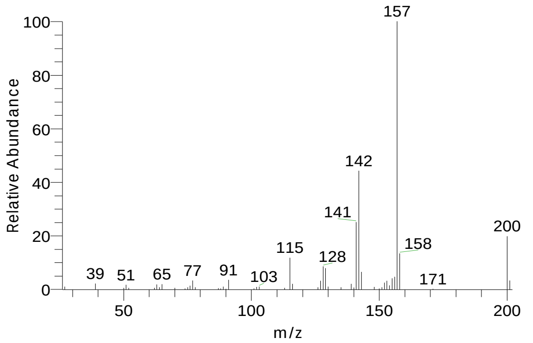

1,1,6-TRIMETHYL-1,2-DIHYDRO NAPHTHALENE
Formula C13H16, MW 172, CAS# NA, Entry# 56092
CALACORENE

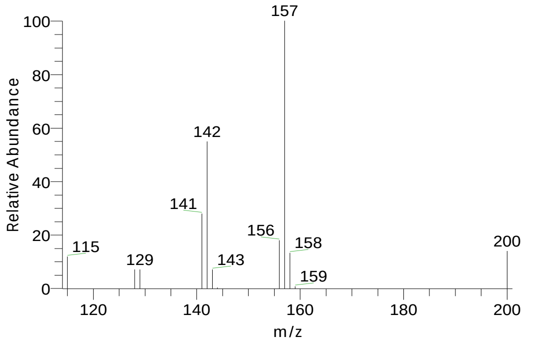

à-Calacorene
Formula C15H20, MW 200, CAS# 21391-99-1, Entry# 146806
1-Isopropyl-4,7-dimethyl-1,2-dihydronaphthalene, (S)-

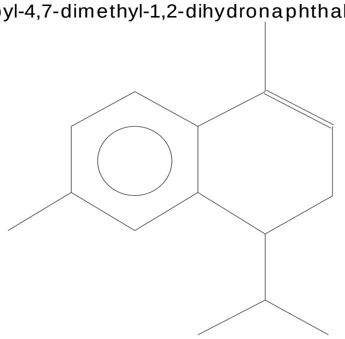

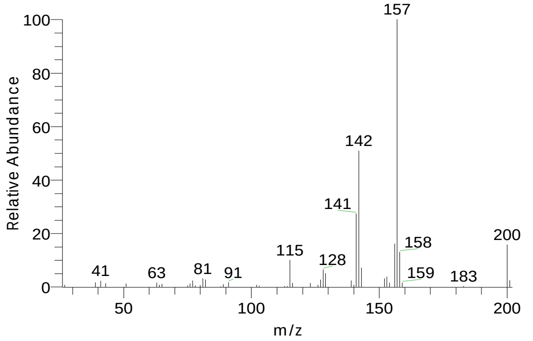

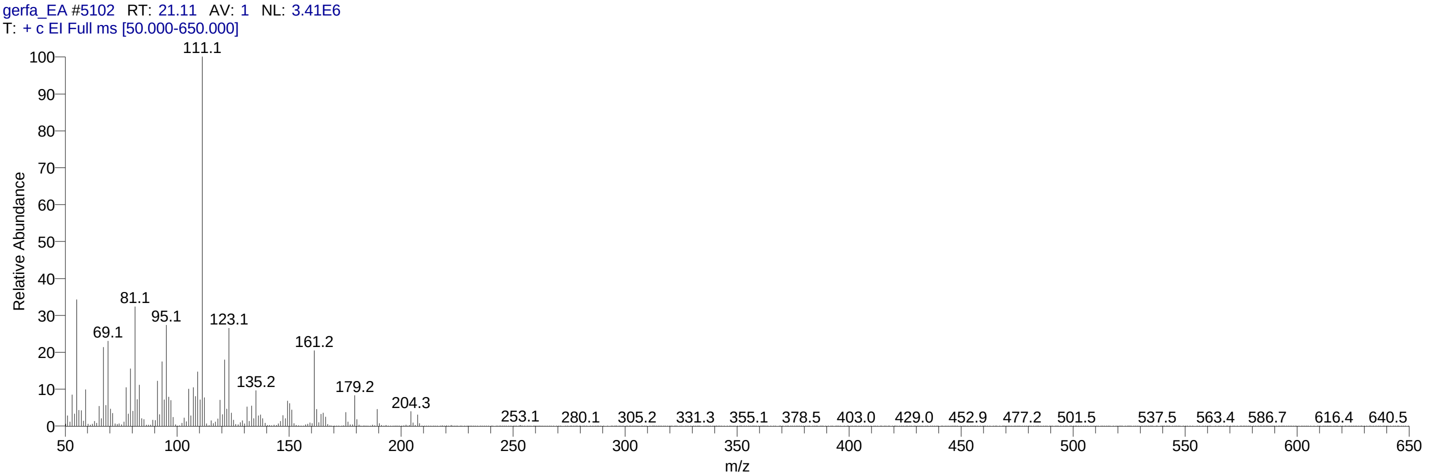

RT	Compound Name	Area %	Molecular Formula	Molecular Weight	Cas #	MF	Library
21.11	Caryophyllenyl alcohol	0.14	C15H26O	222	913176-41-7	901	mainlib
21.11	Caryophyllenyl alcohol	0.14	C15H26O	222	913176-41-7	856	replib

My GC-MS Report

RT	Compound Name	Area %	Molecular Formula	Molecular Weight	Cas #	MF	Library
21.11	4aH-Cycloprop[e]azulen-4a-ol, decahydro-1,1,4,7-tetramethyl-, [1aR-(1aà,4á,4aá,7à,7aá,7bà)]-	0.14	C15H26O	222	5986-49-2	804	replib

Compound Structure	Hit Spectrum

Caryophyllenyl alcohol
Formula C15H26O, MW 222, CAS# 913176-41-7, Entry# 88498
4,8,11,11-Tetramethylbicyclo[7.2.0]undec-3-en-5-ol #

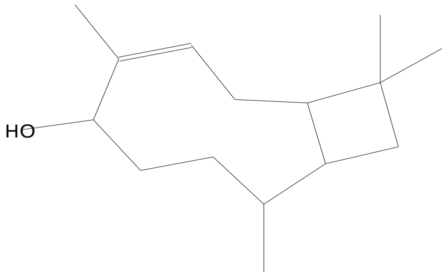

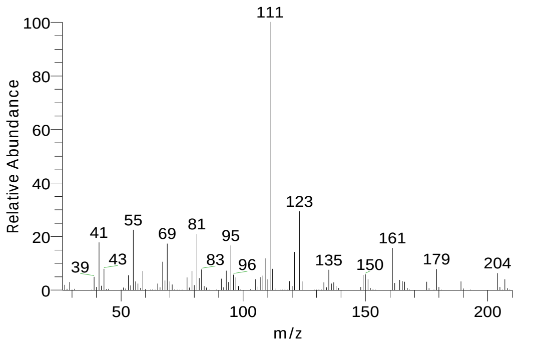

Caryophyllenyl alcohol
Formula C15H26O, MW 222, CAS# 913176-41-7, Entry# 17579
4,8,11,11-Tetramethylbicyclo[7.2.0]undec-3-en-5-ol #

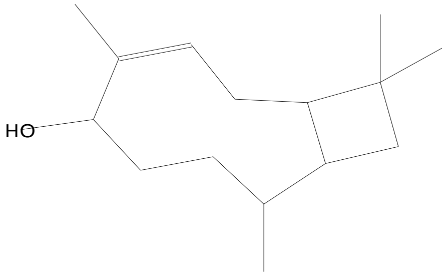

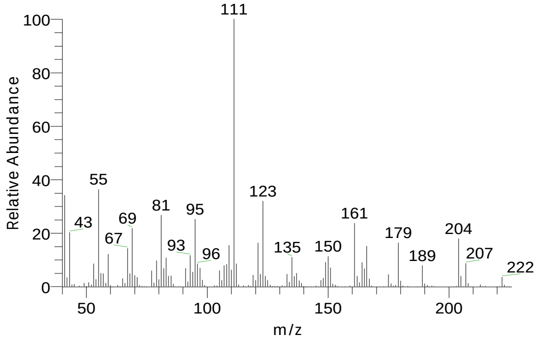

Formula C15H26O, MW 222, CAS# 5986-49-2, Entry# 17563
(1aR,4S,4aS,7R,7aS,7bR)-1,1,4,7-Tetramethyldecahydro-1H-cyclopropa[e]azulen-4a-ol

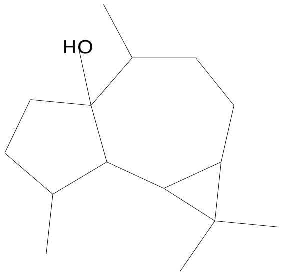

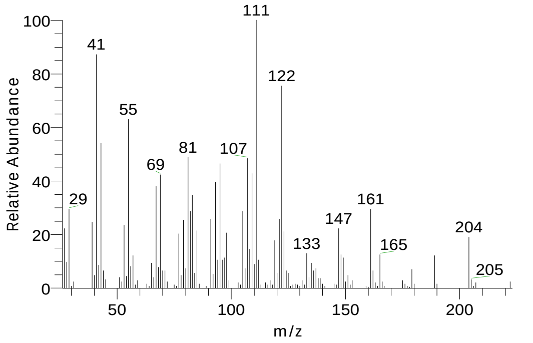

gerfa_EA #5193 RT: 21.41 AV: 1 NL: 1.68E6
T: + c EI Full ms [50.000-650.000]

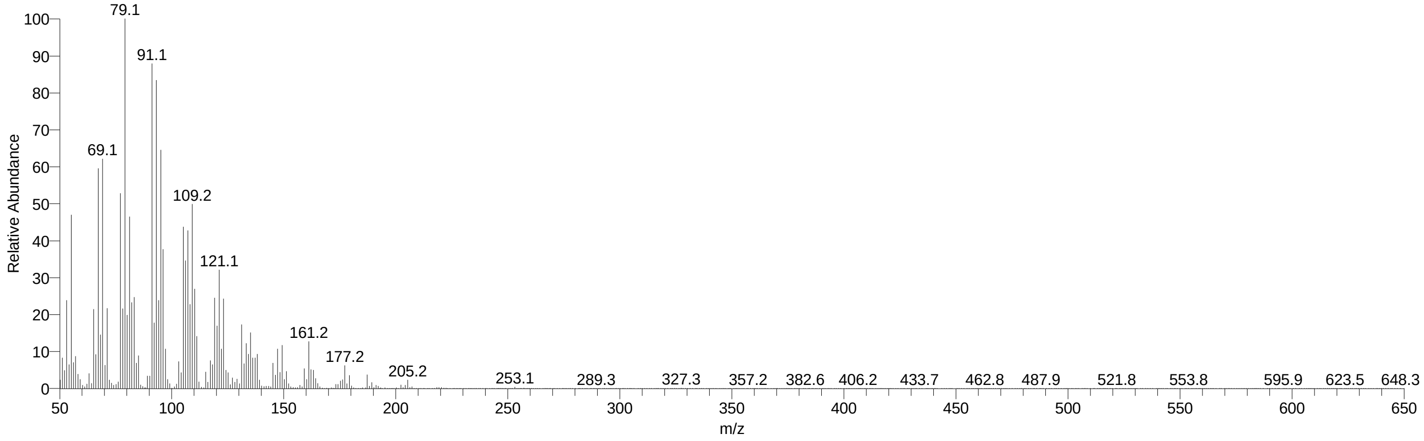

My GC-MS Report

RT	Compound Name	Area %	Molecular Formula	Molecular Weight	Cas #	MF	Library
21.41	(-)-5-OXATRICYCLO[8.2.0.0(4,6)]DODECANE,,12-TRIMETHYL-9-METHYLENE-, [1R-(1R*,4R*,6R*,10S*)]-	0.14	C15H24O	220	1139-30-6	921	WileyRegistry8e
21.41	(-)-5-OXATRICYCLO[8.2.0.0(4,6)]DODECANE,,12-TRIMETHYL-9-METHYLENE-, [1R-(1R*,4R*,6R*,10S*)]-	0.14	C15H24O	220	1139-30-6	925	WileyRegistry8e
21.41	Caryophyllene oxide	0.14	C15H24O	220	1139-30-6	913	mainlib

Compound Structure	Hit Spectrum

Formula C15H24O, MW 220, CAS# 1139-30-6, Entry# 107245
5-OXATRICYCLO[8.2.0.04,6]DODECANE, 4,12,12-TRIMETHYL-9-METHYLENE-, (1R,4R,6R,10S)-

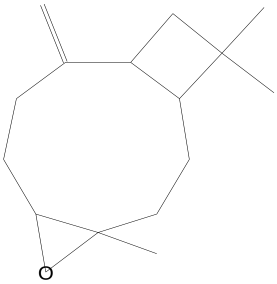

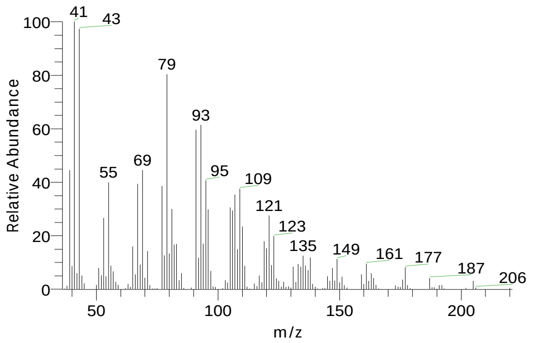

Formula C15H24O, MW 220, CAS# 1139-30-6, Entry# 107244
5-OXATRICYCLO[8.2.0.04,6]DODECANE, 4,12,12-TRIMETHYL-9-METHYLENE-, (1R,4R,6R,10S)-

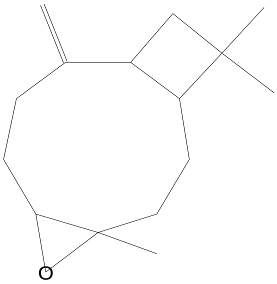

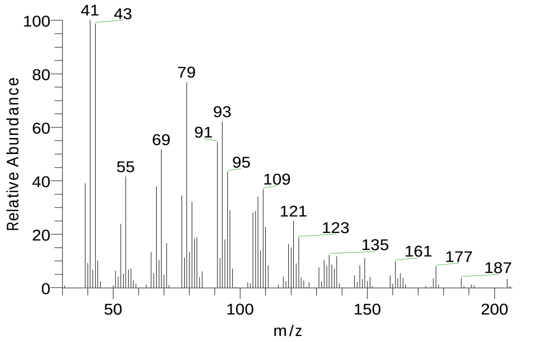

Caryophyllene oxide
Formula C15H24O, MW 220, CAS# 1139-30-6, Entry# 6247

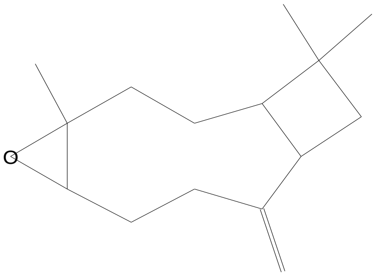

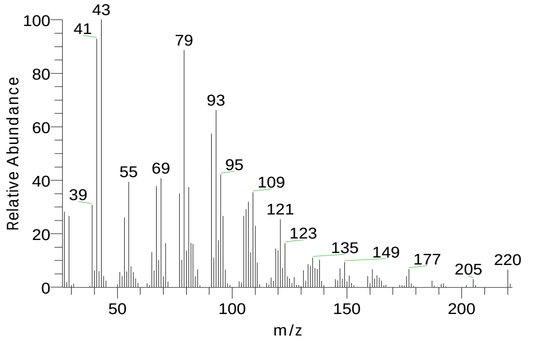

My GC-MS Report

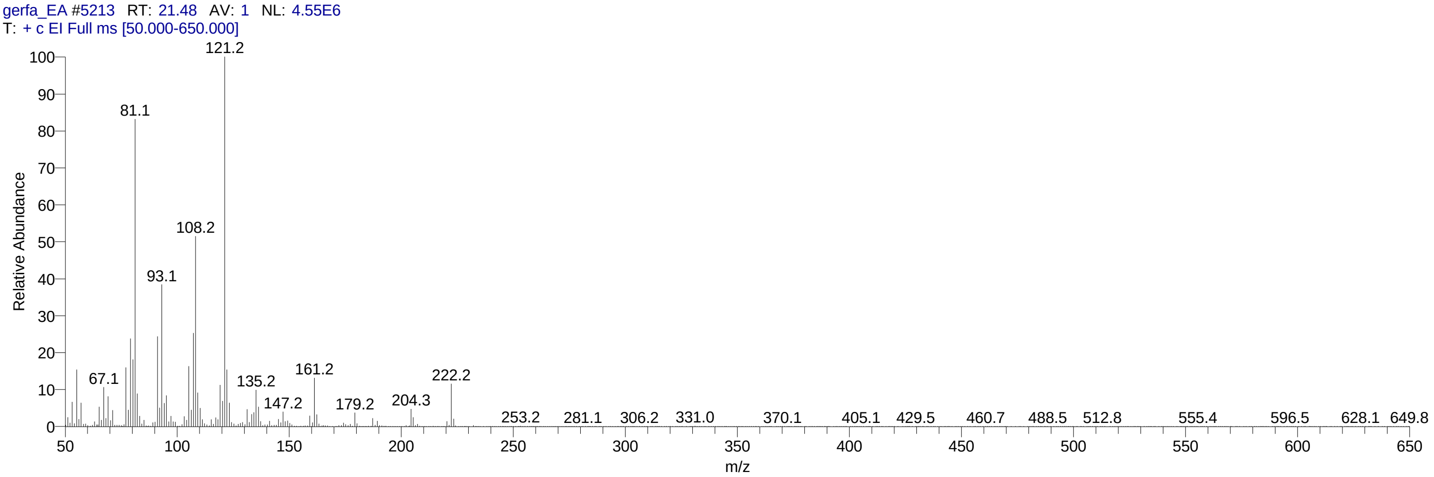

RT	Compound Name	Area %	Molecular Formula	Molecular Weight	Cas #	MF	Library
21.48	(5S,6R,7S,10R)-7-Isopropyl-2,10-dimethylspiro [4.5]dec-1-en-6-ol	0.16	C15H26O	222	72203-99-7	905	mainlib
21.48	gleenol	0.16	C15H26O	222	NA	877	mainlib
21.48	ç-Elemene	0.16	C15H24	204	29873-99-2	820	mainlib

Compound Structure	Hit Spectrum

(5S,6R,7S,10R)-7-Isopropyl-2,10-dimethylspiro[4.5]dec-1-en-6-ol
Formula C15H26O, MW 222, CAS# 72203-99-7, Entry# 102652
Spiro[4.5]dec-1-en-6-ol, 2,10-dimethyl-7-(1-methylethyl)-, (5S,6R,7S,10R)-

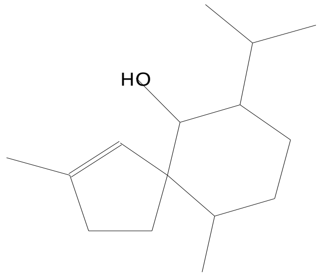

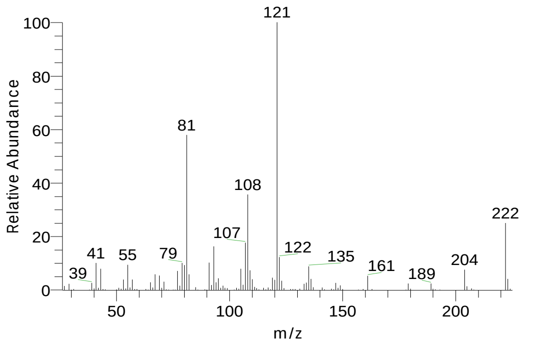

SI 864, RSI 877, mainlib, Entry# 102651, CAS# NA, gleenol

gleenol
Formula C15H26O, MW 222, CAS# NA, Entry# 102651
$:28MYLXGCVCCZCOHU-UHFFFAOYSA-N

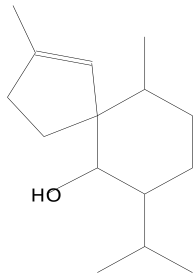

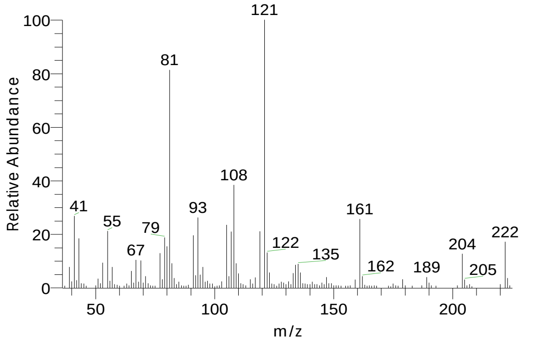

SI 775, RSI 820, mainlib, Entry# 102784, CAS# 29873-99-2, ç-Elemene

ç-Elemene
Formula C15H24, MW 204, CAS# 29873-99-2, Entry# 102784
1-Methyl-2-(1-methylethenyl)-4-(1-methylethylidene)-1-vinylcyclohexane), (1R-trans)-

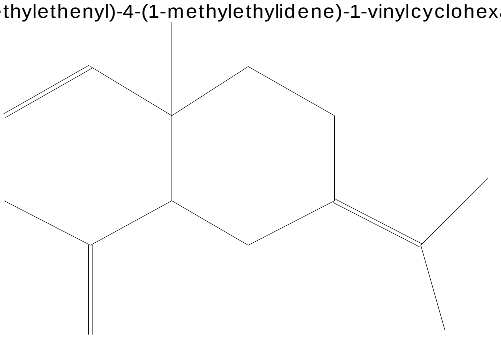

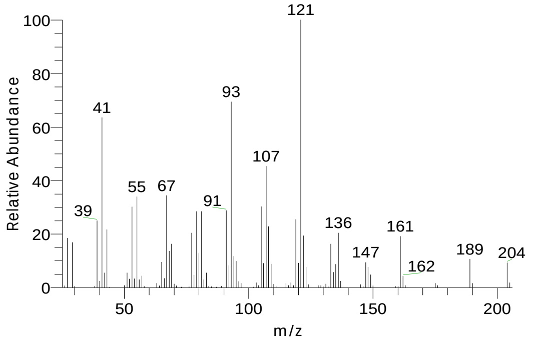

My GC-MS Report

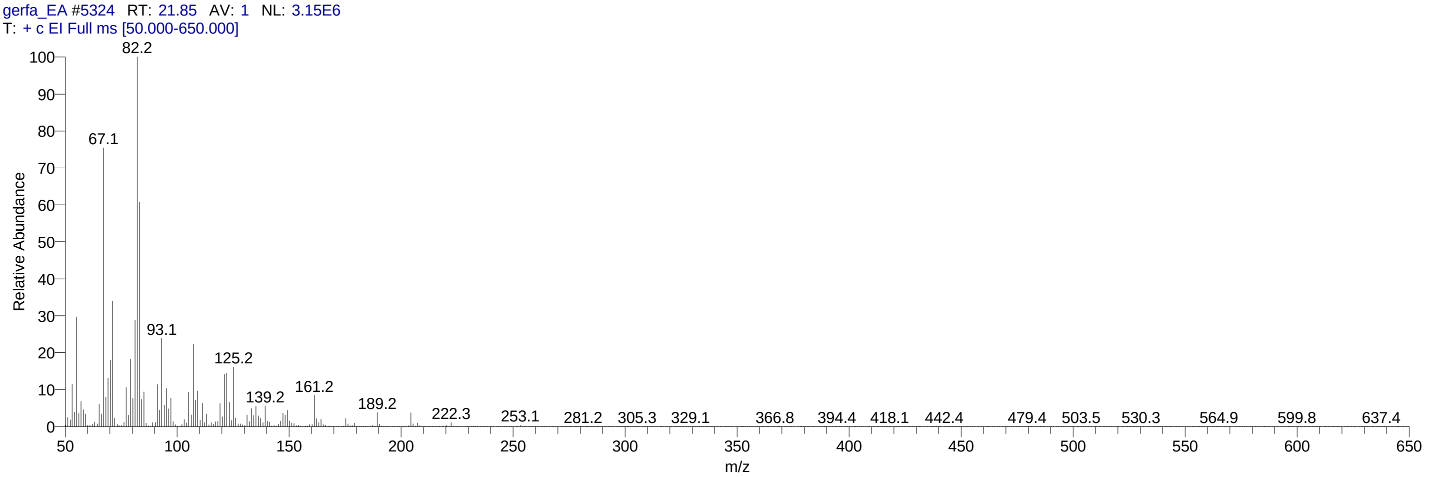

RT	Compound Name	Area %	Molecular Formula	Molecular Weight	Cas #	MF	Library
21.85	3,7-Cycloundecadien-1-ol, 1,5,5,8-tetramethyl-	0.11	C15H26O	222	118014-38-3	889	mainlib
21.85	1,5,5,8-TETRAMETHYL-3,7-CYCLOUNDECADIEN-1-OL #	0.11	C15H26O	222	118014-38-3	889	WileyRegistry8e
21.85	1,5,5,8-TETRAMETHYL-3,7-CYCLOUNDECADIEN-1-OL #	0.11	C15H26O	222	118014-38-3	889	WileyRegistry8e

Compound Structure	Hit Spectrum

3,7-Cycloundecadien-1-ol, 1,5,5,8-tetramethyl-
Formula C15H26O, MW 222, CAS# 118014-38-3, Entry# 51254
1,5,5,8-Tetramethyl-3,7-cycloundecadien-1-ol #

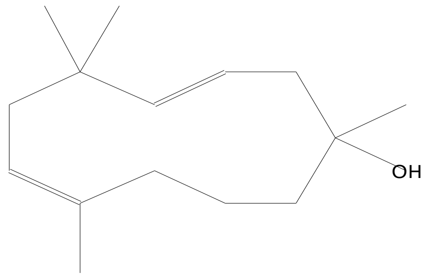

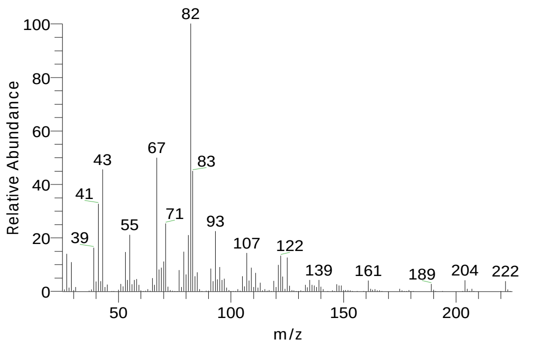

1,5,5,8-TETRAMETHYL-3,7-CYCLOUNDECADIEN-1-OL #
Formula C15H26O, MW 222, CAS# 118014-38-3, Entry# 109756
1,5,5,8-TETRAMETHYL-3,7-CYCLOUNDECADIEN-1-OL

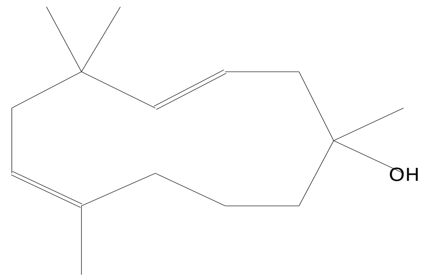

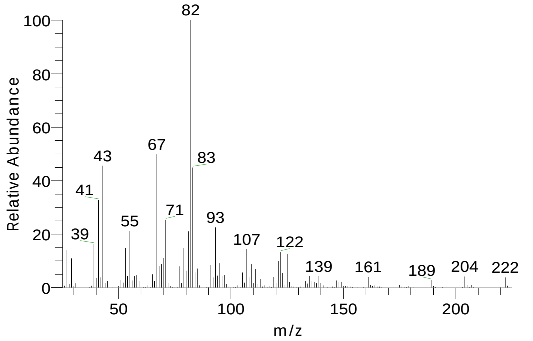

1,5,5,8-TETRAMETHYL-3,7-CYCLOUNDECADIEN-1-OL #
Formula C15H26O, MW 222, CAS# 118014-38-3, Entry# 385407
1,5,5,8-TETRAMETHYL-3,7-CYCLOUNDECADIEN-1-OL

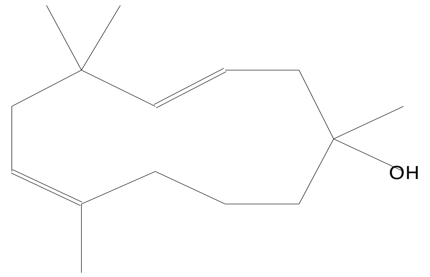

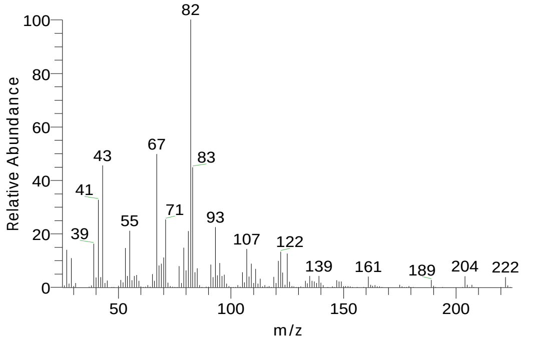

My GC-MS Report

gerfa_EA #5344 RT: 21.92 AV: 1 NL: 1.69E6
T: + c EI Full ms [50.000-650.000]

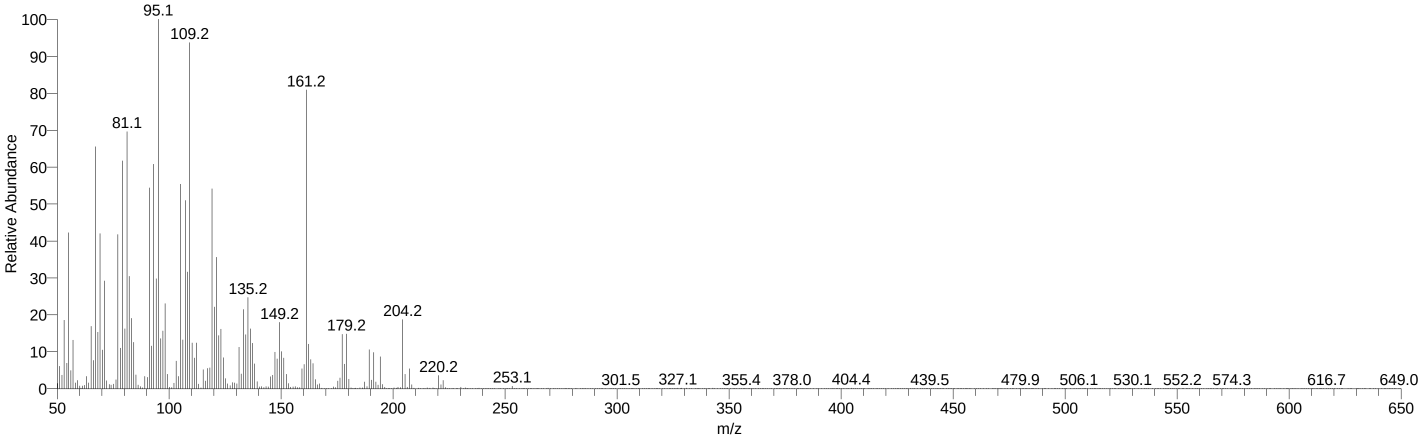

RT	Compound Name	Area %	Molecular Formula	Molecular Weight	Cas #	MF	Library
21.92	(1S,3aS,4S,5S,7aR,8R)-5-Isopropyl-1,7a-dimethyloctahydro-1H-1,4-methanoinden-8-ol	0.14	C15H26O	222	21966-93-8	896	mainlib
21.92	Globulol	0.14	C15H26O	222	51371-47-2	844	mainlib
21.92	1,1,4,7-TETRAMETHYLDECAHYDRO-1H-CYCLOPROPA[E]AZULEN-4-OL #	0.14	C15H26O	222	51371-47-2	844	WileyRegistry8e

Compound Structure | Hit Spectrum

(1S,3aS,4S,5S,7aR,8R)-5-Isopropyl-1,7a-dimethyloctahydro-1H-1,4-methanoinden-8-ol
Formula C15H26O, MW 222, CAS# 21966-93-8, Entry# 86038
Copaborneol

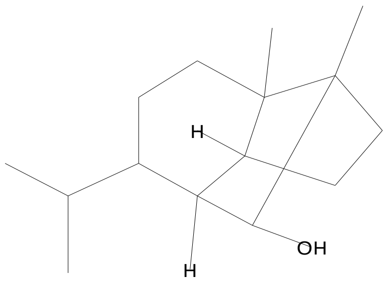

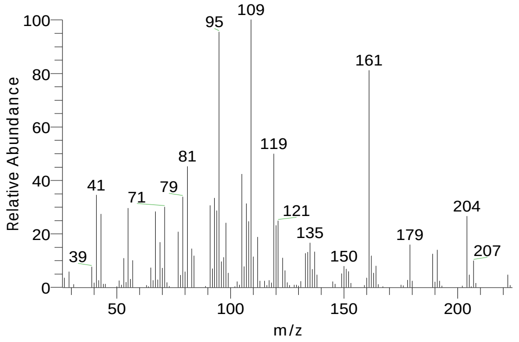

SI 816, RSI 844, mainlib, Entry# 6178, CAS# 51371-47-2, Globulol

Globulol
Formula C15H26O, MW 222, CAS# 51371-47-2, Entry# 6178
1,1,4,7-Tetramethyldecahydro-1H-cyclopropa[e]azulen-4-ol, (1aà,4à,4aà,7à,7aá,7bà)-

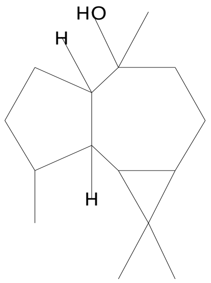

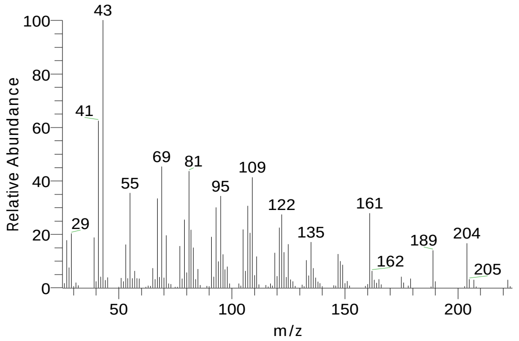

1,1,4,7-TETRAMETHYLDECAHYDRO-1H-CYCLOPROPA[E]AZULEN-4-OL #
Formula C15H26O, MW 222, CAS# 51371-47-2, Entry# 390796
1,1,4,7-TETRAMETHYLDECAHYDRO-1H-CYCLOPROPA[E]AZULEN-4-OL

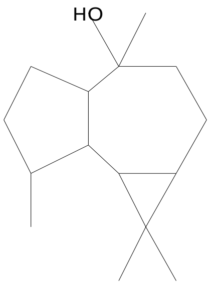

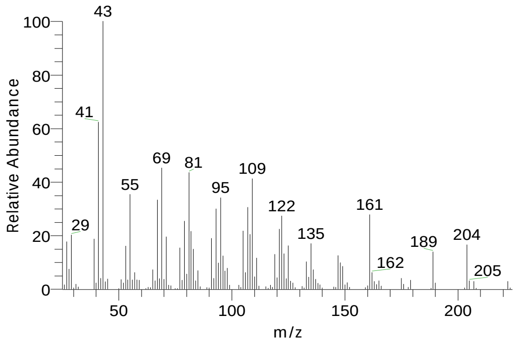

My GC-MS Report

gerfa_EA #5403 RT: 22.12 AV: 1 NL: 2.07E6
T: + c EI Full ms [50.000-650.000]

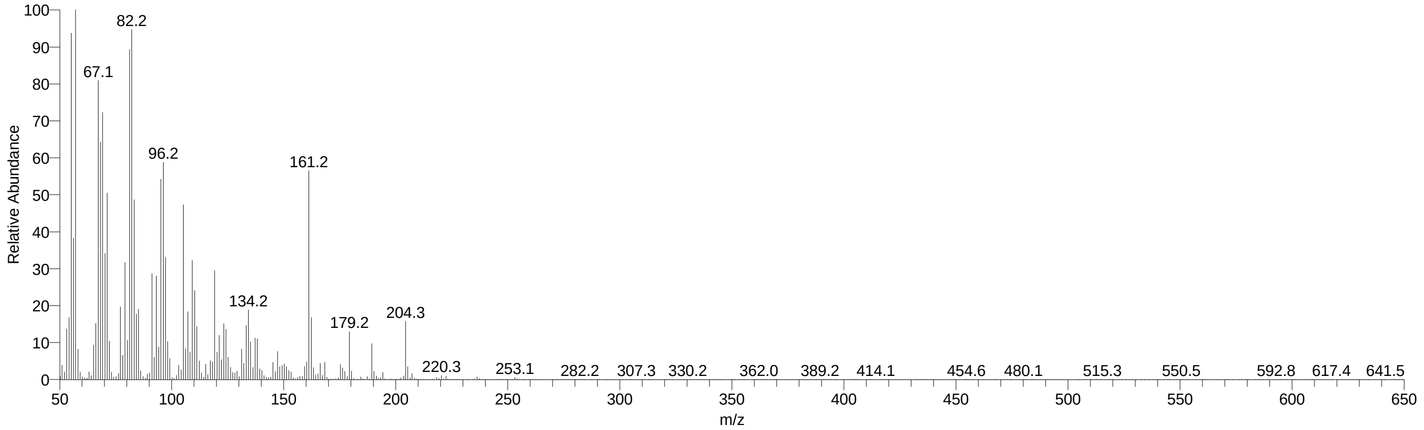

RT	Compound Name	Area %	Molecular Formula	Molecular Weight	Cas #	MF	Library
22.12	6-epi-shyobunol	0.22	C15H26O	222	NA	806	mainlib
22.12	Carotol	0.22	C15H26O	222	465-28-1	813	replib
22.12	3A(1H)-AZULENOL, 2,3,4,5,8,8A-HEXAHYDRO-6,8A-DIMETHYL-3-(1-METHYLETHYL)-, [3R-(3à,3Aà,8Aà)]-	0.22	C15H26O	222	465-28-1	808	WileyRegistry8e

Compound Structure | Hit Spectrum

6-epi-shyobunol
Formula C15H26O, MW 222, CAS# NA, Entry# 49759
elema-1,3-dien-6à-ol

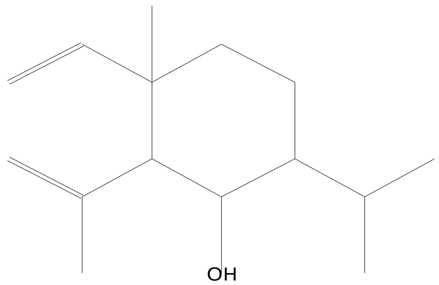

SI 794, RSI 806, mainlib, Entry# 49759, CAS# NA, 6-epi-shyobunol

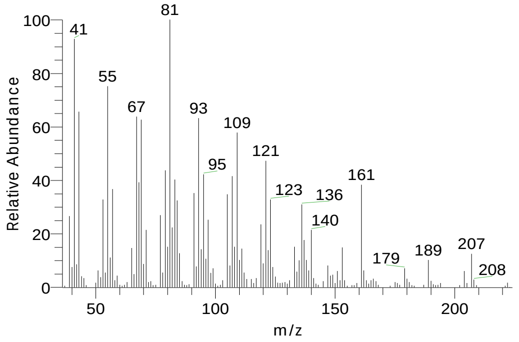

Carotol
Formula C15H26O, MW 222, CAS# 465-28-1, Entry# 24838
3a(1H)-Azulenol, 2,3,4,5,8,8a-hexahydro-6,8a-dimethyl-3-(1-methylethyl)-, [3R-(3à,3aà,8aà)]-

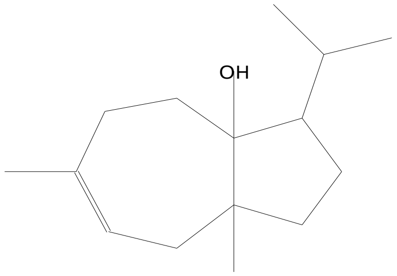

SI 774, RSI 813, replib, Entry# 24838, CAS# 465-28-1, Carotol

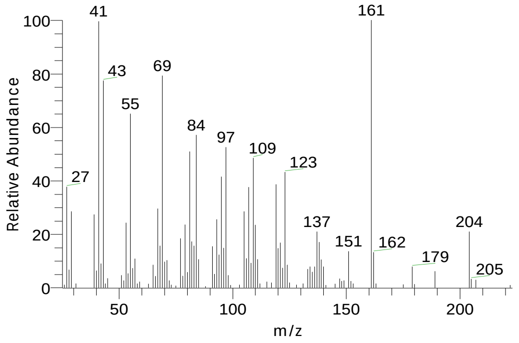

Formula C15H26O, MW 222, CAS# 465-28-1, Entry# 109801
3-ISOPROPYL-6,8A-DIMETHYL-2,3,4,5,8,8A-HEXAHYDRO-3A(1H)-AZULENOL #

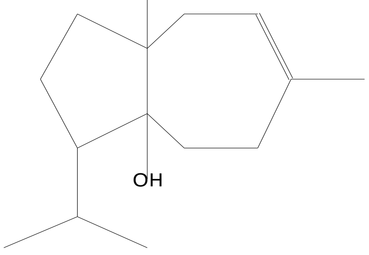

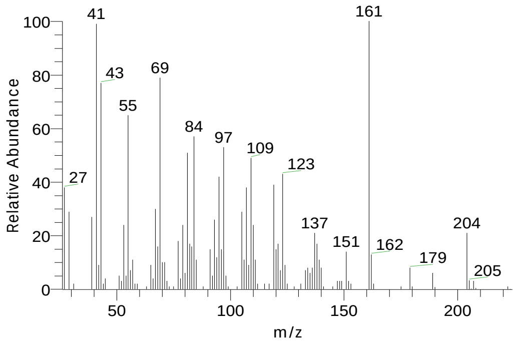

My GC-MS Report

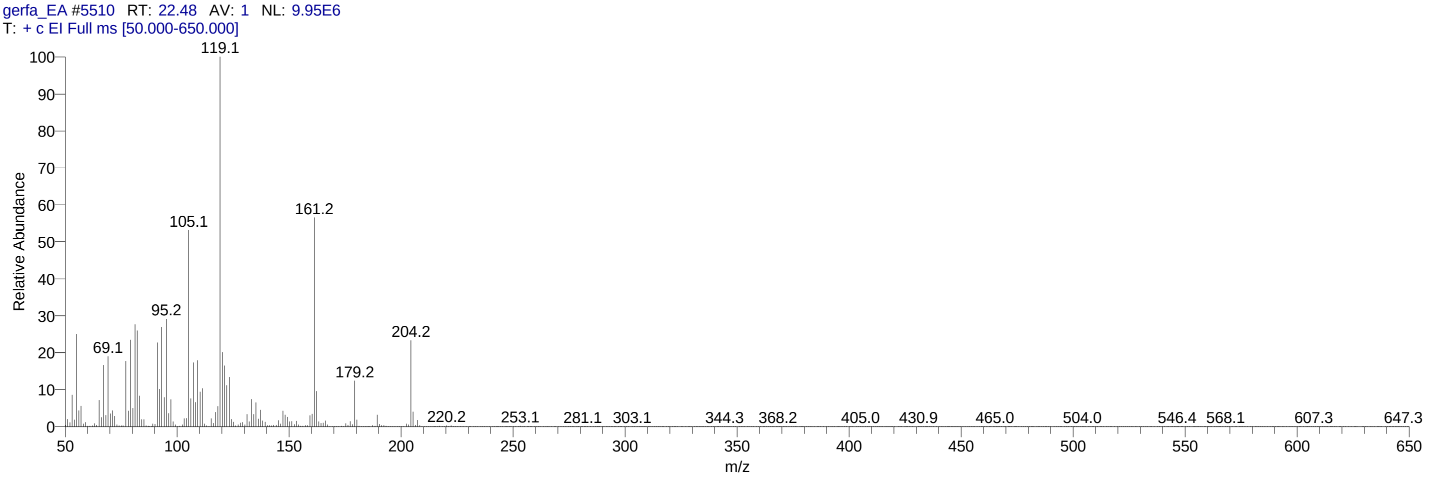

RT	Compound Name	Area %	Molecular Formula	Molecular Weight	Cas #	MF	Library
22.48	Di-epi-1,10-cubenol	0.53	C15H26O	222	73365-77-2	939	mainlib
22.48	Epicubenol	0.53	C15H26O	222	19912-67-5	919	mainlib
22.48	Cubenol	0.53	C15H26O	222	21284-22-0	914	mainlib

Compound Structure | Hit Spectrum

Di-epi-1,10-cubenol
Formula C15H26O, MW 222, CAS# 73365-77-2, Entry# 100531
(1S,4S,4aS,8aR)-1-Isopropyl-4,7-dimethyl-1,3,4,5,6,8a-hexahydronaphthalen-4a(2H)-ol

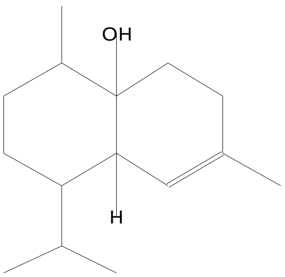

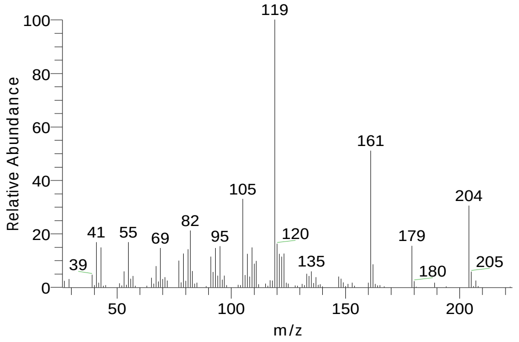

SI 904, RSI 919, mainlib, Entry# 100535, CAS# 19912-67-5, Epicubenol

Epicubenol
Formula C15H26O, MW 222, CAS# 19912-67-5, Entry# 100535
(1S,4R,4aS,8aR)-1-Isopropyl-4,7-dimethyl-1,2,3,4,4a,5,6,8a-octahydronaphthalen-4a-ol

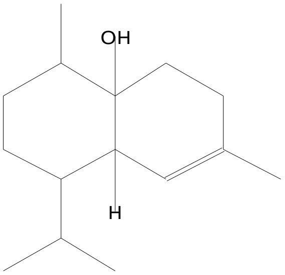

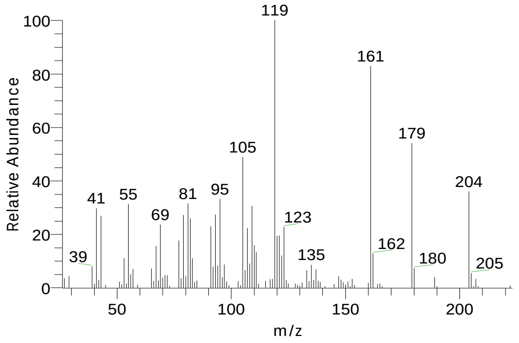

SI 889, RSI 914, mainlib, Entry# 98767, CAS# 21284-22-0, Cubenol

Cubenol
Formula C15H26O, MW 222, CAS# 21284-22-0, Entry# 98767
1-Isopropyl-4,7-dimethyl-1,3,4,5,6,8a-hexahydro-4a(2H)-naphthalenol-, [1S-(1à,4á,4aá,8aà)]-

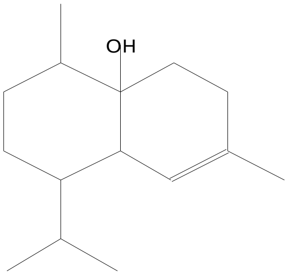

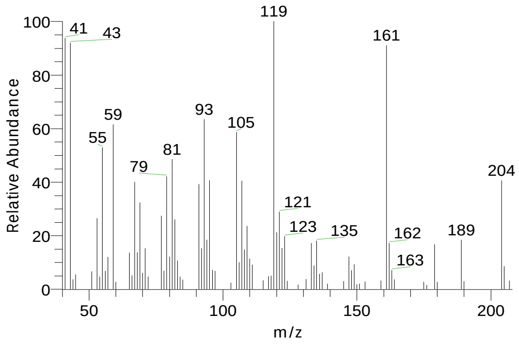

My GC-MS Report

gerfa_EA #5606 RT: 22.80 AV: 1 NL: 1.35E7
T: + c EI Full ms [50.000-650.000]

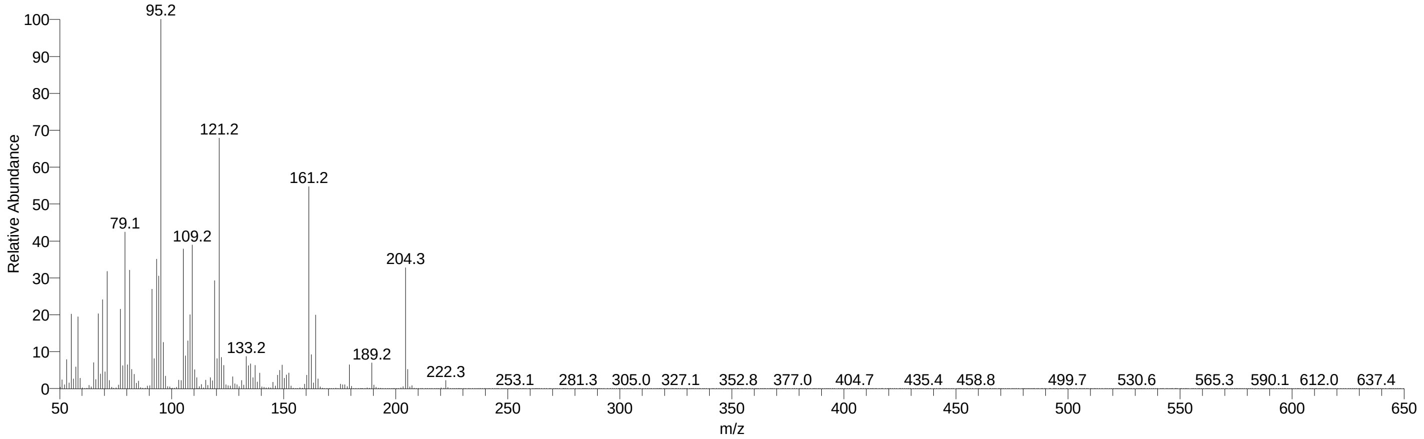

RT	Compound Name	Area %	Molecular Formula	Molecular Weight	Cas #	MF	Library
22.80	.tau.-Muurolol	0.77	C15H26O	222	19912-62-0	949	replib
22.80	à-Cadinol	0.77	C15H26O	222	481-34-5	934	replib
22.80	.tau.-Muurolol	0.77	C15H26O	222	19912-62-0	923	mainlib

Compound Structure	Hit Spectrum

.tau.-Muurolol
Formula C15H26O, MW 222, CAS# 19912-62-0, Entry# 3342
4-Isopropyl-1,6-dimethyl-1,2,3,4,4a,7,8,8a-octahydro-1-naphthalenol-, [1S-(1à,4à,4aà,8aà)]-

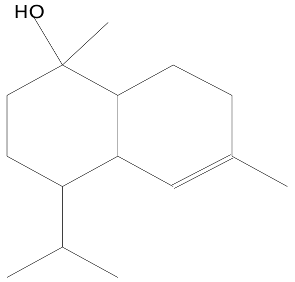

SI 932, RSI 949, replib, Entry# 3342, CAS# 19912-62-0, .tau.-Muurolol

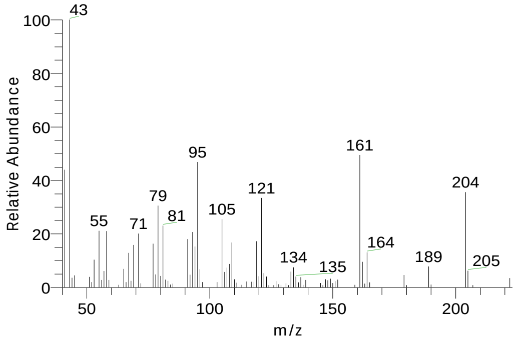

à-Cadinol
Formula C15H26O, MW 222, CAS# 481-34-5, Entry# 3029
4-Isopropyl-1,6-dimethyl-1,2,3,4,4a,7,8,8a-octahydro-1-naphthalenol #

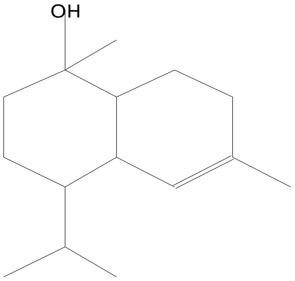

SI 924, RSI 934, replib, Entry# 3029, CAS# 481-34-5, à-Cadinol

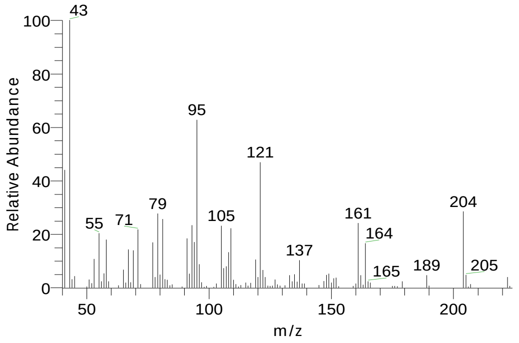

.tau.-Muurolol
Formula C15H26O, MW 222, CAS# 19912-62-0, Entry# 68534
4-Isopropyl-1,6-dimethyl-1,2,3,4,4a,7,8,8a-octahydro-1-naphthalenol-, [1S-(1à,4à,4aà,8aà)]-

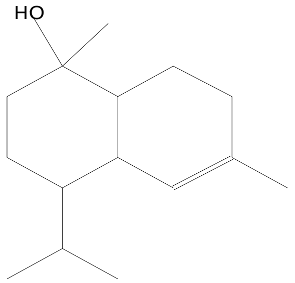

SI 923, RSI 923, mainlib, Entry# 68534, CAS# 19912-62-0, .tau.-Muurolol

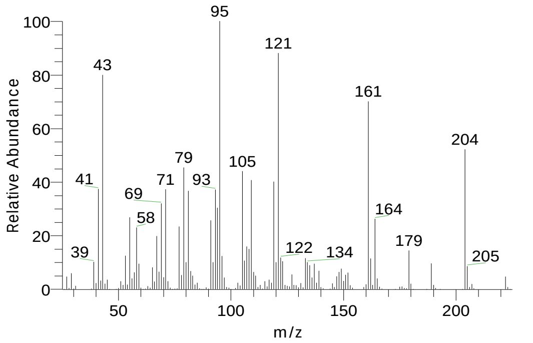

My GC-MS Report

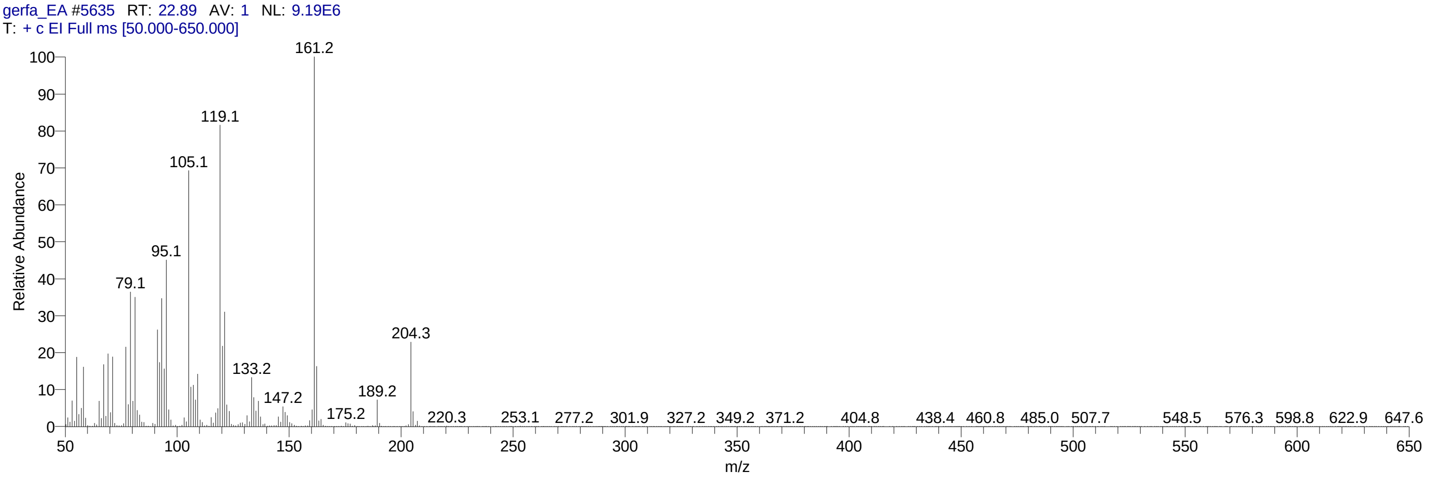

RT	Compound Name	Area %	Molecular Formula	Molecular Weight	Cas #	MF	Library
22.89	1-Naphthalenol, 1,2,3,4,4a,7,8,8a-octahydro-1,6-dimethyl-4-(1-methylethyl)-, [1R-(1à,4á,4aá,8aá)]-	0.37	C15H26O	222	19435-97-3	947	mainlib
22.89	1-NAPHTHALENOL, 1,2,3,4,4A,7,8,8A-OCTAHYDRO-1,6-DIMETHYL-4-(1-METHYLETHYL)-, [1R-(1à,4á,4Aá,8Aá)]-	0.37	C15H26O	222	19435-97-3	947	WileyRegistry8e
22.89	1-Naphthalenol, 1,2,3,4,4a,7,8,8a-octahydro-1,6-dimethyl-4-(1-methylethyl)-, [1R-(1à,4á,4aá,8aá)]-	0.37	C15H26O	222	19435-97-3	934	replib

Compound Structure	Hit Spectrum

Formula C15H26O, MW 222, CAS# 19435-97-3, Entry# 150252
(1R,4S,4aR,8aS)-4-Isopropyl-1,6-dimethyl-1,2,3,4,4a,7,8,8a-octahydronaphthalen-1-ol

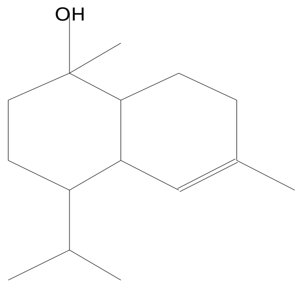

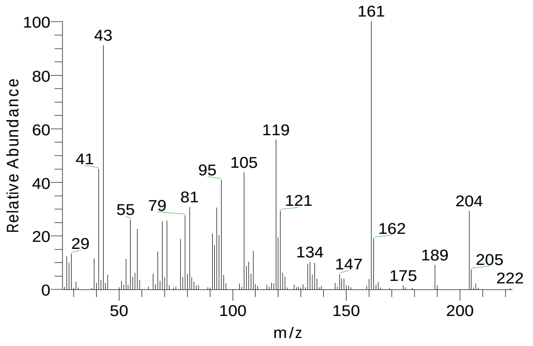

Formula C15H26O, MW 222, CAS# 19435-97-3, Entry# 109844
4-ISOPROPYL-1,6-DIMETHYL-1,2,3,4,4A,7,8,8A-OCTAHYDRO-1-NAPHTHALENOL #

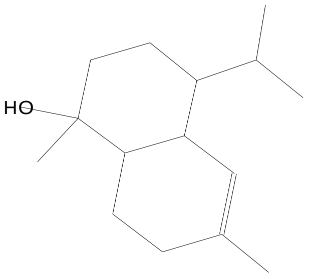

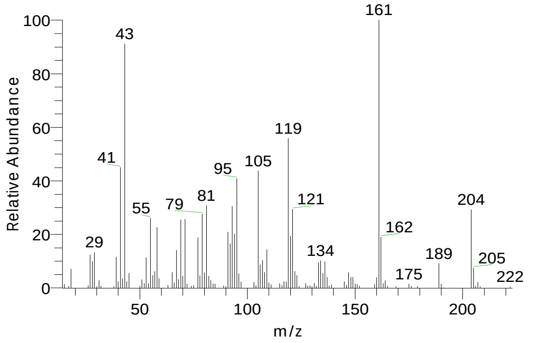

My GC-MS Report

Compound Structure | Hit Spectrum

Formula C15H26O, MW 222, CAS# 19435-97-3, Entry# 24849
(1R,4S,4aR,8aS)-4-Isopropyl-1,6-dimethyl-1,2,3,4,4a,7,8,8a-octahydronaphthalen-1-ol

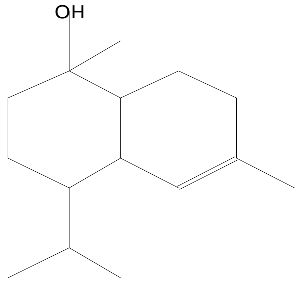

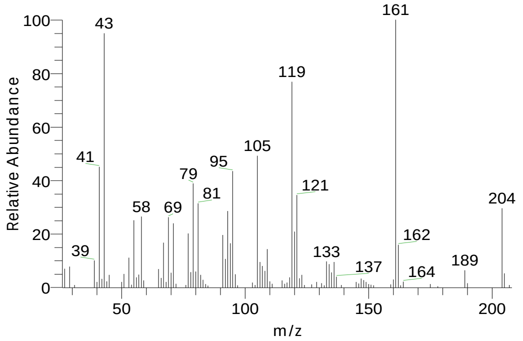

gerfa_EA #7757 RT: 30.01 AV: 1 NL: 7.40E6
T: + c EI Full ms [50.000-650.000]

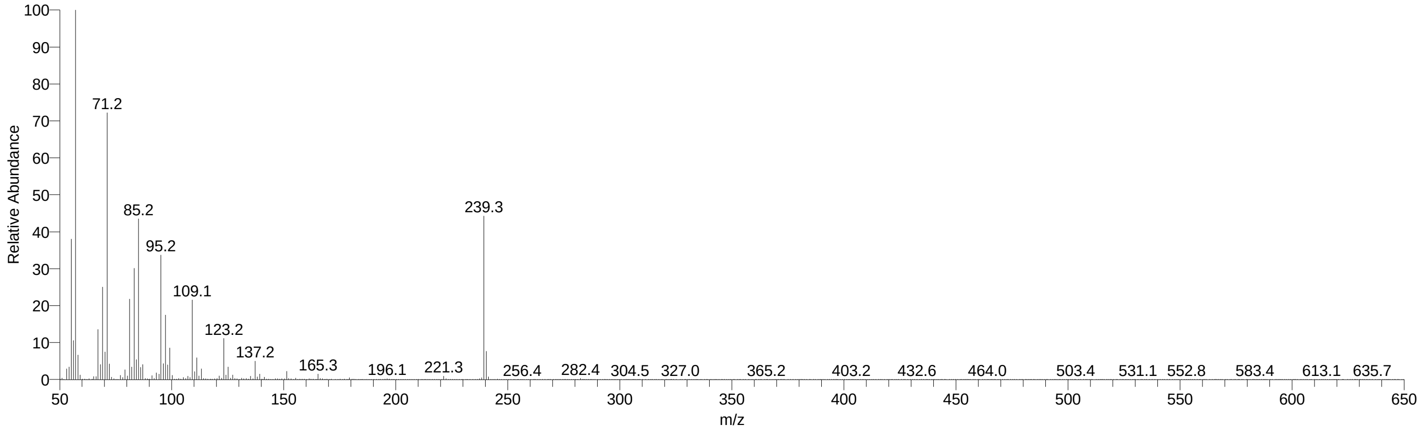

RT	Compound Name	Area %	Molecular Formula	Molecular Weight	Cas #	MF	Library
30.01	Palmitic acid vinyl ester	0.25	C18H34O2	282	693-38-9	919	replib
30.01	Palmitic acid vinyl ester	0.25	C18H34O2	282	693-38-9	862	mainlib
30.01	Hexadecanoic acid, 4-nitrophenyl ester	0.25	C22H35NO4	377	1492-30-4	823	mainlib

Compound Structure | Hit Spectrum

Palmitic acid vinyl ester
Formula C18H34O2, MW 282, CAS# 693-38-9, Entry# 6202
Hexadecanoic acid, ethenyl ester

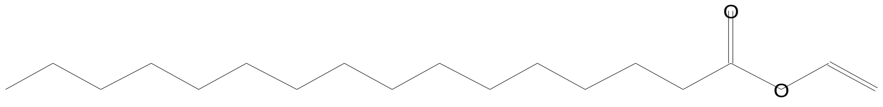

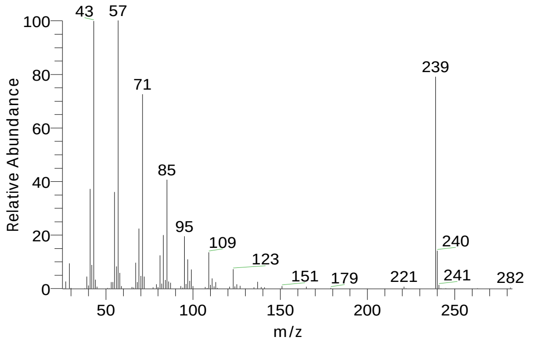

My GC-MS Report

Compound Structure | Hit Spectrum

Palmitic acid vinyl ester
Formula C18H34O2, MW 282, CAS# 693-38-9, Entry# 24552
Hexadecanoic acid, ethenyl ester

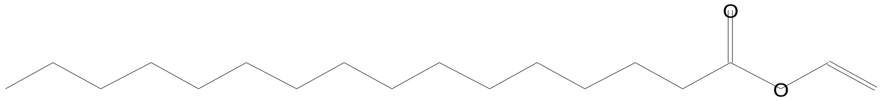

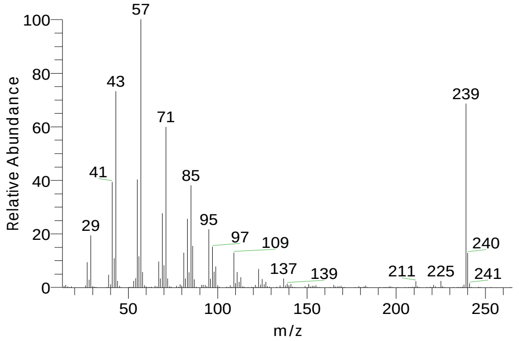

Hexadecanoic acid, 4-nitrophenyl ester
Formula C22H35NO4, MW 377, CAS# 1492-30-4, Entry# 7638
4-Nitrophenyl palmitate #

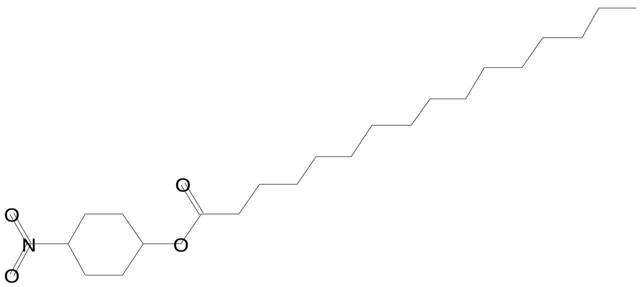

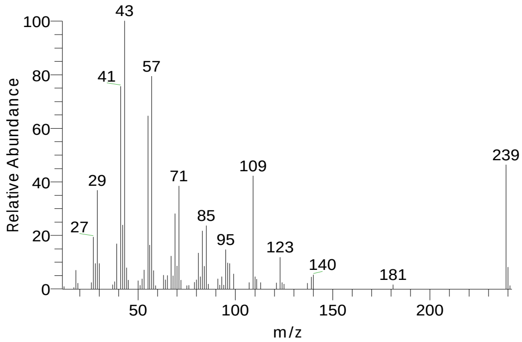

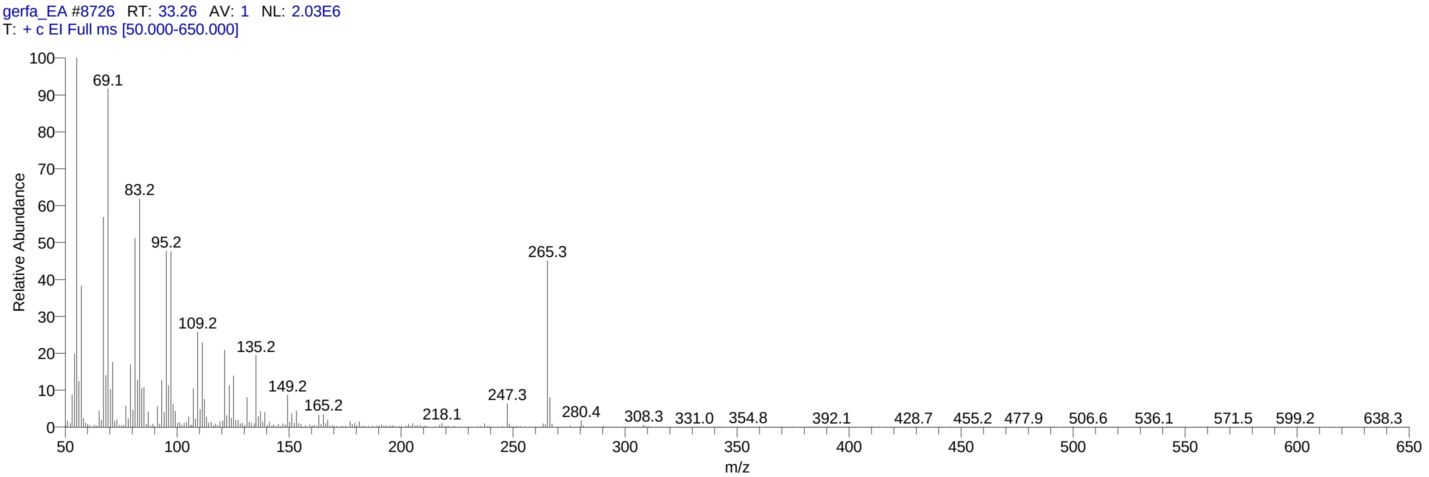

RT	Compound Name	Area %	Molecular Formula	Molecular Weight	Cas #	MF	Library
33.26	E,E,Z-1,3,12-Nonadecatriene-5,14-diol	0.12	C19H34O2	294	NA	720	mainlib
33.26	2,5-FURANDIONE, 3-(DODECENYL)DIHYDRO-	0.12	C16H26O3	266	25377-73-5	797	WileyRegistry8e
33.26	2-Dodecen-1-yl(-)succinic anhydride	0.12	C16H26O3	266	19780-11-1	794	mainlib

My GC-MS Report

Compound Structure | Hit Spectrum

E,E,Z-1,3,12-Nonadecatriene-5,14-diol
Formula C19H34O2, MW 294, CAS# NA, Entry# 21026
(3E,12Z)-1,3,12-Nonadecatriene-5,14-diol #

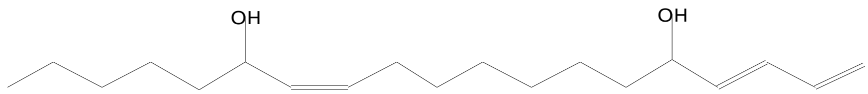

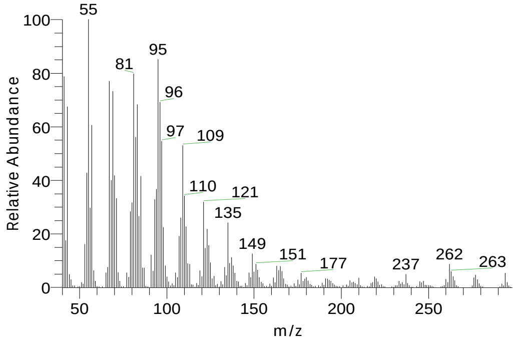

2,5-FURANDIONE, 3-(DODECENYL)DIHYDRO-
Formula C16H26O3, MW 266, CAS# 25377-73-5, Entry# 156913
2,5-FURANDIONE, 3-DODECYL-

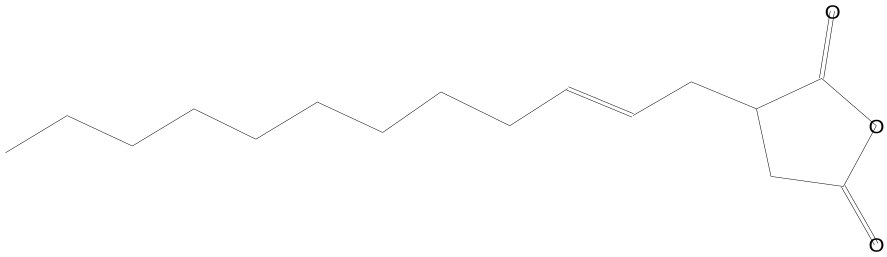

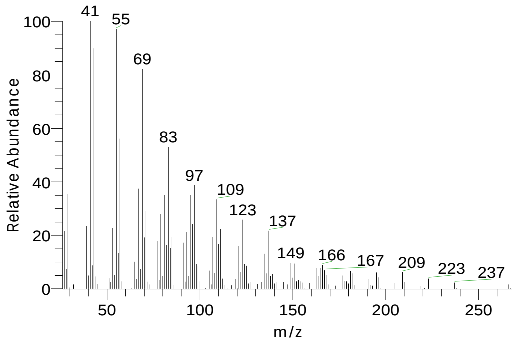

2-Dodecen-1-yl(-)succinic anhydride
Formula C16H26O3, MW 266, CAS# 19780-11-1, Entry# 2686
2,5-Furandione, 3-dodecenyl-

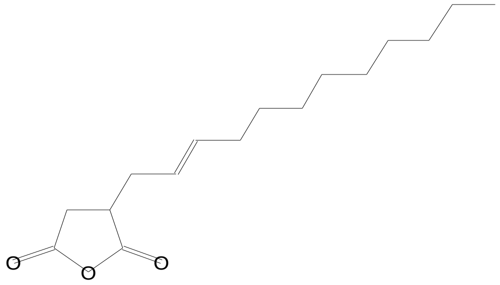

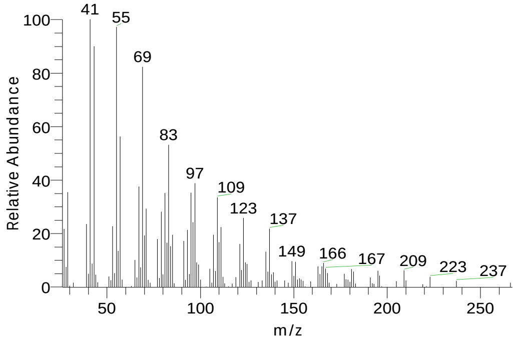

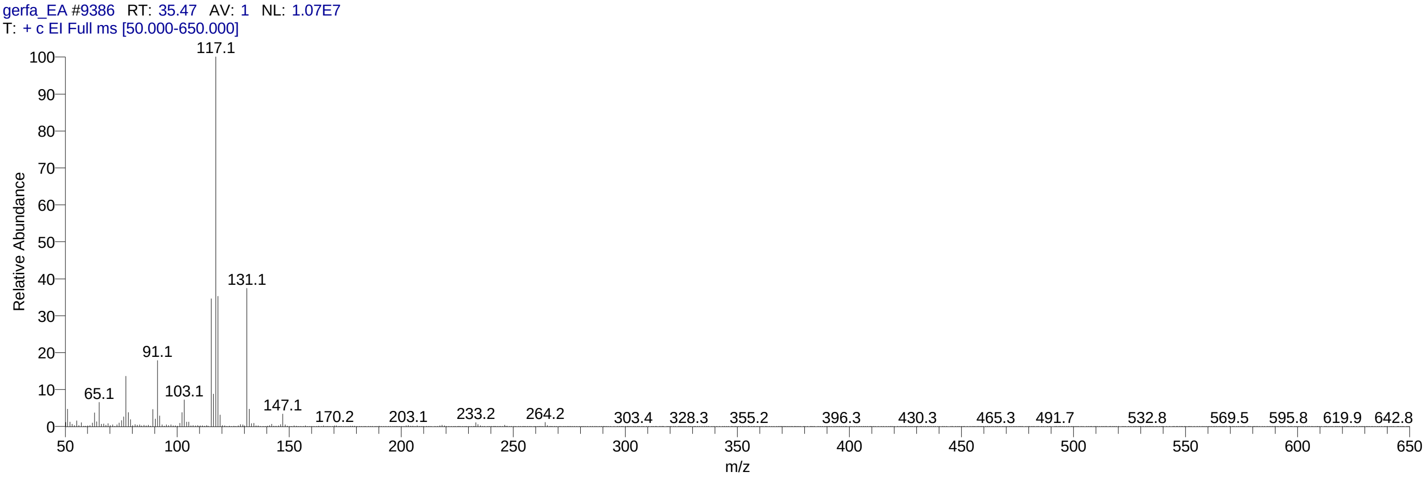

RT	Compound Name	Area %	Molecular Formula	Molecular Weight	Cas #	MF	Library
35.47	(1-METHYLCYCLOPROPYL)BENZENE	0.21	C10H12	132	NA	814	WileyRegistry8e
35.47	DELTACYCLENE	0.21	C9H10	118	7785-10-6	897	WileyRegistry8e

My GC-MS Report

RT	Compound Name	Area %	Molecular Formula	Molecular Weight	Cas #	MF	Library
35.47	Deltacyclene	0.21	C9H10	118	7785-10-6	896	mainlib

Compound Structure	Hit Spectrum

(1-METHYLCYCLOPROPYL)BENZENE
Formula C10H12, MW 132, CAS# NA, Entry# 380177
CYCLOPROPAN, 1-METHYL-1-PHENYL-

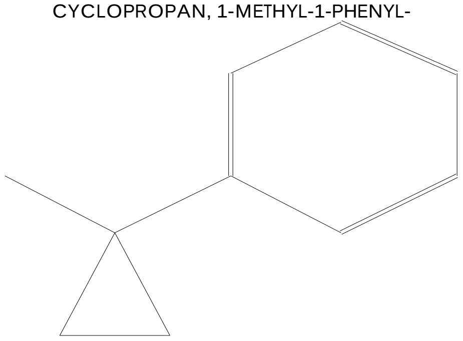

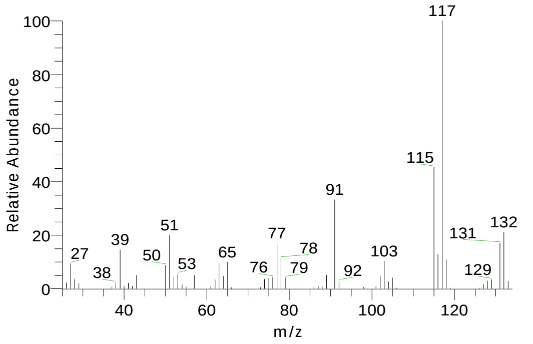

DELTACYCLENE
Formula C9H10, MW 118, CAS# 7785-10-6, Entry# 358032
TETRACYCLO[4,3,0,0(4,9),0(5,7)]NON-2-ENE

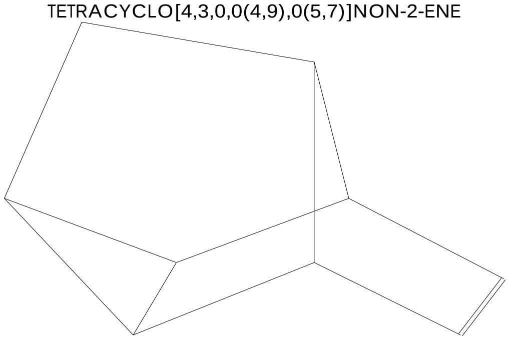

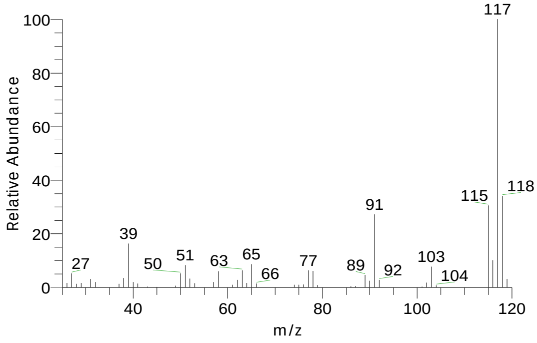

SI 769, RSI 896, mainlib, Entry# 97452, CAS# 7785-10-6, Deltacyclene

Deltacyclene
Formula C9H10, MW 118, CAS# 7785-10-6, Entry# 97452
Tetracyclo[4,3,0,0(4,9),0(5,7)]non-2-ene

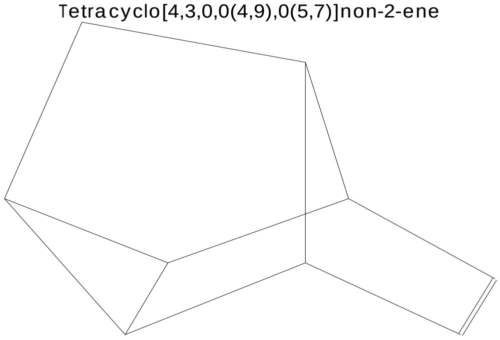

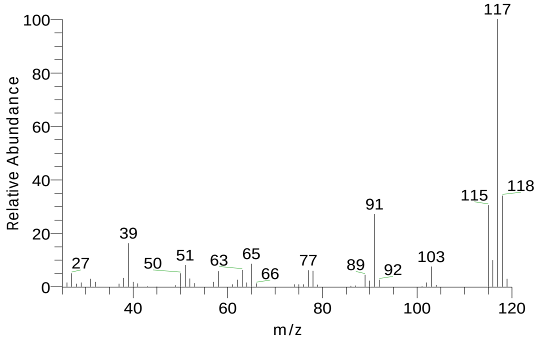

Supplement: Supplementary file 1 [file molecules-25-00996-s001.zip › molecules-727712-SM-final/Figure S2.pdf]
