# Supplementary material for: Phytochemical Characterization and Chemotherapeutic Potential of Cinnamomum verum Extracts on the Multiplication of Protozoan Parasites In Vitro and In Vivo
Source: Molecules. 2020 Feb 24;25(4):996. doi: 10.3390/molecules25040996 (PMC7070835; doi:10.3390/molecules25040996)
Supplement: Supplementary file 1 [file molecules-25-00996-s001.zip › molecules-727712-SM-final/Table S1.pdf]

Table S1. The IC_{50} and selectivity index of diminazene aceturate (DA), methanolic *S. aromaticum* (MESA) and methanolic *O. europaea* (MEOE).

Drug	Parasite	IC_{50} (µg/mL)[a] parasites	EC_{50} (µg/mL)[b]			Selective indices [c]		
			MDBK	NIH/3T3	HFF	MDBK	NIH/3T3	HFF
**DA**	***B. bovis***	0.25 ± 0.02	> 100	> 100	> 100	> 400	> 400	> 400
	B. bigemina	0.11 ± 0.01	> 100	> 100	> 100	> 909	> 909	> 909
	B. divergens	0.35 ± 0.03	> 100	> 100	> 100	> 285.7	> 285.7	> 285.7
	B. caballi	0.003 ± 0.001	> 100	> 100	> 100	> 33333	> 33333	> 33333
	T. equi	0.37 ± 0.01	> 100	> 100	> 100	> 270.3	> 270.3	> 270.3
**MESA**[d]	***B. bovis***	109.8 ± 3.8	894.7± 4.9	>1000	>1000	8.1	> 9.1	> 9.1
	B. bigemina	8.7 ± 0.09	894.7± 4.9	>1000	>1000	102.8	> 114.9	>114.9
	B. divergens	76.4 ± 4.5	894.7± 4.9	>1000	>1000	11.7	> 13.1	> 13.1
	B. caballi	19.6 ± 2.2	894.7± 4.9	>1000	>1000	45.6	> 51.1	> 51.1
	T. equi	60.0 ± 7.3	894.7± 4.9	>1000	>1000	14.9	> 16.7	> 16.7
**MEOE**[e]	***B. bovis***	107.1 ± 9.2	794.7± 41.9	>1500	>1500	7.4	> 14	> 14
	B. bigemina	47.7 ± 2.3	794.7± 41.9	>1500	>1500	16.7	> 31.4	> 31.4
	B. divergens	101.1 ± 8.9	794.7± 41.9	>1500	>1500	7.9	> 14.8	> 14.8
	B. caballi	105.5 ± 11.1	794.7± 41.9	>1500	>1500	7.5	> 14.2	> 14.2
	T. equi	19.3 ± 2.1	794.7± 41.9	>1500	>1500	41.2	> 77.7	> 77.7

[a] Half-maximal inhibitory concentration of extracts on piroplasm parasites *in vitro*. [b] Half-maximal effective concentration of extracts on cell lines. The values were determined from the dose-response curve using non-linear regression (curve fit analysis). The values are the means of triplicate experiments. [c] Ratio of the cell lines EC_{50} to the parasite IC_{50}. High numbers are favorable. [d][7], [e][9].
